# Photogenerated Oxetanes as a Gateway to Uphill Cyclopropanation

**DOI:** 10.1002/anie.3547092

**Published:** 2026-06-26

**Authors:** Tim Köglmeier, Thomas J. Tiefel, Oliver Reiser

**Affiliations:** ^1^ Institut Für Organische Chemie Universität Regensburg Regensburg Germany

**Keywords:** biomass upgrading, cyclopropanes, isomerization, photochemistry

## Abstract

Despite their privileged status in pharmaceutical chemistry, the sustainable synthesis of cyclopropanes remains a fundamental challenge. Conventional methods rely on hazardous diazo precursors or pre‐functionalized substrates that generate stoichiometric byproducts. Here, we report an overall endergonic, catalytic route to cyclopropanes from abundant, renewable starting materials. Furan oxetanes, readily formed by photochemical [2 + 2] cycloaddition of aldehydes and furan, undergo an intramolecular S_N_‐type rearrangement under Lewis acid catalysis, producing cyclopropanes with ideal atom economy. The strategy enables access to *meso*‐cyclopropane dicarbaldehydes, which are versatile intermediates that streamline the preparation of otherwise challenging bioactive motifs. By harnessing photogenerated oxetanes as cyclopropane precursors, this work offers a sustainable way to densely functionalized cyclopropanes from simple feedstocks under mild conditions.

## Graphical abstract

An atom‐economical route to densely functionalized cyclopropanes from furans and photochemically activated aldehydes is enabled by a unique oxetane‐to‐cyclopropane rearrangement. These cyclopropanes can be used to access complex bioactive scaffolds and serve as a platform for asymmetric cyclopropane synthesis.

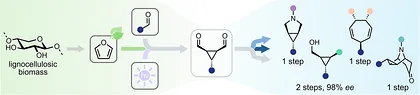

## 1 Introduction

The development of sustainable methods in chemistry is guided by the 12 Principles of Green Chemistry, which provide a framework for reducing environmental impact [1]. In particular, waste prevention, high atom economy, and the use of renewable feedstocks are challenging to implement for transformations that require significant energy input. A prominent example is the cyclopropane ring, a privileged structural motif prevalent in bioactive compounds and synthetic intermediates [2, 3]. Its inherent ring strain [4] makes it difficult to obtain, as its synthesis requires an energy source to overcome the thermodynamic barrier. A common approach involves highly reactive carbene intermediates generated by the decomposition of diazo precursors [5, 6], which have an even higher intrinsic energy than cyclopropanes. However, their explosive nature is problematic for safety and practicality at the process scale [7]. Because of their excellent synthetic and commercial availability, aldehydes have recently been used as a safe, low‐energy starting point for the synthesis of cyclopropanes [8, 9, 10]. Yet, without a diazo compound to act as a traceless carbene source, cyclopropanations with aldehyde‐derived precursors inevitably produce stoichiometric waste. The requirement for high‐energy starting materials or the inevitable production of waste strongly contrasts with the synthesis of cyclobutanes. Despite possessing comparable ring strain, these four‐membered counterparts can be synthesized by photochemical cycloaddition of stable and abundant precursors [4, 11]. To emulate this method, an ideal approach would convert readily available, low‐energy starting materials into cyclopropanes by incorporating light energy. Although some thermal [12, 13] and photoisomerization‐based [14, 15, 16, 17, 18] approaches to cyclopropanes that improve the atom economy of the key step have been reported, they still require multi‐step substrate syntheses, generating overall stoichiometric waste.

Based on these limitations, we envisioned a strategy that bypasses conventional precursors altogether by using light to generate a high‐energy intermediate, which would then undergo isomerization to form the cyclopropane ring with high atom economy. A promising candidate for the first reaction is the Paternò–Büchi reaction, which, like the photochemical cyclobutane synthesis, generates four‐membered rings with perfect atom economy. This photochemical [2 + 2] cycloaddition converts abundant aldehydes and alkenes into oxetanes, which possess ring strain comparable to that of cyclopropanes [4]. Furthermore, their structure features two electrophilic carbons adjacent to oxygen, making them susceptible to nucleophilic attack [19]. Thus, we envisioned that an oxetane with a nucleophilic carbon atom in the *γ* position to the oxetane oxygen atom could undergo an intramolecular nucleophilic substitution (S_N_) reaction to furnish a cyclopropane.

**FIGURE 1:**
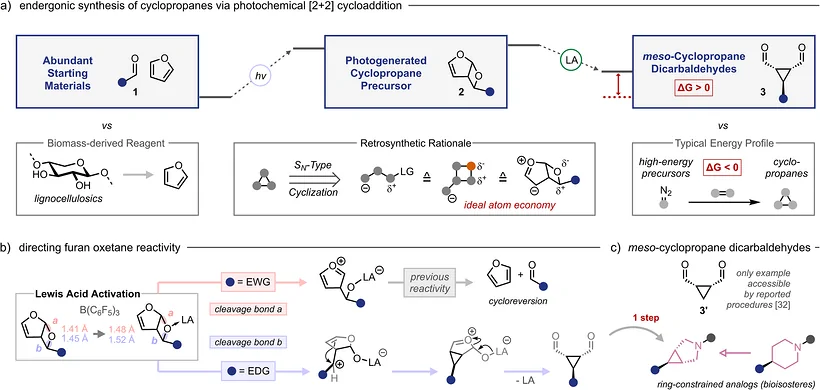
Furan oxetanes as photochemically generated cyclopropane precursors. (a) [2 + 2]‐cycloaddition of an aldehyde with furan as an atom‐economic, endergonic, and renewable gateway to cyclopropane dicarbaldehydes enabled by the ambiphilic properties of furan oxetanes. (b) Lewis acid (LA) catalysis activates both oxetane C–O bonds. Selective cleavage of the desired bond is enabled by attachment of an electron‐donating group. (c) Rearrangement of furan oxetanes affords previously elusive *meso*‐cyclopropane dicarbaldehydes, which facilitate the synthesis of pharmaceutically relevant structures.

Among the plethora of possible oxetanes, we chose furan oxetanes to test the feasibility of this approach (Figure 1a). This choice was motivated by their convenient and efficient production from aldehydes and furan, as reported in synthetic and mechanistic studies by Toki [20, 21], Griesbeck [22, 23, 24, 25], and others [26, 27, 28, 29], with quantum yields of up to 39% reported for this transformation [30]. Furthermore, furan oxetanes contain a nucleophilic site in the desired position, in the form of an enol ether double bond, and generally exhibit good bench stability [26]. Additionally, furan is considered a key renewable platform chemical [31], making it a green reagent that aligns perfectly with the sustainable goals of this approach. Finally, we hypothesized that unlocking S_N_‐type cyclization reactivity in furan oxetanes would provide access to substituted meso‐cyclopropane dicarbaldehydes. This specific scaffold remains elusive despite being an appealing building block for introducing three‐dimensional complexity into molecules [32, 33]. While routes to cyclopropane monocarbaldehydes [34, 35] and of a limited number to trans‐dicarbaldehydes [36, 37] are known, general methods for preparing their substituted meso counterparts are still missing, making these structures difficult to obtain via conventional approaches.

## 2 Results and Discussion

### 2.1 Reaction Development

Since oxetanes are relatively inert toward nucleophiles [38], we reasoned that forcing the reactivity of furan oxetanes toward S_N_‐type cyclization would require additional activation of the oxetane C–O σ‐bond. Brønsted or Lewis acid catalysis can be employed to withdraw electron density, thus activating the C–O σ‐bonds and increasing the carbon's electrophilicity [39, 40]. Existing literature, however, reveals that under exposure to acids, furan oxetanes exhibit a tendency to restore the aromaticity of the furan ring. This proceeds by cleavage of the oxetane C–O σ‐bond adjacent to the five‐membered ring (Figure 1b, bond a), leading to either cycloreversion [41] or elimination [42]. Cleavage of the desired oxetane C–O σ‐bond (Figure 1b, bond b) was reported only when two oxetane bonds were cleaved simultaneously, resulting in a carbonyl−olefin metathesis that occurred as a side‐reaction on silica [42] or under photochemical [26, 27] conditions. We reasoned that an electron‐donating group would be needed to stabilize the emerging cationic character and enable S_N_‐type reactivity (Figure 1b). This approach would give access to cyclopropanes bearing electron‐donating groups, complementing the vast number of cyclopropanes obtained from diazo precursors stabilized by electron‐withdrawing groups.

To test this hypothesis, we investigated phenyl‐substituted furan oxetane **2a** and para‐methoxyphenyl‐substituted furan oxetane **2b** with different acids (for gram‐scale syntheses of **2a** and **2b**, see the Supporting Information [SI]). While exposure of **2a** to trifluoroacetic acid only led to the previously known cycloreversion, treating **2b** with this acid indeed produced traces of the desired *meso*‐cyclopropane dicarbaldehyde **3b**. Optimization revealed that different Lewis acids promote the reaction even more successfully than Brønsted acids, and the strong Lewis acid B(C_6_F_5_)_3_ in chloroform proved optimal (for details see the SI). While large amounts of water or bases negatively influenced the reaction, small amounts of weak bases like sodium acetate trihydrate boosted the yield of **3b** to 90%. Under these improved conditions, even phenyl furan oxetane **2a** produced cyclopropane **3a** in a 33% yield. In all cases, only the diastereomer bearing the aryl substituent trans to both carbaldehyde groups was formed. Reduction with sodium borohydride yielded the cyclopropane diol **4b**, the structure of which was determined by x‐ray crystallography.

### 2.2 Substrate Scope

We subsequently explored the scope of our protocol in a two‐step approach: As the first step, an aldehyde was irradiated in neat furan, followed by removal of the excess furan by distillation. In the second step, the crude furan oxetane was subjected to the optimized rearrangement conditions, which afforded yields comparable to those obtained with the purified furan oxetanes. Because 1,4‐dialdehydes are known to undergo reversible polymerization at elevated concentrations, [43] we employed quantitative ^1^H NMR spectroscopy against an internal standard to accurately quantify the monomeric dialdehyde products.

Initially focusing on the electron‐donating effect of the aryl substituents, we found that meta‐ and ortho‐substitution with a methoxy group produced the corresponding cyclopropanes in yields of 28% (**3c**) and 87% (**3d**), respectively, which is consistent with the substituents’ electronic properties [44] and further emphasizes the critical role of cation stabilization at the benzylic position. Following this trend, a wide range of electron‐rich aryl aldehydes produced *meso*‐cyclopropane dicarbaldehydes in high to excellent yields, and notably, the 3,4‐dimethylfuran‐derived product **3r** was also obtained in good yield (Table 1). Conversely, electron‐withdrawing substituents in the aryl moiety significantly reduced cyclopropane yields in favor of conversion to the starting aldehydes and furan.

**TABLE 1:** Synthesis of meso‐cyclopropane dicarbaldehydes.

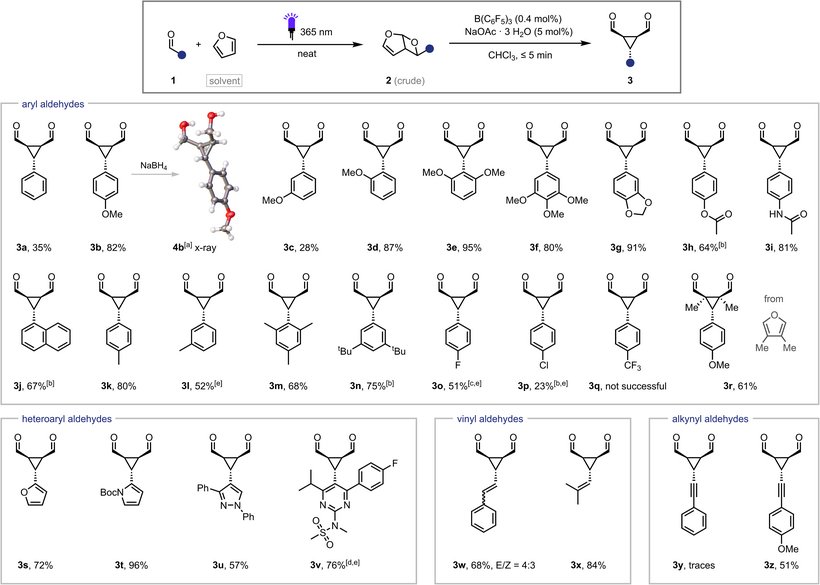

*Note*: 0.5 mmol aldehyde in neat furan (reagent and solvent, 5 mL, 59 mmol, 135 equiv.); irradiation (365 nm, 1.5–6 W) under nitrogen at 19°C for 1.5–19 h. Crude furan oxetanes, NaOAc·3H_2_O (5 mol%), and B(C_6_F_5_)_3_ (0.4 mol%) in CHCl_3_ (5 mL) were stirred at 25°C until full conversion. Yields were determined via ^1^H NMR spectroscopy with 1,3‐dinitrobenzene as internal standard.

^a^Crude **3b** obtained from the rearrangement of pure **2b** was treated with NaBH_4_ (2 equiv) in MeOH at 0°C, which afforded **4b** in 87% yield relative to **2b**.

^b^B(C_6_F_5_)_3_ loading of 0.6–0.8 mol%.

^c^B(C_6_F_5_)_3_ loading of 1.5 mol%.

^d^B(C_6_F_5_)_3_ loading of 4.0 mol%.

^e^Increased rearrangement times of up to 40 min.

The reaction sequence was successfully applied also to heterocyclic aldehydes, providing access to the pharmaceutically important heteroaryl substituted cyclopropanes [2, 10], including furan, pyrrole, pyrazole, and pyrimidyl groups. For electron‐rich heteroaryl derivatives **3s–u**, the rearrangement proceeded faster compared to electron‐rich aryl aldehydes and led to yields up to 96%. Starting from the densely functionalized pyrimidine aldehyde **1v**, a rosuvastatin precursor, cyclopropane **3v** was produced in good yield, demonstrating the potential of this method for late‐stage functionalization. Beyond aryl and heteroaryl aldehydes, an extension to conjugated alkenyl and alkynyl aldehydes was possible provided that electron‐donating substituents are present (**3w–z**). Expansion of the scope to non‐conjugated aldehydes was not successful, as the butyraldehyde‐derived oxetane reverted exclusively to its starting materials. The rearrangement reactions generally proceeded swiftly, with few substrates taking longer than 5 min to reach full conversion. Some particularly electron‐rich substrates achieved full conversion after less than 1 min, while longer reaction times led to product degradation. In contrast, substrates with electron‐poor substituents required longer reaction times (up to 40 min, SI).

### 2.3 Applications

**FIGURE 2:**
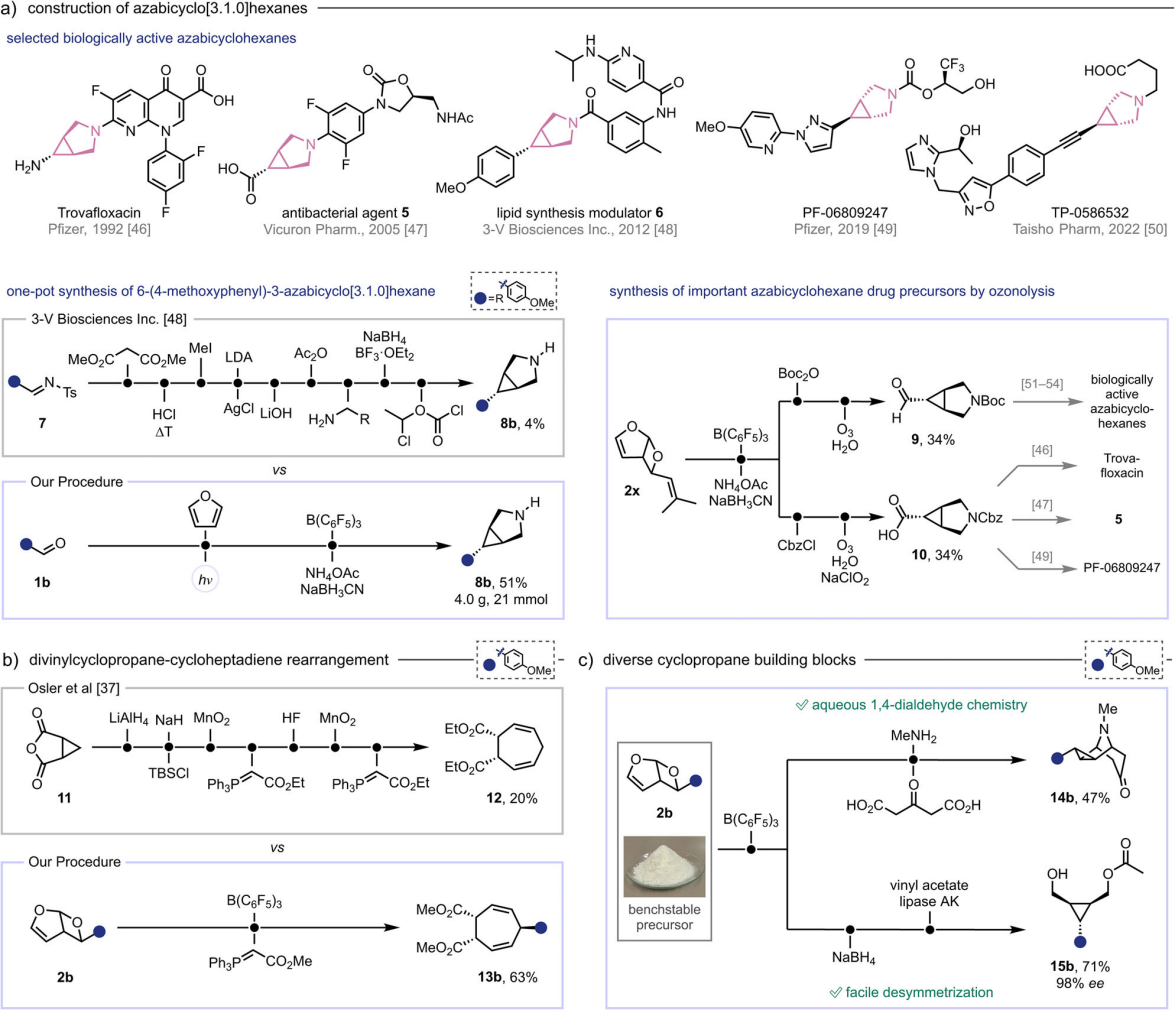
Streamlined synthesis of cyclic compounds using furan oxetanes as cyclopropane precursors. (a) One‐pot syntheses of pharmaceutical azabicyclohexanes, bearing the challenging aryl substituent (left), carboxyl, or carbaldehyde functionalities (right). (b) One‐pot synthesis of a cycloheptadiene. (c) Synthesis of diverse cyclopropane building blocks by subjecting the cyclopropane dicarbaldehyde to an aqueous Robinson Schöpf cyclization (top) or reduction followed by asymmetric alcohol protection (bottom).

To establish furan oxetanes as practical cyclopropane precursors, we selected pharmaceutically relevant structures that are challenging to synthesize using established methods. For this purpose, selected furan oxetanes were synthesized at a multi‐gram scale (12–18 g, for details see the SI). Ring‐constrained analogs are common in drug design to rigidify pharmacophoric orientations [45], and fused cyclopropanes, especially 3‐azabicyclo[3.1.0]hexanes, are present in many active pharmaceutical ingredients (Figure 2a, top) [46, 47, 48, 49, 50, 51, 52, 53, 54, 55, 56]. Although cyclopropanation of 3‐pyrroline or maleimide substrates with carbenes is well‐established [57], accessing 3‐azabicyclo[3.1.0]hexanes bearing aryl‐ and other electron‐donating substituents remains challenging [58]. Reported syntheses for these analogues require multiple, redox‐heavy steps [48, 59]. In contrast, starting with anisaldehyde, we prepared the previously difficult‐to‐obtain 3‐azabicyclo[3.1.0]hexane **8b** at a multi‐gram scale via a photoreaction with furan, Lewis acid rearrangement, and subsequent in situ reductive amination (Figure 2a, left). Additionally, we obtained the established drug precursors **9** [45, 46, 48, 49, 56] and **10** [51, 52, 53, 54, 55] from furan oxetane **2x** via a sequence of rearrangement, reductive amination, protection, and ozonolysis. This route bypasses the conventional diazoacetate cyclopropanation, which suffers from poor diastereoselectivity and requires several subsequent steps to obtain the aldehyde and carboxylic acid functionalities [58, 60].

Furthermore, we envisioned that the direct access to *meso*‐cyclopropane dicarbaldehydes demonstrated by our approach could streamline the synthesis of important carbocyclic systems. An example can be found in the preparation of seven‐membered rings, which are present in a range of important natural products and bioactive compounds [61]. A promising approach to obtain unsaturated variants, specifically cycloheptadienes, involves the thermal rearrangement of 1,2‐divinylcyclopropanes [62]. In a previous synthesis, reported by Osler et al. [37], a meso‐divinylcyclopropane intermediate was prepared from cyclopropane **11** via a seven‐step reaction sequence of alternating oxidations and Wittig olefinations (Figure 2b), producing cycloheptadiene **12**. This route was necessary because *meso*‐cyclopropane dicarbaldehydes cannot be directly obtained by oxidation of 1,2‐bis(hydroxymethyl) cyclopropanes [32, 37]. In comparison, we achieved the one‐pot synthesis of cycloheptadiene **13b** in 63% yield via the rearrangement of furan oxetane **2b**, followed by the addition of a phosphorus ylide.

The synthetic versatility of our approach was further demonstrated by the rapid construction of the complex synthetic alkaloid **14** (Figure 2c). Using a conventional Robinson–Schöpf condensation [63], we obtained the desired tricycle in 47% yield by adding the crude **3b** to an aqueous acidic medium containing the reagents. This procedure provides convenient access to a ring‐constrained pseudopelletierine scaffold, which is found in various bioactive compounds [64]. To demonstrate the potential of the *meso*‐cyclopropanes obtained from furan oxetanes as a starting point for asymmetric synthesis, the rearrangement step was followed by a reduction. This gives facile access to the novel cyclopropane diol **4b**, which can be easily desymmetrized by enzymatic alcohol protection using Lipase AK obtained from *Pseudomonas fluorescens*, yielding mono‐acetate **15b** in 73% over two steps with an enantiomeric excess of 98%.

### 2.4 Mechanistic Investigations

**FIGURE 3:**
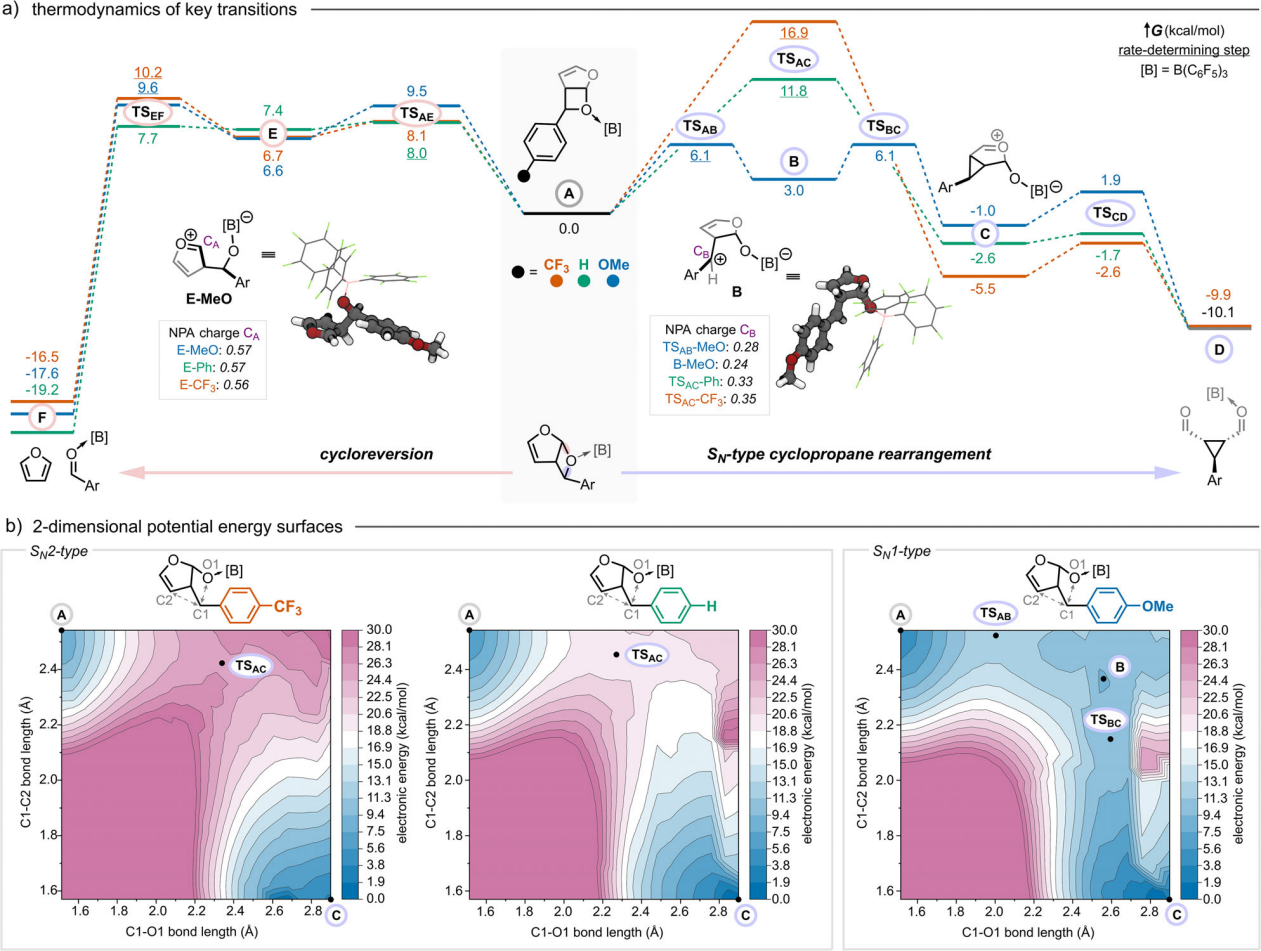
Mechanistic Investigations. (a) Thermodynamics of the two possible Lewis acid‐catalyzed reactions of furan oxetanes **2a**, **2b**, and **2q**, leading to cycloreversion (left) and cyclopropane formation (right), obtained at ωB97M‐V/def2‐QZVPP@SMD(CHCl_3_)//PBEh‐3c@CPCM(CHCl_3_) level of theory. (b) Two‐dimensional relaxed potential energy surface scans of the transition from different oxetanes to cyclopropanes, obtained at PBEh‐3c@CPCM(CHCl_3_) level of theory.

We next sought to understand the underlying reaction mechanism and validate our original retrosynthetic rationale. Our experiments revealed that this transformation involves two competing pathways: a formal cycloreversion, which regenerates the starting materials, and the targeted rearrangement to cyclopropane dicarbaldehydes, with the outcome strongly dependent on the electronic properties of the aryl substituent. To rationalize the observed selectivity, we calculated energy profiles for both pathways across representative substrates with different electronic properties (Figure 3a). In all cases, coordination of the Lewis acid to the oxetane oxygen atom activates both C–O bonds, as indicated by a 4–5% elongation (approximately 0.07 Å). The subsequent trajectories are determined by the C–O bond that is cleaved, leading either to cycloreversion or cyclopropane formation. The cycloreversion pathway proceeds via two steps, beginning with the opening of the respective oxetane C–O bond. This barrier is largely unaffected by substituent effects, as the cationic character emerging in the transition state and intermediate is mainly stabilized by the neighboring oxygen atom. In contrast, the cyclopropane‐forming rearrangement shows a pronounced substituent dependence. For more electron‐withdrawing aryl substituents, the transition from oxetane to cyclopropane is concerted and resembles an intramolecular S_N_2‐type displacement. However, for a strongly electron‐withdrawing aryl substituent, like (*p*‐CF_3_‐phenyl), the activation barrier of the rearrangement pathway exceeds that of cycloreversion, making the latter the exclusive outcome. For a phenyl substituent, the barrier for cyclopropane formation decreases to a level competitive with that for cycloreversion. For a more electron‐donating aryl substituent (*p*‐OMe‐phenyl), this barrier is lowered even further, and the mechanism from oxetane to cyclopropane switches to a stepwise pathway. The C–O bond cleavage forms a betaine intermediate containing a benzylic carbocation that is attacked by the enol ether double bond of the furan ring, overall resembling an intramolecular S_N_1‐reaction (Figure 3b). Analysis of Natural Population Analysis (NPA) charges confirms that the more electron‐donating residue decreases the emerging benzylic cationic charge during the cyclopropane‐yielding transition, while the emerging cationic charge during cycloreversion is largely unaffected by the different aryl substituents.

Finally, it is noteworthy that the cyclopropane dicarbaldehyde product exhibits a higher energy level than the starting materials furan and aldehyde, making the light‐ and Lewis acid‐mediated tandem reaction involving the oxetane‐to‐cyclopropane rearrangement endergonic with a positive Gibbs free energy of approximately 13–15 kcal/mol. This confirms that the light energy is not only used to drive the transformation but also partially stored within the strained cyclopropane products.

## 3 Conclusion

In conclusion, this work establishes that the Lewis acid‐catalyzed rearrangement of furan oxetanes, obtained by irradiation of aldehydes in furan, provides broad access to novel substituted *meso*‐cyclopropane dicarbaldehydes, expanding the toolbox of non‐conventional building blocks [65] for cyclopropane synthesis. The rearrangement shows good general feasibility across aryl, heteroaryl, vinyl, and alkynyl aldehydes with yields benefiting from the electron‐donating properties of the substituent, a trend rationalized by density functional theory (DFT) calculations that elucidate the underlying mechanism. This combined strategy enables the endergonic synthesis of cyclopropanes with perfect atom economy. We expect that the introduction of oxetanes as photogenerated precursors to cyclopropanes will impact safety, sustainability, and practicability of cyclopropane synthesis.

## Author Contributions

**Tim Köglmeier**: conceptualization, methodology, formal analysis, visualization, writing – original draft, writing – review and editing, investigation. **Thomas J. Tiefel**: conceptualization, formal analysis, visualization, writing – original draft, writing – review and editing, investigation. **Oliver Reiser**: conceptualization, writing – review and editing, writing – original draft, funding acquisition, supervision.

## Conflicts of Interest

The authors declare no conflicts of interest.

## Supporting information




**Supporting File**: The authors have cited additional references within the Supporting Information [20, 21, 22, 23, 24, 25, 26, 27, 28, 29, 30, 46, 66, 67, 68, 69, 70, 71, 72, 73, 74, 75, 76, 77, 78, 79, 80, 81, 82].

## Data Availability

The data that supports the findings of this study are available in the Supporting Information of this article.
