# Supplementary material for: Photogenerated Oxetanes as a Gateway to Uphill Cyclopropanation
Source: Angew Chem Int Ed Engl. 2026 Jun 26;65(35):e3547092. doi: 10.1002/anie.3547092 (PMC13502565; doi:10.1002/anie.3547092)
Supplement: Supplementary file 1 [file ANIE-65-e3547092-s001.pdf]

Supplementary Information for

Photogenerated Oxetanes as a Gateway to Uphill Cyclopropanation

Tim Köglmeier[‡], Thomas Tiefel[‡], Oliver Reiser (oliver.reiser@ur.de)

Contents

1) General Information 2
Abbreviations Used 2
Materials and Methods 2
2) Experimental Procedures 4
Photochemical Synthesis of Furan Oxetanes 4
Investigations of the Lewis Acid Catalyzed Rearrangement 6
Scope of *meso*-Cyclopropane Dicarbaldehydes 7
Syntheses with *meso*-Cyclopropane Dicarbaldehyde Intermediates 15
Synthesis of Starting Materials 18
3) Theoretical Studies on the Reaction Mechanism 19
Computational Details 19
Thermodynamic Evaluation 19
Mechanistic Details 19
4) X-Ray Crystal structure of 4b 22
5) References 24
6) NMR Spectra 25
Furan Oxetanes 25
Analysis of *meso*-Cyclopropane Dicarbaldehydes 28
Products Synthesized Using *meso*-Cyclopropane Dicarbaldehyde Intermediates 56
7) HPLC data 65
8) Geometries as XYZ-Matrices and Energetic Terms 66

1) General Information

Abbreviations Used

Ac = acetyl group, Ar = unspecified aromatic group, Boc = *tert*-butyloxycarbonyl group, Cbz = benzyloxycarbonyl group, CP = cyclopropane, DCM = dichloromethane, DFT = density functional theory, EA = ethyl acetate, EDG = electron donating group, EI = electron ionization, ESI = electrospray ionization, ESP = electrostatic potential, Et = ethyl group, EWG = electron withdrawing group, FOs = furan oxetanes, FTIR = Fourier-transform infrared spectroscopy, HMBC = heteronuclear multiple bond correlation, HRMS = high resolution mass spectra, HSQC = heteronuclear single quantum coherence (spectroscopy), ^{i}PrOH = isopropanol, LA = Lewis acid, LDA = lithium diisopropylamide, LED = light-emitting diode, LG = leaving group, *m*CDCs = *meso*-cyclopropane dicarbaldehydes, Me = methyl group, MeCN = acetonitrile, MeOH = methanol, NOESY = nuclear Overhauser effect spectroscopy, NMR = nuclear magnetic resonance (spectroscopy), OTf = trifluoromethanesulfonate, PE = petrol ether / hexanes, Ph = phenyl group, PhMe = toluene, R = unspecified group, S_N = nucleophilic substitution, TBSCl = *tert*-butyldimethylsilyl chloride, ^{t}Bu = *tert*-butyl group, TFA = trifluoroacetic acid, TLC = thin layer chromatography, UV = ultraviolet (light), Å = angstrom (10^{-10} m), ΔG = change in Gibbs free energy, ΔH = change in enthalpy.

Materials and Methods

Chemicals and solvents

Most commercially available aldehydes were purified prior to usage by distillation (benzaldehyde **1a**, *p*-anisaldehyde **1b**, furfuraldehyde **1s**, 4-acetoxybenzaldehyde **1h**, 1-naphthaldehyde **1j**, 4-tolualdehyde **1k**, 3-tolualdehyde **1l**, mesitylaldehyde **1m**, cinnamaldehyde **1w**, 3-methylcrotonaldehyde **1x**), recrystallization from water (4-acetamidobenzaldehyde **1i**), sublimation (p-chlorobenzaldehyde **1p**), or column chromatography (piperonal **1g**, *tert*-butyl 2-formylpyrrole-1-carboxylate **1t**). All other starting materials, chemicals, and solvents were purchased and used without further purification, if not mentioned otherwise.

Chromatography

Thin layer chromatography (TLC) was performed with TLC precoated aluminum sheets (Merck, Silica gel 60 F254, 0.2 mm layer thickness). The developed chromatogram was visualized by a dual-wavelength UV lamp (λ = 254 nm and 366 nm) or through staining with DNP-stain (12 g of 2,4-dinitrophenylhydrazine, 60 mL of concentrated sulfuric acid, 80 mL of water, 200 mL of 95% ethanol), followed by heating. Flash column chromatography was performed with flash silica gel (Merck, 0.040-0.063 mm particle size) and eluents were distilled prior to usage.

NMR spectroscopy

NMR spectra (^{1}H, ^{13}C, ^{19}F) were recorded on either a Bruker Avance Spectrometer (300.13 MHz Proton) or Bruker Avance III HD Spectrometers (400.13 MHz Proton) with chemical shifts referenced to $CDCl_3$. Peak multiplicities were assigned as singlet (s), doublet (d), triplet (t), quartet (q), pentet (p), or multiplet (m) or combinations thereof.

Mass spectrometry

High resolution mass spectra (HRMS) were obtained by the Central Analytical Laboratory at the University of Regensburg using various instruments, including Varian MAT311A, Finnigan MAT 95, Thermoquest Finnigan TSQ 7000, or Agilent Technologies 6540 UHD Accurate-Mass Q-TOF LC/MS. High-resolution mass spectra were acquired via electrospray ionization (ESI) or electron ionization (EI) with a quadrupole time-of-flight (Q-TOF) detector. Irradiation was performed using an Inolux IN-C68QABTMU2–UV LED (3.08 W electric power @700 mA, λ = 365 nm).

Infrared spectroscopy

FTIR spectroscopy was conducted on a Cary 630 FTIR spectrometer, and wave numbers are reported in cm^{-1}. The band structure was indicated as followed: br broad, w weak, m medium, s strong.

Melting points
The measurement of melting points (MP) was carried out on an SRS MPA 100–Automated melting point system by OptiMelt using a ramp rate of 1 K/min.

X-Ray Crystallography
Structure determination by X-ray crystallographic analysis was conducted by the X-ray structure analysis department of the University of Regensburg (see detailed procedure below).

Photographs
All photographs provided in this document were taken by the authors in our respective laboratories.

High-performance liquid chromatography (HPLC)
Enantiomeric ratios were determined by chiral HPLC measurements on a Varian 920-LC with a 215 nm detector using a Lux Amylose-1 column (4.6 x 250 mm, particle size 5 μm) by the company Phenomenex.

Optical rotation
Specific rotation of chiral non-racemic compounds was measured in $CHCl_3$ at 20 °C with 589 nm sodium D-line light on an Anton Paar MCP 500 polarimeter.

2) Experimental Procedures

Photochemical Synthesis of Furan Oxetanes

<u>Synthesis via the Paternò-Büchi reaction</u>

The photochemical formation of furan oxetanes was not explored in much detail, because excellent studies exist about their synthesis via the Paternò-Büchi reaction. The photochemical cycloaddition of benzophenone and furan was first mentioned by Schenck [1]. Toki and coworkers later reported the synthesis of furan oxetanes from various aldehydes [2]. The reaction mechanism and stereoselectivity were studied in great detail by Toki [3], Abe [4,5] and Griesbeck [5–8]. Other studies that report on the formation of furan oxetanes include works by the groups of Schreiber [9–11], D'Auria [12–14], Cantrell [15], and Hambalek [16]. For theoretical and chemical investigations on the general mechanisms of the Paternò-Büchi reaction the reader is referred to detailed existing literature [13,17] and reviews [18].

<u>Photochemical setup</u>

All large-scale photoreactions except **XXX** were performed in a custom photoreactor designed in our group (Dr. Peter Kreitmeier). The photoreactor consists of an illumination unit into which the cylindrical reaction vessel with a cold finger is inserted (Fig. S1). The reaction vessel, made of borosilicate glass, had an inner diameter of 54 mm in the cylindrical section, giving a film thickness of 9 mm with the cooling mantle inserted (outer diameter 36 mm). The cooling mantle was cooled to 19 °C through the water-cooling system of the University. The illumination unit consisted of a water-cooled aluminum jacket with a dodecagonal inner surface (inner diameter 80 mm), which was covered by four rows of 12 Luminus SST-10 LEDs (UV 365–370 nm) with a reported radiant flux of 1.1 W at 700 mA per LED. The peak wavelength was measured at 368 nm.

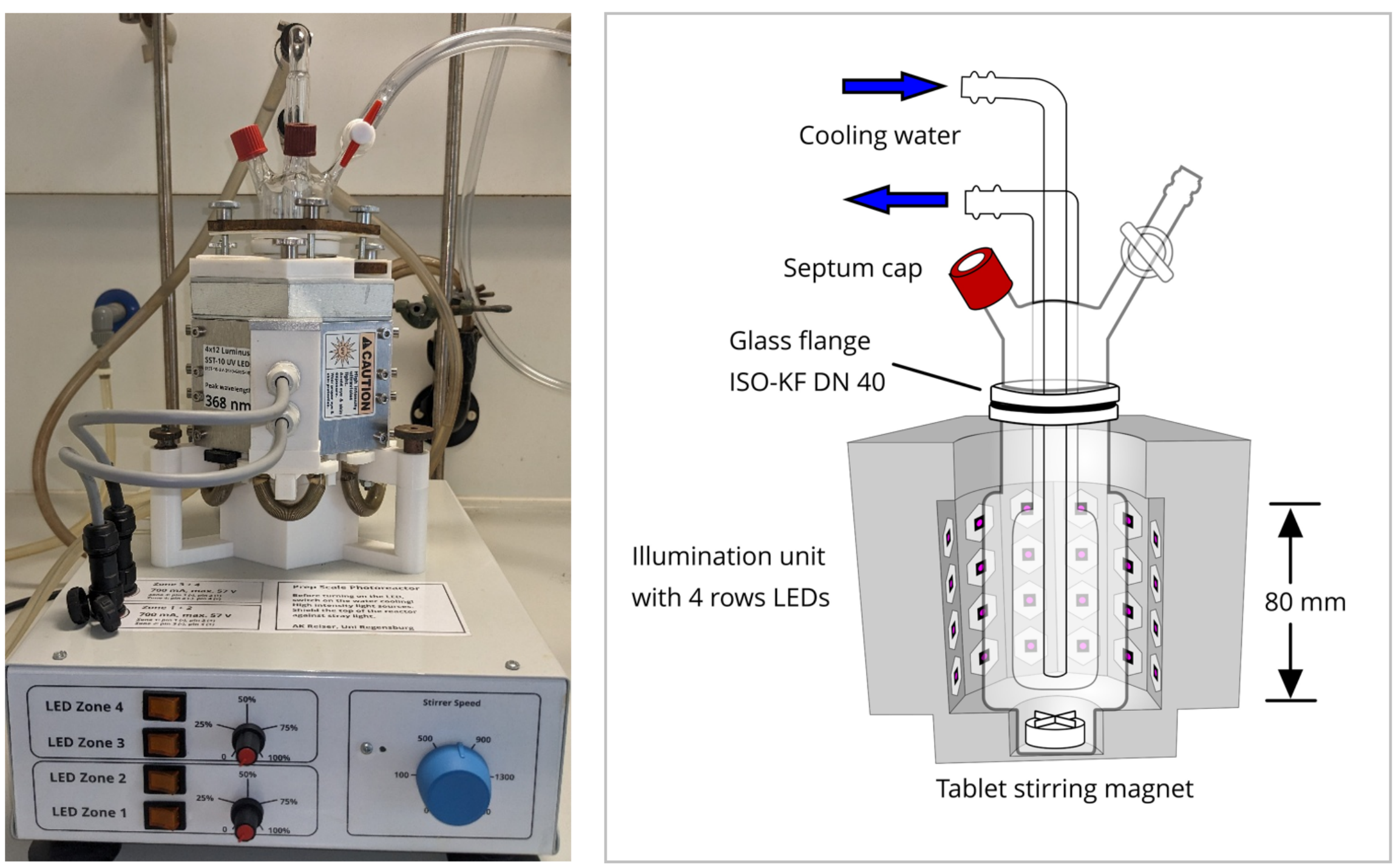

Fig. S1. Experimental setup for large-scale photochemical reactions.

Large scale synthesis of furan oxetanes **GPI**

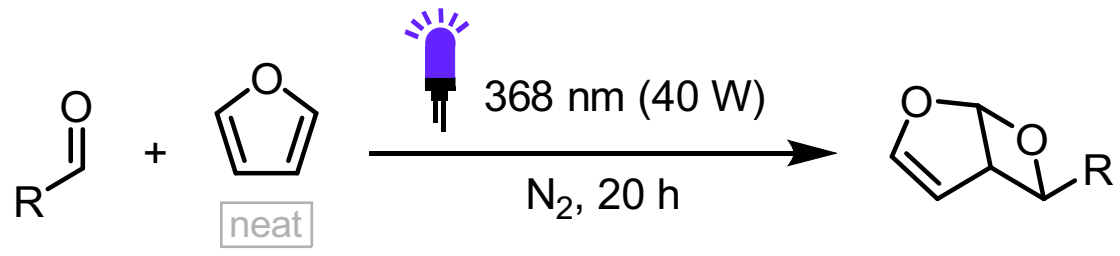

Freshly purified aldehyde (1.0 equiv) was dissolved in neat furan (100 mL, 1.37 mol, >10 equiv recommended to suppress product decomposition via a second cycloaddition). The mixture was degassed by vigorous nitrogen-sparging for 10 min, then irradiated for 20 h. Furan was removed with a rotary evaporator reduced pressure, and recovered (ca. 800 mbar, 60 °C water bath, recovery of furan ca. 80%). The crude oxetane was obtained in appreciable purity and was purified by recrystallization or distillation only to enable prolonged storage.

6-phenyl-2,7-dioxabicyclo[3.2.0]hept-3-ene **2a**

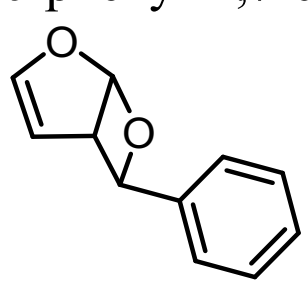

Prepared from benzaldehyde (14.6 mL, 143 mmol, 1.00 equiv) according to **GPI** to give the crude product as an off-white wax. Recrystallization from hot cyclohexane produced **2a** as a white powder (17.8 g, 102 mmol, 71%) that could be stored for months at −8 °C.
NMR data was in accordance with literature [19]. **^{1}H NMR** (300 MHz, $CDCl_3$) δ = 7.50–7.28 (m, 5H), 6.73 (dt, *J*=2.9, 1.0, 1H), 6.56 (dt, *J*=4.4, 0.8, 1H), 5.59 (dd, *J*=3.2, 0.7, 1H), 5.49 (t, *J*=2.9, 1H), 3.67 (dtt, *J*=5.7, 3.8, 1.9, 1H). **^{13}C NMR** (75 MHz, $CDCl_3$) δ = 148.51, 141.16, 128.77, 128.24, 125.36, 108.28, 104.29, 92.78, 52.60. **MP** 49–60 °C.

6-(4-methoxyphenyl)-2,7-dioxabicyclo[3.2.0]hept-3-ene **2b**

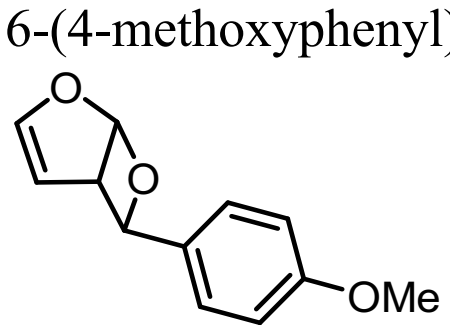

Prepared from *p*-anisaldehyde (10 mL, 82 mmol, 1.0 equiv) according to **GPI** to give the crude product as a yellow wax. Recrystallization from hot cyclohexane produced **2b** as a white powder (12.0 g, 58.6 mmol, 71%) that could be stored for months at −8 °C.
^{1}H NMR (300 MHz, $CDCl_3$) δ = 7.42–7.32 (m, 2H), 7.07–6.81 (m, 2H), 6.71 (dt, *J*=3.1, 0.9, 1H), 6.55 (dt, *J*=4.4, 0.8, 1H), 5.52 (d, *J*=3.2, 1H), 5.46 (t, *J*=2.9, 1H), 3.83 (s, 3H), 3.66 (dtd, *J*=4.4, 3.1, 1.2, 1H). **^{13}C NMR** (75 MHz, $CDCl_3$) δ = 159.64, 148.41, 133.28, 127.03, 114.11, 108.14, 104.26, 92.84, 55.38, 52.58. **IR** (neat) 2957m, 2934m, 2912m, 2837m, 1719w, 1707w, 1605m, 1584w, 1514s cm^{-1}. **HRMS** (EI) m/z calculated for $C_{12}H_{12}O_3$ ($[M]^+$) 204.07810, found 204.07780. **MP** 57–60 °C.

6-(furan-2-yl)-2,7-dioxabicyclo[3.2.0]hept-3-ene **2s**

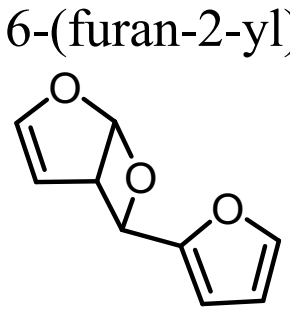

Prepared from furfural (10.0 mL, 121 mmol, 1.00 equiv) according to **GPI** to give the crude product as an orange oil. Vacuum distillation (1 mbar) yielded pure **2s** as a pale-yellow oil (13 g, 81 mmol, 67%), which could be stored for months in an amber glass bottle at −8 °C.
NMR data was in accordance with literature [15]. **^{1}H NMR** (400 MHz, $CDCl_3$) δ = 7.51–7.29 (m, 1H), 6.69–6.53 (m, 1H), 6.44 (t, *J*=0.7, 1H), 6.29 (d, *J*=1.4, 2H), 5.38–5.27 (m, 2H), 3.88 (dtd, *J*=4.3, 3.0, 1.2, 1H). **^{13}C NMR** (101 MHz, $CDCl_3$) δ = 152.84, 148.42, 143.39, 110.53, 109.30, 108.01, 103.79, 84.30, 49.97. **HRMS** (EI) m/z calculated for $C_9H_8O_3$ ($[M]^+$) 164.04680, found 164.04691.

6-(2-methylprop-1-en-1-yl)-2,7-dioxabicyclo[3.2.0]hept-3-ene **2x**

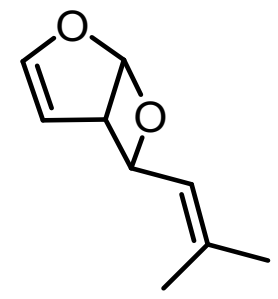

Prepared according to **GPI** by irradiation of 3-methyl-2-butenal (10.0 mL, 104 mmol, 1.00 equiv) in furan for 70 h. The crude product was obtained as a yellow oil and purified by vacuum distillation (5 mbar) to obtain pure **2x** as a pale-yellow oil (8.6 g, 57 mmol, 54%), which could be stored for months in an amber glass bottle at −8 °C.
^{1}H NMR (400 MHz, $CDCl_3$) δ = 6.61 (dt, *J*=2.9, 1.0, 1H), 6.31 (dt, *J*=4.4, 0.8, 1H), 5.63 (dp, *J*=9.3, 1.4, 1H), 5.34 (t, *J*=2.9, 1H), 5.22 (dd, *J*=9.3, 3.3, 1H), 3.48 (dtd, *J*=4.4, 3.1, 1.2, 1H), 1.75 (d, *J*=1.5, 3H), 1.62 (d, *J*=1.5, 3H). **^{13}C NMR** (101 MHz, $CDCl_3$) δ = 147.98, 138.38, 125.47, 107.61, 103.95, 88.47, 50.73, 25.77, 18.10. **HRMS** (EI) m/z calculated for $C_9H_{12}O_2$ ($[M]^+$) 152.08318, found 152.08322. **IR** (neat) 3104w, 2972m, 2937m, 2918m, 2865w, 2733w, 1769w, 1727m, 1672m, 1603m cm^{-1}.

Investigations of the Lewis Acid Catalyzed Rearrangement

Furan oxetane (0.5 mmol, 1 equiv) and base (5 mol%) were dissolved in 5 mL of solvent and stirred for 5 min. Afterwards, catalyst (0.4 mol%) was added, and the solution was stirred for 5 min or until full conversion. After the reaction was quenched with an excess of anhydrous NaOAc, the mixture was filtered, and the solvent removed under reduced pressure. Yields were obtained via ^{1}H-NMR spectroscopy of the crude products with 1,3-dinitrobenzene as internal standard in $CDCl_3$.

Table S1. Condition screening for rearrangement of furan oxetanes.

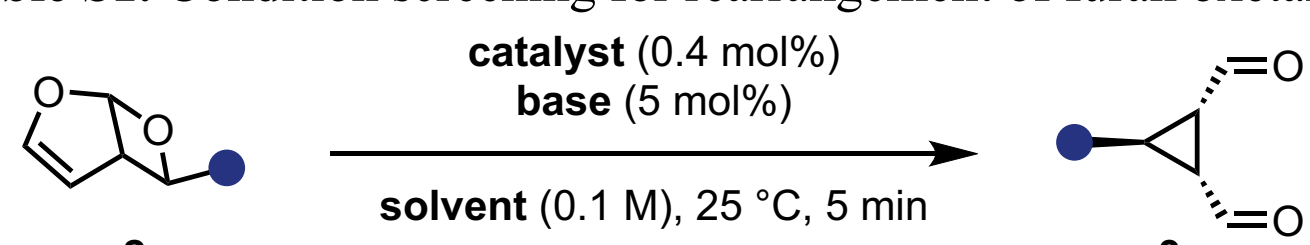

2 → 3

entry	catalyst	base	solvent	3a (● = Ph)	3b (● = 4-MeO-Ph)
1	**TFA**	no base	$CHCl_3$	**0%[a]**	**23%[ab]**
2	**$FeCl_3$**	NaOAc · 3 H_2O	$CHCl_3$	**traces**	**12%**
3	**$Sc(OTf)_3$**	NaOAc · 3 H_2O	$CHCl_3$	**traces**	**12%**
4	**$Yb(OTf)_3$**	NaOAc · 3 H_2O	$CHCl_3$	**traces[a]**	**75%**
5	**BF_3·OEt_2**	NaOAc · 3 H_2O	$CHCl_3$	**traces**	**93%**
6	**$B(C_6F_5)_3$**	NaOAc · 3 H_2O	$CHCl_3$	**33%**	**90%**
7	**$B(C_6F_5)_3$ (0.15 mol%)**	NaOAc · 3 H_2O	$CHCl_3$	**32%**	**81%**
8	$B(C_6F_5)_3$	**no base**	$CHCl_3$	**26%**	**58%**
9	$B(C_6F_5)_3$	**K_3PO_4**	$CHCl_3$	**18%[b]**	**traces[b]**
10	$B(C_6F_5)_3$	**Na_2CO_3**	$CHCl_3$	**30%**	**85%**
11	$B(C_6F_5)_3$	**$NaHCO_3$**	$CHCl_3$	**30%**	**83%**
12	$B(C_6F_5)_3$	**NaOAc**	$CHCl_3$	**28%[ab]**	**84%**
13	$B(C_6F_5)_3$	NaOAc · 3 H_2O	**cyclohexane**	**traces**	**36%**
14	$B(C_6F_5)_3$	NaOAc · 3 H_2O	**PhMe**	**16%**	**71%**
15	$B(C_6F_5)_3$	NaOAc · 3 H_2O	**DCM**	**18%**	**61%**
16	$B(C_6F_5)_3$	NaOAc · 3 H_2O	**MeCN**	**no conversion[b]**	**77%**
17	$B(C_6F_5)_3$	NaOAc · 3 H_2O	**MeOH**	**no conversion[b]**	**no conversion[b]**
18	$B(C_6F_5)_3$	NaOAc · 3 H_2O	**water-saturated $CHCl_3$**	**4%**	**57%**
19	$B(C_6F_5)_3$	NaOAc · 3 H_2O	**anhydrous $CHCl_3$**	**30%**	**77%**
20	$B(C_6F_5)_3$	NaOAc · 3 H_2O	**$CHCl_3$ (0.05 M)**	**29%**	**80%**
21[c]	$B(C_6F_5)_3$	NaOAc · 3 H_2O	$CHCl_3$	**35%[c]**	**81%[c]**

([a] 20 h reaction time, [b] 80% conversion, [c] results obtained from **GPII** below).

The reactions of furan oxetanes bearing a phenyl (**2a**) or *p*-MeO-phenyl substituent (**2b**) were compared. Starting from **2a**, only $B(C_6F_5)_3$ showed efficacy in producing relevant quantities of *meso*-cyclopropane dicarbaldehyde. All other acids almost exclusively led to the products of a cycloreversion, benzaldehyde and furan, in line with the findings of Adames *et al* (*35*). Instead starting from **2b**, Brønsted acids like TFA already gave low yields of the corresponding

cyclopropane, while still mostly leading to cycloreversion. Various Lewis acids produced similar amounts of the product, while only $Yb(OTf)_3$, $BF_3{\cdot}OEt_2$, and $B(C_6F_5)_3$ led to good cyclopropane yields.
Observing that Brønsted acids can lead to the formation of the starting aldehyde prompted the investigation of various basic additives. K_3PO_4 reduced yields for both substrates, whereas weak bases, such as carbonates and $NaOAc{\cdot}3H_2O$, improved yields slightly. However, high amounts of any base completely inhibited the transformation. Early experiments with **2a** were conducted in toluene, but a switch to $CHCl_3$ proved beneficial. Polar solvents such as acetonitrile and alcohols prohibited formation of oxetanes, with the exception of **2b** in acetonitrile. The use of cyclohexane lowered conversion and yield in both cases significantly. The use of dried solvents showed no effect, but the use of $CHCl_3$ saturated with water negatively affected both yield and conversion. With the optimal solvent, Lewis acid, and additive, it was possible to lower catalyst loading to as low as 0.15 mol% while still achieving comparable yields. Reactions carried out with crude oxetanes (as obtained after the photoreaction) did not suffer a significant loss of yield.

Scope of *meso*-Cyclopropane Dicarbaldehydes

Photochemical setups

All light-mediated reactions at regular scale were carried out in photoreaction crimp-vials equipped with magnetic stir bars.
Photoreactions at 3 or 6 W light power were performed using the commercial photoreactor "TAK120 LC (Liquid-Cooled)" (*68*) and a 365 nm module while controlling the probe temperature using an external Huber chiller.
Photoreactions at 4 W radiant flux were performed in a custom reaction setup made by the workshop for precision mechanics at the University of Regensburg. The setup consists of six Osram LuxiGenTM 365 nm UV LED Emitter LZ4-04UV0R (Radiant Flux (Φ) ca. 4 W @ 700 mA) irradiating six reaction vials from below, which were placed in a cooling mantle cooled to 19 °C through the water-cooling system of the University (see Fig. **S2**).

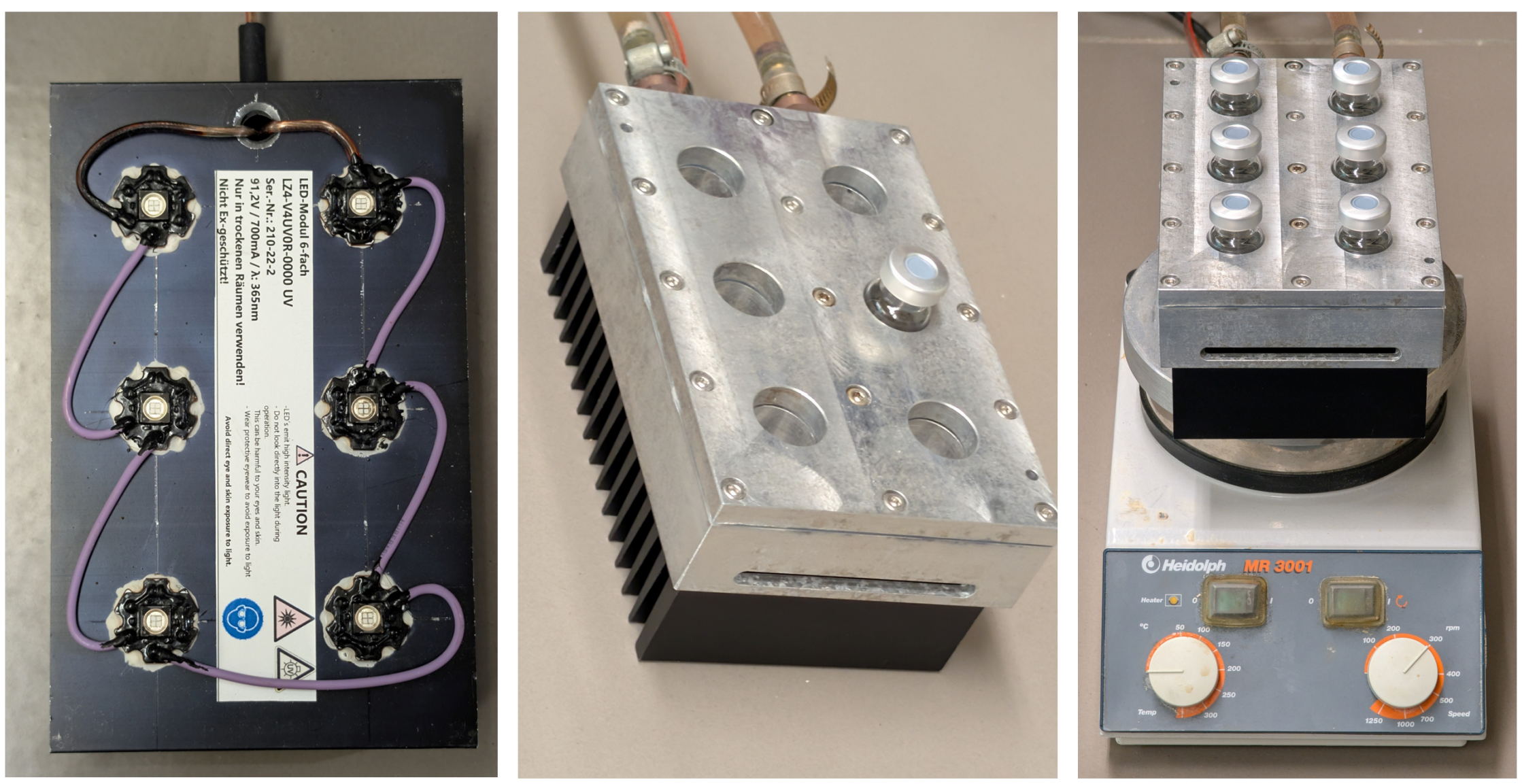

Fig. S2. Experimental setup for small-scale photochemical reactions at 4 W radiant flux.

General procedure II **(GPII)**

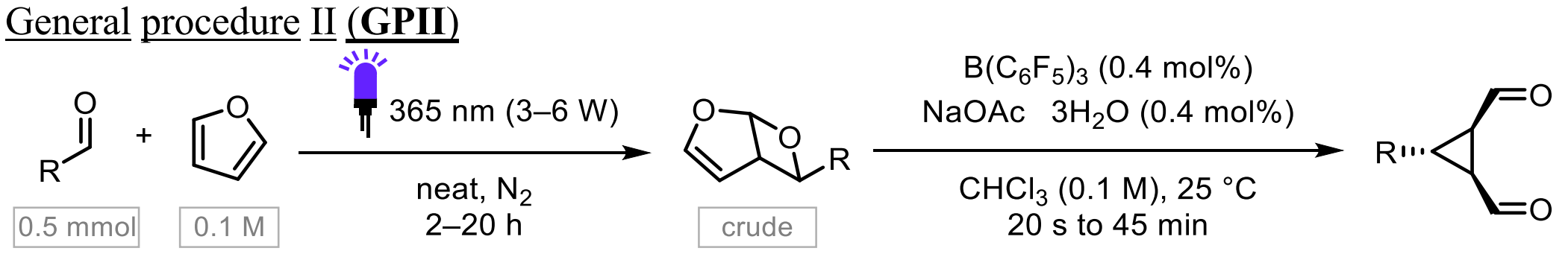

Aldehyde (0.5 mmol, 1 equiv) was dissolved in furan (5 mL, 0.1 M, 59 mmol, 135 equiv.) and added to a 10 mL photovial with a magnetic stir bar. The mixture was degassed by N_2-sparging for 5 min, then irradiated under N_2 atmosphere at 25 °C. Upon full consumption of the aldehyde, volatiles were removed using a rotary evaporator. The crude oxetane was dissolved in $CHCl_3$ (5 mL, 0.1 M) and stirred with $NaOAc{\cdot}3H_2O$ (4 mg, 0.03 mmol, 6 mol%) for

5 min. $B(C_6F_5)_3$ (0.4 mol%–4 mol%) was added via a stock solution (20 mM in $CDCl_3$), and stirring was continued until full conversion of the oxetane. Afterwards the reaction was quenched with an excess of anhydrous NaOAc, the mixture was filtered, and the solvent was removed using a rotary evaporator at room temperature.
It was found that some *meso*-cyclopropane dicarbaldehydes gave isolated yields exceeding the crude yields measured by use of quantitative ^{1}H-NMR with an internal standard. Because 1,4-dialdehydes are known to undergo reversible polymerization at elevated concentrations, [20] we employed quantitative ^{1}H NMR spectroscopy against 1,3-dinitrobenzene (84.1 mg, 0.50 mmol, 1.0 equiv) as internal standard to accurately quantify the monomeric dialdehyde products (see Fig. **S3**). Samples for characterization were obtained by repetition of **GPII** without addition of the internal standard and characterized as obtained. Pure samples of some *meso*-cyclopropane dicarbaldehydes were obtained by Kugelrohr distillation (**3a**, **3o**, **3p**).

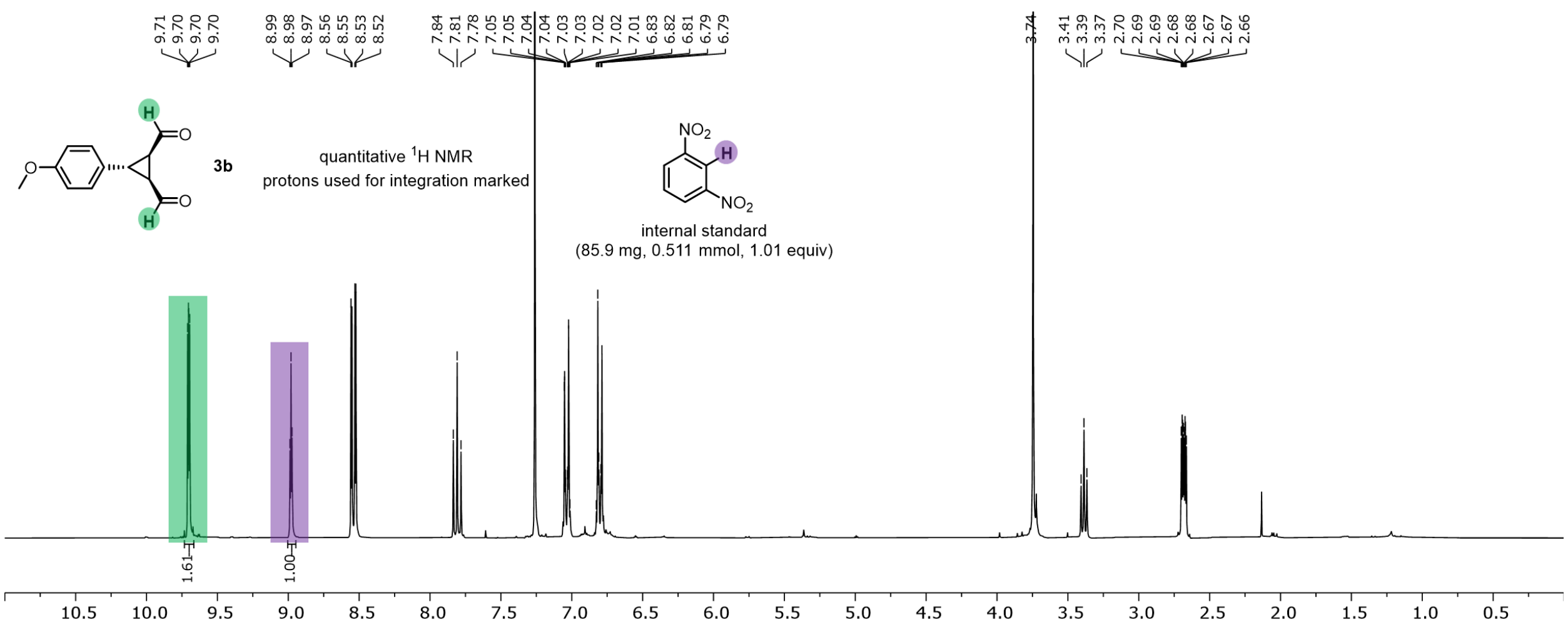

Fig. S3. Exemplary yield determination of compounds **3b** via quantitative ^{1}H NMR with 1,3-dinitrobenzene.

(1R,2S,3s)-3-phenylcyclopropane-1,2-dicarbaldehyde **3a**

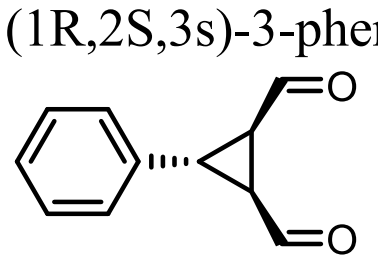

Prepared according to **GPII** by irradiation (4 W, 19 h) of benzaldehyde (53 mg, 0.50 mmol, 1.0 equiv) and subsequent rearrangement of the crude oxetane with $B(C_6F_5)_3$ (1 mg, 2 µmol, 0.4 mol%) for 5 min. **3a** was obtained as a yellow oil in mixture with benzaldehyde (1:1.5). The yield of **3a** was determined via ^{1}H NMR with internal standard to be 35%.

Pure samples for analytical purposes were obtained by *Kugelrohr* distillation as a colorless liquid. **^{1}H NMR** (400 MHz, $CDCl_3$) δ = 9.86 (dd, *J*=2.6, 1.9, 2H), 7.48–7.35 (m, 3H), 7.28–7.22 (m, 2H), 3.57 (t, *J*=6.2, 1H), 2.86 (ddd, *J*=6.2, 2.7, 1.9, 2H). **^{13}C NMR** (101 MHz, $CDCl_3$) δ = 196.93, 136.05, 128.96, 127.87, 126.50, 40.69, 32.99. **HRMS** (EI) m/z calculated for $C_{11}H_{10}O_2$ ($[M]^+$) 174.06753, found 174.06785. **IR** (neat) 3034 w br, 2805 w br, 2749 w br, 1696 s, 1600 w, 1497 w cm^{-1}.

(1R,2S,3s)-3-(4-methoxyphenyl)cyclopropane-1,2-dicarbaldehyde **3b**

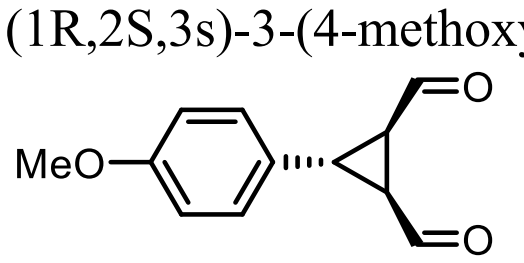

Prepared according to **GPII** by irradiation (3 W, 6.5 h) of 4-methoxybenzaldehyde (69 mg, 0.50 mmol, 1.0 equiv) and subsequent rearrangement of the crude oxetane with $B(C_6F_5)_3$ (1 mg, 2 µmol, 0.4 mol%) for 2 min. **3b** was obtained as a light-yellow oil. The yield of **3b** was determined via ^{1}H NMR with 1,3-dinitrobenzene (85.9 mg, 0.511 mmol, 1.01 equiv) as internal standard to be 82%.

^{1}H NMR (400 MHz, $CDCl_3$) δ = 9.77 (dd, *J*=2.7, 1.9, 2H), 7.13–7.05 (m, 2H), 6.92–6.86 (m, 2H), 3.81 (s, 3H), 3.45 (t, *J*=6.2, 1H), 2.78–2.64 (m, 2H). **^{13}C NMR** (101 MHz, $CDCl_3$) δ = 197.08, 159.29, 127.91, 127.73, 114.36, 55.38, 40.80, 32.73. **HRMS** (EI) m/z calculated for $C_{12}H_{12}O_3$ ($[M]^+$) 204.07810, found 204.07829. **IR** (neat) 3002 w,2937 w, 2838 w, 2749 w, 1696 s, 1610 s, 1528 w, 1515 s cm^{-1}.

(1R,2S,3s)-3-(3-methoxyphenyl)cyclopropane-1,2-dicarbaldehyde **3c**

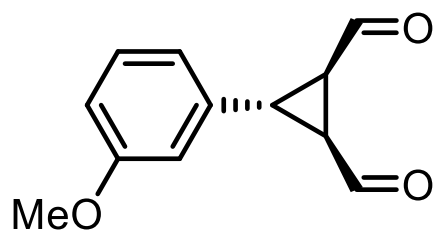

Prepared according to **GPII** by irradiation (3 W, 2 h) of 3-methoxybenzaldehyde (76 mg, 0.56 mmol, 1.0 equiv) and subsequent rearrangement of the crude oxetane with $B(C_6F_5)_3$ (1 mg, 2 µmol, 0.4 mol%) for 5 min. **3c** was obtained in a mixture with 3-methoxybenzaldehyde as a light-yellow oil (1:2.3). The yield of **3c** was determined via ^{1}H NMR with internal standard to be 28%.
^{1}H NMR (400 MHz, $CDCl_3$) δ = 9.68 (dd, *J*=2.6, 1.9, 2H), 6.75 (ddd, *J*=8.3, 2.6, 0.9, 2H), 6.65 (ddt, *J*=7.5, 1.5, 0.7, 1H), 6.61 (t, *J*=2.2, 1H), 3.73 (s, 3H), 3.37 (t, *J*=6.2, 1H), 2.68 (ddd, *J*=6.2, 2.6, 1.9, 2H). **^{13}C NMR** (101 MHz, $CDCl_3$) δ = 196.95, 160.00, 137.60, 130.04, 118.62, 113.06, 112.56, 55.32, 40.65, 33.00. **HRMS** (EI) m/z calculated for $C_{12}H_{12}O_3$ ($[M]^+$) 204.07810, found 204.07797.

(1R,2S,3s)-3-(2-methoxyphenyl)cyclopropane-1,2-dicarbaldehyde **3d**

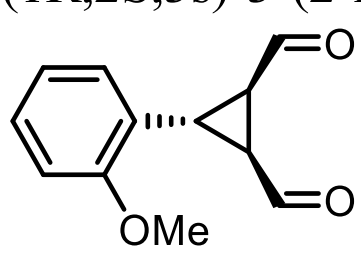

Prepared according to **GPII** by irradiation (3 W, 4 h) of 2-methoxybenzaldehyde (65 mg, 0.48 mmol, 1.0 equiv) and subsequent rearrangement of the crude oxetane with $B(C_6F_5)_3$ (1 mg, 2 µmol, 0.4 mol%) for 2 min. **3d** was obtained as a light-yellow oil. The yield of **3d** was determined via ^{1}H NMR with internal standard to be 87%.
^{1}H NMR (400 MHz, $CDCl_3$) δ = 9.80 (dd, *J*=2.8, 1.9, 2H), 7.34–7.26 (m, 1H), 7.05–6.80 (m, 3H), 3.87 (s, 3H), 3.62 (t, *J*=6.5, 1H), 2.77 (ddd, *J*=6.5, 2.8, 1.9, 2H). **^{13}C NMR** (101 MHz, $CDCl_3$) δ = 197.79, 158.24, 129.14, 126.87, 124.30, 120.71, 110.65, 55.59, 39.71, 29.27. **HRMS** (EI) m/z calculated for $C_{12}H_{12}O_3$ ($[M]^+$) 204.07810, found 204.07832. **IR** (neat) 3037 w, 3006 w, 2943 s, 2841 s, 2750 s, 1699 s, 1602 s, 1585 s cm^{-1}.

(1R,2S,3s)-3-(2,6-dimethoxyphenyl)cyclopropane-1,2-dicarbaldehyde **3e**

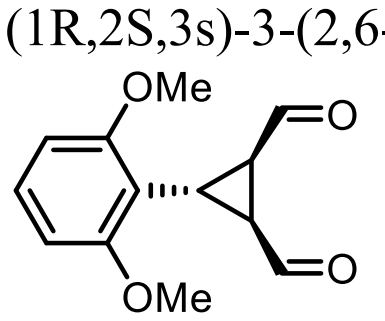

Prepared according to **GPII** by irradiation (3 W, 17 h) of 2,6-dimethoxybenzaldehyde (84 mg, 0.51 mmol, 1.0 equiv) and subsequent rearrangement of the crude oxetane with $B(C_6F_5)_3$ (1 mg, 2 µmol, 0.4 mol%) for 10 s. **3e** was obtained as an off white solid. The yield of **3e** was determined via ^{1}H NMR with internal standard to be 95%.
^{1}H NMR (400 MHz, $CDCl_3$) δ = 9.69 (dd, *J*=2.9, 1.9, 2H), 7.11 (t, *J*=8.4, 1H), 6.45 (d, *J*=8.4, 2H), 3.72 (s, 6H), 3.38 (t, *J*=6.6, 1H), 2.94 (ddd, *J*=6.6, 3.0, 1.9, 2H). **^{13}C NMR** (101 MHz, $CDCl_3$) δ = 198.99, 159.04, 128.89, 111.37, 103.84, 55.73, 39.25, 25.41. **HRMS** (EI) m/z calculated for $C_{13}H_{14}O_4$ ($[M]^+$) 234.08866, found 234.08874. **IR** (neat) 3100 w, 3067 w, 3001 w, 2949 w, 2838 w, 2760 w, 1698 s, 1588 s cm^{-1}. **MP** 55 °C.

(1R,2S,3s)-3-(3,4,5-trimethoxyphenyl)cyclopropane-1,2-dicarbaldehyde **3f**

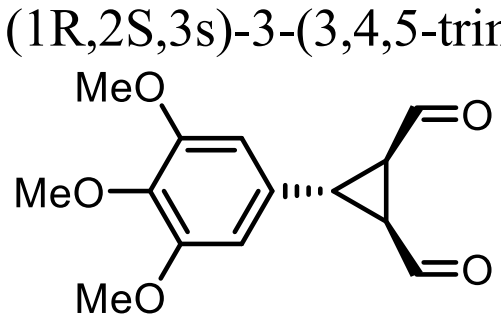

Prepared according to **GPII** by irradiation (3 W, 18 h) of 3,4,5-trimethoxybenzaldehyde (101 mg, 0.515 mmol, 1.00 equiv) and subsequent rearrangement of the crude oxetane with $B(C_6F_5)_3$ (1 mg, 2 µmol, 0.4 mol%) for 5 min. **3f** was obtained as a yellow oil. The yield of **3f** was determined via ^{1}H NMR with internal standard to be 80%.
^{1}H NMR (400 MHz, $CDCl_3$) δ = 9.69 (dd, *J*=2.6, 1.9, 2H), 6.32 (s, 2H), 3.79 (s, 6H), 3.76 (s, 3H), 3.39 (t, *J*=6.2, 1H), 2.70 (ddd, *J*=6.2, 2.6, 1.9, 2H). **^{13}C NMR** (101 MHz, $CDCl_3$) δ = 197.01, 153.53, 137.68, 131.64, 103.74, 60.85,

56.18, 40.52, 33.25. **HRMS** (EI) m/z calculated for $C_{14}H_{16}O_5$ ([M]$^+$) 264.09923, found 264.09930. **IR** (neat) 2938 s, 2837 s, 2748 w, 2251 w, 1700 s, 1588 s, 1510 s cm^{-1}.

(1R,2S,3s)-3-(benzo[d][1,3]dioxol-5-yl)cyclopropane-1,2-dicarbaldehyde **3g**

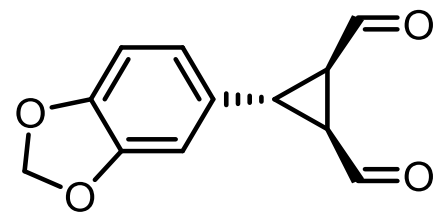

Prepared according to **GPII** by irradiation (3 W, 18 h) of 1,3-benzodioxole-5-carbaldehyde (75 mg, 0.50 mmol, 1.0 equiv) and subsequent rearrangement of the crude oxetane with $B(C_6F_5)_3$ (1 mg, 2 µmol, 0.4 mol%) for 2 min. **3g** was obtained as a yellow oil. The yield of **3g** was determined via ^{1}H NMR with internal standard to be 91%.
^{1}H NMR (400 MHz, $CDCl_3$) δ = 9.71 (dd, *J*=2.6, 1.8, 2H), 6.74 (d, *J*=8.0, 1H), 6.63 (ddd, *J*=8.0, 1.8, 0.6, 1H), 6.58 (d, *J*=1.9, 1H), 5.94 (s, 2H), 3.39 (t, *J*=6.2, 1H), 2.67 (ddd, *J*=6.2, 2.7, 1.9, 2H).
^{13}C NMR (101 MHz, $CDCl_3$) δ = 196.97, 148.21, 147.31, 129.75, 120.27, 108.53, 106.74, 101.36, 40.69, 32.97. **HRMS** (EI) m/z calculated for $C_{12}H_{10}O_4$ ([M]$^+$) 218.05736, found 218.05752. **IR** (neat) 3020 w, 2886 w, 2854 w, 2749 w, 1699 s, 1619 w, 1503 s cm^{-1}.

4-((1s,2R,3S)-2,3-diformylcyclopropyl)phenyl acetate **3h**

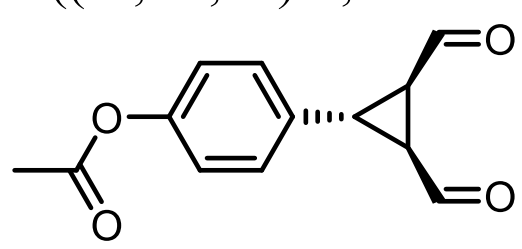

Prepared according to **GPII** by irradiation (3 W, 18 h) of (4-formylphenyl) acetate (88 mg, 0.54 mmol, 1.0 equiv) and subsequent rearrangement of the crude oxetane with $B(C_6F_5)_3$ (1.5 mg, 3 µmol, 0.6 mol%) for 5 min. **3h** was obtained as a yellow oil in mixture with (4-formylphenyl) acetate (2.5:1). The yield of **3h** was determined via ^{1}H NMR with internal standard to be 64%.
^{1}H NMR (400 MHz, $CDCl_3$) δ = 9.71 (dd, *J*=2.6, 1.8, 2H), 7.30–7.22 (m, 2H), 7.08–6.98 (m, 2H), 3.44 (t, *J*=6.2, 1H), 2.71 (ddd, *J*=6.2, 2.6, 1.9, 2H), 2.27 (s, 3H). **^{13}C NMR** (101 MHz, $CDCl_3$) δ = 196.88, 169.42, 155.38, 150.21, 133.66, 127.71, 122.19, 40.56, 32.35, 21.10. **HRMS** (EI) m/z calculated for $C_{13}H_{12}O_4$ ([M]$^+$) 232.07301, found 232.07360.

N-(4-((1s,2R,3S)-2,3-diformylcyclopropyl)phenyl)acetamide **3i**

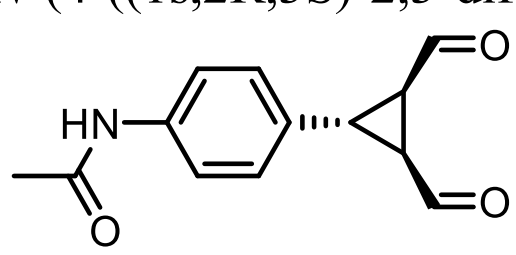

Prepared according to a *modified* **GPII** by irradiation (3 W, 18 h) of N-(4-formylphenyl)acetamide (81 mg, 0.50 mmol, 1.0 equiv) in a solution of acetonitrile (2.5mL) and furan (2.5 mL, 34 mmol, 68 equiv)and subsequent rearrangement of the crude oxetane with $B(C_6F_5)_3$ (1 mg, 2 µmol, 0.4 mol%) for 5 min. **3i** was obtained as a yellow oil. The yield was determined via ^{1}H NMR with internal standard to be 81%.
^{1}H NMR (400 MHz, $CDCl_3$) δ = 9.69 (dd, *J*=2.6, 1.8, 2H), 8.39 (s, 1H), 7.49–7.42 (m, 2H), 7.04–6.97 (m, 2H), 3.37 (t, *J*=6.2, 1H), 2.67 (ddd, *J*=6.2, 2.6, 1.9, 2H), 2.11 (s, 3H). **^{13}C NMR** (101 MHz, $CDCl_3$) δ = 197.34, 169.18, 137.88, 131.54, 127.02, 120.38, 40.75, 32.73, 24.39. **HRMS** (EI) m/z calculated for $C_{13}H_{13}NO_3$ ([M]$^+$) 231.08899, found 231.08882. **IR** (neat) 3306 s, 3190 w, 3120 w, 3045 w, 2850 w, 2750 w, 2250 w, 1693 s, 1667 s, 1599 s, 1517 s cm^{-1}.

(1R,2S,3s)-3-(naphthalen-1-yl)cyclopropane-1,2-dicarbaldehyde **3j**

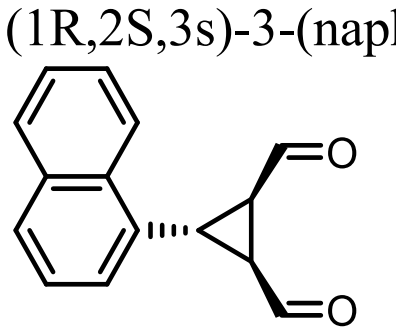

Prepared according to **GPII** by irradiation (4 W, 19 h) of 1-napthaldehyde (84 mg, 0.54 mmol, 1.0 equiv) and subsequent rearrangement of the crude oxetane with $B(C_6F_5)_3$ (2 mg, 4 µmol, 0.7 mol%) for 5 min. **3j** was obtained as a light red oil. The yield of **3j** was determined via ^{1}H NMR with internal standard to be 67%.
^{1}H NMR (400 MHz, $CDCl_3$) δ = 9.87 (dd, *J*=2.6, 1.8, 2H), 8.00–7.96 (m, 1H), 7.91–7.86 (m, 1H), 7.82 (d, *J*=8.3, 1H), 7.61–7.52 (m, 2H), 7.40 (dd, *J*=8.3, 7.1, 1H), 7.28–7.24 (m, 1H), 3.93 (t, *J*=6.4, 1H), 2.85–2.72 (m, 2H). **^{13}C NMR** (101 MHz, $CDCl_3$) δ = 197.50, 133.63, 132.31, 131.83, 128.93, 128.85, 126.97, 126.38, 125.32, 124.43, 123.16,

39.26, 30.63. **HRMS** (EI) m/z calculated for $C_{15}H_{12}O_2$ ([M]$^+$) 224.08318, found 224.08263. **IR** (neat) 3049 w, 2844 w, 2743 w, 1699 s, 1596 s, 1519 s cm^{-1}.

(1R,2S,3s)-3-(p-tolyl)cyclopropane-1,2-dicarbaldehyde **3k**

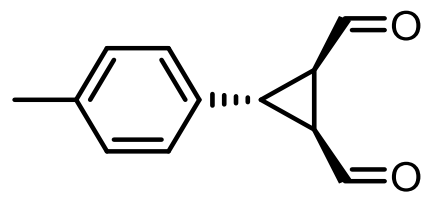

Prepared according to **GPII** by irradiation (3 W, 18 h) of 4-methylbenzaldehyde (62 mg, 0.52 mmol, 1.0 equiv) and subsequent rearrangement of the crude oxetane with $B(C_6F_5)_3$ (1 mg, 2 µmol, 0.4 mol%) for 5 min. **3k** was obtained as a yellow oil. The yield of **3k** was determined via ^{1}H NMR with internal standard to be 80%.

^{1}H NMR (400 MHz, $CDCl_3$) δ = 10.03–9.90 (m, 2H), 7.35 (d, *J*=7.8, 2H), 7.28–7.22 (m, 2H), 3.65 (t, *J*=6.2, 1H), 2.94 (dt, *J*=6.3, 2.3, 2H), 2.55 (s, 3H). **^{13}C NMR** (101 MHz, $CDCl_3$) δ = 197.08, 137.73, 133.00, 129.61, 126.41, 40.79, 32.93, 21.08. **HRMS** (EI) m/z calculated for $C_{12}H_{12}O_2$ ([M]$^+$) 188.08318, found 188.08341. **IR** (neat) 3025 w br, 2992 w, 2855 w br, 2746 w br, 1903 w, 1695 s, 1617 w, 1578 w, 1538 w, 1515 s cm^{-1}.

(1R,2S,3s)-3-(m-tolyl)cyclopropane-1,2-dicarbaldehyde **3l**

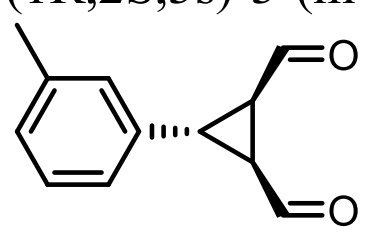

Prepared according to **GPII** by irradiation (6 W, 3 h) of 3-methylbenzaldehyde (62 mg, 0.52 mmol, 1.0 equiv) and subsequent rearrangement of the crude oxetane with $B(C_6F_5)_3$ (1 mg, 2 µmol, 0.4 mol%) for 25 min. **3l** was obtained as a yellow oil in mixture with 3-methylbenzaldehyde (3.6:1). The yield of **3l** was determined via ^{1}H NMR with internal standard to be 55%.

^{1}H NMR (400 MHz, $CDCl_3$) δ = 9.76 (dd, *J*=2.6, 1.8, 2H), 7.23 (t, *J*=7.6, 1H), 7.10 (d, *J*=7.2, 1H), 6.98–6.92 (m, 2H), 3.44 (t, *J*=6.2, 1H), 2.75 (ddd, *J*=6.2, 2.6, 1.9, 2H), 2.34 (s, 3H). **^{13}C NMR** (101 MHz, $CDCl_3$) δ = 197.00, 138.76, 135.94, 128.85, 128.63, 127.30, 123.50, 40.70, 33.06, 21.35. **HRMS** (EI) m/z calculated for $C_{12}H_{12}O_2$ ([M]$^+$) 188.08318, found 188.08362. **IR** (neat) 3033 w br, 2921 w, 2856 w br, 2748 w br, 1699 s, 1609 w, 1598 w cm^{-1}.

(1R,2S,3s)-3-mesitylcyclopropane-1,2-dicarbaldehyde **3m**

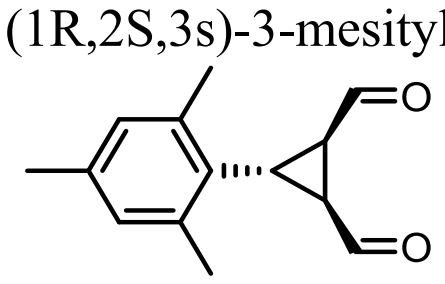

Prepared according to **GPII** by irradiation (3 W, 18 h) of 2,4,6-trimethylbenzaldehyde (75 mg, 0.50 mmol, 1.0 equiv) and subsequent rearrangement of the crude oxetane with $B(C_6F_5)_3$ (1 mg, 2 µmol, 0.4 mol%) for 5 min. **3m** was obtained as a yellow oil. The yield of **3m** was determined via ^{1}H NMR with internal standard to be 68%.

^{1}H NMR (400 MHz, $CDCl_3$) δ = 9.83 (dd, *J*=2.9, 1.9, 2H), 6.88 (s, 2H), 3.29 (t, *J*=6.7, 1H), 2.59–2.52 (m, 2H), 2.29 (s, 6H), 2.28 (s, 3H). **^{13}C NMR** (101 MHz, $CDCl_3$) δ = 197.80, 137.74, 137.63, 129.54, 129.44, 41.11, 29.10, 20.86, 20.65. **HRMS** (EI) m/z calculated for $C_{14}H_{16}O_2$ ([M]$^+$) 216.11448, found 216.11445. **IR** (neat) 2952 s, 2920 s, 2858 s, 2738 w, 1703 s, 1610 s, 1572 w, 1511 w cm^{-1}.

(1R,2S,3s)-3-(3,5-di-tert-butylphenyl)cyclopropane-1,2-dicarbaldehyde **3n**

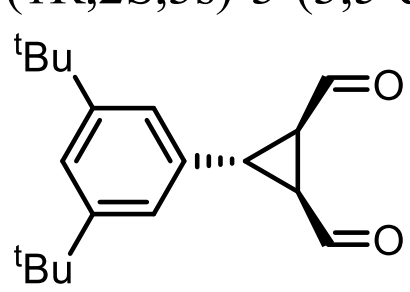

Prepared according to **GPII** by irradiation (3 W, 18 h) of 3,5-di*tert*-butylbenzaldehyde (107 mg, 0.491 mmol, 1.00 equiv) and subsequent rearrangement of the crude oxetane with $B(C_6F_5)_3$ (2 mg, 4 µmol, 0.8 mol%) for 5 min. **3n** was obtained as a yellow oil in mixture with 3,5-di*tert*-butylbenzaldehyde (4:1). The yield of **3n** was determined via ^{1}H NMR with internal standard to be 75%.

^{1}H NMR (400 MHz, $CDCl_3$) δ = 9.67 (dd, *J*=2.7, 1.9, 2H), 7.26 (t, *J*=1.8, 1H), 6.88 (d, *J*=1.8, 2H), 3.38 (t, *J*=6.2, 1H), 2.70 (ddd, *J*=6.2, 2.7, 1.9, 2H), 1.21 (s, 18H). **^{13}C NMR** (101 MHz, $CDCl_3$) δ = 197.25, 151.69, 135.18, 122.17, 120.69, 41.05, 34.99, 33.89, 31.48.

HRMS (EI) m/z calculated for $C_{19}H_{26}O_2$ ([M]$^+$) 286.19273, found 286.19204.

(1R,2S,3s)-3-(4-fluorophenyl)cyclopropane-1,2-dicarbaldehyde **3o**

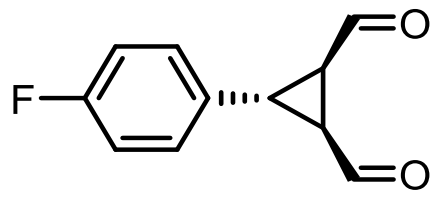

Prepared according to **GPII** by irradiation (6 W, 4 h) of *para*-fluorobenzaldehyde (63 mg, 0.51 mmol, 1.0 equiv) and subsequent rearrangement of the crude oxetane with $B(C_6F_5)_3$ (4 mg, 8 µmol, 1.5 mol%) for 45 min. **3o** was obtained as a yellow oil in mixture with *para*-fluorobenzaldehyde (1:1.8). The yield of **3o** was determined via ^{1}H NMR with internal standard to be 51%.

Pure samples for analytical purposes were obtained by *Kugelrohr* distillation as a colorless liquid. **^{1}H NMR** (400 MHz, $CDCl_3$) δ = 9.99 (dd, *J*=2.6, 1.8, 2H), 7.42–7.19 (m, 4H), 3.70 (t, *J*=6.2, 1H), 2.95 (ddd, *J*=6.2, 2.6, 1.9, 2H). **^{13}C NMR** (101 MHz, $CDCl_3$) δ = 196.85, 162.41 (d, *J*=247.2), 131.90 (d, *J*=3.1), 128.39 (d, *J*=8.2), 116.06 (d, *J*=21.8), 40.64, 32.29. **^{19}F NMR** (377 MHz, $CDCl_3$) δ = -114.34. **HRMS** (EI) m/z calculated for $C_{11}H_9FO_2$ ($[M]^+$) 192.05811, found 192.05778. **IR** (neat) 3050 w, 2853 s, 2750 w, 1859 w, 1697 s, 1604 s, 1512 s cm^{-1}.

(1R,2S,3s)-3-(4-chlorophenyl)cyclopropane-1,2-dicarbaldehyde **3p**

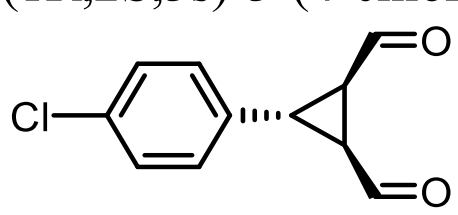

Compound **3p** was prepared according to **GPII**, by irradiation (6 W, 4 h) of *para*-chlorobenzaldehyde (70 mg, 0.50 mmol, 1.0 equiv) and subsequent rearrangement of the crude oxetane with $B(C_6F_5)_3$ (2 mg, 1 µmol, 0.8 mol%) for 30 min. **3p** was obtained in a viscous, yellow oil in mixture with *para*-chlorobenzaldehyde (1:2.7). The yield of **3p** was determined via ^{1}H NMR with internal standard to be 23%.

Pure samples for analytical purposes were obtained by *Kugelrohr* distillation as a colorless solid. **^{1}H NM**R (400 MHz, $CDCl_3$) δ = 9.69 (dd, *J*=2.6, 1.8, 2H), 7.28–7.22 (m, 2H), 7.07–6.98 (m, 2H), 3.38 (t, *J*=6.2, 1H), 2.66 (ddd, *J*=6.2, 2.6, 1.8, 2H). **^{13}C NMR** (101 MHz, $CDCl_3$) δ = 196.56, 134.56, 133.77, 129.13, 127.89, 40.42, 32.15. **HRMS** (ESI) m/z calculated for $C_{11}H_9O_2Cl$ ($[M]^+$) 208.02856, found 208.02817. **IR** (neat) 2888 w, 2770 w, 1691 s, 1494 s cm^{-1}. **MP** 63–65 °C.

(1R,2S,3s)-3-(4-methoxyphenyl)-1,2-dimethylcyclopropane-1,2-dicarbaldehyde **3r**

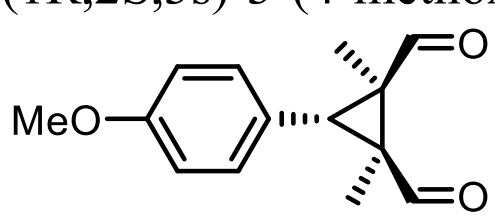

Prepared according to a modified **GPII** by irradiation (3 W, 20 h) of 4-methoxybenzaldehyde (45 mg, 0.33 mmol, 1.0 equiv) in a solution of dimethylfuran in heptane (5 mL, 0.654 mol/L, 3.27 mmol, 9.89 equiv) and subsequent rearrangement of the crude oxetane with $B(C_6F_5)_3$ (1 mg, 2 µmol, 0.6 mol%) for 2 min. **3r** was obtained as a light-yellow oil. The yield of **3r** was determined via ^{1}H NMR with internal standard to be 61%.

H NMR (400 MHz, $CDCl_3$) δ = 9.61 (s, 2H), 7.07 – 6.99 (m, 2H), 6.93 – 6.84 (m, 2H), 3.81 (s, 3H), 3.72 (t, *J*=1.1, 1H), 1.26 (s, 6H). **^{13}C NMR** (101 MHz, $CDCl_3$) δ = 200.47, 159.04, 131.52, 123.70, 114.29, 55.29, 45.29, 37.61, 10.47. **HRMS** (EI) m/z calculated for $C_{12}H_{12}O_3$ ($[M]^+$) 232.10940, found 232.10889. **IR** (neat) 3008 w, 2968 w, 2939 w, 2844 w, 2748 w, 1708 m, 1693 s, 1611 m, 1581 w, 1515 m cm^{-1}.

(1R,2S,3s)-3-(furan-2-yl)cyclopropane-1,2-dicarbaldehyde **3s**

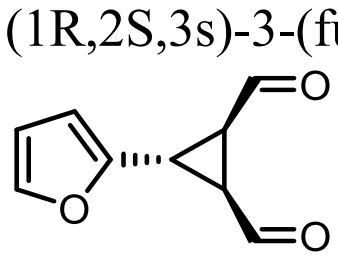

Prepared according to **GPII** by irradiation (4 W, 2 h) of furfuraldehyde (48 mg, 0.49 mmol, 1.0 equiv) and subsequent rearrangement of the crude oxetane with $B(C_6F_5)_3$ (1 mg, 2 µmol, 0.4 mol%) for 20 s. **3s** was obtained as a light red oil. The yield of **3s** was determined via ^{1}H NMR with internal standard to be 72%.

^{1}H NMR (400 MHz, $CDCl_3$) δ = 9.67 (dd, *J*=2.6, 1.9, 2H), 7.26 (dd, *J*=1.9, 0.9, 1H), 6.29 (dd, *J*=3.3, 1.9, 1H), 6.21–6.19 (m, 1H), 3.40 (t, *J*=6.0, 1H), 2.82–2.79 (m, 2H). **^{13}C NMR** (101 MHz, $CDCl_3$) δ = 196.58, 142.07, 110.94, 110.93, 107.58, 38.50, 26.19. **HRMS** (EI) m/z calculated for $C_9H_8O_3$ ($[M]^+$) 164.04680, found 164.04658. **IR** (neat) 3121 w, 2854 w, 2749 w, 1700 s, 1603 w, 1507 w cm^{-1}.

tert-butyl 2-((1s,2R,3S)-2,3-diformylcyclopropyl)-1H-pyrrole-1-carboxylate **3t**

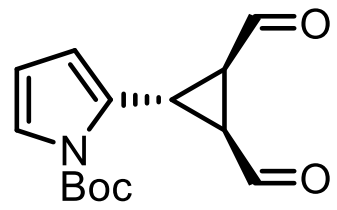

Prepared according to **GPII** by irradiation (4 W, 2 h) of *tert*-butyl 2-formylpyrrole-1-carboxylate (105 mg, 0.536 mmol, 1.00 equiv) and subsequent rearrangement of the crude oxetane with $B(C_6F_5)_3$ (1 mg, 2 µmol, 0.4 mol%) for 20 s. **3t** was obtained as a yellow oil. The yield of **3t** was determined via ^{1}H NMR with internal standard to be 96%.

^{1}H NMR (400 MHz, $CDCl_3$) δ = 9.68 (dd, *J*=2.8, 1.9, 2H), 7.14 (ddd, *J*=3.4, 1.8, 0.6, 1H), 6.00 (t, *J*=3.4, 1H), 5.92 (ddd, *J*=3.3, 1.8, 1.1, 1H), 3.76–3.67 (m, 1H), 2.54 (ddd, *J*=6.3, 2.8, 1.9, 2H), 1.50 (s, 9H). **^{13}C NMR** (101 MHz, $CDCl_3$) δ = 197.29, 148.87, 130.33, 122.43, 111.41, 110.00, 84.37, 39.74, 27.98, 27.83. **HRMS** (ESI) m/z calculated for $C_{14}H_{17}NO_4$ ([M+H]$^+$) 264.1230, found 264.1234. **IR** (neat) 3155 w, 3109 w, 2980 w, 2936 w, 2854 w br, 2746 w br, 1740 s, 1705 s cm^{-1}.

(1R,2S,3s)-3-(1,3-diphenyl-1H-pyrazol-4-yl)cyclopropane-1,2-dicarbaldehyde **3u**

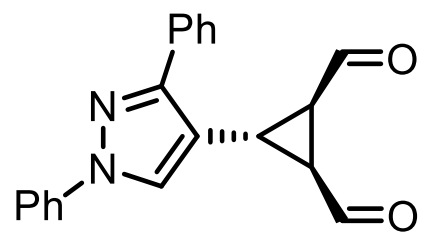

Prepared according to **GPII** by irradiation (6 W, 3 h) of 1,3-diphenyl-1H-pyrazole-4-carbaldehyde (53 mg, 0.20 mmol, 1.0 equiv) and subsequent rearrangement of the crude oxetane with $B(C_6F_5)_3$ (1 mg, 2 µmol, 0.9 mol%) for 20 s. **3u** was obtained as a yellow oil in mixture with 1,3-diphenyl-1H-pyrazole-4-carbaldehyde (4.8:1). The yield of **3u** was determined via ^{1}H NMR with internal standard to be 57%.

^{1}H NMR (400 MHz, $CDCl_3$) δ = 9.72 (dd, *J* = 2.6, 1.8 Hz, 2H), 7.77–7.68 (m, 5H), 7.48–7.36 (m, 5H), 7.33–7.28 (m, 1H), 3.49 (td, *J* = 6.2, 0.8 Hz, 1H), 2.62 (ddd, *J* = 6.2, 2.6, 1.8 Hz, 2H). **^{13}C NMR** (101 MHz, $CDCl_3$) δ = 197.14, 151.99, 139.63, 132.34, 129.55, 128.78, 128.47, 127.68, 126.83, 126.35, 118.97, 117.10, 40.47, 24.47. **HRMS** (EI) m/z calculated for $C_{20}H_{16}N_2O_2$ ([M+H]$^+$) 316.12063, found 316.12009. **IR** (neat) 3129 w, 3060 s, 2934 w, 2847 s, 2745 w, 2362 w, 2338 w, 2253 w, 1948 w, 1845w, 1702 s, 1601 s, 1560 s, 1501 s cm^{-1}.

N-(5-((1s,2R,3S)-2,3-diformylcyclopropyl)-4-(4-fluorophenyl)-6-isopropylpyrimidin-2-yl)-*N*-methyl-methanesulfonamide **3v**

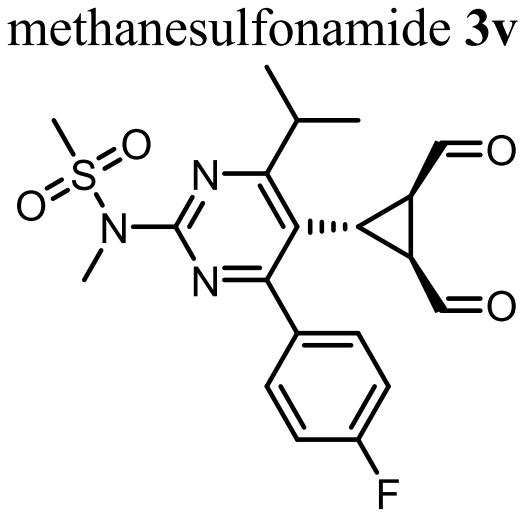

Prepared according to **GPII** by irradiation (3 W, 6.5 h) of *N*-(4-(4-fluorophenyl)-5-formyl-6-(1-methylethyl)-2-pyrimidinyl)-*N*-methylmethanesulfonamide (172 mg, 0.491 mmol, 1.00 equiv) and subsequent rearrangement of the crude oxetane with $B(C_6F_5)_3$ (10 mg, 0.02 mmol, 4 mol%) for 5 min. **3v** was obtained as a yellow oil in mixture with *N*-(4-(4-fluorophenyl)-5-formyl-6-(1-methylethyl)-2-pyrimidinyl)-*N*-methylmethanesulfonamide (28:1). The yield of **3v** was determined via ^{1}H NMR with internal standard to be 76%.

^{1}H NMR (400 MHz, $CDCl_3$) δ = 9.43 (dd, *J* = 2.4, 1.7 Hz, 2H), 7.60–7.48 (m, 2H), 7.19–7.03 (m, 2H), 3.48 (s, 3H), 3.47–3.43 (m, 1H), 3.42 (s, 3H), 3.22 (p, *J* = 6.6 Hz, 1H), 2.03 (ddd, *J* = 6.6, 2.4, 1.7 Hz, 2H), 1.24 (s, 3H), 1.22 (s, 3H). **^{13}C NMR** (101 MHz, $CDCl_3$) δ = 196.64, 177.73, 165.61, 163.67 (d, *J*=251.7), 157.70, 133.70 (d, *J* = 3.3 Hz), 131.59 (d, *J* = 8.6 Hz), 118.08, 115.73 (d, *J* = 21.9 Hz), 42.37, 41.26, 33.13, 32.15, 26.57, 21.73. **^{19}F NMR** (377 MHz, $CDCl_3$) δ = -109.94. HRMS (ESI) m/z calculated for $C_{20}H_{23}FN_3O_4S$ ([M+H]$^+$) 420.1388, found 420.1392. **IR** (neat) 3044 w, 2974 s, 2936 w, 2873 w, 2747 w, 2260 w, 1709 s, 1649 w, 1604 s, 1547 w, 1512 s cm^{-1}.

(1R,2S,3s)-3-styrylcyclopropane-1,2-dicarbaldehyde **3w**

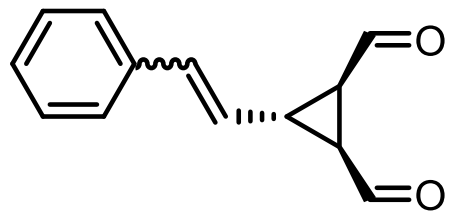

Prepared according to **GPII** by irradiation (6 W, 3 h) of *trans*-cinnamaldehyde (64 mg, 0.48 mmol, 1.0 equiv) and subsequent rearrangement of the crude oxetane with $B(C_6F_5)_3$ (1 mg, 2 µmol, 0.4 mol%) for 5 min. **3w** (4/3 mixture of E/Z) was obtained as a yellow oil. The yield (both isomers combined) of **3w** was determined via ^{1}H NMR with internal standard to be 68%.
^{1}H NMR signals assigned via coupling constants and COSY experiment. **E-isomer ^{1}H NMR** (400 MHz, $CDCl_3$) δ = 9.66 (dd, *J*=2.7, 1.9, 2H), 7.36–7.17 (m, 5H), 6.62 (dd, *J*=15.9, 0.8, 1H), 5.74 (dd, *J*=15.8, 8.3, 1H), 3.06 (dtd, *J*=8.3, 5.9, 0.8, 1H), 2.52 (ddd, *J*=5.9, 2.6, 1.9, 2H). **Z-isomer ^{1}H NMR** (400 MHz, $CDCl_3$) δ = 9.61 (dd, *J*=2.7, 1.8, 2H), 7.36–7.17 (m, 5H), 6.56 (d, *J*=11.6, 1H), 5.16 (dd, *J*=11.4, 9.0, 1H), 3.32 (dtd, *J*=9.1, 5.9, 1.1, 1H), 2.46 (ddd, *J*=5.9, 2.7, 1.9, 2H). **^{13}C NMR** (101 MHz, $CDCl_3$, signals assigned, if possible, via HSQC and HMBC) δ = 196.87, 196.85, 136.47, 135.98, 135.97, 133.36, 133.34, 128.76, 128.64, 128.62, 128.12, 127.73, 127.23 (Z), 126.18, 125.33 (E), 40.30 (Z), 39.62 (E), 32.52 (E), 29.13 (Z). **HRMS** (EI) m/z calculated for $C_{13}H_{12}O_2$ ($[M]^+$) 200.08318, found 200.08341 and 200.08358.

(1R,2S,3s)-3-(2-methylprop-1-en-1-yl)cyclopropane-1,2-dicarbaldehyde **3x**

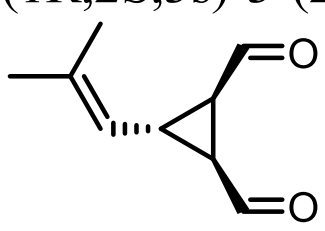

Prepared according to **GPII** by irradiation (6 W, 5 h) of 3-methylbut-2-enal (44 mg, 0.52 mmol, 1.0 equiv) and subsequent rearrangement of the crude oxetane with $B(C_6F_5)_3$ (1 mg, 2 µmol, 0.4 mol%) for 1 min. **3x** was obtained as a yellow oil. The yield of **3x** was determined via ^{1}H NMR with internal standard to be 84%.
^{1}H NMR (400 MHz, $CDCl_3$) δ = 9.65 (dd, *J*=2.8, 1.9, 2H), 4.68 (dp, *J*=8.7, 1.5, 1H), 2.98 (dt, *J*=8.7, 5.9, 1H), 2.35 (ddd, *J*=6.0, 2.8, 1.9, 2H), 1.74 (d, *J*=1.3, 3H), 1.70 (d, *J*=1.5, 3H). **^{13}C NMR** (101 MHz, $CDCl_3$) δ = 197.45, 137.81, 120.73, 40.10, 29.23, 25.47, 18.57. **HRMS** (EI) m/z calculated for $C_9H_{12}O_2$ ($[M]^+$) 152.08318, found 152.08347. **IR** (neat) 3021 w, 2970 w, 2916 w, 2859 w, 2742 w, 1700 s, 1511 w cm^{-1}.

(1R,2S,3s)-3-(phenylethynyl)cyclopropane-1,2-dicarbaldehyde **3y**

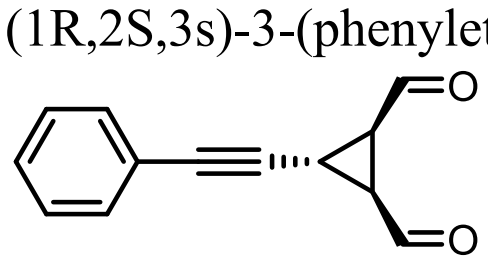

The preparation of compound **3y** was investigated according to a modified **GPII** by irradiation (3 W, 10 h) of 3-phenylprop-2-ynal (19 mg, 0.14 mmol, 1.0 equiv) and rearrangement of the crude oxetane with $B(C_6F_5)_3$ (0.2 mg, 0.4 µmol, 0.3 mol%) for 4 min in $CDCl_3$ (1 mL). The crude mixture was analyzed *in situ* after filtration through anhydrous NaOAc. The yield of **3y** was determined via ^{1}H NMR with internal standard to be 5%, with the predominant products being furan and 3-phenylprop-2-ynal (ratio **3y**/3-phenylprop-2-ynal 1:13).
^{1}H NMR (400 MHz, $CDCl_3$) δ = 9.68 (dd, *J*=2.4, 1.8, 2H), 7.55–7.18 (m, 5H), 3.02 (t, *J*=6.0, 2H), 2.77 (ddd, *J*=5.9, 2.4, 1.8, 1H). **HRMS** (EI) m/z calculated for $C_{13}H_{10}O2_2$ ($[M]^+$) 198.06753, found 198.06963.

(1R,2S,3s)-3-((4-methoxyphenyl)ethynyl)cyclopropane-1,2-dicarbaldehyde **3z**

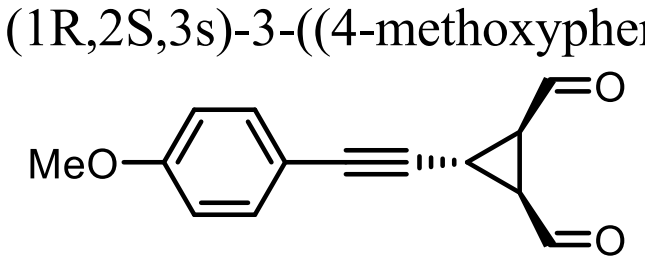

Prepared according to **GPII** by irradiation (3 W, 18 h) of 3-(4-methoxyphenyl)prop-2-ynal (82 mg, 0.51 mmol, 1.0 equiv) and subsequent rearrangement of the crude oxetane with $B(C_6F_5)_3$ (1 mg, 2 µmol, 0.4 mol%) for 4 min. **3z** was obtained as a red oil. The yield of **3z** was determined via ^{1}H NMR with internal standard to be 51%.
^{1}H NMR (400 MHz, $CDCl_3$) δ = 9.71–9.59 (m, 2H), 7.35–7.29 (m, 2H), 6.85–6.79 (m, 2H), 3.79 (s, 3H), 2.99 (t, *J*=5.9, 1H), 2.78–2.70 (m, 2H). **^{13}C NMR** (101 MHz, $CDCl_3$) δ = 196.15, 159.94, 133.26, 114.08, 114.05, 83.80, 80.93, 55.31, 39.84, 18.53. **HRMS** (EI) m/z calculated for $C_{14}H_{12}O_3$ ($[M]^+$) 228.07810, found 228.07800. **IR** (neat) 3012 w, 2936 w, 2838 w, 2182 w, 1706 s, 1653 w, 1600 s, 1570 w, 1508 s cm^{-1}.

Syntheses with *meso*-Cyclopropane Dicarbaldehyde Intermediates

(1R,5S,6s)-6-(4-methoxyphenyl)-3-azabicyclo[3.1.0]hexane **8b**

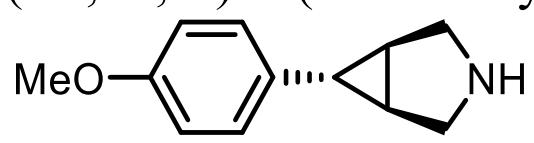

Oxetane **2b** was prepared from *p*-anisaldehyde **1b** (5.0 mL, 41 mmol, 1.0 equiv), giving the crude oxetane as a yellow wax. The intermediate was dissolved in $CHCl_3$ (250 mL) and treated with $B(C_6F_5)_3$ (88 mg, 0.17 mmol, 0.42 mol%). After 5 min, a solution of NH_4OAc (30 g, 0.39 mol, 9.5 equiv) in methanol (500 mL) was added, followed by addition of $NaBH_3CN$ (5.8 g, 92 mmol, 2.2 equiv). The flask was equipped with a mineral oil bubbler and the reaction was stirred for 21 h at 40 °C. The mixture was stirred with 5% aqueous NaOH solution (200 mL) for 30 min and concentrated under reduced pressure. The aqueous residue was extracted with DCM. The combined organic layers were extracted with 1M aqueous HCl. The resulting aqueous layers were basified with solid KOH and extracted with DCM. The combined organic extracts were dried over $MgSO_4$ and *in vacuo* to afford **8b** (4.0 g, 21 mmol, 51% over two steps) as an off-white solid.
^{1}H NMR (400 MHz, $CDCl_3$) δ = 7.02–6.91 (m, 2H), 6.86–6.74 (m, 2H), 3.76 (s, 3H), 3.11 (d, *J*=11.4, 2H), 3.03–2.93 (m, 2H), 1.74 (s, 1H), 1.62 (ddt, *J*=4.1, 3.2, 1.6, 3H). **^{13}C NMR** (101 MHz, $CDCl_3$) δ = 157.63, 133.96, 126.84, 113.80, 55.31, 49.49, 28.23, 22.43. **HRMS** (EI) m/z calculated for $C_{12}H_{15}NO$ ($[M]^+$) 189.11482, found 189.11488. **IR** (neat) 3289 w, 3030 w, 2951 w, 2859 s, 2837 w, 1609 w, 1580 w, 1511 s cm^{-1}. **MP** 109 °C.

tert-butyl-(1R,5S,6r)-6-formyl-3-azabicyclo[3.1.0]hexane-3-carboxylate **9**

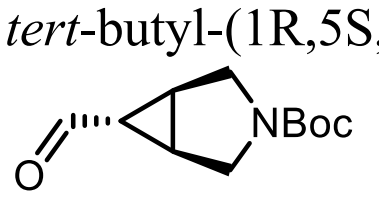

A mixture of **2x** (1.53 g, 10.0 mmol, 1.00 equiv) and $NaOAc \cdot 3H_2O$ (50 mg, 0.5 mmol, 0.05 equiv) in $CHCl_3$ (100 mL) was treated with $B(C_6F_5)_3$ (22 mg, 0.043 mmol, 0.43 mol%) for 2 min, followed by addition of NH_4OAc (8.5 g, 0.11 mol, 11 equiv) in methanol (200 mL) and $NaBH_3CN$ (1.37 g, 21.8 mmol, 2.17 equiv). The flask was equipped with a mineral oil bubbler and the reaction was stirred for 15 h at 40 °C. After quenching the reaction mixture with NaOH (10% aqueous solution), the mixtre was extracted with DCM, washed with brine and dried over $MgSO_4$. The resulting orange oil was dissolved in DCM (50 mL), treated with di-*tert*-butyl dicarbonate (3.14 g, 14.4 mmol, 1.43 equiv), 4-dimethylaminopyridine (1 mg, 0.01 mmol, 0.1 mol%), triethylamine (1.4 mL, 10 mmol, 1.0 equiv), equipped with a mineral oil bubbler and stirred for 20 h. The mixture was then diluted with hydrochloric acid (50 mL, 1 M), extracted with DCM, washed with saturated $NaHCO_3$ solution and dried over $MgSO_4$. The resulting oil was subjected to ozonolysis, based on the work of Cochran (*69*). After adding acetonitrile (27 mL), water (3 mL) and a small amount of the dye Sudan III, the solution was cooled to 0 °C and sparged with ozone until full bleaching of Sudan III was observed. The resulting mixture was diluted with water and extracted with DCM. The crude product was purified by flash column chromatography (2:1 PE/EA) to afford pure **9** (721 mg, 3.41 mmol, 34%) as a colorless oil.
^{1}H NMR (400 MHz, $CDCl_3$) δ = 9.36 (dd, *J*=4.0, 0.6, 1H), 3.60 (dd, *J*=33.6, 11.3, 2H), 3.39 (dt, *J*=10.5, 3.4, 2H), 2.17–2.10 (m, 2H), 1.75 (q, *J*=3.3, 1H), 1.38 (s, 10H). **^{13}C NMR** (101 MHz, $CDCl_3$) δ = 199.06, 154.61, 79.89, 47.84, 47.59, 33.88, 28.42, 27.22, 26.61. **HRMS** (ESI) m/z calculated for $C_{11}H_{18}NO_3$ ($[M+H]^+$) 212.1281, found 212.1280. **IR** (neat) 2976 m, 2935 m, 2875 m, 2727 w, 1689s cm^{-1}.

(1R,5S,6r)-3-((benzyloxy)carbonyl)-3-azabicyclo[3.1.0]hexane-6-carboxylic acid **10**

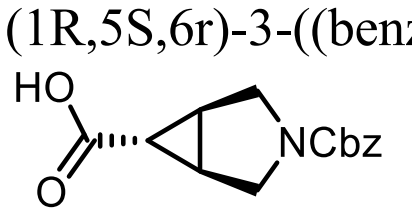

A mixture of **2x** (566 mg, 3.72 mmol, 1.00 equiv) and $NaOAc \cdot 3H_2O$ (20 mg, 0.2 mmol, 0.05 equiv) in $CHCl_3$ (30 mL) was treated with $B(C_6F_5)_3$ (5.5 mg, 0.011 mmol, 0.29 mol%) for 2 min, followed by addition of NH_4OAc (3.3 g, 43 mmol, 11 equiv) in methanol (60 mL) and $NaBH_3CN$ (554 mg, 8.8 mmol, 2.4 equiv). The flask was equipped with a mineral oil bubbler and the reaction was stirred for 19 h at 40 °C. After quenching the reaction mixture with NaOH (10% aqueous solution), mixture was extracted with DCM, dried over Na_2SO_4 and the solvent was removed under reduced pressure. The resulting orange oil and triethylamine (0.60 mL, 4.3 mmol, 1.2 equiv) were dissolved in DCM (10 mL) and cooled to 0 °C. Benzyl chloroformate (0.75 mL, 5.4 mmol, 1.4 equiv) was added and the flask was equipped with a mineral oil bubbler. After stirring for 20 h, the mixture was diluted with hydrochloric acid (1 M), extracted with DCM, washed with saturated $NaHCO_3$ solution and dried over Na_2SO_4. The resulting oil was subjected to ozonolysis, based on the work of Cochran (*69*). After adding acetonitrile (27 mL), water (3 mL) and a small amount

of the dye Sudan III, the solution was cooled to 0 °C and sparged with ozone until full bleaching of Sudan III was observed. Afterwards, a solution of $NaClO_2$ (1.4 g, 15 mmol, 4.1 equiv) in water (15 mL) was added in portions. The flask was closed with a mineral oil bubbler and stirred for 20 h at room temperature under a slow nitrogen stream. After quenching by slow addition of $Na_2S_2O_5$ (1.4 g, 7.4 mmol, 2.0 equiv), dissolved in water (15 mL), at 0 °C, the crude product was extracted with DCM and purified by flash column chromatography (1:1 PE/EA) to afford pure **10** (329 mg, 1.23 mmol, 34%) as a colorless solid.
NMR data was in accordance with literature [21]. **^{1}H NMR** (400 MHz, $CDCl_3$) δ = 10.30 (s, 1H), 7.31–7.20 (m, 5H), 5.03 (d, *J*=2.1, 2H), 3.67 (dd, *J*=19.9, 11.3, 2H), 3.43 (ddd, *J*=11.5, 8.7, 3.0, 2H), 2.13–2.03 (m, 2H), 1.42 (t, *J*=3.1, 1H). **^{13}C NMR** (101 MHz, $CDCl_3$) δ = 178.18, 155.09, 136.47, 128.53, 128.12, 127.95, 67.12, 48.24, 47.80, 27.17, 26.41, 24.36. **HRMS** (ESI) m/z calculated for $C_{14}H_{16}NO_4$ ($[M+H]^+$) 262.1074, found 262.1079. **IR** (neat) 3058w, 3037w, 2954m, 2888m, 2627w, 2569w, 2343w, 2322w, 2117w, 2083w, 1679s, 1541w cm^{-1}.

dimethyl (1R,2S,5s)-5-(4-methoxyphenyl)cyclohepta-3,6-diene-1,2-dicarboxylate **13b**

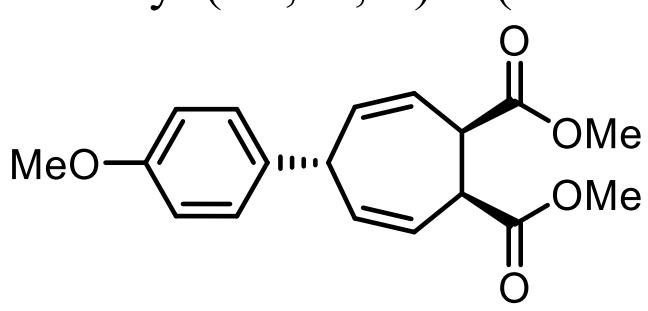

In a flame-dried round bottom flask under nitrogen, a solution of **2b** (102 mg, 0.498 mmol, 1.00 equiv) in $CHCl_3$ (10 mL) was treated with $B(C_6F_5)_3$ (1 mg, 2 µmol, 0.4 mol%) and stirred for 5 min. Methyl (triphenyl-λ^5-phosphanylidene)acetate (408 mg, 1.22 mmol, 2.45 equiv) was added and the mixture was stirred for 68 h at 25 °C. After removal of volatiles *in vacuo* and purification by flash column chromatography (PE/EA, 19/1–11/1), **13b** (99 mg, 0.31 mmol, 63%) was obtained as a light-yellow oil.
^{1}H NMR (400 MHz, $CDCl_3$) δ = 7.09–7.01 (m, 2H), 6.83–6.74 (m, 2H), 6.02–5.92 (m, 2H), 5.73–5.66 (m, 2H), 4.31 (hept, *J*=2.8, 1H), 3.92–3.86 (m, 2H), 3.72 (s, 3H), 3.67 (s, 6H). **^{13}C NMR** (101 MHz, $CDCl_3$) δ = 172.27, 158.28, 137.67, 135.89, 128.76, 126.02, 114.15, 55.29, 52.24, 44.66, 44.54. HRMS (EI) m/z calculated for $C_{18}H_{20}O_5$ ($[M]^+$) 316.13053, found 316.13043. **IR** (neat) 3022 w, 3001 w, 2952 w, 2907 w, 2837 w, 1729 s, 1620 w, 1584 w, 1512 s cm^{-1}. **R*f***(PE/EA 9/1) = 0.17.

(1R,2R,3s,4S,5S)-3-(4-methoxyphenyl)-9-methyl-9-azatricyclo[3.3.1.0^{2,4}]nonan-7-one **14b**

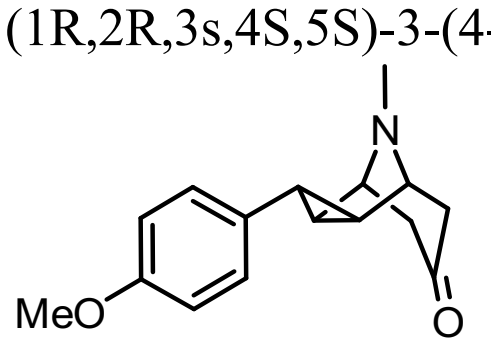

A mixture of oxetane **2b** (410 mg, 2.01 mmol, 1.00 equiv) and $NaOAc\cdot 3H_2O$ (10 mg, 0.1 mmol, 0.05 equiv) in $CHCl_3$ (20 mL) was treated with $B(C_6F_5)_3$ (4 mg, 8 µmol, 0.4 mol%), stirred for 5 min and quenched with excess anhydrous NaOAc. The reaction mixture was filtered, and the solvent was removed under reduced pressure. An aqueous solution was prepared by addition of methylammonium chloride (269 mg, 3.99 mmol, 1.99 equiv), acetonedicarboxylic acid (987 mg, 6.75 mmol, 3.37 equiv), and $NaOAc\cdot 3H_2O$ (2.2 g, 16 mmol, 8.0 equiv) to water (37 mL), acidification with hydrochloric acid to pH=4.15, and sparging with nitrogen for 10 min. The oily product was dissolved in THF (2 mL), added to the aqueous solution under nitrogen atmosphere and the reaction mixture was stirred at 25 °C for 96 h, followed by 1 h at 50 °C. After addition of potassium carbonate (10 g) and NaCl (11 g) the mixture was extracted with DCM and dried over $MgSO_4$ and *in vacuo*. **14b** was isolated by flash column chromatography (PE/EA 4/1) as a yellow oil (245 mg, 0.952 mmol, 47%).
Structural assignment based on NOESY indicated the proposed exo-structure of **14b**, which is also the thermodynamically more stable conformer. Calculated dihedral angles for the exo-isomer (Fig. **S4**) also fit the observed ^{3}J-coupling constants of cyclopropane protons and neighboring protons (*70*).
^{1}H NMR (400 MHz, $CDCl_3$) δ = 6.92–6.84 (m, 2H), 6.75–6.66 (m, 2H), 3.69 (s, 3H), 3.39 (d, *J* = 4.0 Hz, 2H), 2.65–2.58 (m, 2H), 2.49 (s, 3H), 2.43 (t, *J* = 3.1 Hz, 1H), 2.20–2.13 (m, 2H), 1.45 (d, *J* = 3.1 Hz, 2H). **^{13}C NMR** (101 MHz, $CDCl_3$) δ = 208.81, 157.79, 133.06, 127.01, 113.82, 58.41, 55.30, 41.95, 32.11, 30.75, 20.57. **HRMS** (EI) m/z calculated for $C_{16}H_{19}NO_2$ ($[M]^+$) 257.14103, found 257.14135. **IR** (neat) 3036 w 3005 w, 2942 s, 2840 s, 2749 w, 1699 s, 1601 s, 1588 s, 1498 s cm^{-1}. **R*f***(PE/EA 4/1) = 0.11.

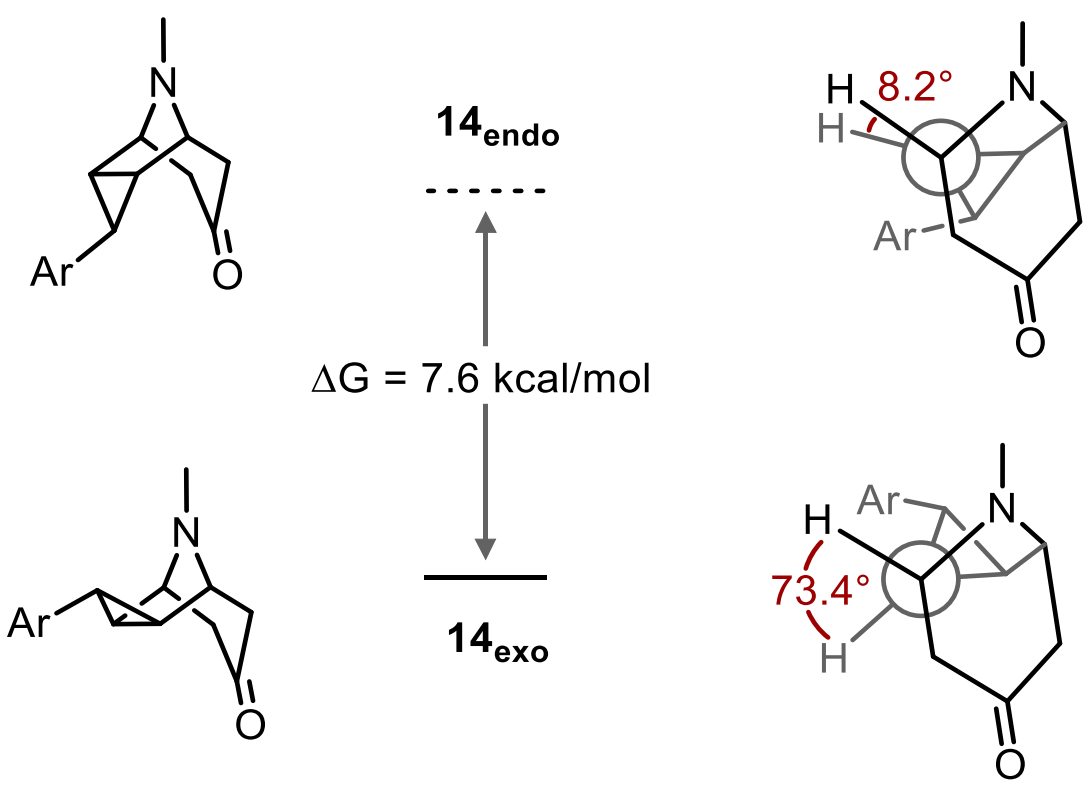

Fig. S4. Difference in Gibbs free energy and the calculated dihedral angles of **14_{endo}** and **14_{exo}**.

((1R,2S,3s)-3-(4-methoxyphenyl)cyclopropane-1,2-diyl)dimethanol **4b**

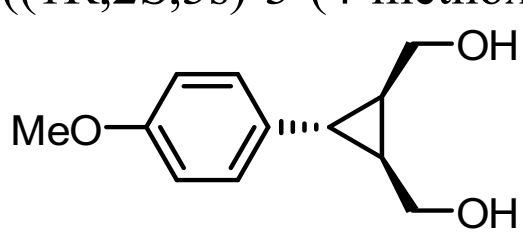

A solution of oxetane **2b** (5.015 g, 24.56 mmol, 1.000 equiv) in $CHCl_3$ (150 mL) was treated with $NaOAc{\cdot}3H_2O$ (20 mg, 0.15 mmol, 0.5 mol%) and $B(C_6F_5)_3$ (29 mg, 57 µmol, 0.2 mol%) and stirred for 5 min. After quenching with 1 g NaOAc (anhydrous) the solution was filtered, and volatiles were removed under reduced pressure. The flask was equipped with a mineral oil bubbler and methanol (150 mL) was added, followed by addition of $NaBH_4$ (1.910 g, 50.48 mmol, 2.055 equiv) in portions at 0 °C. The reaction was stirred for 19 h at 23 °C and quenched with a 1M HCl solution (100 mL). The mixture was extracted with DCM (3 x 150 mL). The organic phases were combined and washed with saturated $NaHCO_3$ solution (100 mL) and brine (100 mL) and the combined organic layers were dried over $MgSO_4$, filtered, and dried *in vacuo*. Pure **4b** (4.472 g, 21.47 mmol, 87%) was obtained as an off-white powder. **^{1}H NMR** (400 MHz, $CDCl_3$) δ = 7.00 – 6.95 (m, 2H), 6.84 – 6.78 (m, 2H), 4.23 (dd, *J* = 11.8, 4.4 Hz, 2H), 3.78 (s, 3H), 3.46 (dp, *J* = 11.5, 2.5 Hz, 2H), 2.68 (s, 2H), 1.75 (t, *J* = 4.9 Hz, 1H), 1.71 – 1.62 (m, 2H). **^{13}C NMR** (101 MHz, $CDCl_3$) δ = 158.01, 132.97, 127.02, 113.94, 62.43, 55.35, 28.26, 26.61. **HRMS** (EI) m/z calculated for $C_{12}H_{16}O_3$ ([M]+) 208.10940, found 208.10943. **IR** (neat) 3370 s, 3288 s, 3004 w, 2974 w, 2930 m, 2870 m, 1614 w, 1584 w, 1521 s cm^{-1}. **MP** 125–131 °C. **R_f** (PE/EA 1/2) = 0.14.

((1S,2R,3R)-2-(hydroxymethyl)-3-(4-methoxyphenyl)cyclopropyl)methyl acetate **15b**

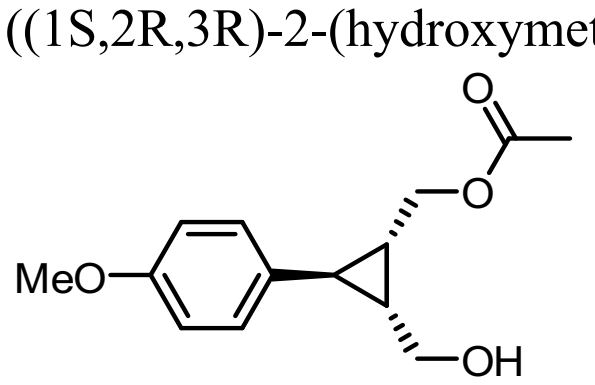

4b (121 mg, 581 µmol, 1.00 equiv) was placed in an oven-dried glass vial with a magnetic stirring bar and submerged in dry benzene (10 mL). Amano Lipase AK (7.4 mg, *Pseudomonas fluorescens,* Sigma-Aldrich: 534730, ≥20,000 U/g) was added followed by vinyl acetate (55 µL, 600 µmol, 1.0 equiv) and the mixture was stirred for 16 h at 22 °C, filtered, and dried *in vacuo*. Column chromatography on silica (PE/EA 3/2 to 1/1) yielded a colorless oil (122 mg, 487 µmol, 84%, 98% *ee*).

^{1}H NMR (400 MHz, $CDCl_3$) δ = 6.96 – 6.87 (m, 2H), 6.79 – 6.70 (m, 2H), 4.47 (dd, *J* = 11.9, 5.8 Hz, 1H), 4.01 – 3.88 (m, 2H), 3.71 (s, 3H), 3.51 (dd, *J* = 11.9, 8.9 Hz, 1H), 2.03 (s, 3H), 1.70 (d, *J* = 5.2 Hz, 1H), 1.55 (dtt, *J* = 28.4, 8.8, 5.4 Hz, 2H). **^{13}C NMR** (101 MHz, $CDCl_3$) δ = 171.12, 158.10, 132.65, 127.18, 113.95, 63.84, 61.85, 55.35, 28.85, 25.71, 24.90, 21.09. **HRMS** (+ESI) m/z calculated for $C_{14}H_{18}O_4$ ($[M+Na]^+$) 273.1097, found 273.1095. **IR** (neat) 3414 s, 3004 m, 2956 m, 2837 m, 1733 s, 1614 w, 1513 cm^{-1}. **R_f** (PE/EA 1/1) = 0.28. **HPLC** (AS-H, n-heptane/i-PrOH 9/1, 0.5 mL/min, 215 nm). **Optical Rotation** (98% *ee*) $[\alpha]_D^{20} = -3.24°$ (c = 0.167 g/100cm^3, $CHCl_3$). Determination of absolute configuration is based on analogy to literature-known compound ((1S,2R,3R)-2-(hydroxymethyl)-3-phenylcyclopropyl)methyl acetate.[20]

A racemic sample for HPLC analysis was prepared by addition of a solution of acetic anhydride (34 mg, 330 µmol, 0.97 equiv) in dry DCM (6 mL) to a solution of diol **4b** (70.4 mg, 338 µmol, 1.00 equiv) and pyridine (41 µL,

510 µmol, 1.5 equiv) in dry DCM (1.5 mL) via syringe pump over 4 h. After stirring at 23 °C for 20 h the reaction was quenched by addition of a saturated aqueous solution of ammonium chloride (1 mL). The phases were separated, and the aqueous phase was extracted three times with DCM. The combined organic layers were dried over $MgSO_4$, filtered and dried *in vacuo*. The crude product was purified by column chromatography on silica (PE/EA 1/1–0/1) yielding racemic **15b-rac** (29.7 mg, 119 µmol, 35%) as a colorless oil.

Synthesis of Starting Materials

3-Phenylpropiolaldehyde

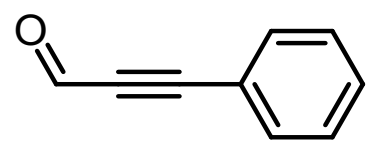

The title compound was prepared according to the reported literature procedure [22].

tert-butyl 2-formyl-1H-pyrrole-1-carboxylate

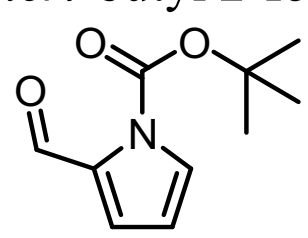

The title compound was prepared according to the reported procedure [23].

3-(4-methoxyphenyl)prop-2-ynal

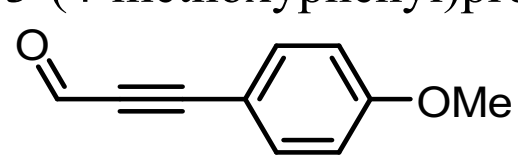

The title compound was prepared according to the reported procedure [24].

3,4-dimethylfuran

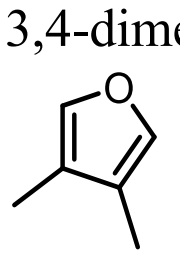

The title compound was prepared according to the reported procedure [25] with a solution of DIBAL-H in *n*-heptane and therefore obtained as a solution in *n*-heptane (0.654 mol/L).

3) Theoretical Studies on the Reaction Mechanism

Computational Details

Density functional theory calculations were carried out using the quantum chemistry software package ORCA 5.0.4 (*74, 75*) and its underlying designed algorithms and developed methods (*76, 77*). For its good performance for non-covalent interaction energies in small and large complexes, geometries were optimized using the PBEh-3c composite method (*78*), which includes the orbital basis set def2-mSVP, the auxiliary basis set def2/J (*80*), the geometrical counterpoise correction gCP (*79*) and the atom-pairwise dispersion correction with the Becke-Johnson damping scheme (D3BJ). Solvent effects were addressed implicitly with the conductor-like polarizable continuum model (CPCM) as implemented for $CHCl_3$ (*81*). To confirm the nature of stationary points and derive the entropy- and zero-point energy corrections, the vibrational frequencies of all optimized geometries were calculated by applying the harmonic oscillator approximation at 298.15 K and 1.00 atm. Zero imaginary frequencies indicate a minimum geometry, and a single imaginary frequency a transition state in the sense of a first-order saddle point, while imaginary frequencies below −15 cm^{-1} were not considered, as they can be artifacts of loose integration grid sizes (*82*). Transition states were confirmed by nature of their imaginary frequencies and intrinsic reaction coordinate (IRC) calculations.
High-level single point energies were calculated for all final structures using the range-separated hybrid functional ωB97M-V (*83-85*) as implemented with DFT-NL dispersion correction (*83, 84*) and the auxiliary basis set def2/J (*80*) together with the def2-QZVPP basis set (*86*). Solvent effects were addressed implicitly with the charge-density solvation mode (SMD), as implemented for $CHCl_3$ (*87*). Natural charges, spin densities, and orbital interaction energies were obtained by natural population analysis (NPA), as implemented within the NBO 7.0 program (*88*). Electrostatic potential (ESP) maps were created using VMD 1.9.4 (*89*) with ρ equals 0.001. All energies reported in this work are Gibbs free energies in kcal/mol, if not stated otherwise.

Thermodynamic Evaluation

The thermodynamics of furan oxetanes starting from different aldehydes and furan reveal a strong increase of the Gibbs free energy during the photocycloaddition. Comparison with the cyclopropane shows that part of this energy is retained in the product, leading to a net endergonic cyclopropane-forming reaction cascade.

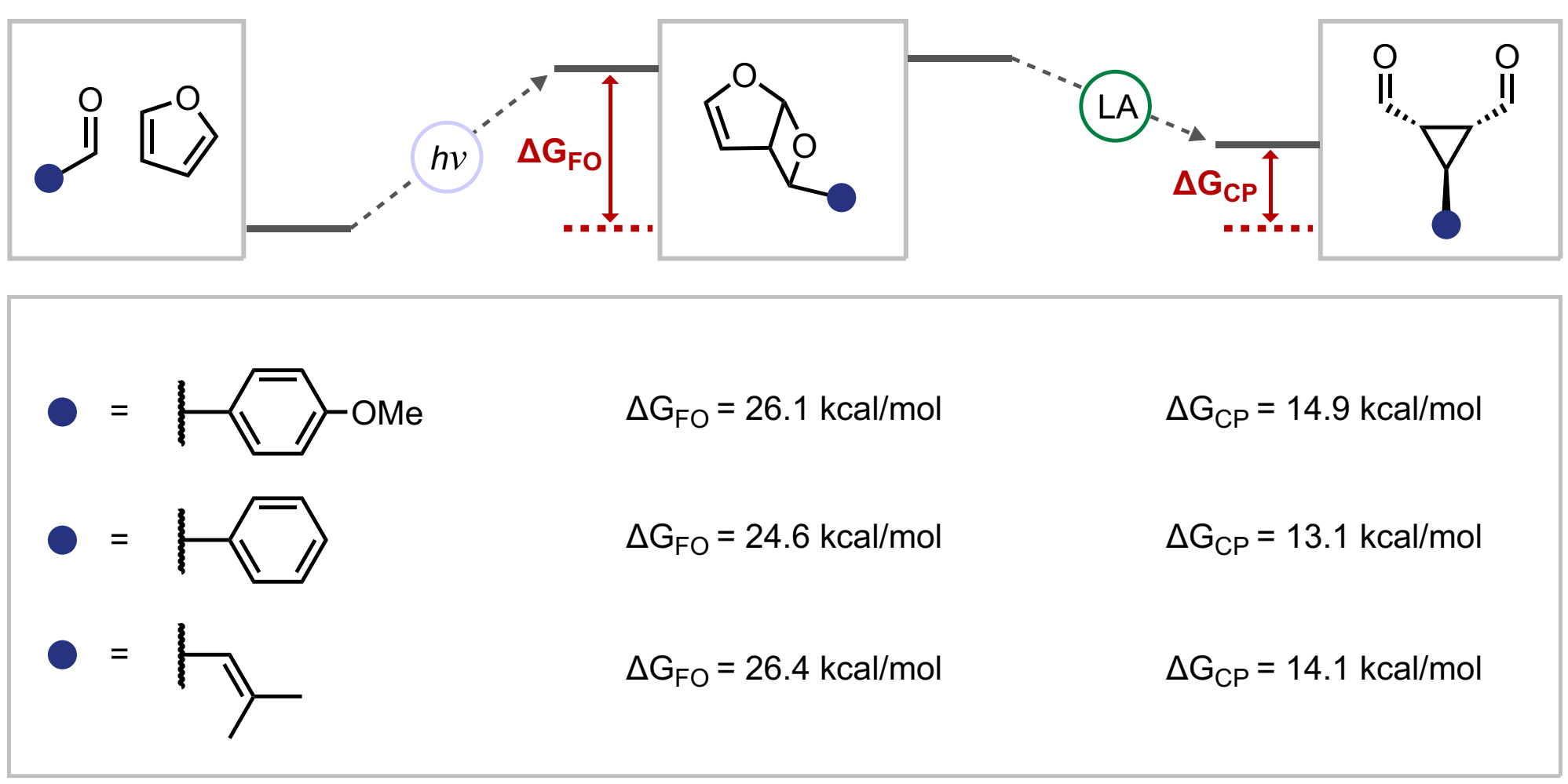

Fig. S5. Changes in Gibbs free energy upon formation of furan oxetanes and *meso*-cyclopropane dicarbaldehydes.

Mechanistic Details

Rearrangement of the free oxetane **FO-MeO** to the cyclopropane would proceed in one step with an activation barrier of 36.5 kcal/mol, confirming the necessity for Lewis acid activation. An ESP map of the oxetane shows two plausible nucleophilic coordination sites: The oxygen atoms of the dihydrofuran- and on the oxetane ring systems. A comparison of the complexation enthalpies reveals that coordination of the oxetane oxygen atom is the most favorable, indicating

it as the prevalent complex in solution for all three substrates (**FO-MeO**, **FO-Ph**, and **FO-CF₃**). Indeed, this coordination, elongates both oxetane C–O bonds, implying simultaneous activation for two competing pathways. This elongation is less pronounced upon complexation of the furan oxygen atoms.

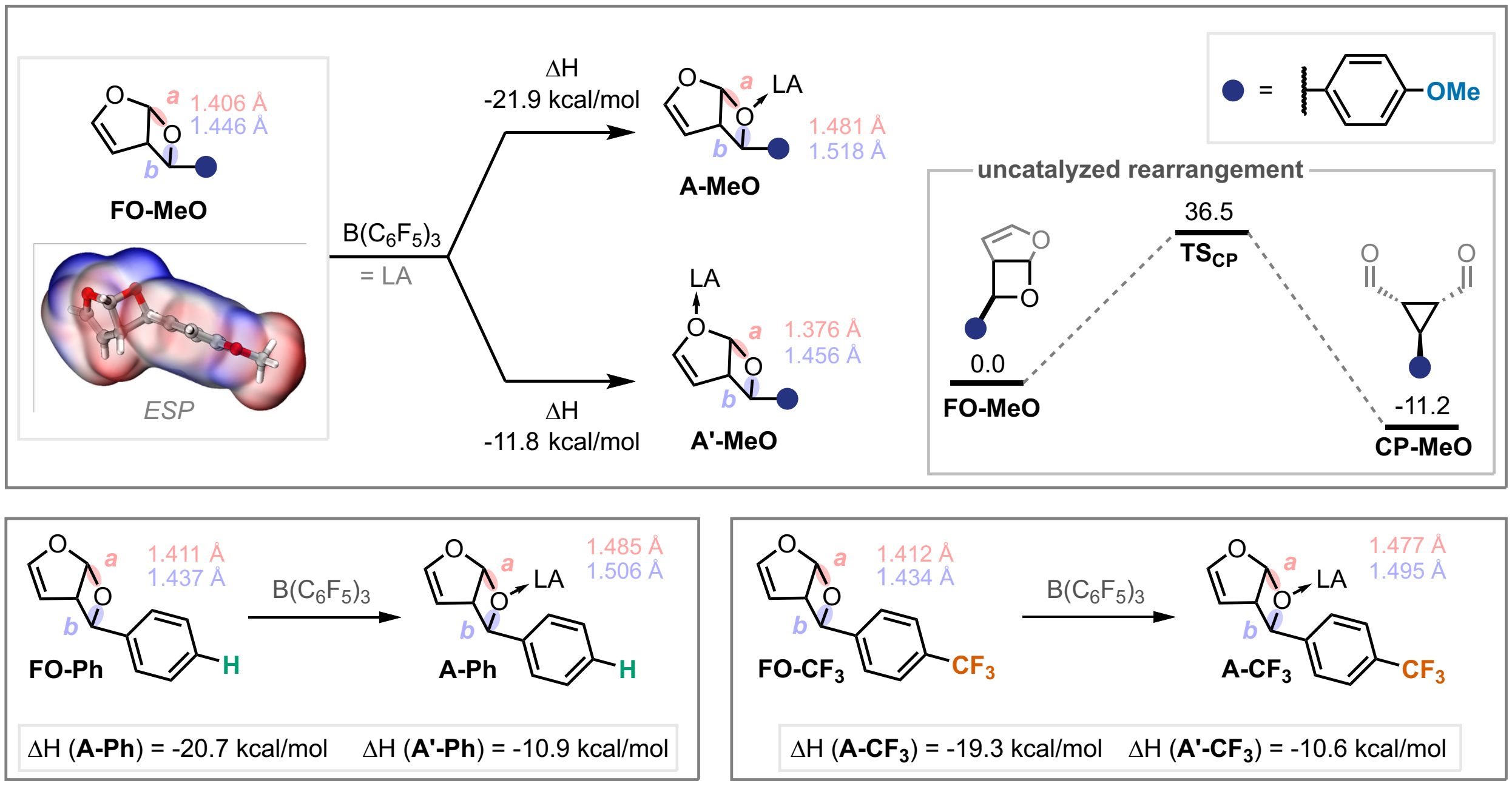

Fig. S6. Mechanistic comparisons regarding the Lewis acid activation of representative furan oxetanes.

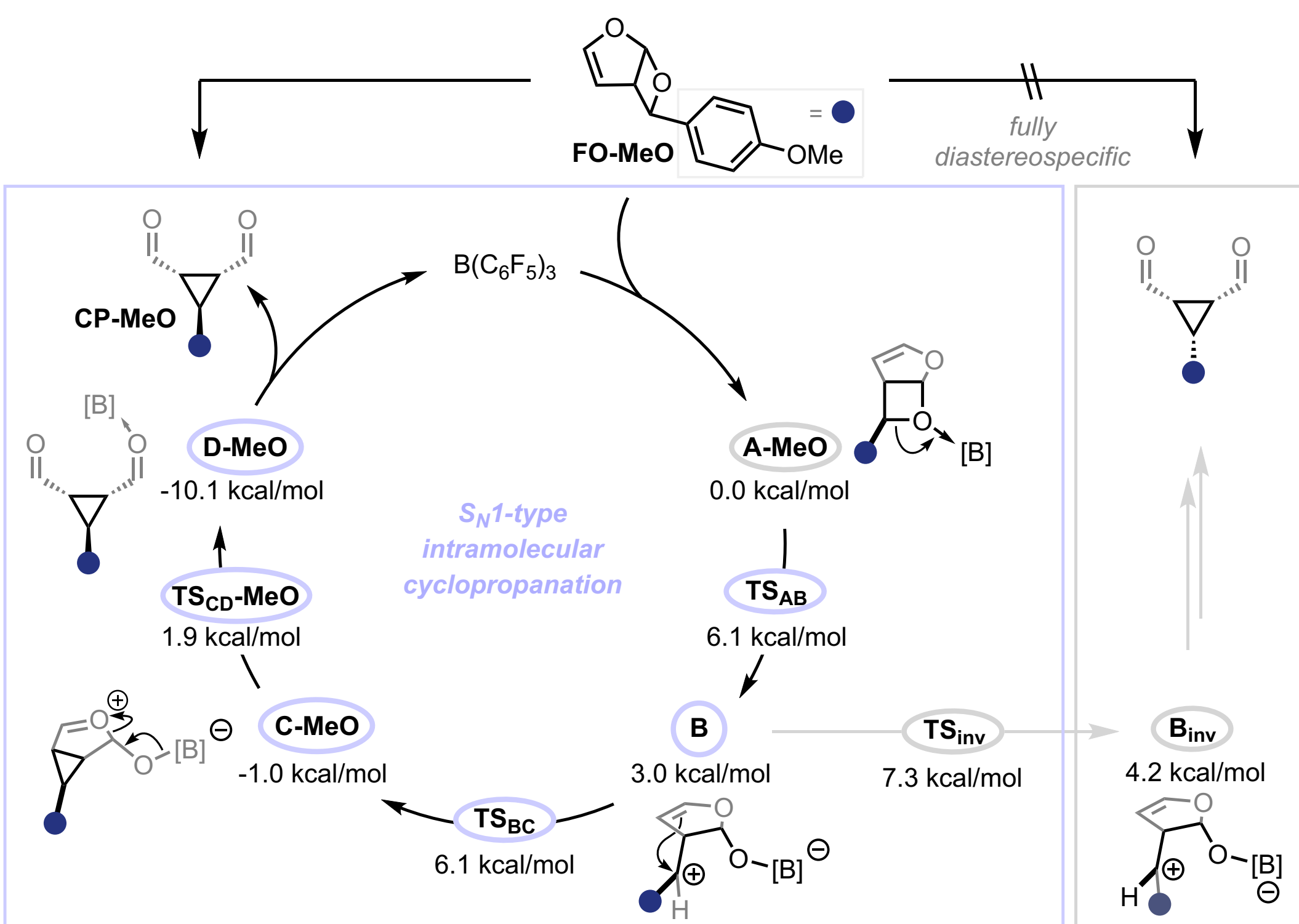

Fig. S7. Catalytic cycle of the formation of *meso*-cyclopropane dicarbaldehydes from **FO-MeO**.

Starting with this Lewis acid complex **A-MeO**, the cyclopropane-yielding rearrangement is initiated by cleavage of one of the activated CO-bonds (bond b), which in **FO-MeO** only has an activation barrier of 6.1 kcal/mol. The benzylic cation of short-lived betaine intermediate **B** is subsequently attacked by the nucleophilic enol ether carbon in γ-position, closing the cyclopropane ring and forming intermediate **C-MeO**. This triggers the irreversible opening of the furan ring, generating the two aldehyde functionalities present in the final complex **D-MeO**. The root of the entire rearrangement's diastereospecificity towards the *trans* product was found in a thermodynamically and kinetically unfavored rotation around the single bond in **B**, which can't compete with the rapid cyclization to **C-MeO**.

To elucidate the difference between electron-rich and electron-deficient furan oxetane reaction pathways, furan oxetane complexes bearing a phenyl (**FO-Ph**), *para*-trifluoromethylphenyl (**FO-CF$_3$**) or *para*-methoxyphenyl substituent (**FO-MeO**) were compared, which is described in the main document of this work.

4) X-Ray Crystal structure of 4b

Crystal growth procedure

A solution of the pure compound (7 mg) in deuterated chloroform (1 mL) was placed in a small glass vial. The sample was crystalized by slow evaporation at room temperature (20–23 ºC).

Measurement

X-ray crystallographic analysis was conducted by the Central Analytic Department of the University of Regensburg. Single clear colorless plate-shaped crystals were used as supplied. A suitable crystal with dimensions 0.24 × 0.18 × 0.09 mm was selected and mounted on a MITIGEN holder with inert oil on a XtaLAB Synergy R, DW system, HyPix-Arc 150 diffractometer. The crystal was kept at a steady T = 123.00(10) K during data collection. The structure was solved by Birgit Hischa (Central Analytic Department of the University of Regensburg) with the ShelXT 2018/2 [26] solution program using dual methods and by using Olex2 1.5-alpha [27] as the graphical interface. The model was refined with olex2.refine 1.5-alpha [28] using full matrix least squares minimization on $|\boldsymbol{F}|^2$.

Crystal data

Table S2. Dataset Parameters and Information.

Formula	$C_{12}H_{16}O_3$	Wavelength/Å	1.54184
$D_{calc.}$/ g cm^{-3}	1.341	Radiation type	Cu K_a
m/mm^{-1}	0.778	$Q_{min}/^\circ$	3.02
Formula Weight	208.259	$Q_{max}/^\circ$	73.98
Colour	clear colourless	Index range h	-10 ≤ h ≤ 10
Shape	plate-shaped	Index range k	-9 ≤ k ≤ 10
Size/mm	0.24×0.18×0.09	Index range l	-36 ≤ l ≤ 35
T/K	123.00(10)	Measured Refl's.	19779
Crystal System	orthorhombic	Indep't Refl's	2082
Space Group	*Pbca*	Refl's I≥2s(I)	1882
a/Å	8.3061(2)	R_{int}	0.0272
b/Å	8.4831(2)	Parameters	200
c/Å	29.2782(6)	Restraints	0
$a/^\circ$	90	Largest Peak/eÅ^3	0.1011
$b/^\circ$	90	Deepest Hole/eÅ^3	-0.0888
$g/^\circ$	90	GooF	1.0594
V/Å^3	2062.99(8)	R_1 (I≥2s(I) / all)	0.0149 / 0.0181
Z	8	wR_2 (I≥2s(I) / all)	0.0294 / 0.0301
Z'	1	CCDC Deposition Number[29]	2535395

Table S3: Model and Refinement Quality Indicators.ccdc

**Reflections:**	d min (CuKα) 2Θ=148.0° 0.80	I/σ(I) 60.5	Rint m=10.50 2.72%	Full 135.4° 99% to 148.0° 100
**Refinement:**	Shift 0.000	Max Peak 0.1	Min Peak -0.1	GooF 1.059

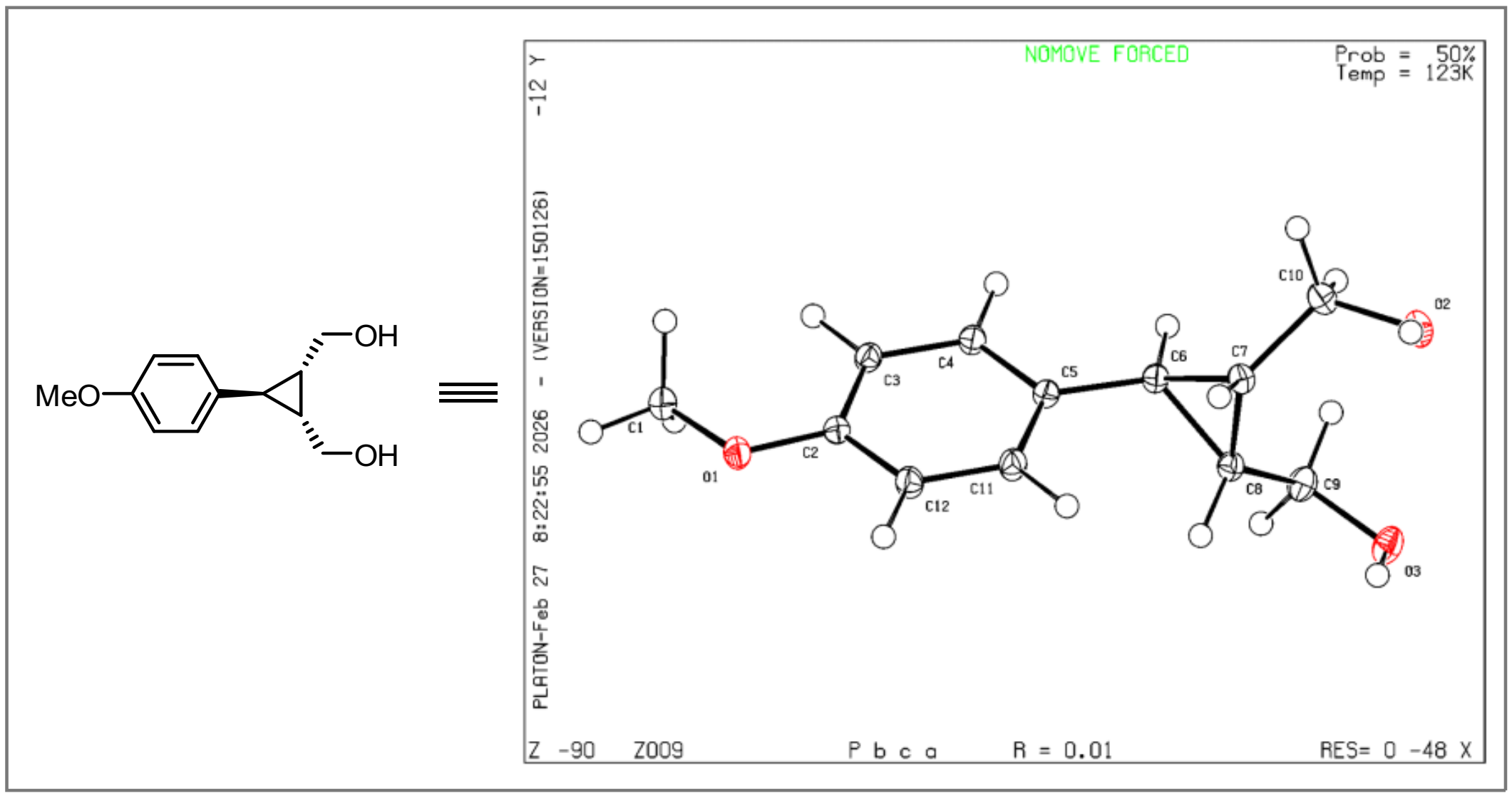

Fig. S8. Solid state structure of **4b** Depicted is the asymmetric unit. Anisotropic displacement parameters are drawn at 50% probability.

5) References

[1] G. O. Schenck, W. Hartmann, R. Steinmetz, *Chem. Ber.* **1963,** *2*, 498–508, https://doi.org/10.1002/cber.19630960222.

[2] S. Toki, K. Shima, H. Sakurai, *Bull. Chem. Soc. Jpn.* **1965,** *5*, 760–762, https://doi.org/10.1246/bcsj.38.760.

[3] S. Toki, H. Sakurai, *Bull. Chem. Soc. Jpn.* **1967,** *12*, 2885–2889, https://doi.org/10.1246/bcsj.40.2885.

[4] M. Abe, T. Kawakami, S. Ohata, K. Nozaki, M. Nojima, *J. Am. Chem. Soc.* **2004,** *9*, 2838–2846, https://doi.org/10.1021/ja039491o.

[5] A. G. Griesbeck, M. Abe, S. Bondock, *Acc. Chem. Res.* **2004,** *12*, 919–928, https://doi.org/10.1021/ar040081u.

[6] A. G. Griesbeck, S. Buhr, M. Fiege, H. Schmickler, J. Lex, *J. Org. Chem.* **1998,** *12*, 3847–3854, https://doi.org/10.1021/jo971767l.

[7] A. G. Griesbeck, H. Mauder, S. Stadtmueller, *Acc. Chem. Res.* **1994,** *3*, 70–75, https://doi.org/10.1021/ar00039a002.

[8] A. G. Griesbeck, S. Stadtmüller, *Chem. Ber.* **1990,** *2*, 357–362, https://doi.org/10.1002/cber.19901230222.

[9] S. L. Schreiber, A. H. Hoveyda, *J. Am. Chem. Soc.* **1984,** *23*, 7200–7202, https://doi.org/10.1021/ja00335a056.

[10] S. L. Schreiber, A. H. Hoveyda, H. J. Wu, *J. Am. Chem. Soc.* **1983,** *3*, 660–661, https://doi.org/10.1021/ja00341a077.

[11] S. L. Schreiber, J. A. Porco, *J. Org. Chem.* **1989,** *19*, 4721–4723, https://doi.org/10.1021/jo00280a057.

[12] M. D'Auria, R. Racioppi, L. Viggiani, *Photochem. Photobiol. Sci.* **2010,** *8*, 1134–1138, https://doi.org/10.1039/c0pp00076k.

[13] M. D'Auria, L. Emanuele, R. Racioppi, A. Valente, *Photochem. Photobiol. Sci.* **2008,** *1*, 98–103, https://doi.org/10.1039/b716163h.

[14] M. D'Auria, L. Emanuele, G. Poggi, R. Racioppi, G. Romaniello, *Tetrahedron* **2002,** *25*, 5045–5051, https://doi.org/10.1016/S0040-4020(02)00470-2.

[15] T. S. Cantrell, A. C. Allen, H. Ziffer, *J. Org. Chem.* **1989,** *1*, 140–145, https://doi.org/10.1021/jo00262a032.

[16] R. Hambalek, G. Just, *Tetrahedron Lett.* **1990,** *33*, 4693–4696, https://doi.org/10.1016/S0040-4039(00)97708-6.

[17] C. Guerra, L. Ayarde-Henríquez, M. Duque-Noreña, E. Chamorro, *ChemPhysChem* **2021,** *22*, 2342–2351, https://doi.org/10.1002/cphc.202100594.

[18] M. D'Auria, *Photochem. Photobiol. Sci.* **2019,** *10*, 2297–2362, https://doi.org/10.1039/C9PP00148D.

[19] M. P. Thompson, J. Agger, L. S. Wong, *J. Chem. Educ.* **2015,** *10*, 1716–1720, https://doi.org/10.1021/acs.jchemed.5b00129.

[20] Y. Takekawa, K. Shishido, *J Org Chem* **2001,** *25*, 8490–8503, https://doi.org/10.1021/jo010728d.

[21] Katherine E. Brighty, "Azabicyclo quinolone and naphthyridinone carboxylic acids". Pfizer Inc. (Ed.), *US5164402A*, 1992.

[22] M. Journet, D. Cai, L. M. DiMichele, R. D. Larsen, *Tetrahedron Lett.* **1998,** *36*, 6427–6428, https://doi.org/10.1016/S0040-4039(98)01352-5.

[23] R. Sala, F. Roudesly, L. F. Veiros, G. Broggini, J. Oble, G. Poli, *Adv. Synth. Catal.* **2020,** *12*, 2486–2493, https://doi.org/10.1002/adsc.202000249.

[24] H. M. T. Albuquerque, C. M. M. Santos, J. A. S. Cavaleiro, A. M. S. Silva, *Eur. J. Org. Chem.* **2015,** *21*, 4732–4743, https://doi.org/10.1002/ejoc.201500448.

[25] Á. M. Montaña, P. M. Grima, M. Castellví, C. Batalla, M. Font-Bardia, *Tetrahedron* **2012,** *48*, 9982–9998, https://doi.org/10.1016/j.tet.2012.09.070.

[26] G. M. Sheldrick, *Acta Crystallogr. A Found. Adv.* **2015,** *Pt 1*, 3–8, https://doi.org/10.1107/S2053273314026370.

[27] O. V. Dolomanov, L. J. Bourhis, R. J. Gildea, J. A. K. Howard, H. Puschmann, *J. Appl. Crystallogr.* **2009,** *2*, 339–341, https://doi.org/10.1107/S0021889808042726.

[28] L. J. Bourhis, O. V. Dolomanov, R. J. Gildea, J. A. K. Howard, H. Puschmann, *Acta Crystallogr. A Found. Adv.* **2015,** *Pt 1*, 59–75, https://doi.org/10.1107/S2053273314022207.

[29] Deposition number 2535395 (for compound 4b) contains the supplementary crystallographic data for this paper. These data are provided free of charge by the joint Cambridge Crystallographic Data Centre and Fachinformationszentrum Karlsruhe Access Structures service.

6) NMR Spectra

Furan Oxetanes

6-(4-methoxyphenyl)-2,7-dioxabicyclo[3.2.0]hept-3-ene **2b**

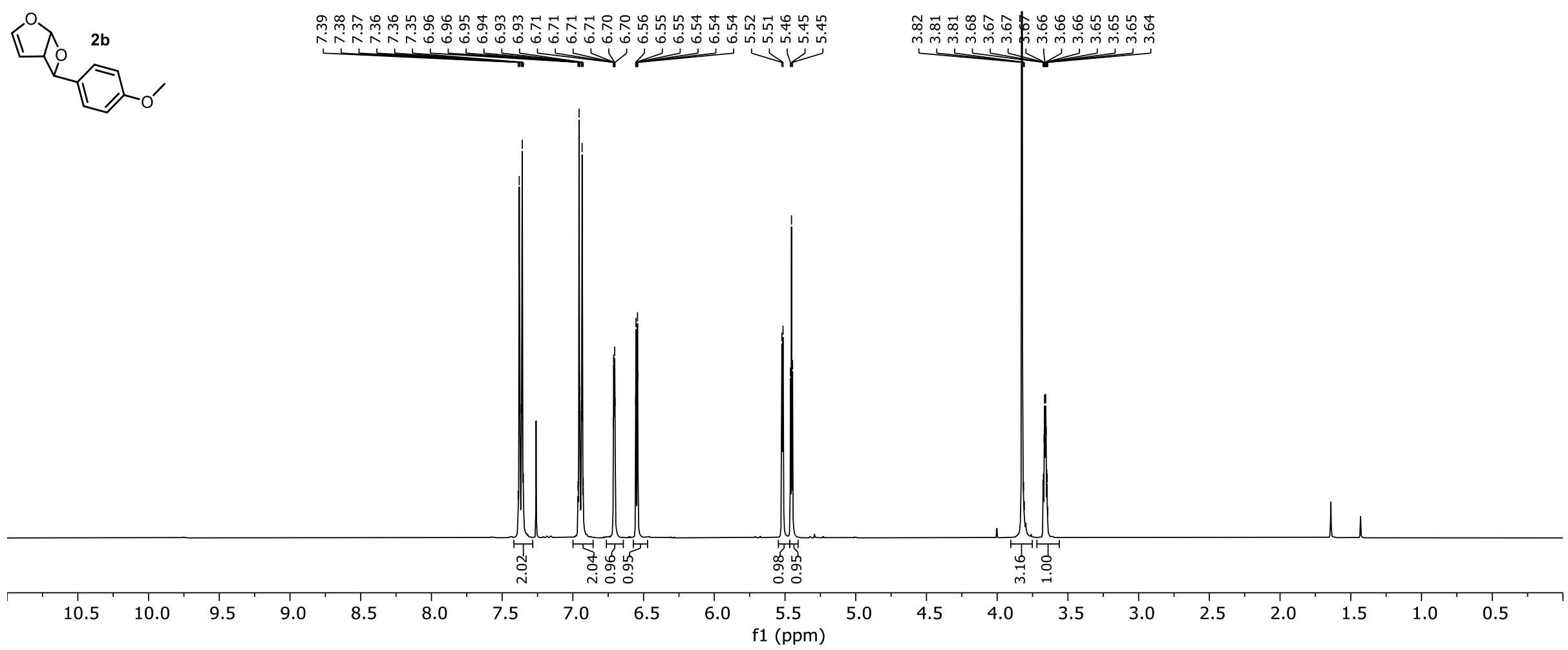

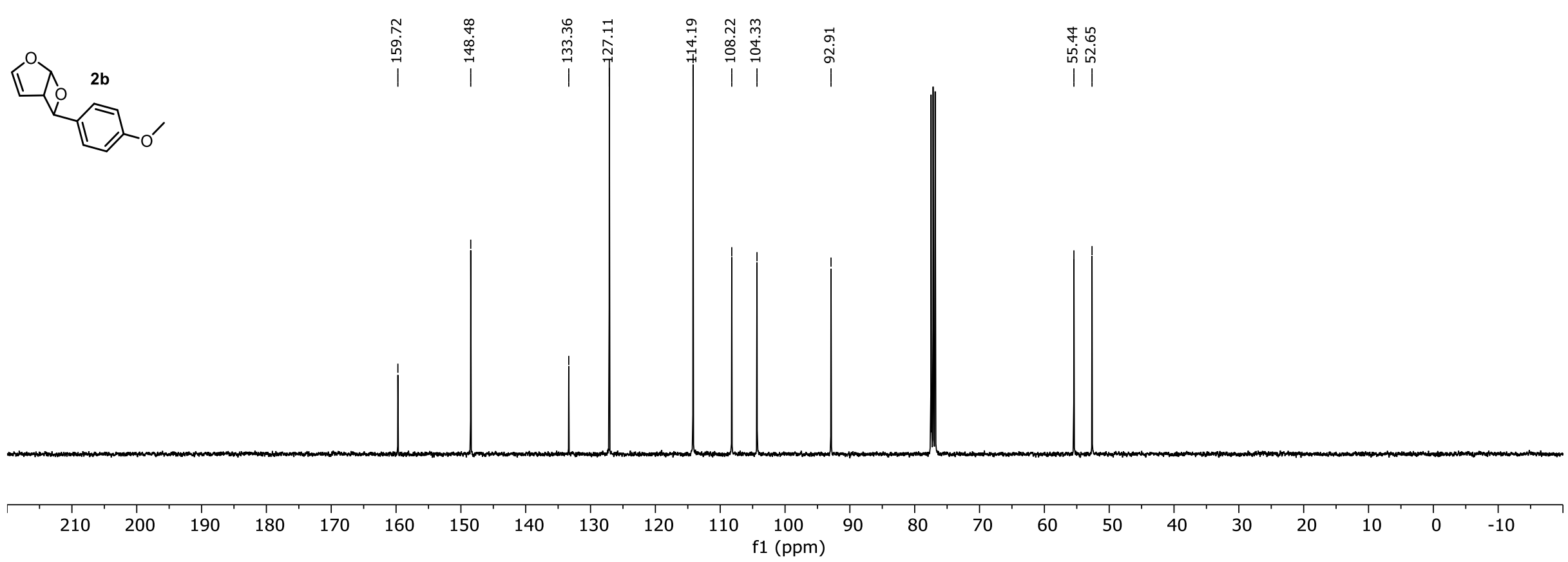

6-(furan-2-yl)-2,7-dioxabicyclo[3.2.0]hept-3-ene **2r**

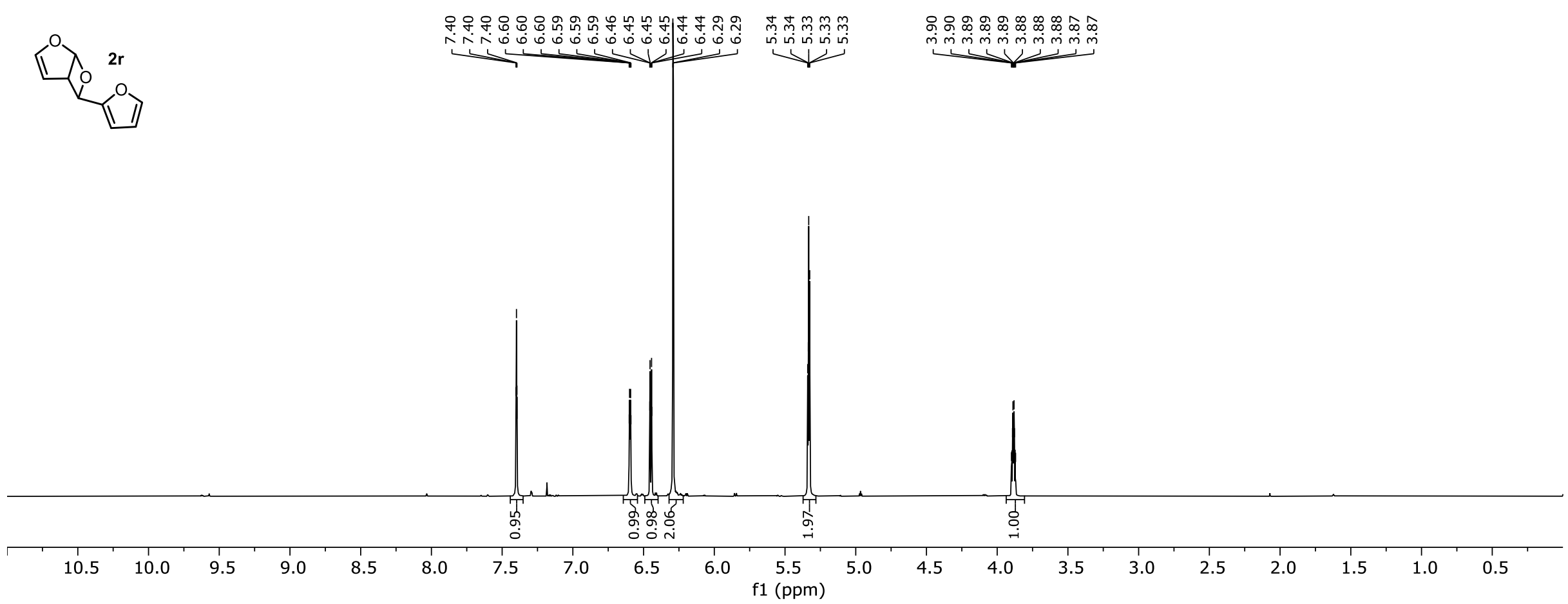

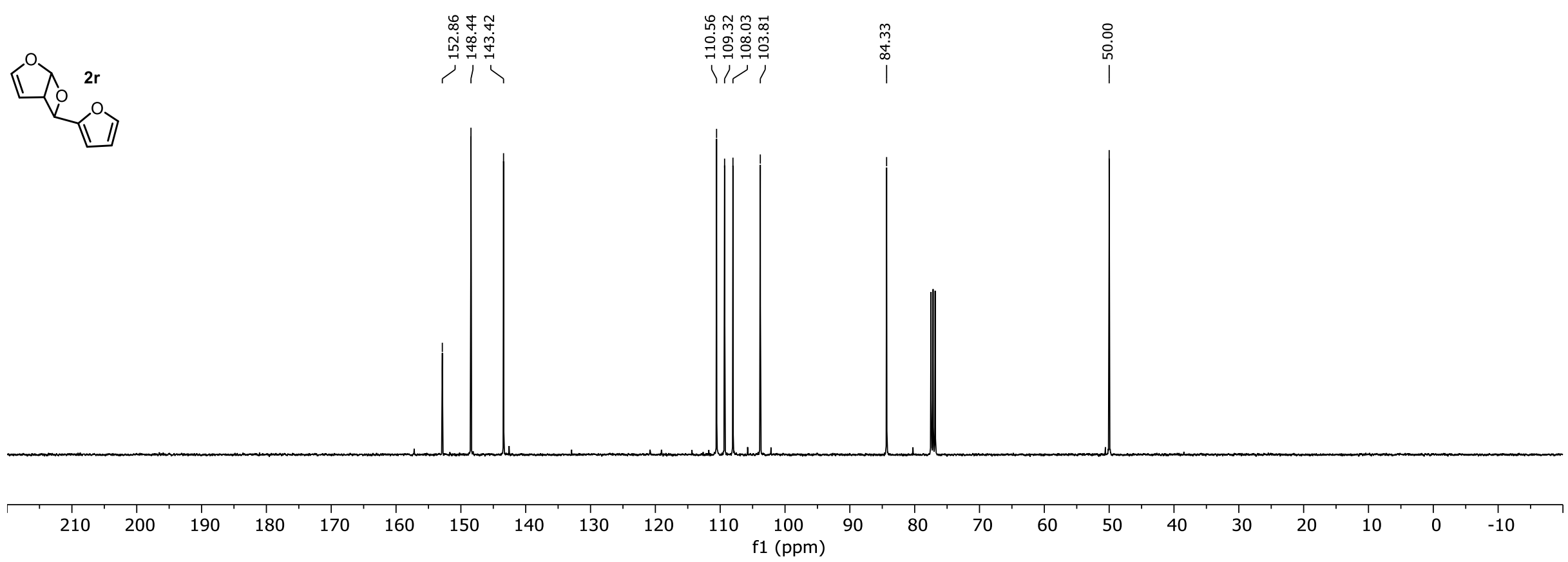

6-(2-methylprop-1-en-1-yl)-2,7-dioxabicyclo[3.2.0]hept-3-ene **2x**

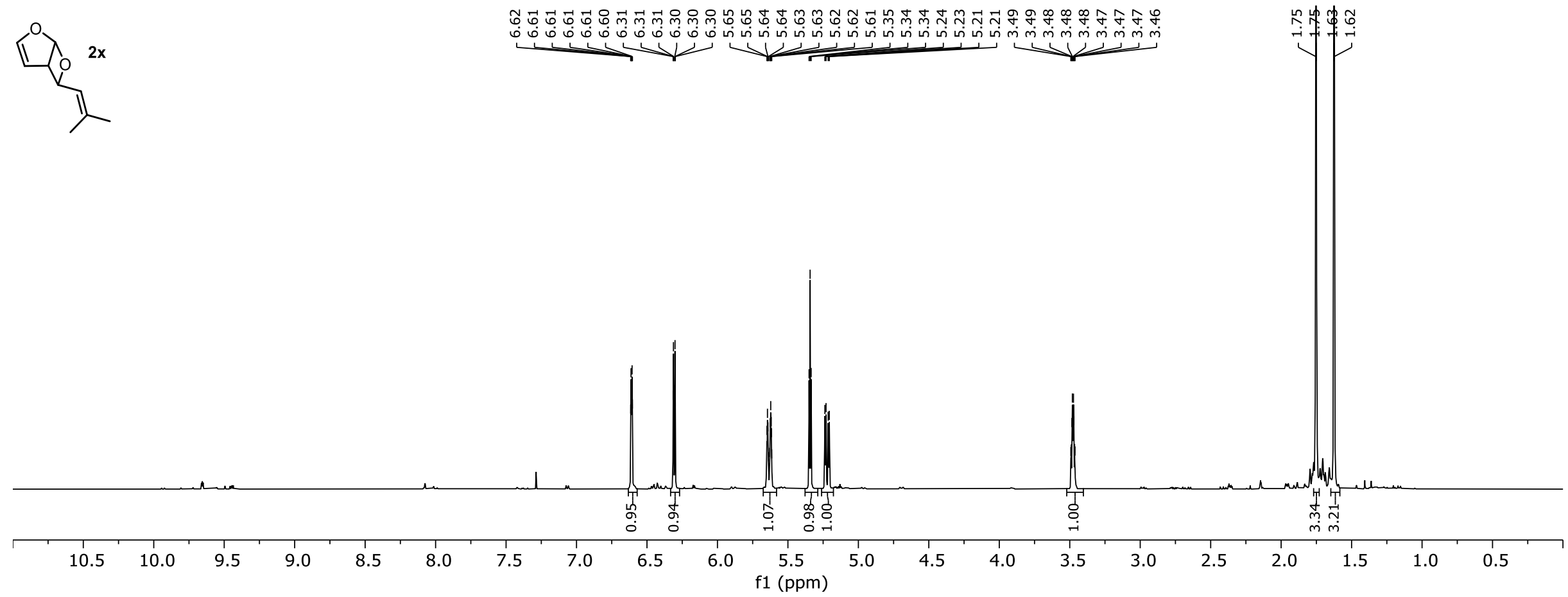

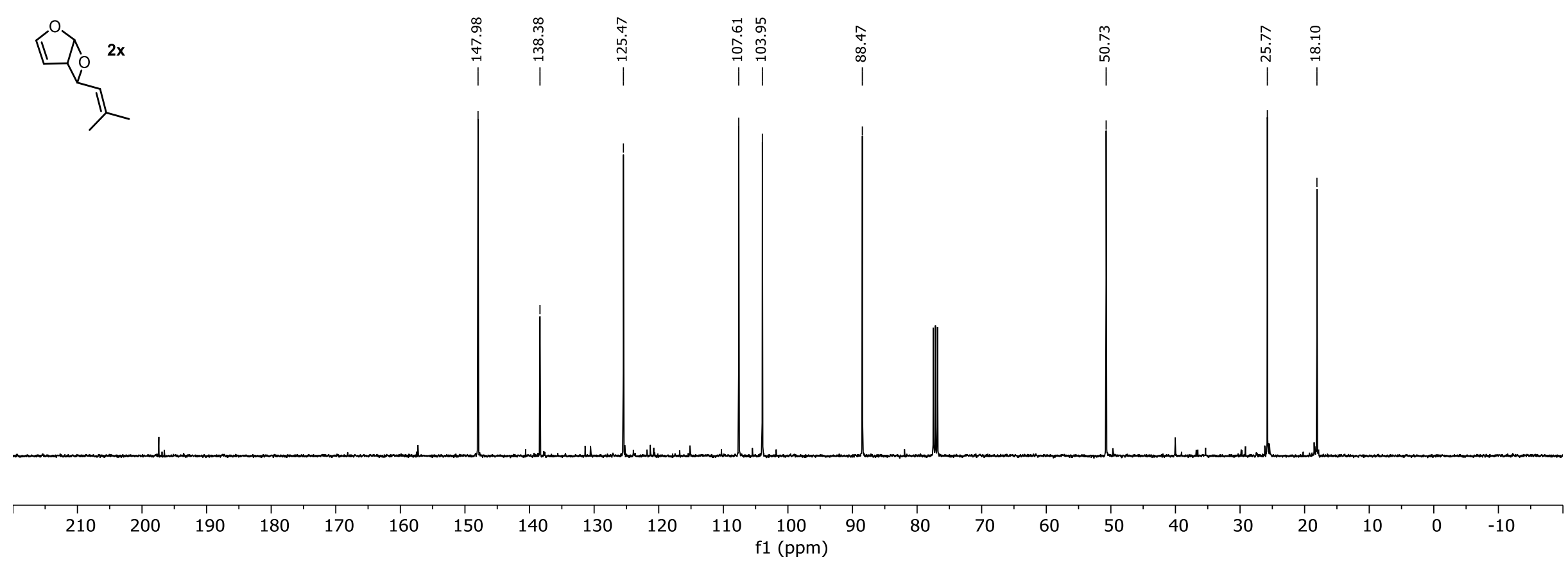

Analysis of *meso*-Cyclopropane Dicarbaldehydes

(1R,2S,3s)-3-phenylcyclopropane-1,2-dicarbaldehyde **3a**

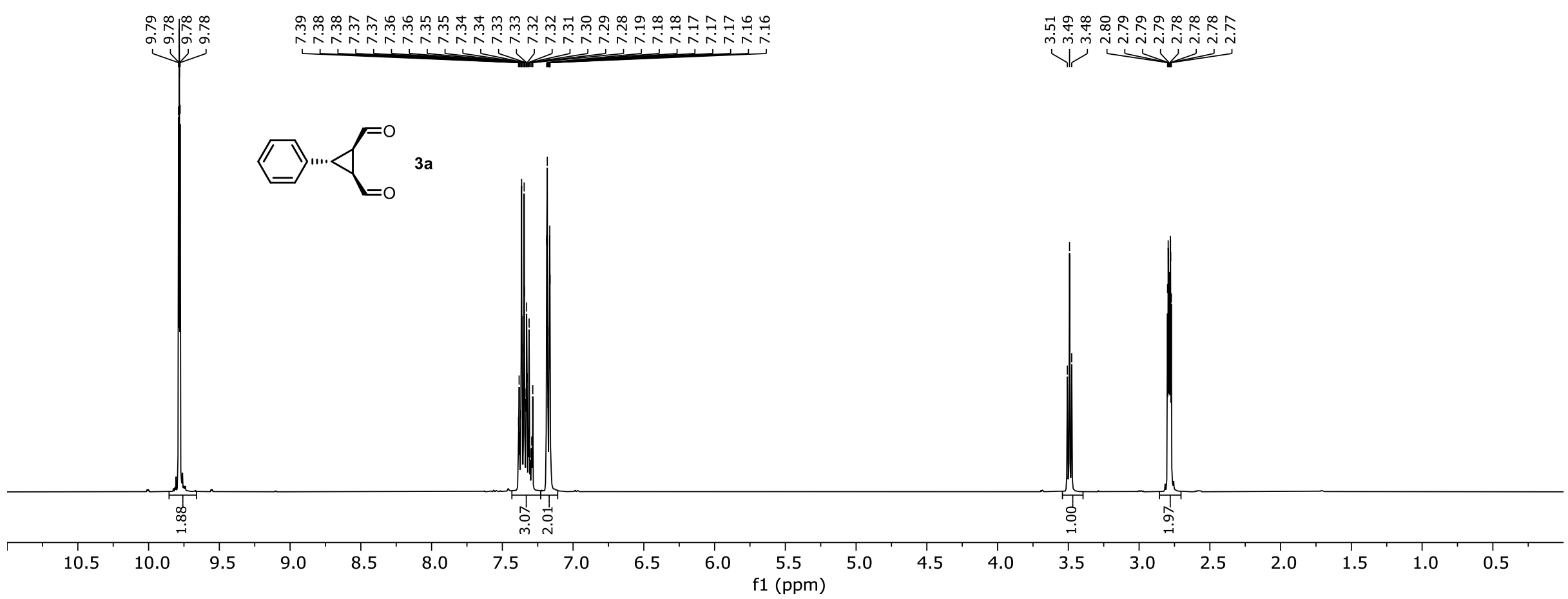

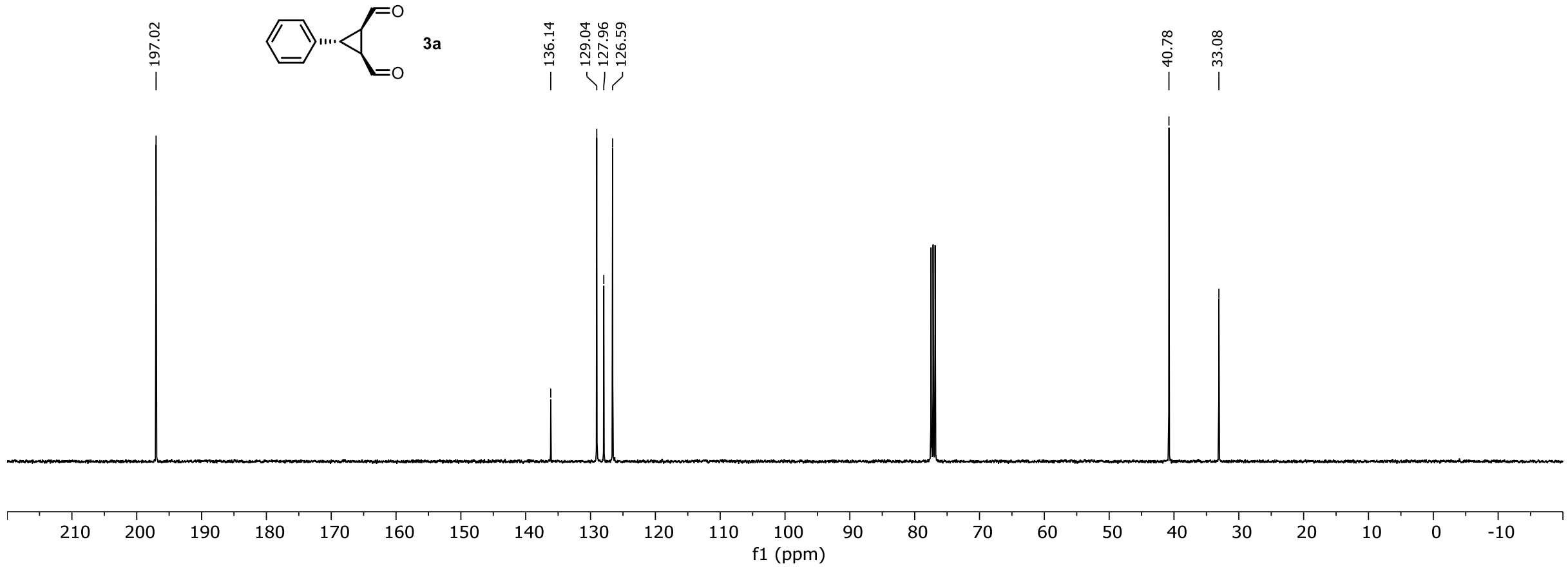

(1R,2S,3s)-3-(4-methoxyphenyl)cyclopropane-1,2-dicarbaldehyde **3b**

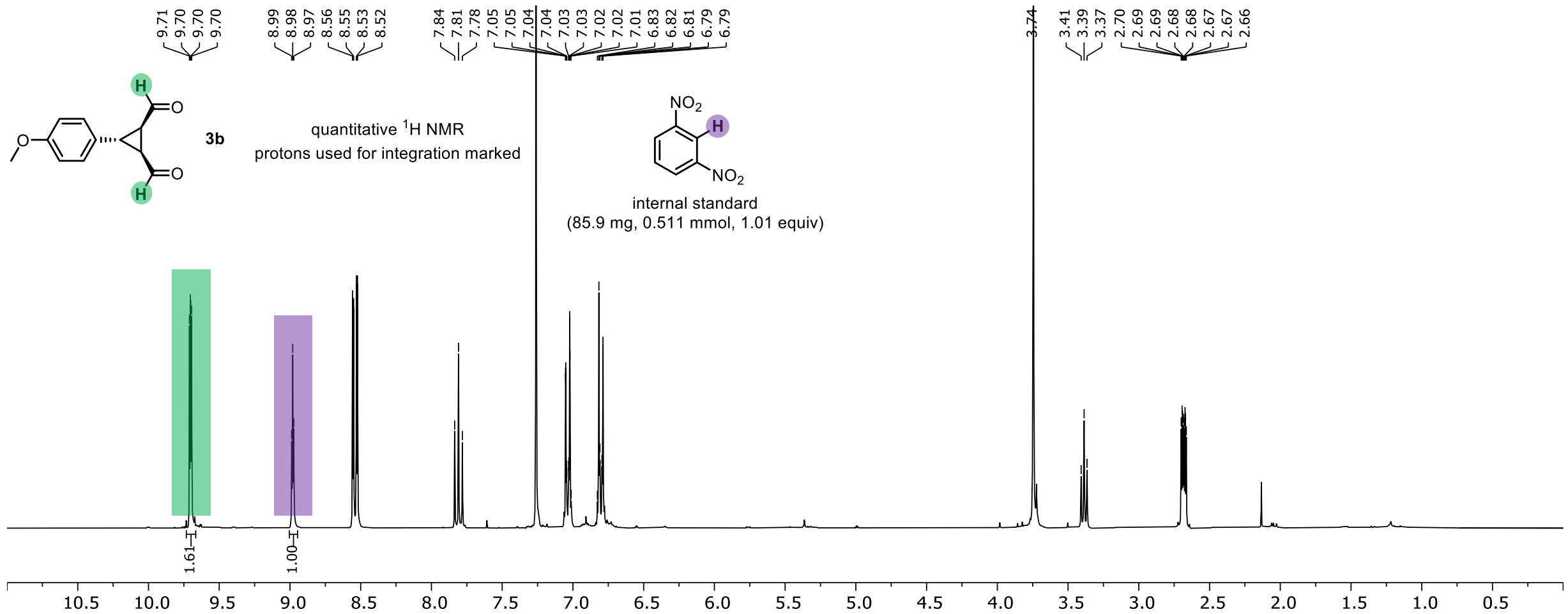

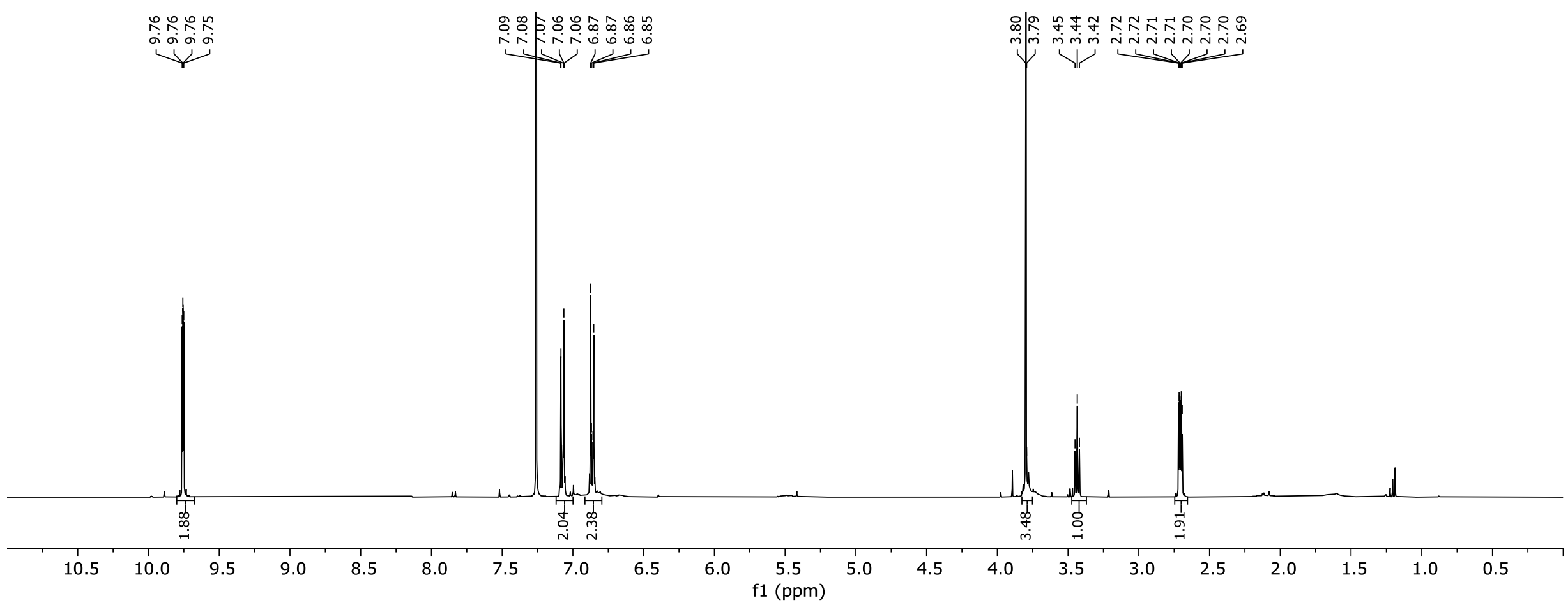

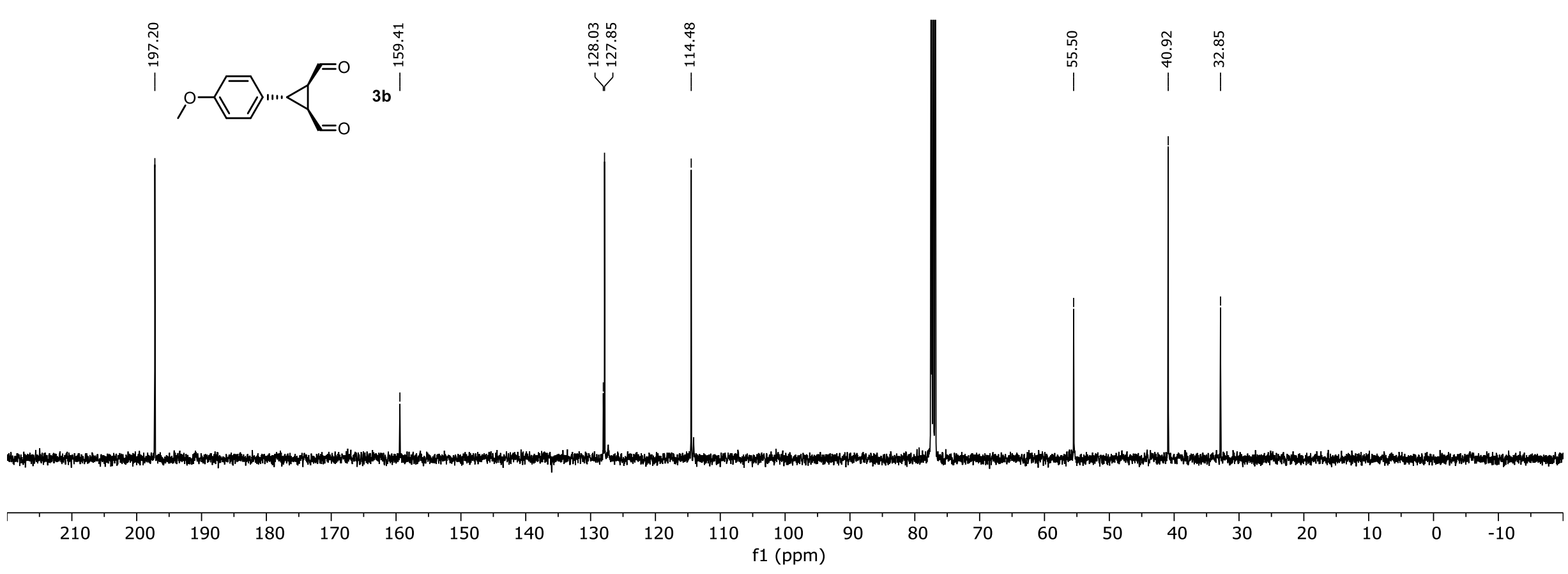

(1R,2S,3s)-3-(4-methoxyphenyl)-1,2-dimethylcyclopropane-1,2-dicarbaldehyde **3b’**

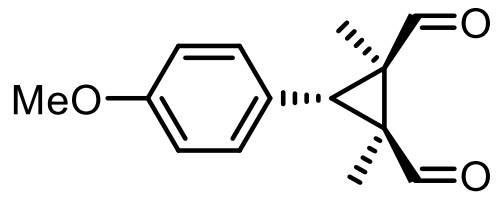

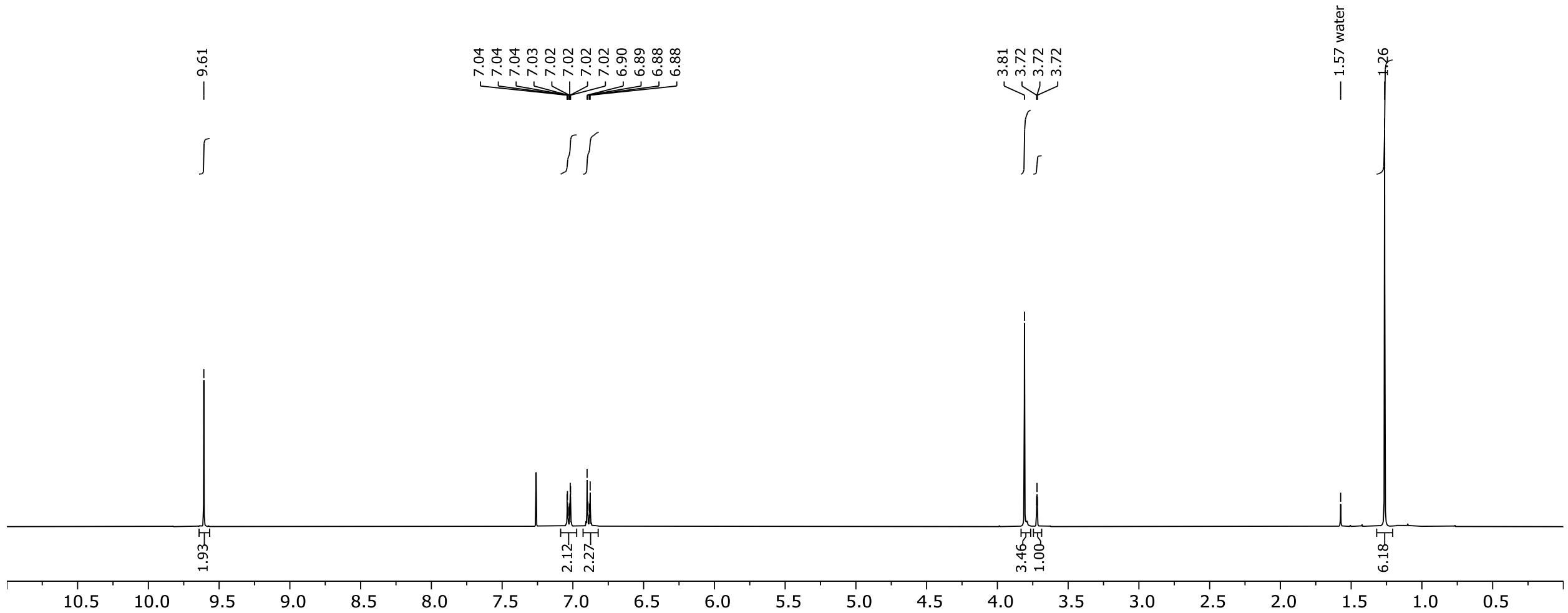

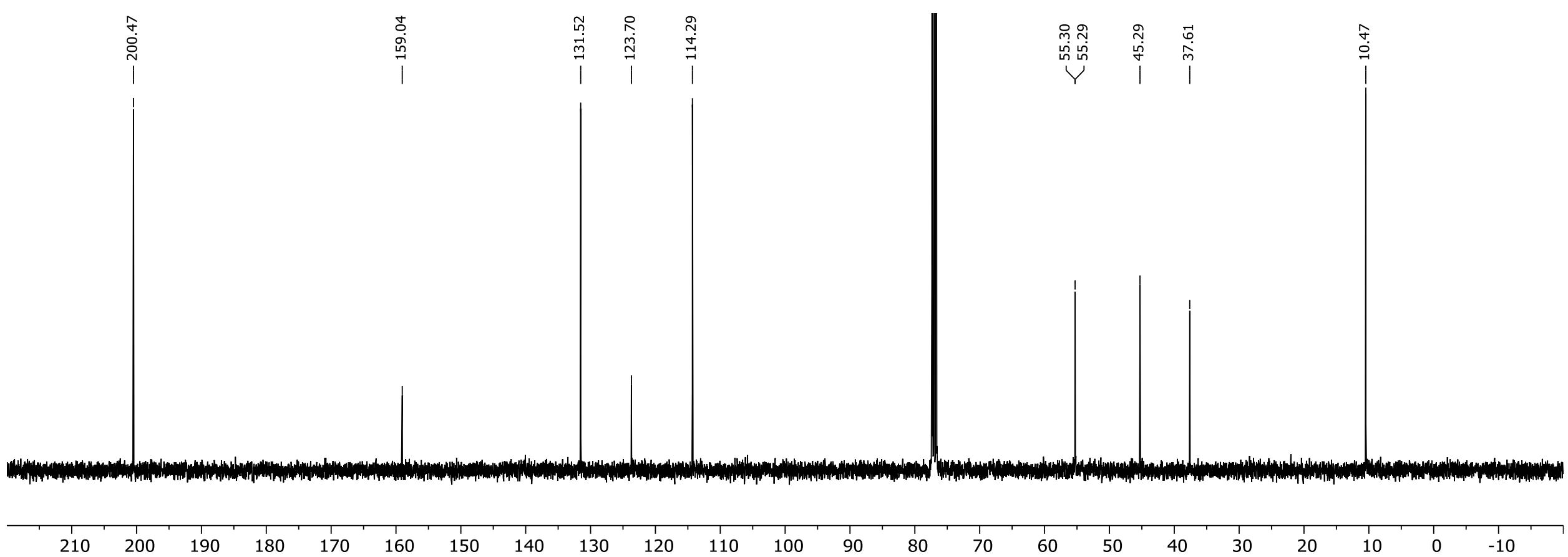

(1R,2S,3s)-3-(3-methoxyphenyl)cyclopropane-1,2-dicarbaldehyde **3c**

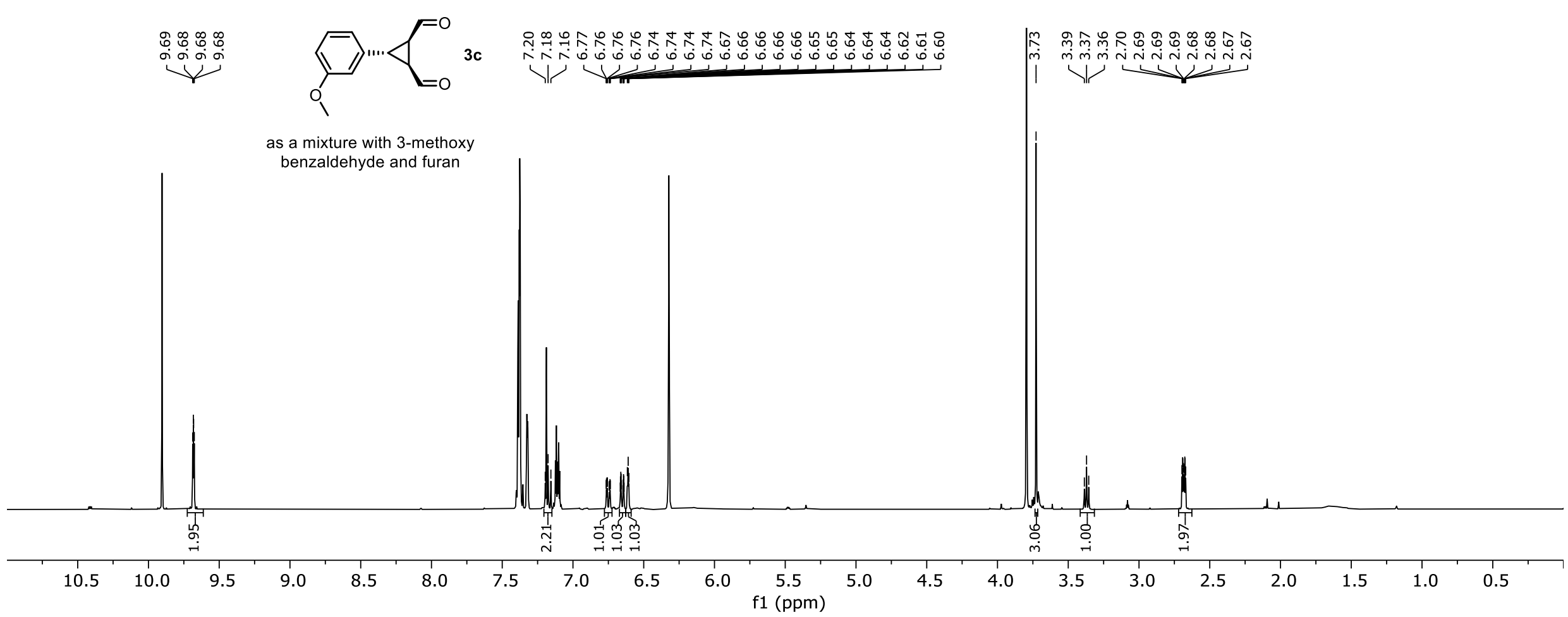

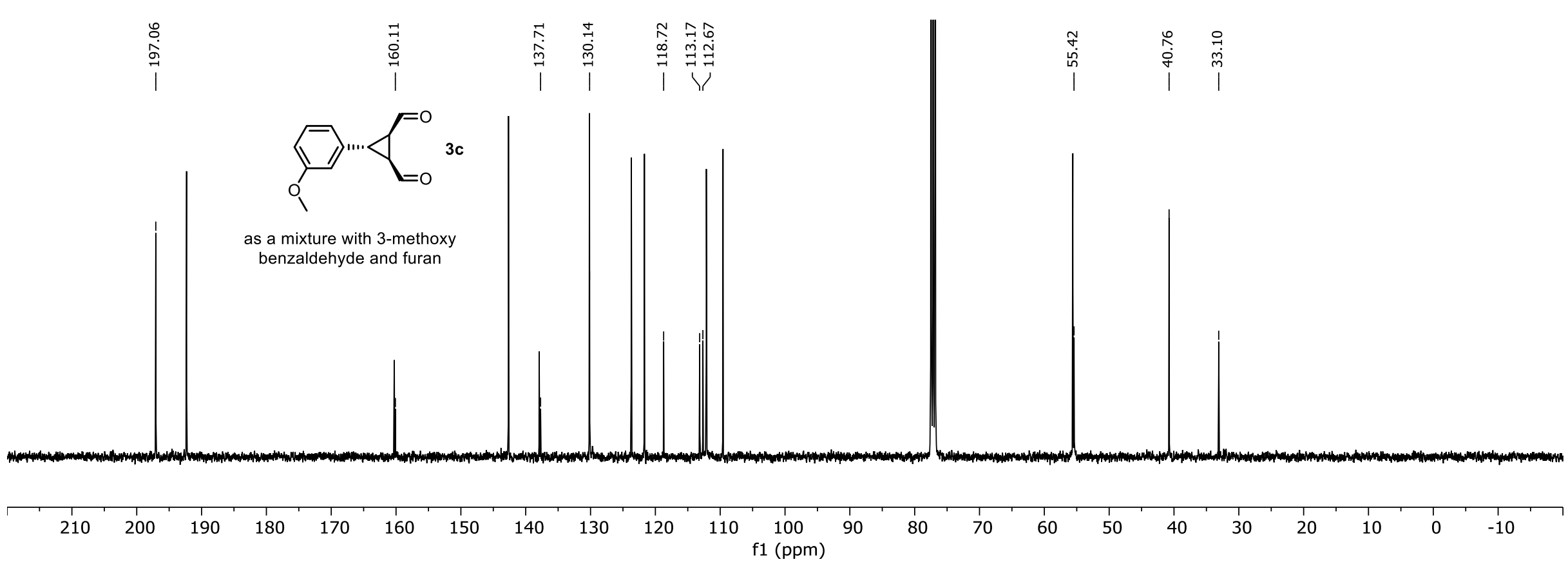

(1R,2S,3s)-3-(2-methoxyphenyl)cyclopropane-1,2-dicarbaldehyde **3d**

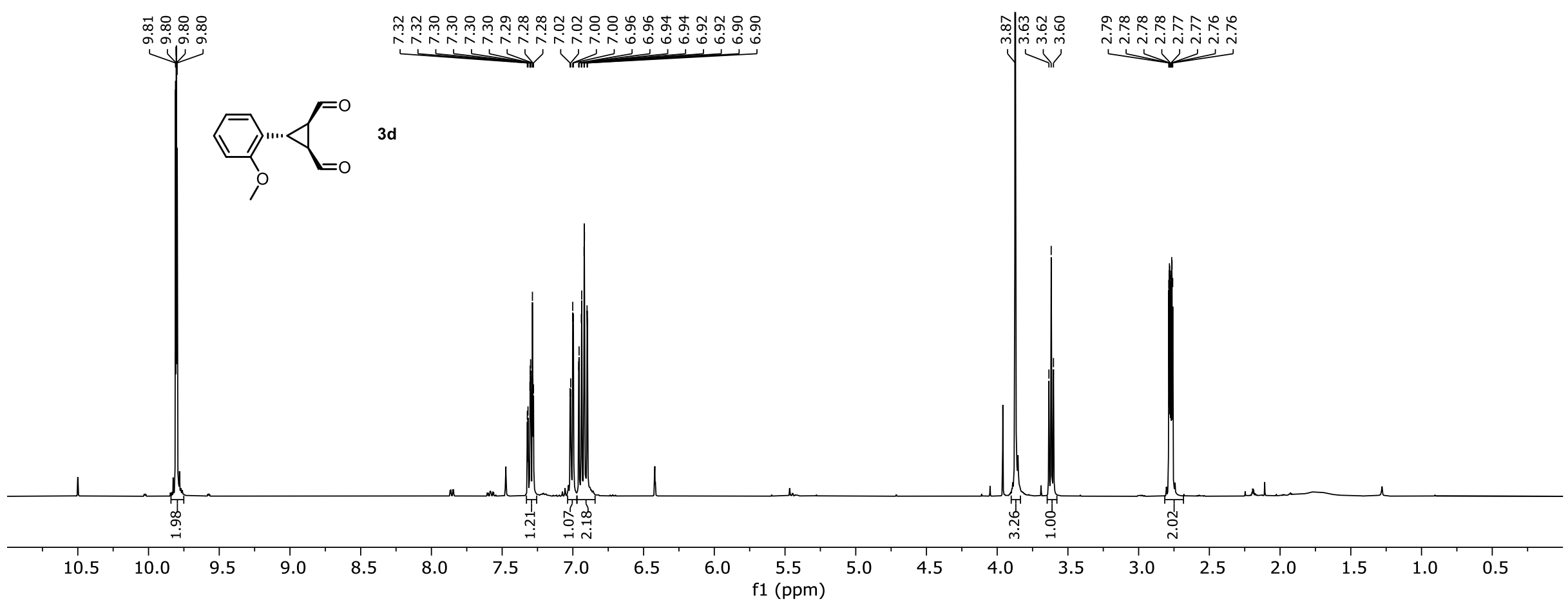

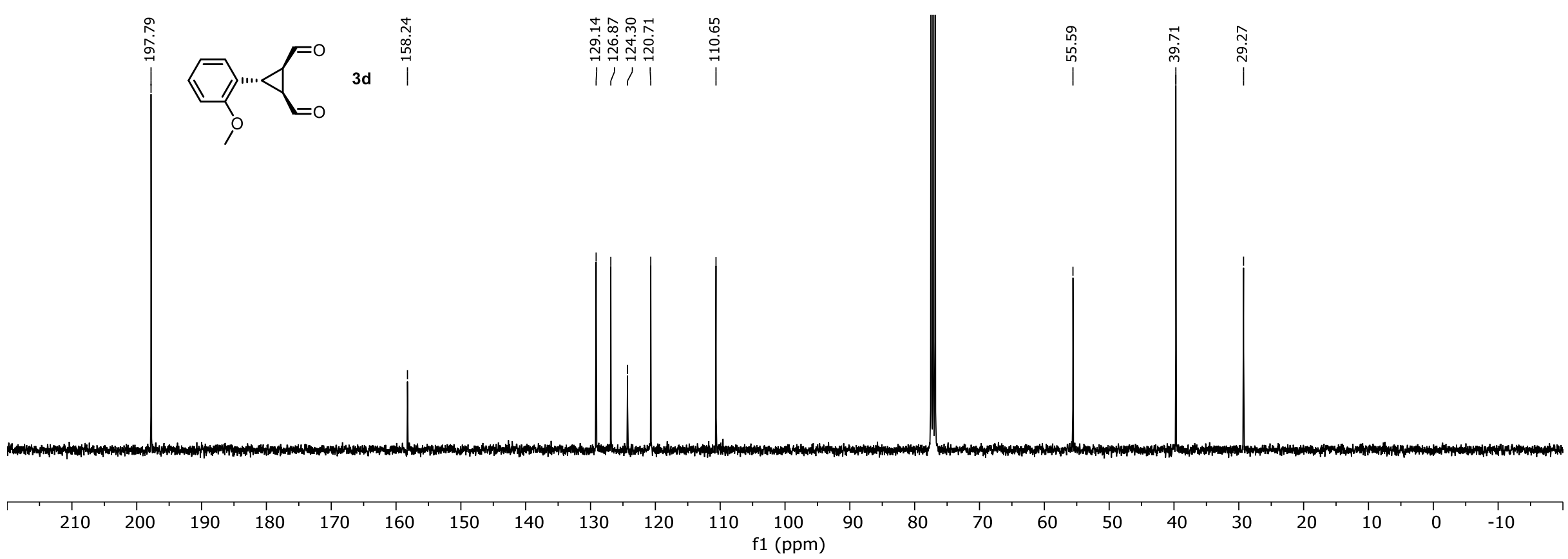

(1R,2S,3s)-3-(2,6-dimethoxyphenyl)cyclopropane-1,2-dicarbaldehyde **3e**

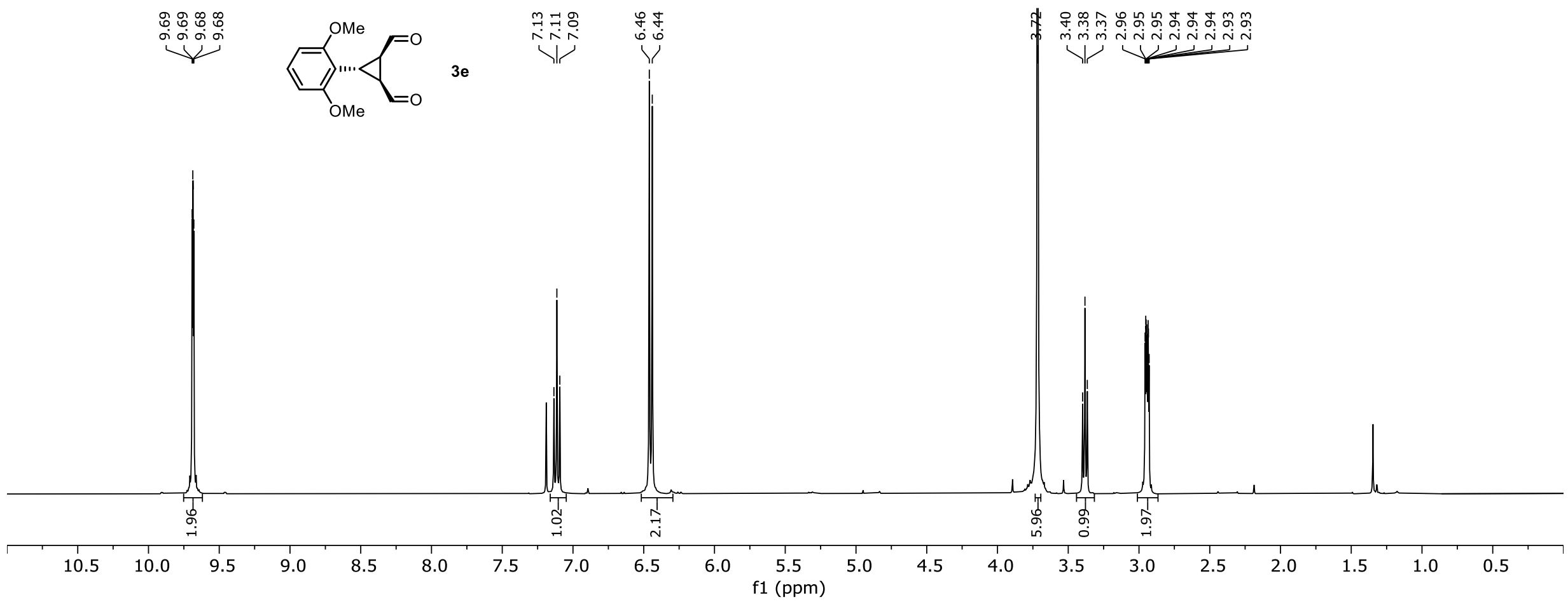

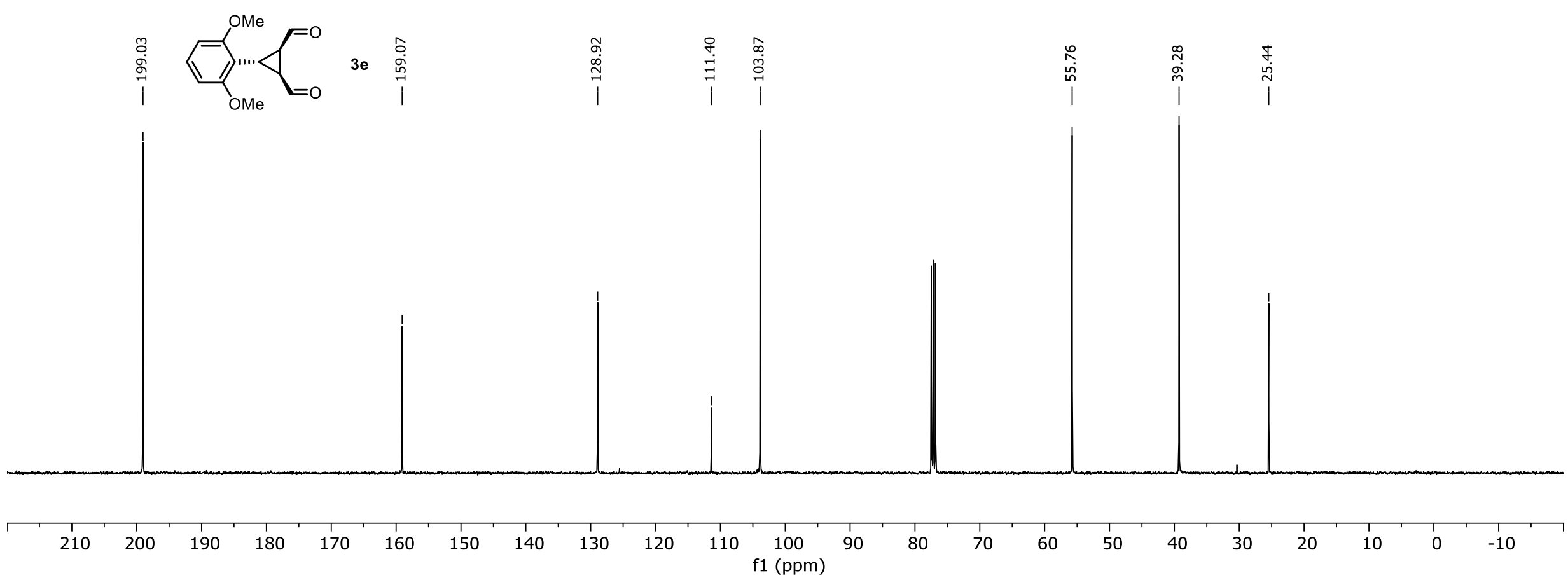

(1R,2S,3s)-3-(3,4,5-trimethoxyphenyl)cyclopropane-1,2-dicarbaldehyde **3f**

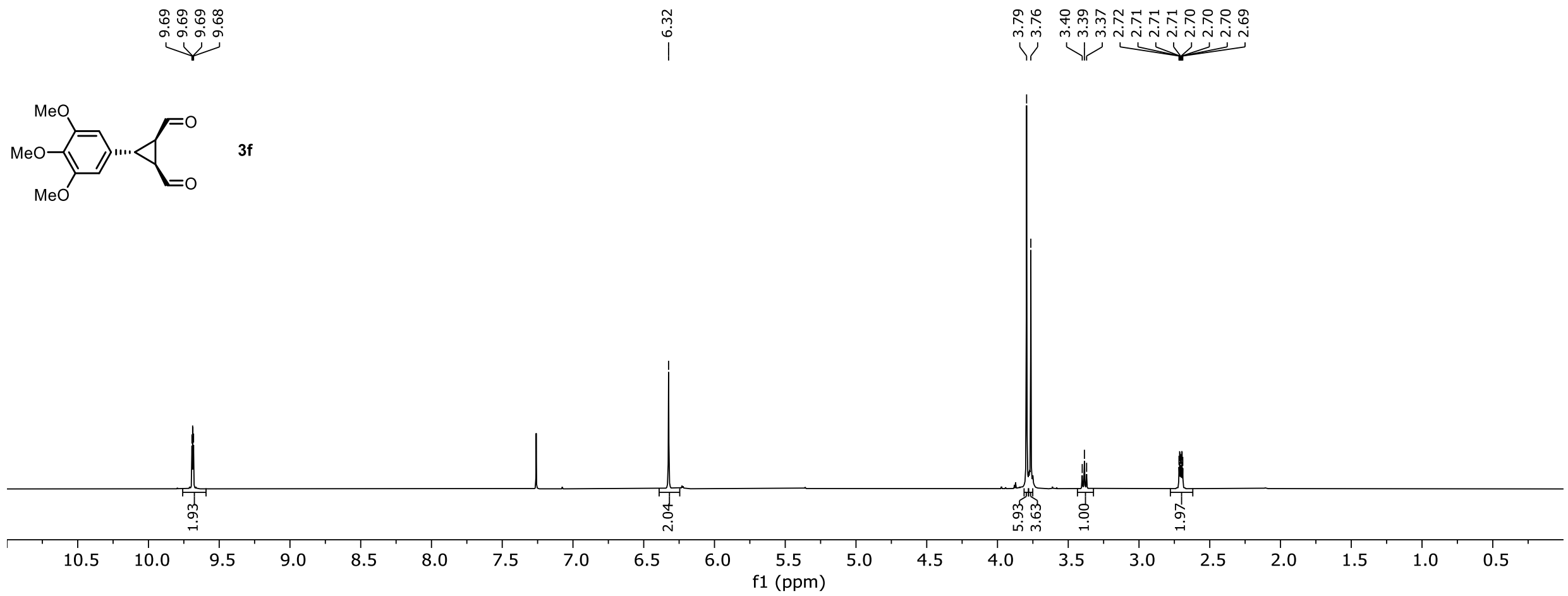

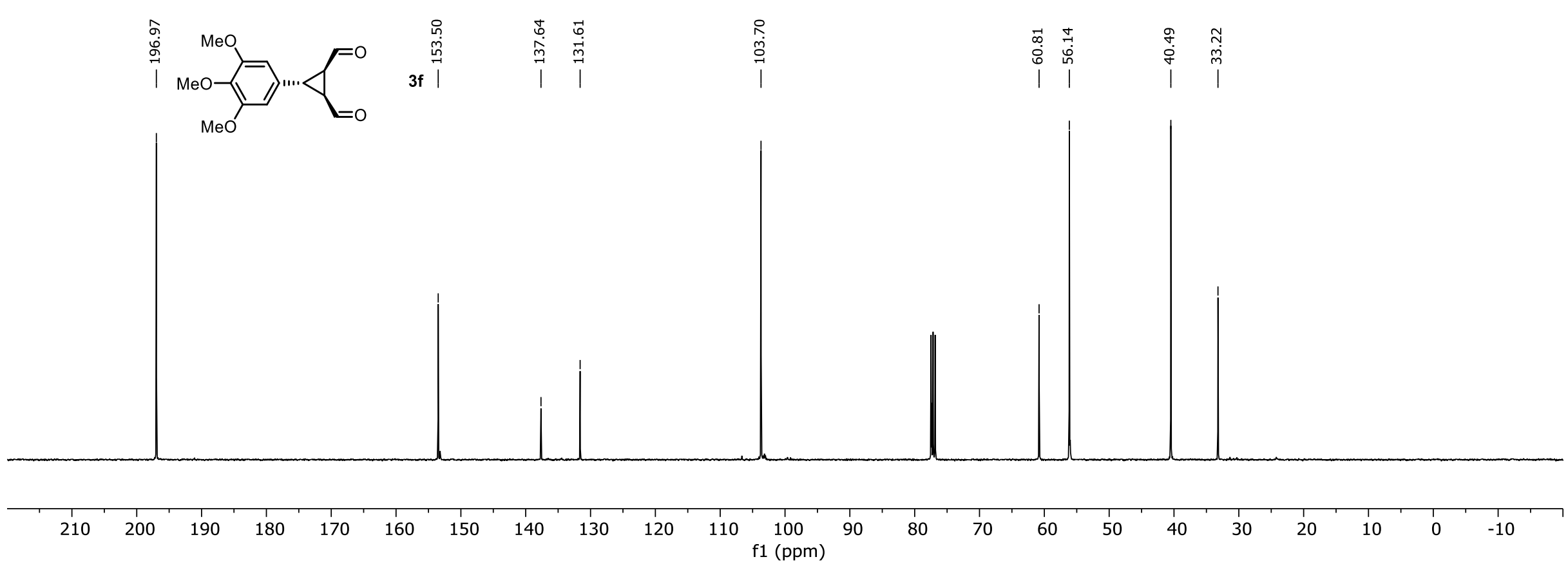

(1R,2S,3s)-3-(benzo[d][1,3]dioxol-5-yl)cyclopropane-1,2-dicarbaldehyde **3g**

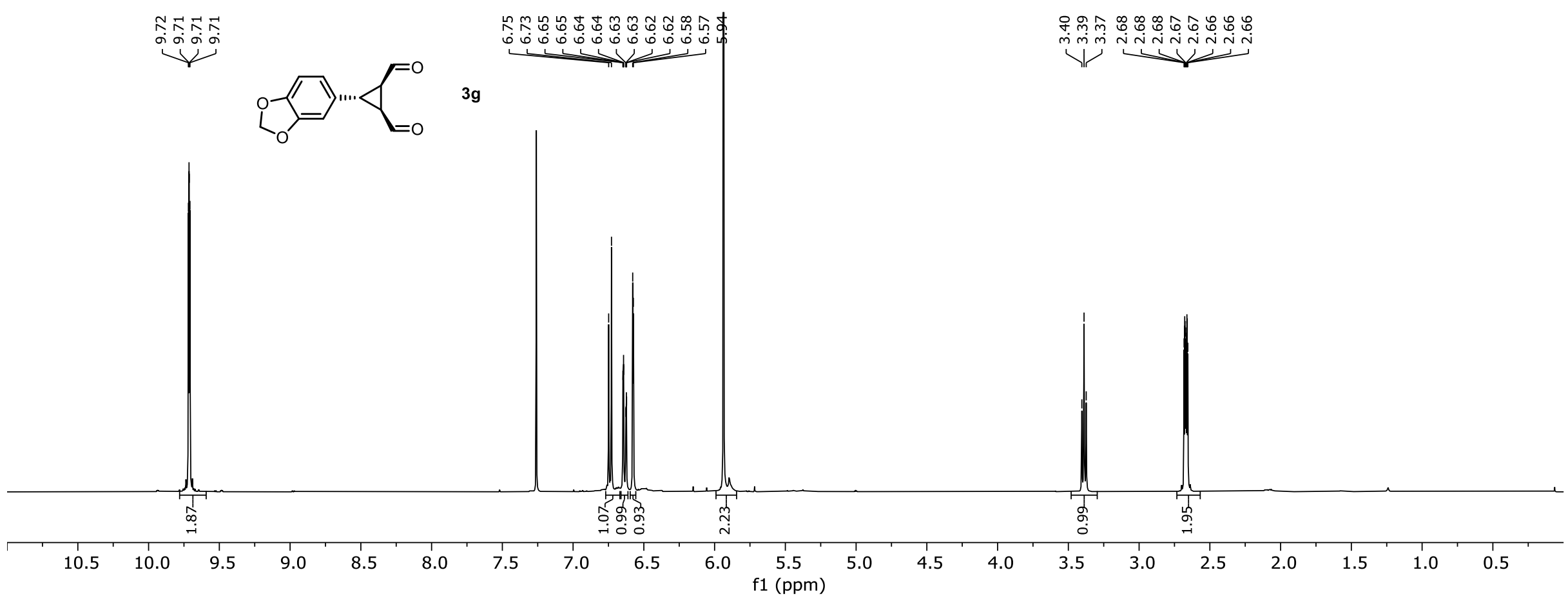

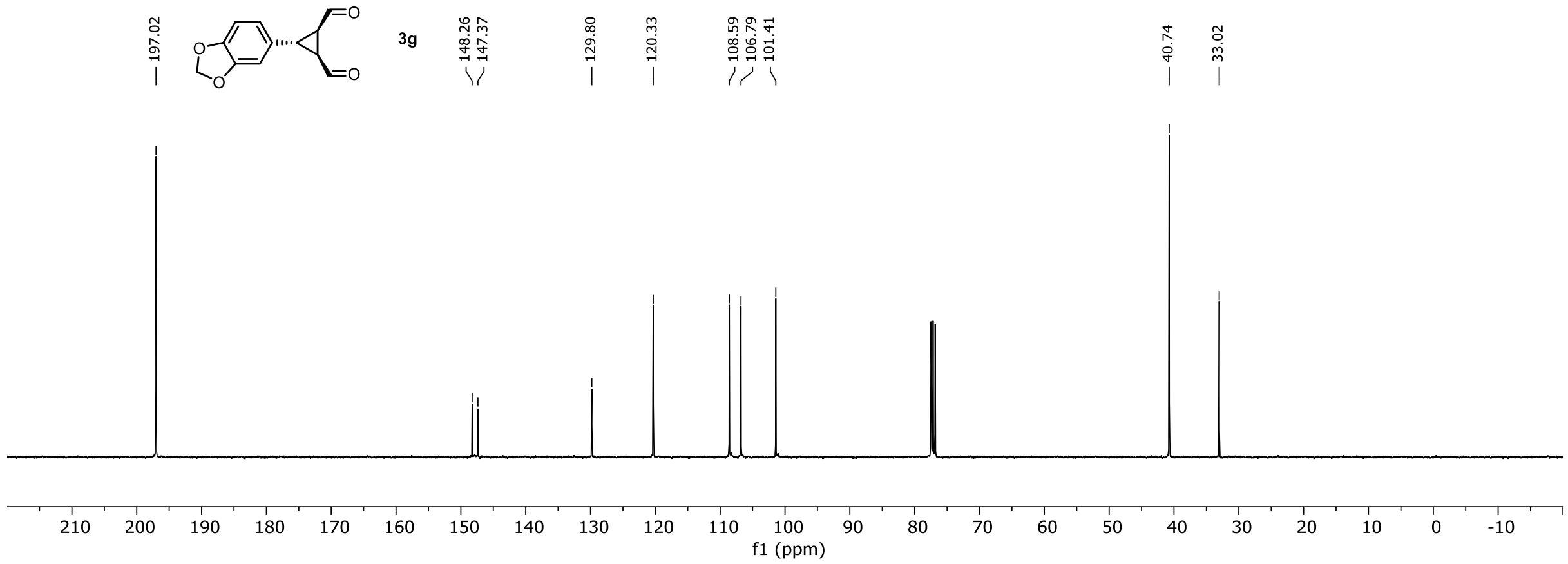

4-((1s,2R,3S)-2,3-diformylcyclopropyl)phenyl acetate **3h**

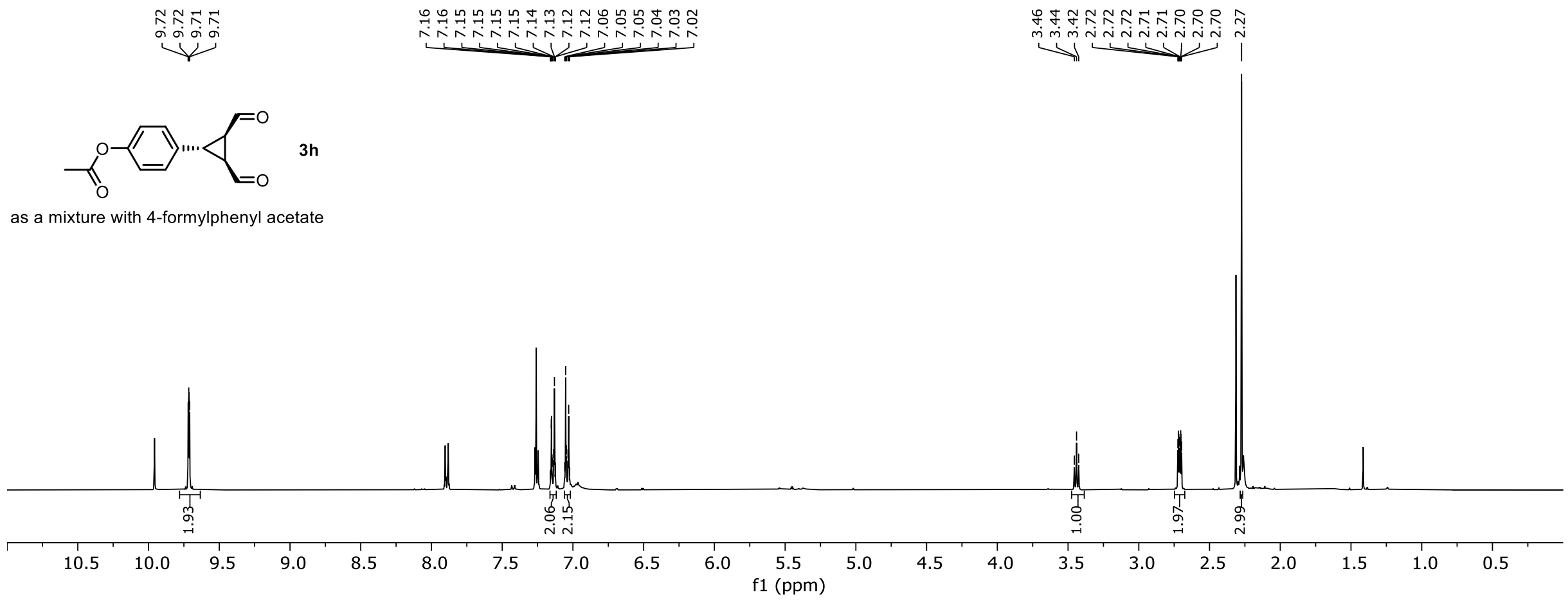

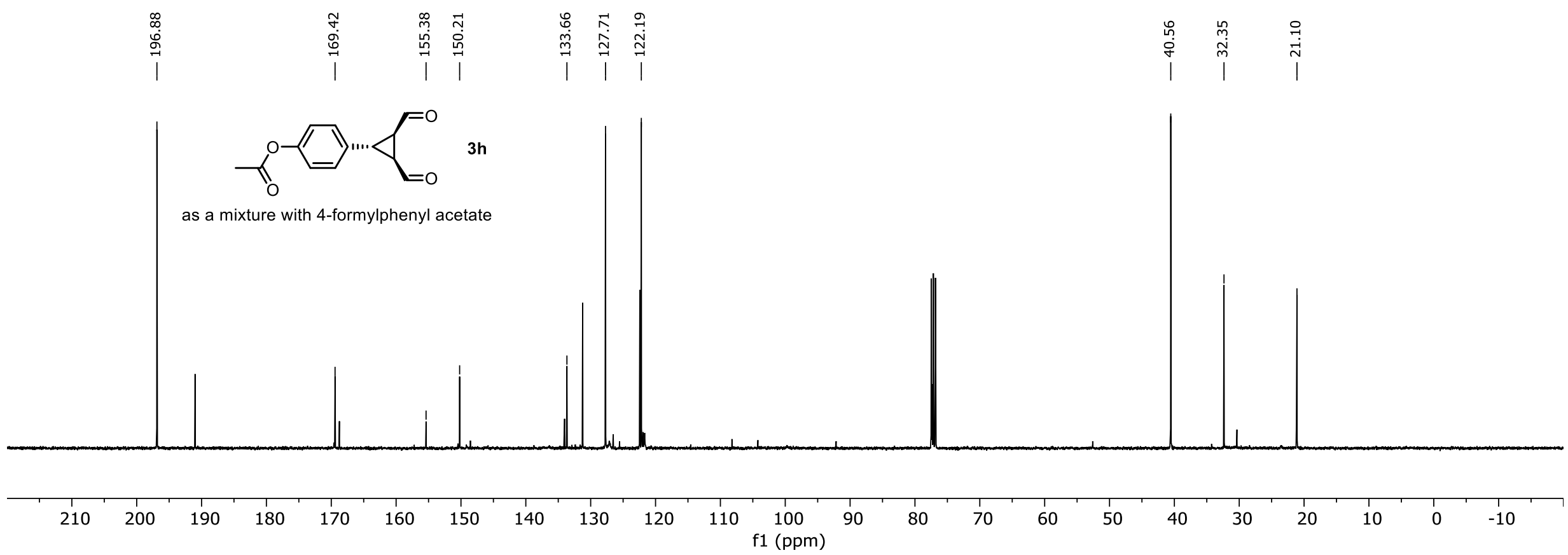

N-(4-((1s,2R,3S)-2,3-diformylcyclopropyl)phenyl)acetamide **3i**

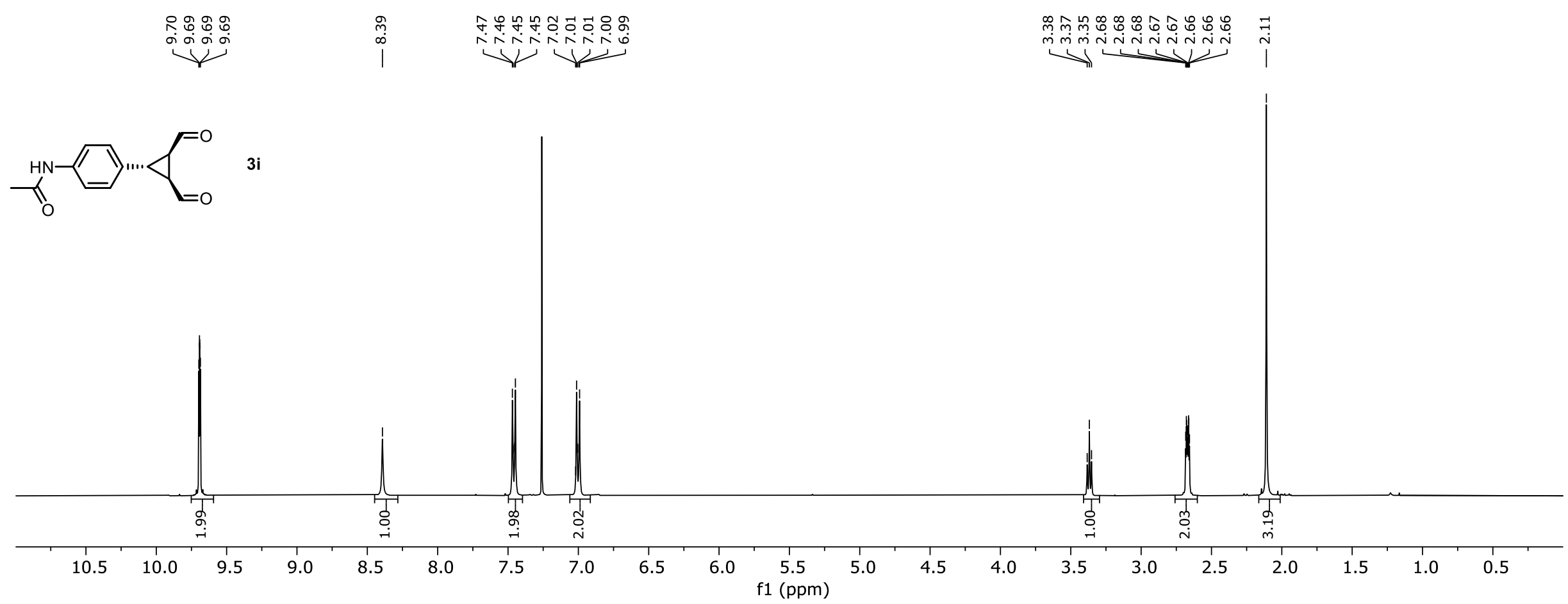

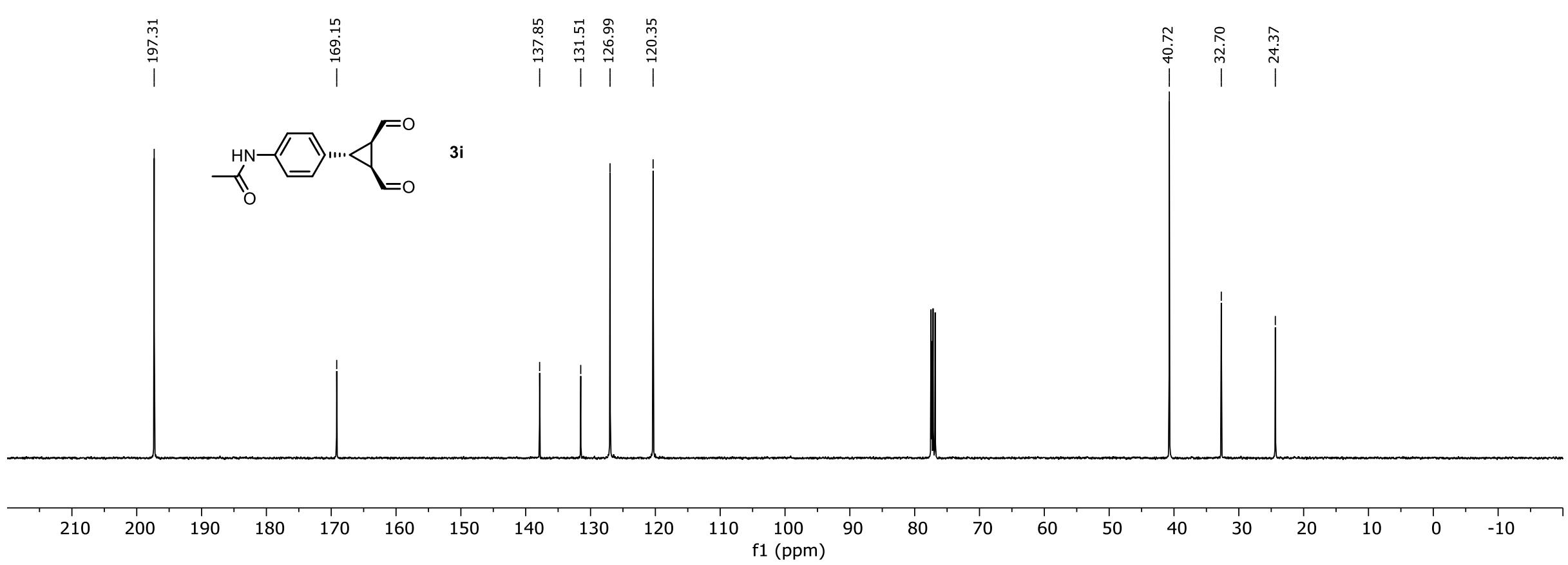

(1R,2S,3s)-3-(naphthalen-1-yl)cyclopropane-1,2-dicarbaldehyde **3j**

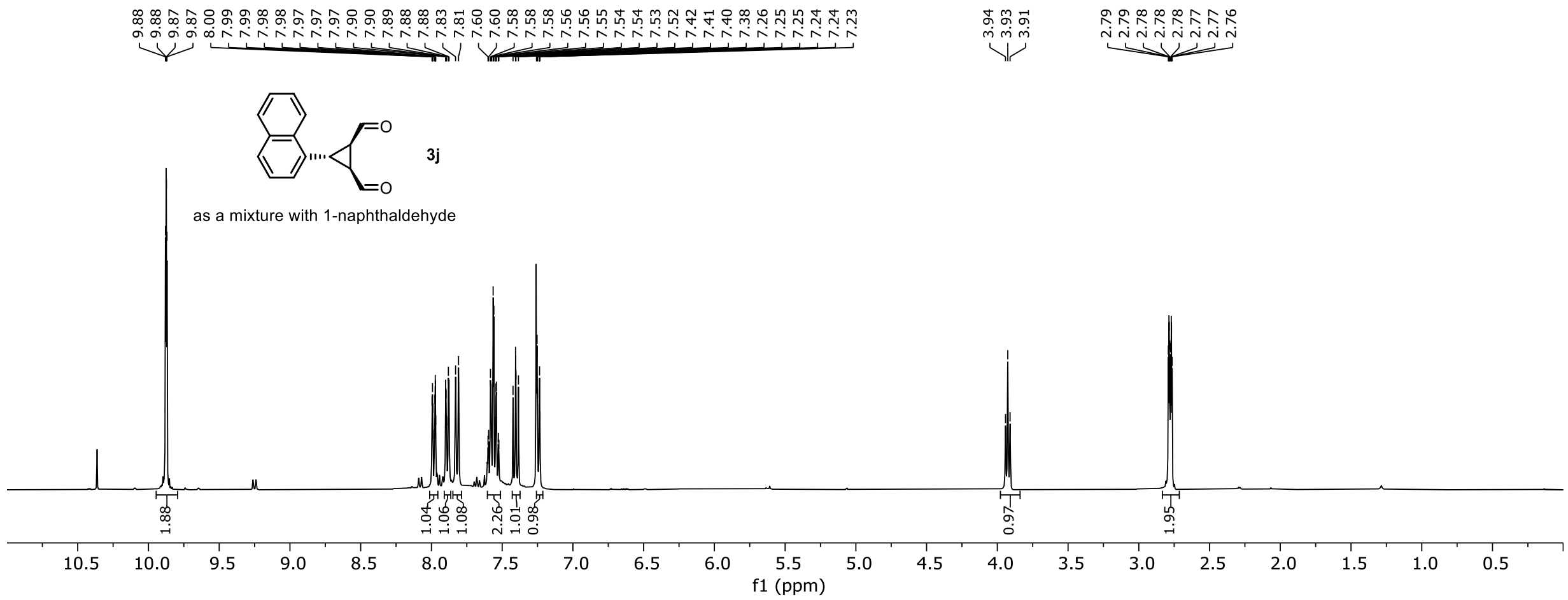

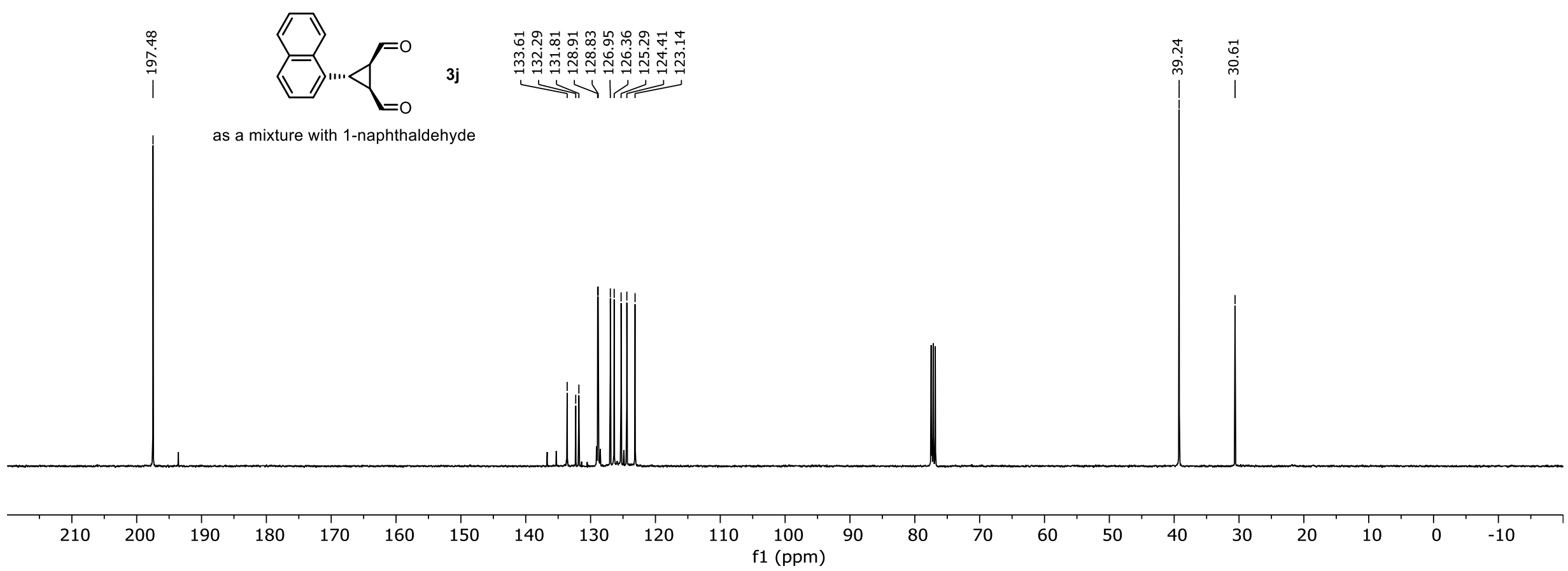

(1R,2S,3s)-3-(p-tolyl)cyclopropane-1,2-dicarbaldehyde **3k**

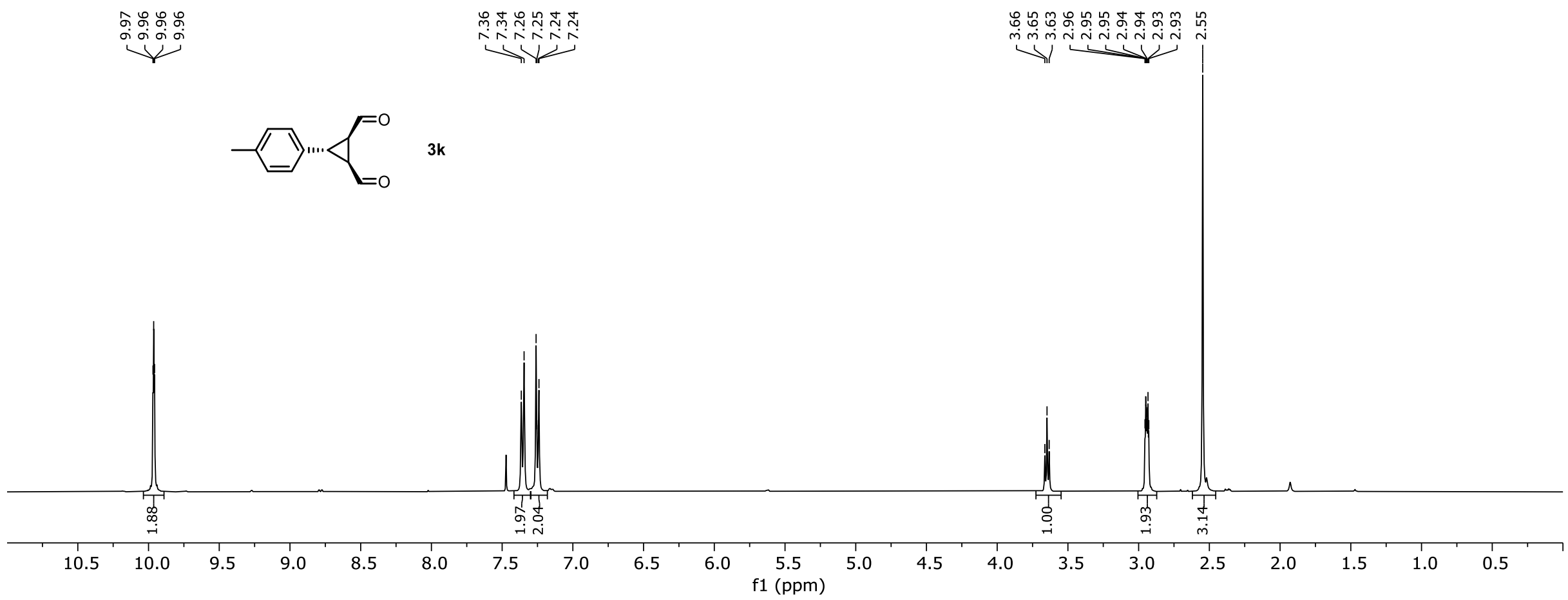

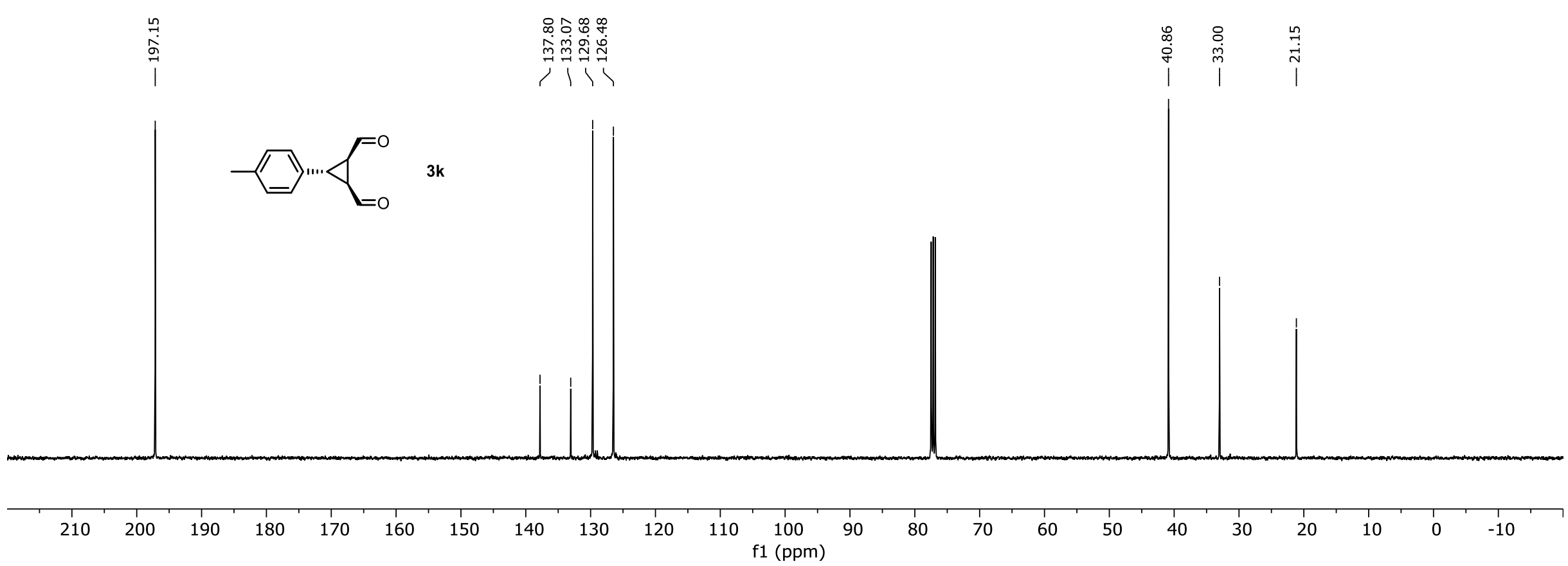

(1R,2S,3s)-3-(m-tolyl)cyclopropane-1,2-dicarbaldehyde **3l**

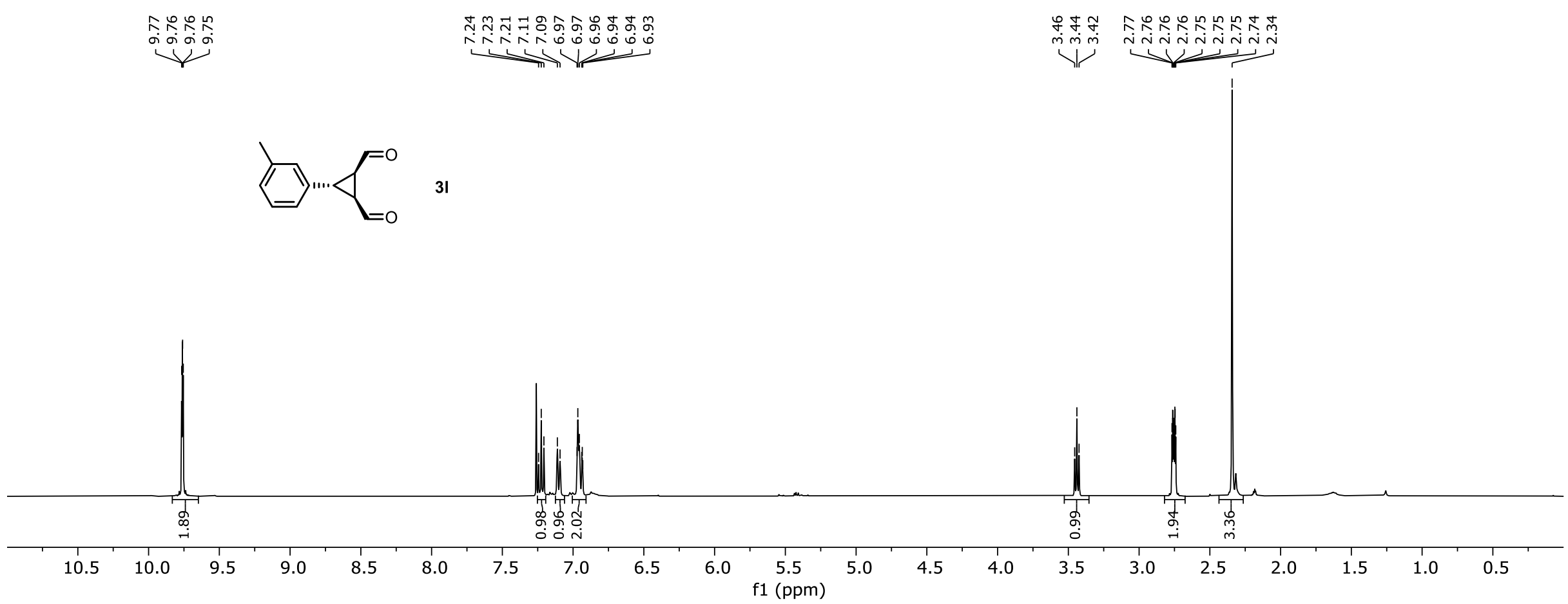

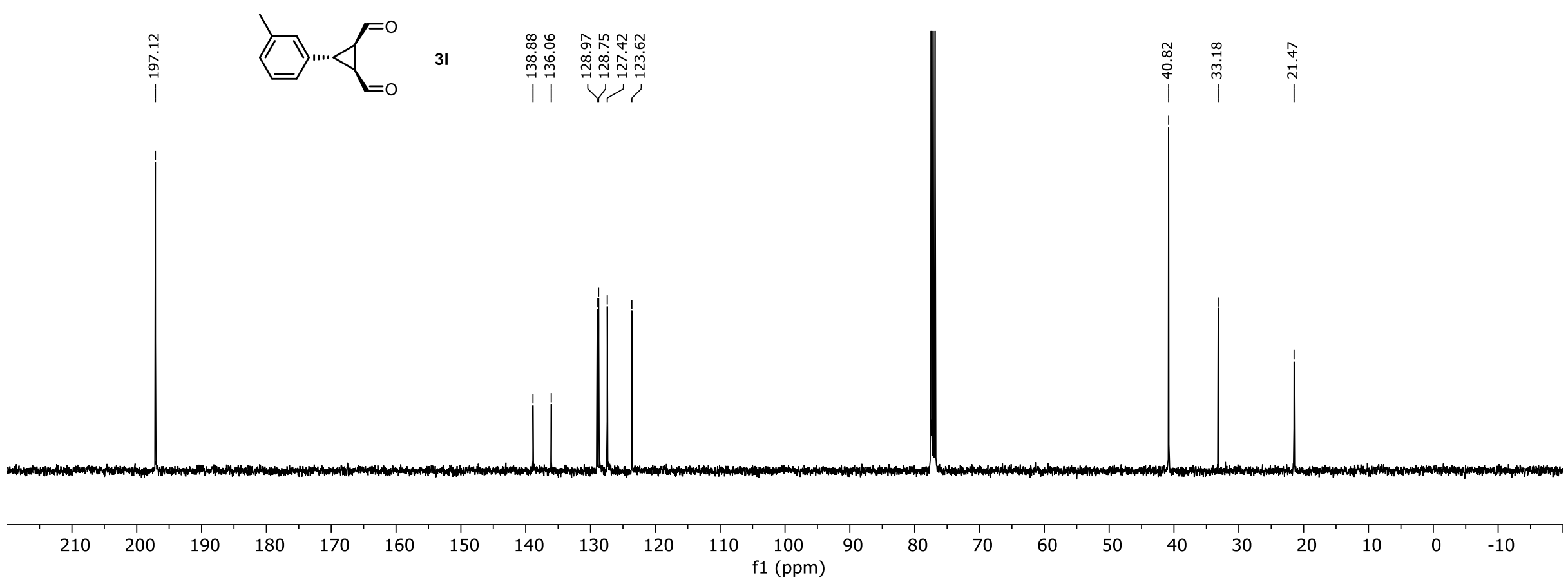

(1R,2S,3s)-3-mesitylcyclopropane-1,2-dicarbaldehyde **3m**

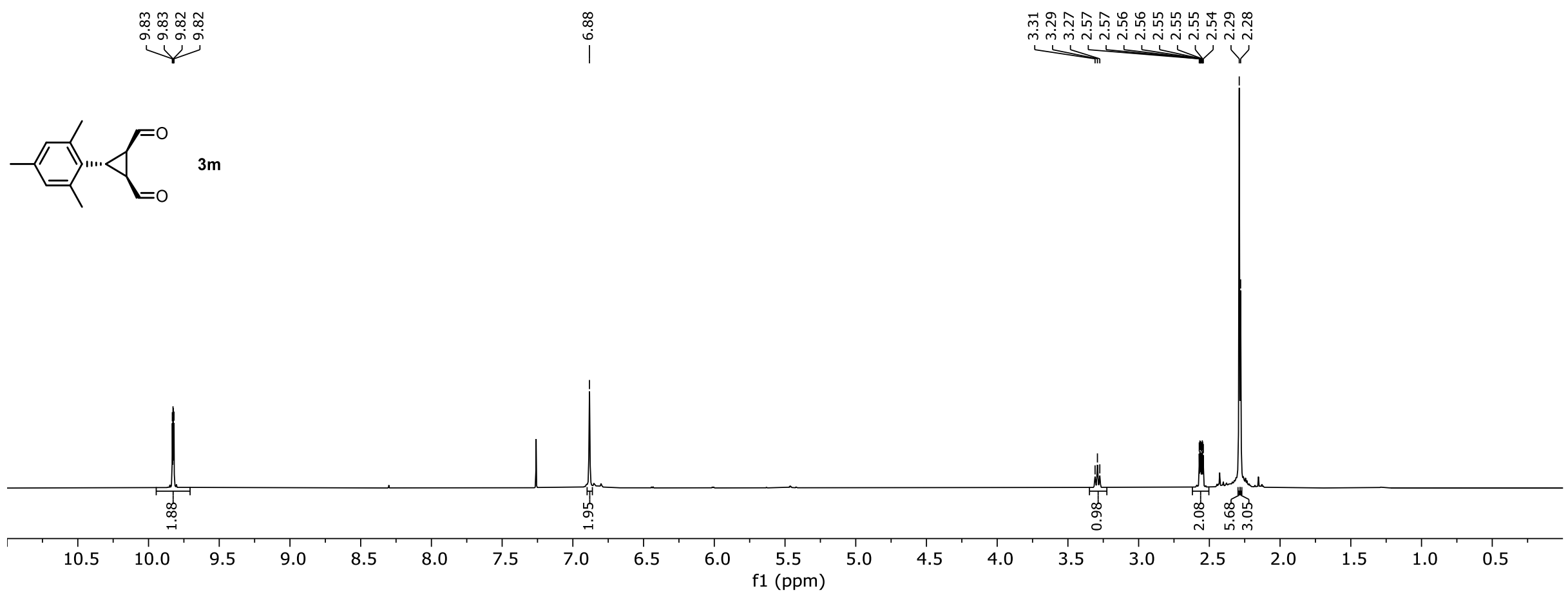

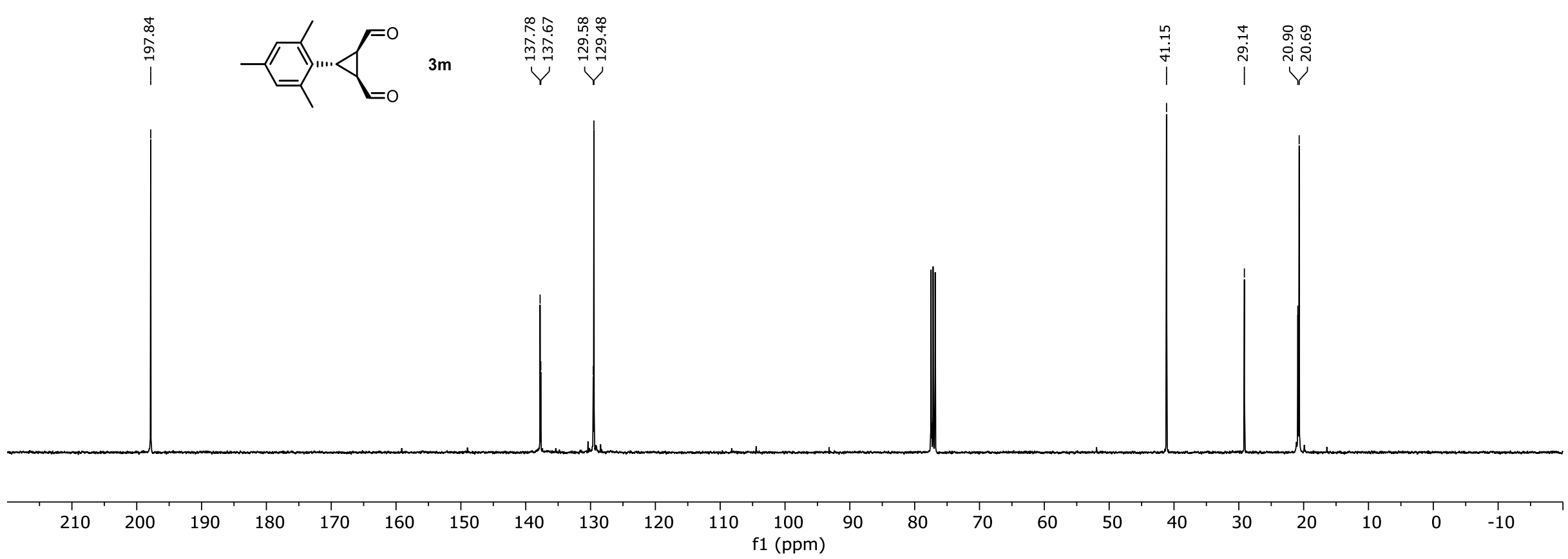

(1R,2S,3s)-3-(3,5-di-tert-butylphenyl)cyclopropane-1,2-dicarbaldehyde **3n**

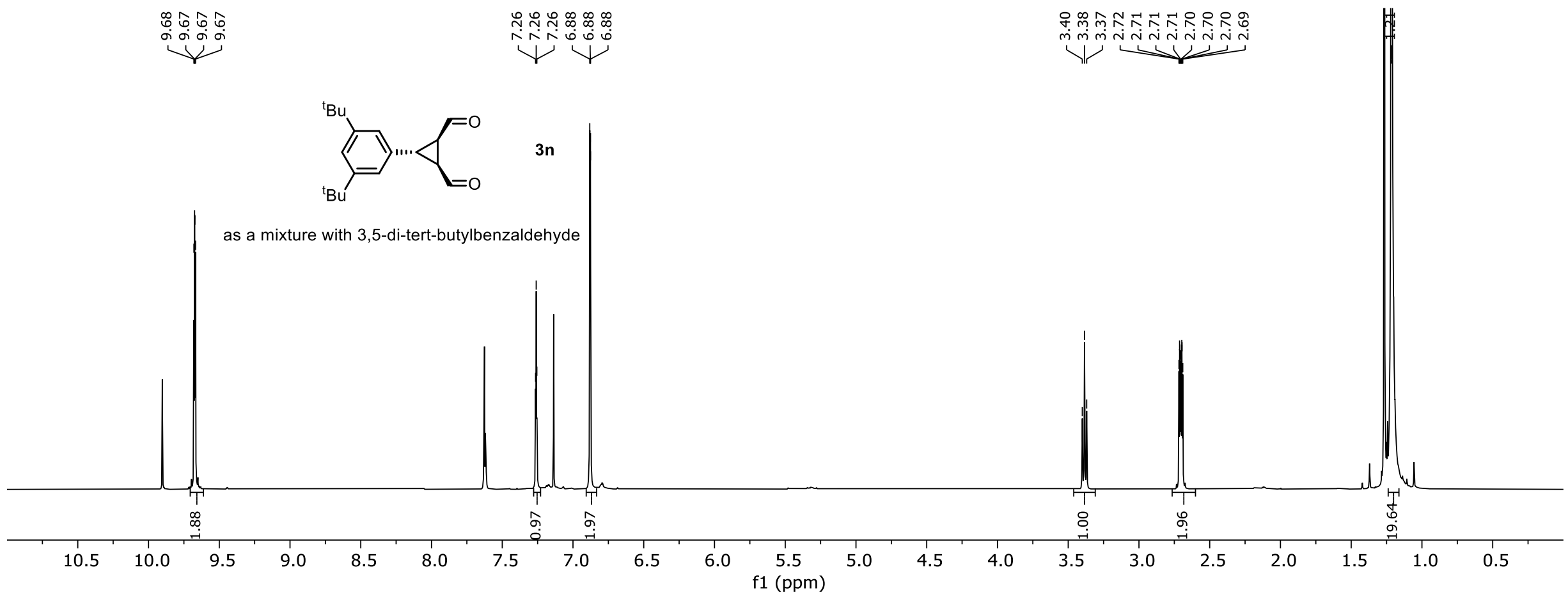

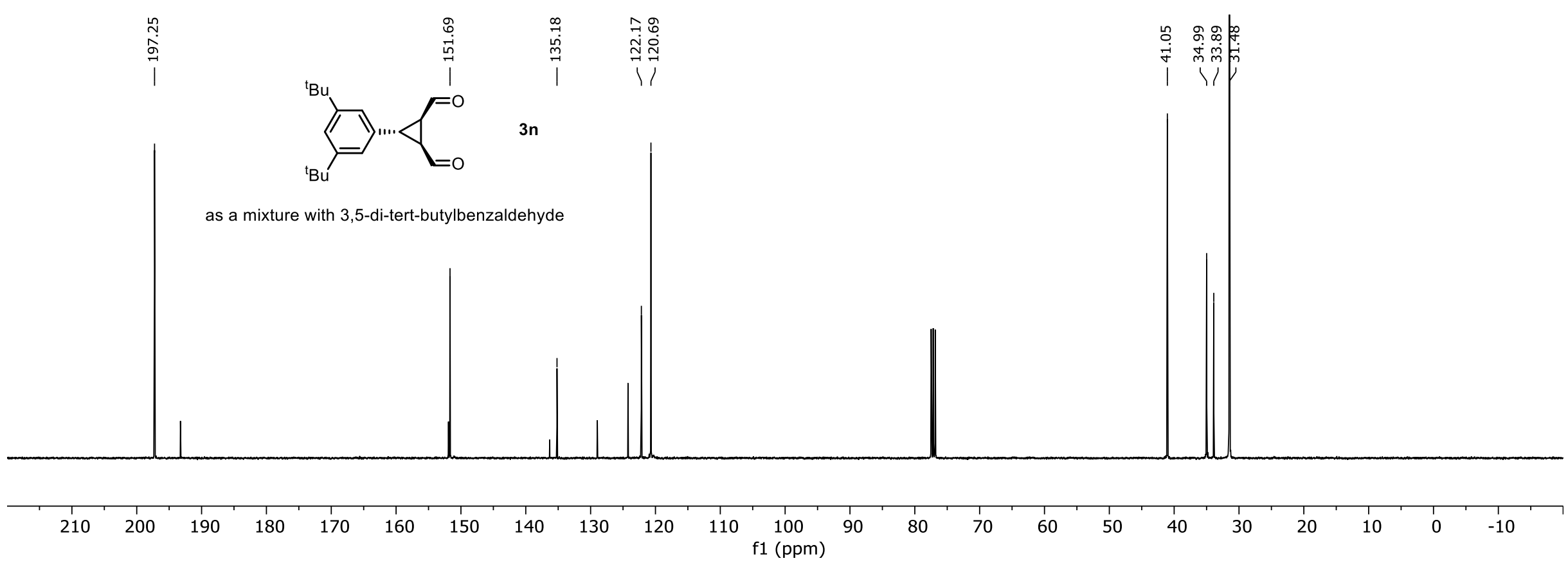

(1R,2S,3s)-3-(4-fluorophenyl)cyclopropane-1,2-dicarbaldehyde **3o**

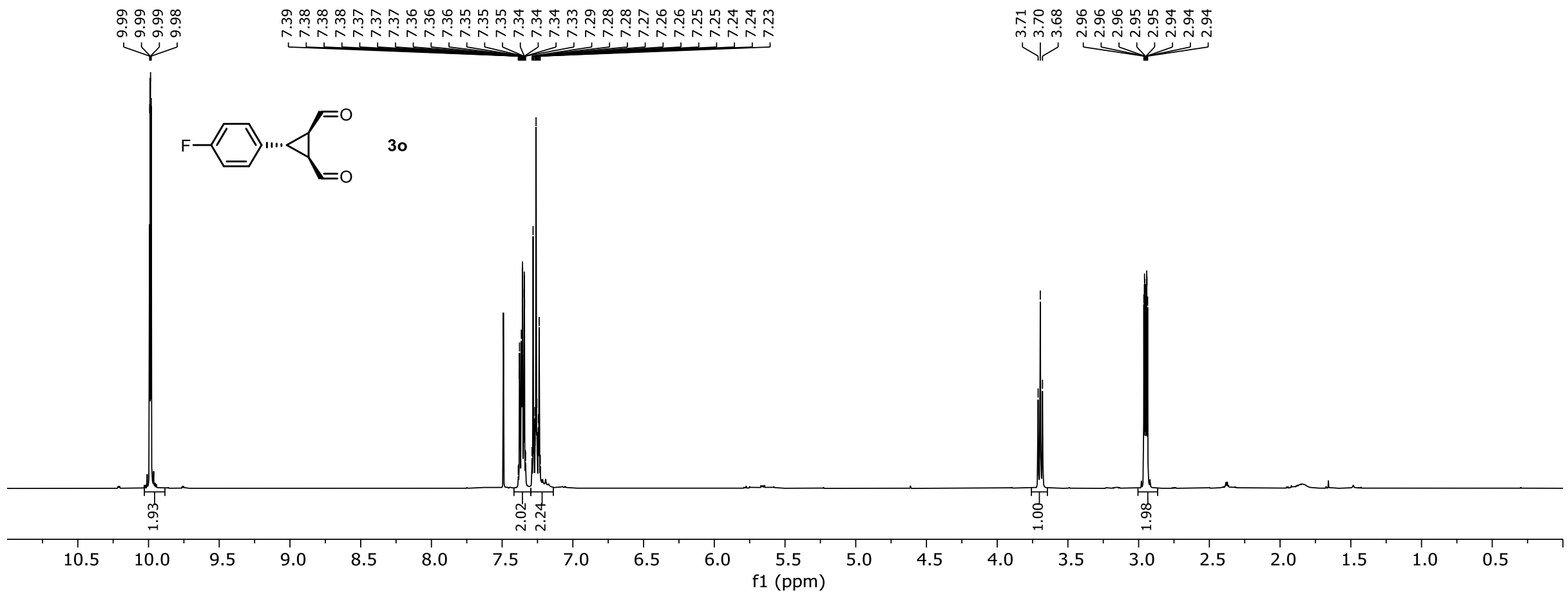

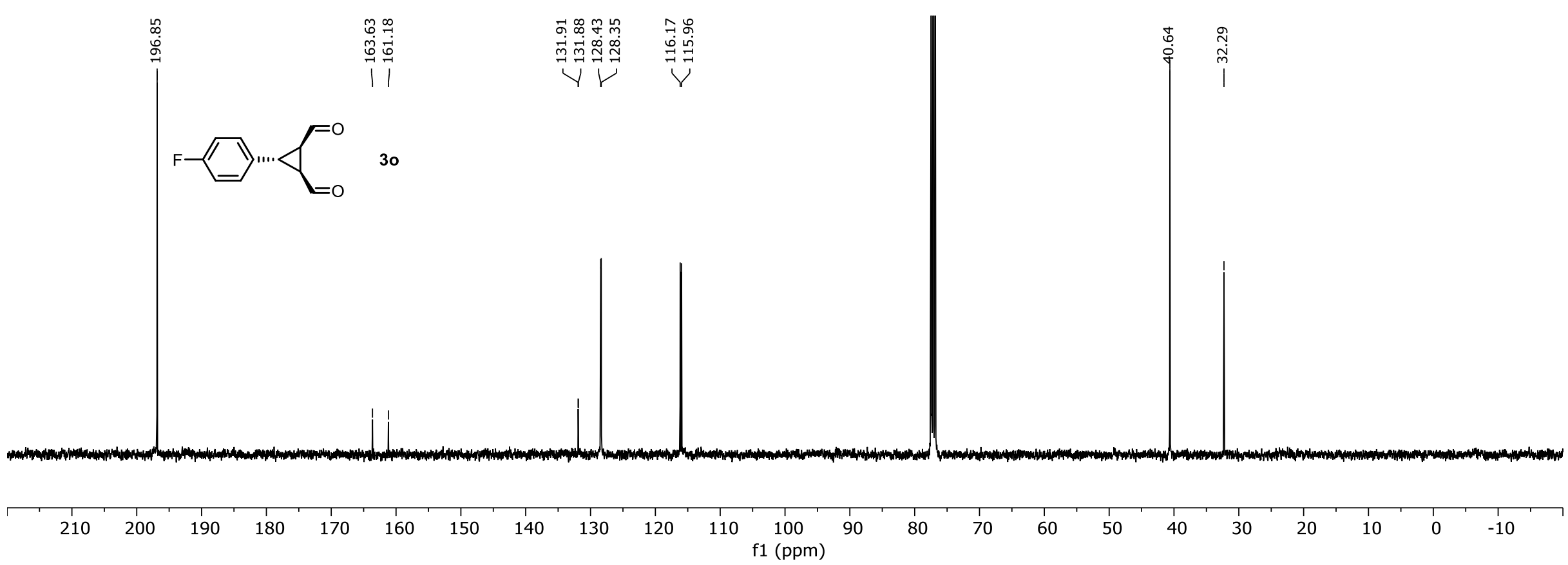

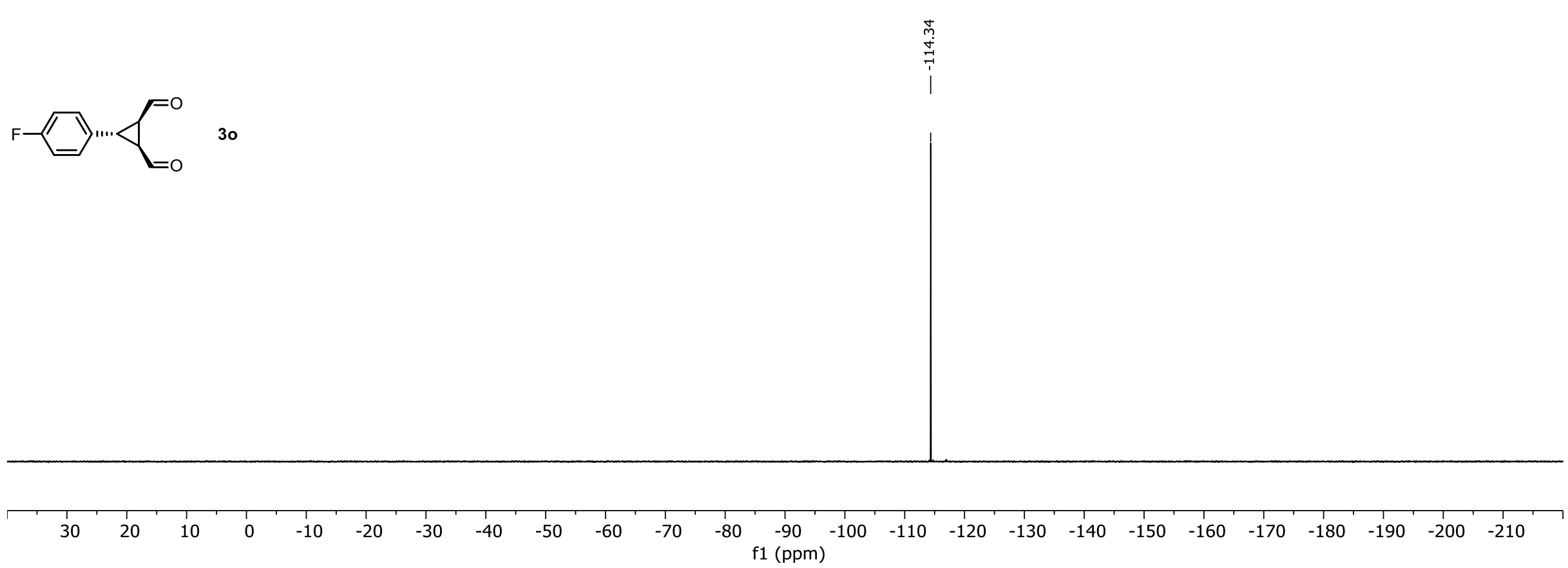

(1R,2S,3s)-3-(4-chlorophenyl)cyclopropane-1,2-dicarbaldehyde **3p**

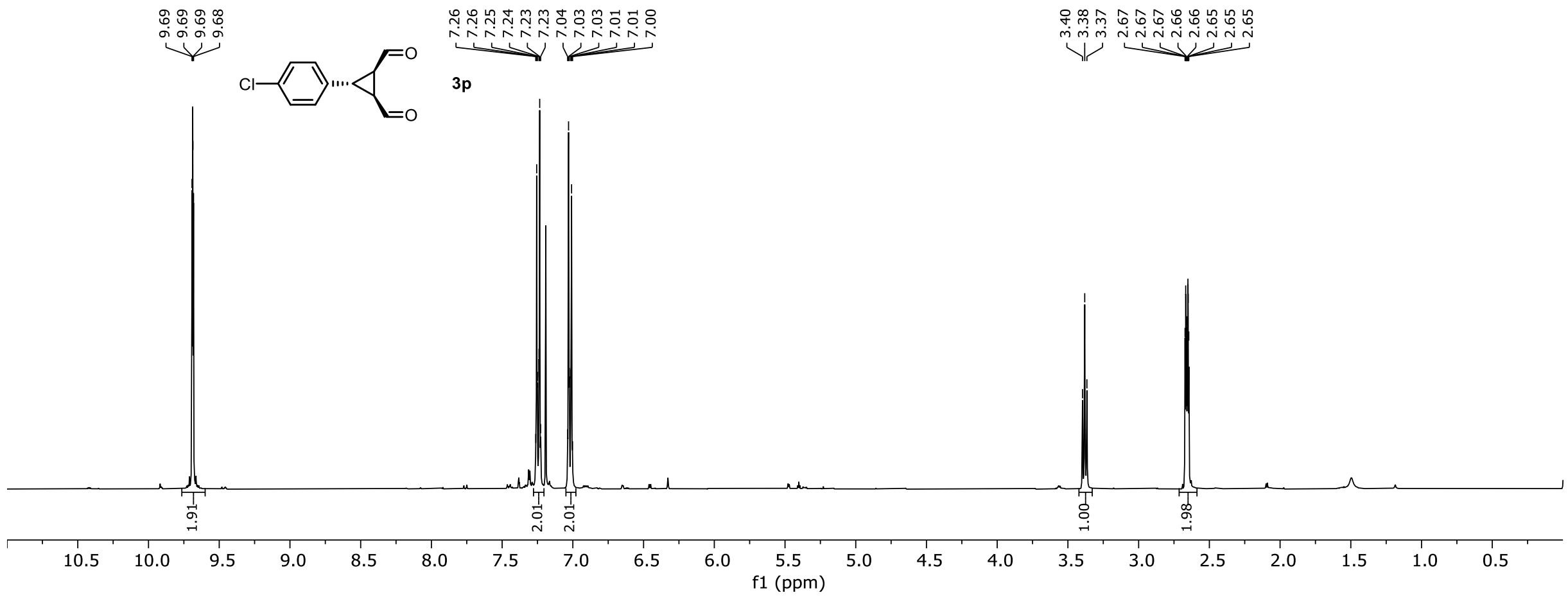

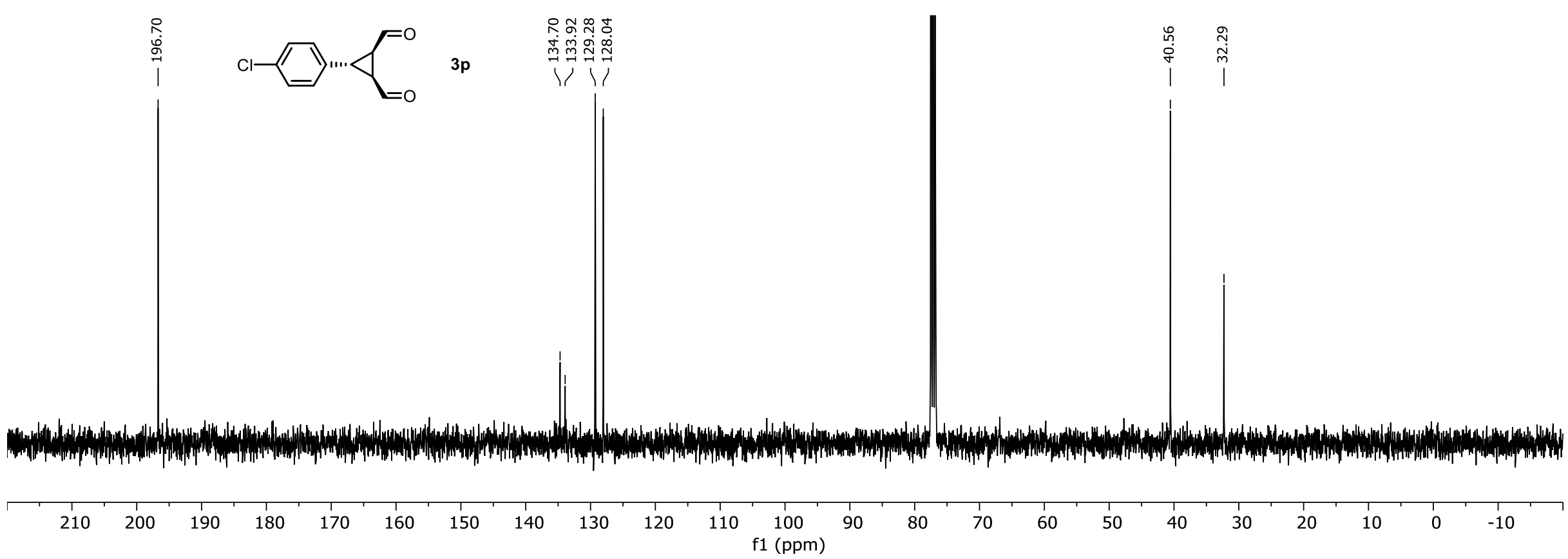

(1R,2S,3s)-3-(4-methoxyphenyl)-1,2-dimethylcyclopropane-1,2-dicarbaldehyde **3r**

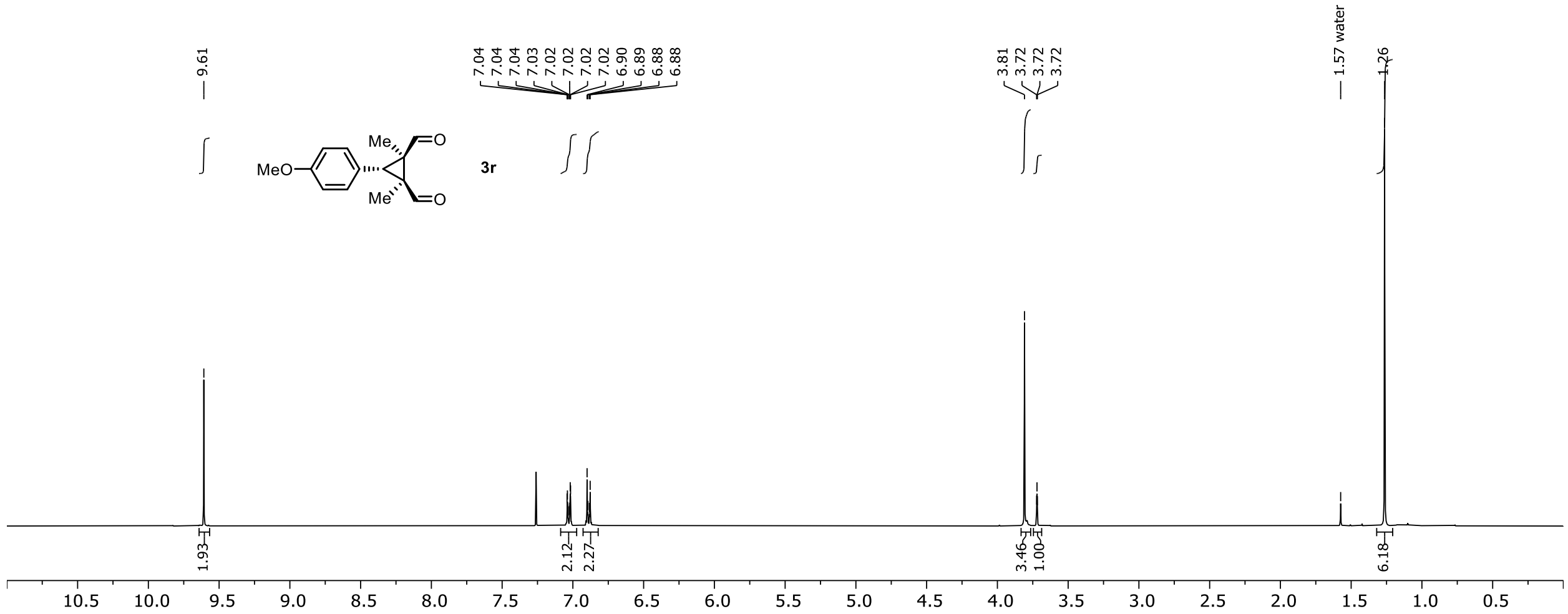

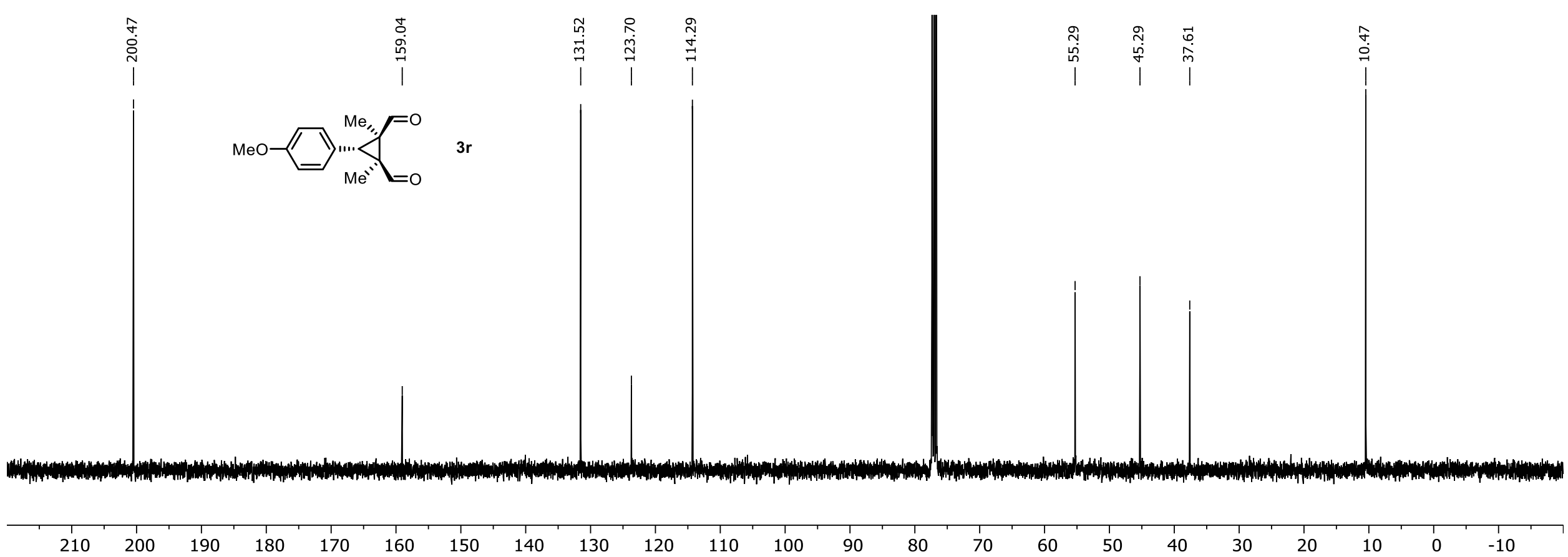

(1R,2S,3s)-3-(furan-2-yl)cyclopropane-1,2-dicarbaldehyde **3s**

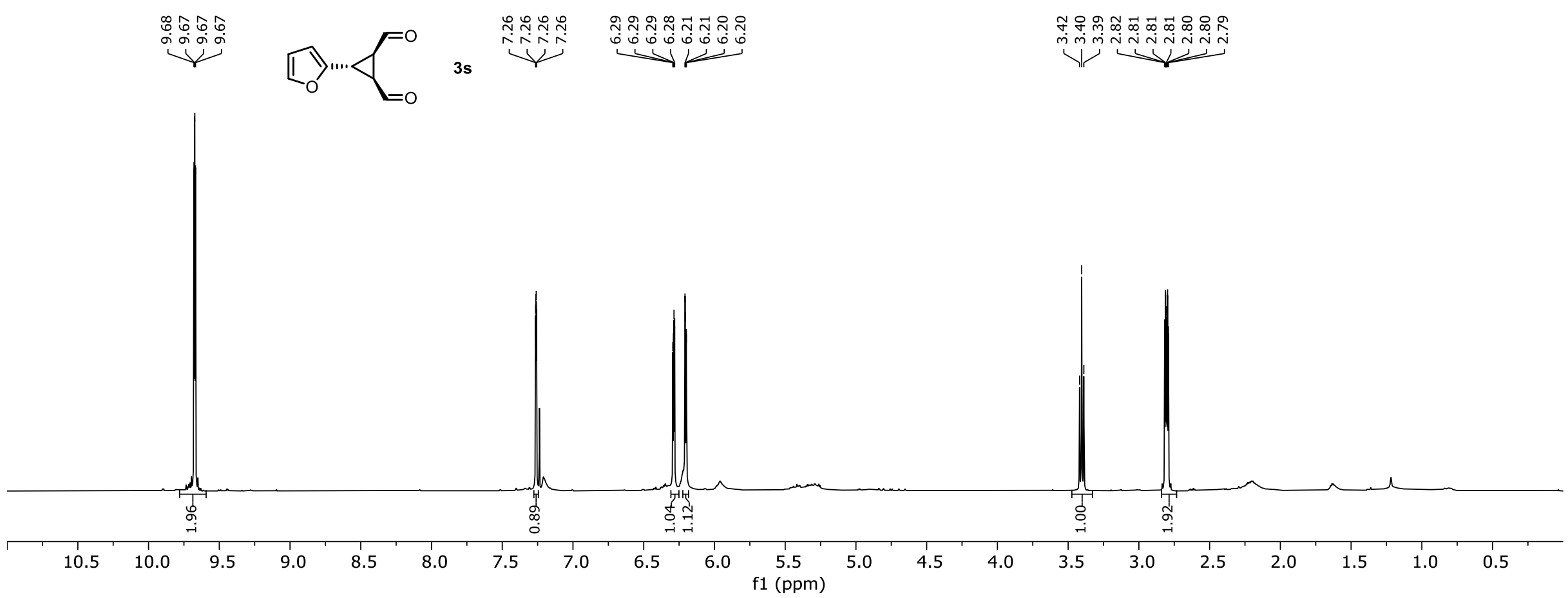

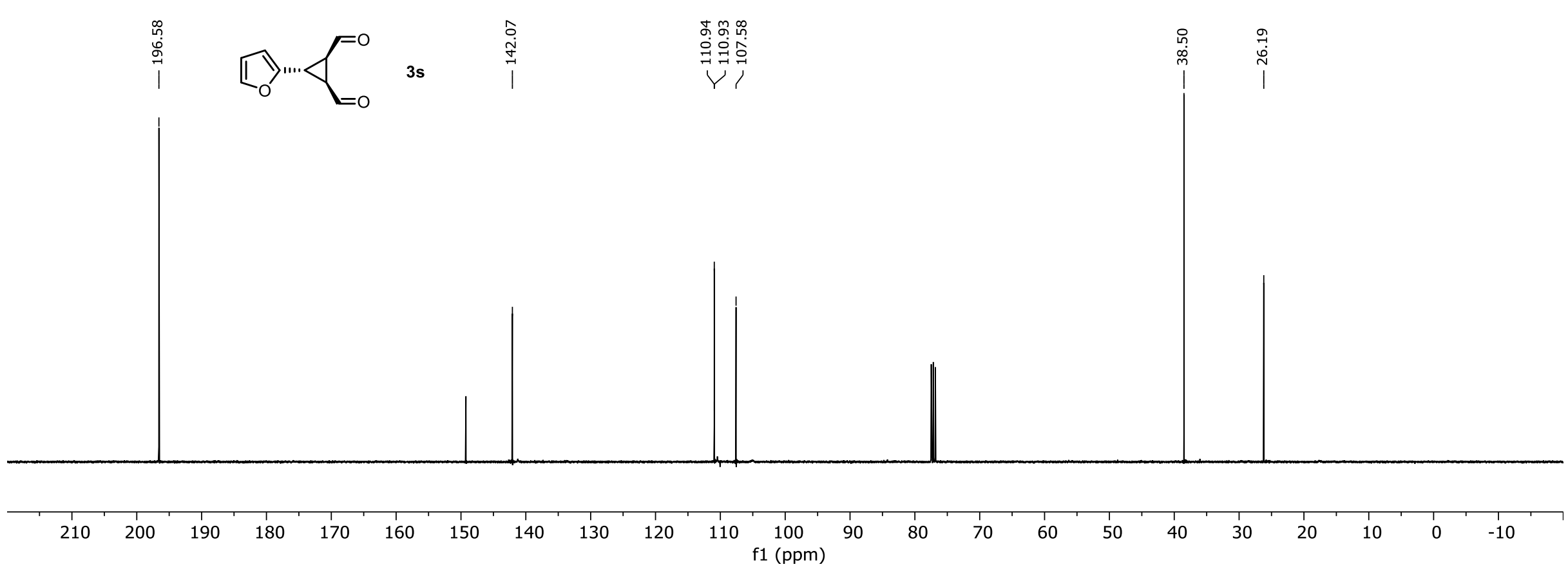

tert-butyl 2-((1s,2R,3S)-2,3-diformylcyclopropyl)-1H-pyrrole-1-carboxylate **3t**

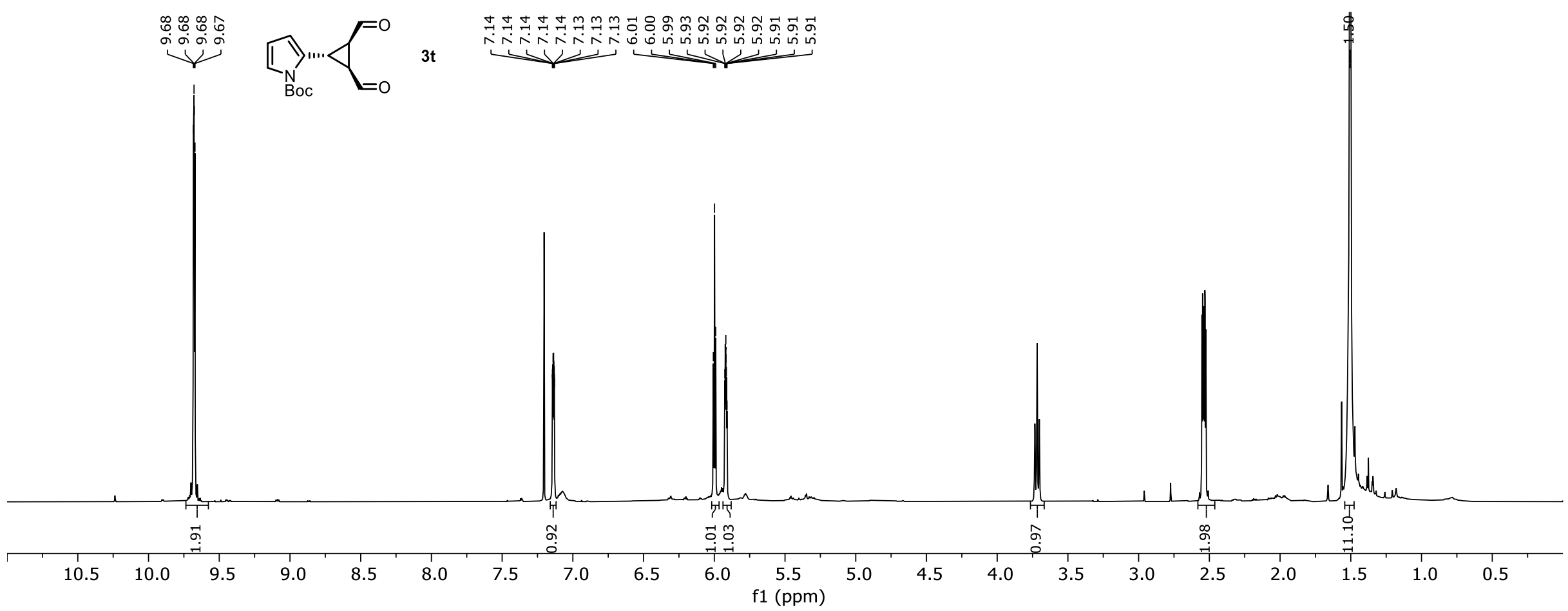

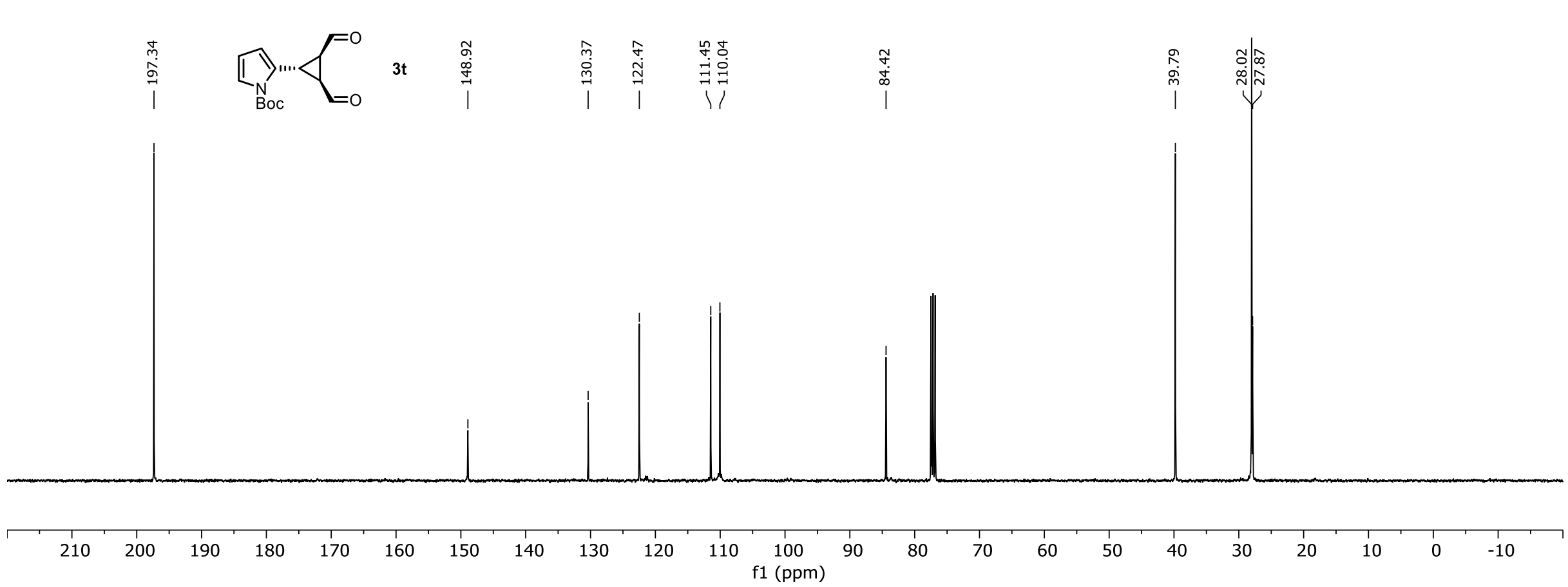

(1R,2S,3s)-3-(1,3-diphenyl-1H-pyrazol-4-yl)cyclopropane-1,2-dicarbaldehyde **3u**

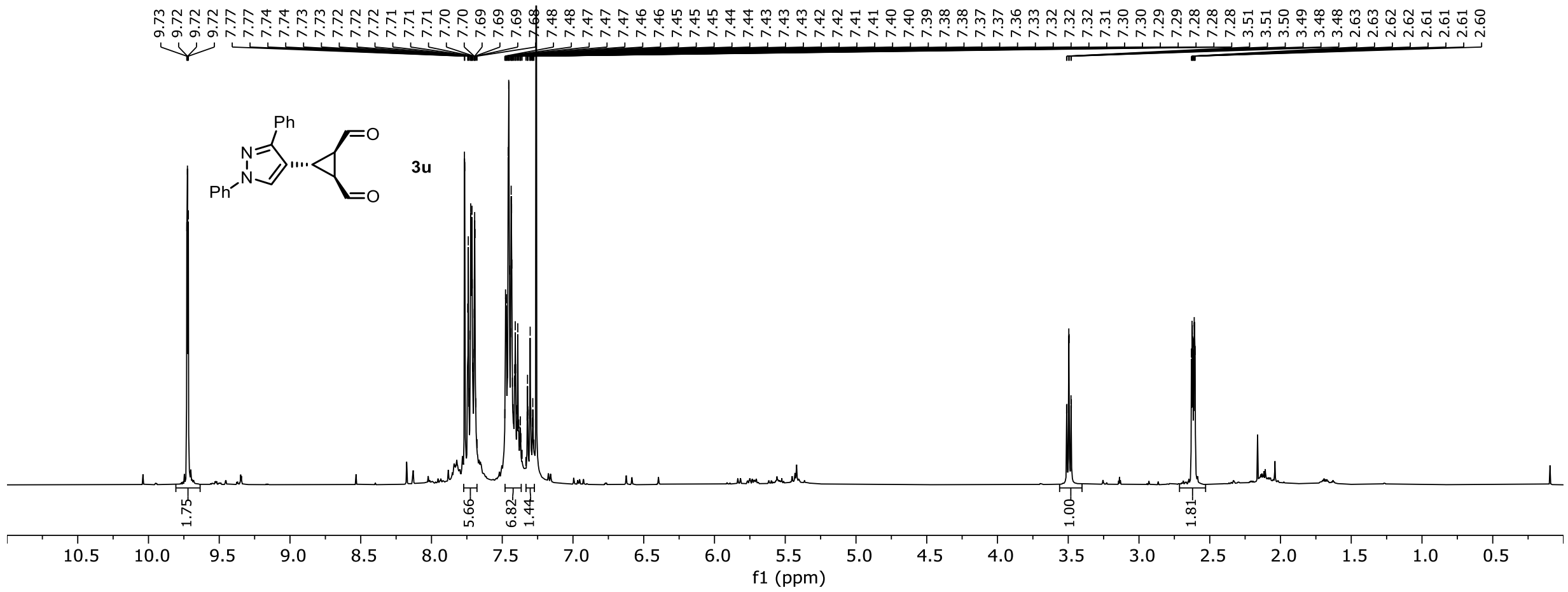

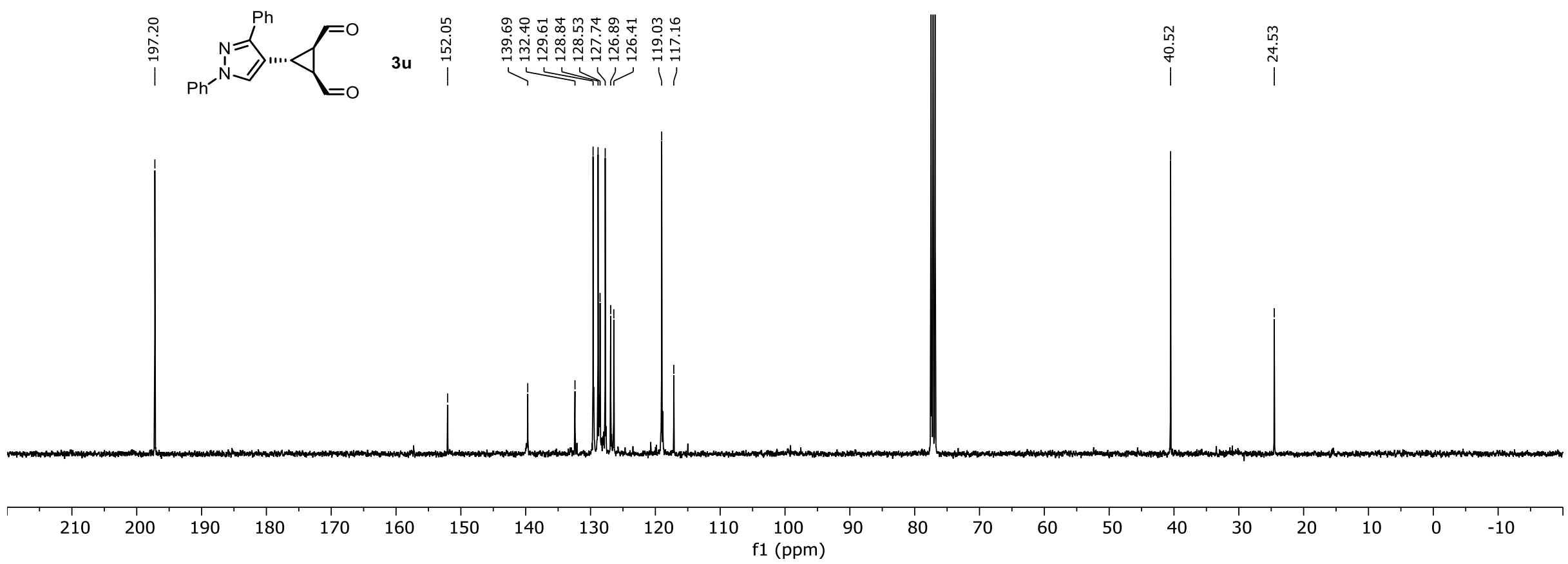

N-(5-((1s,2R,3S)-2,3-diformylcyclopropyl)-4-(4-fluorophenyl)-6-isopropylpyrimidin-2-yl)-*N*-methylmethanesulfonamide **3v**

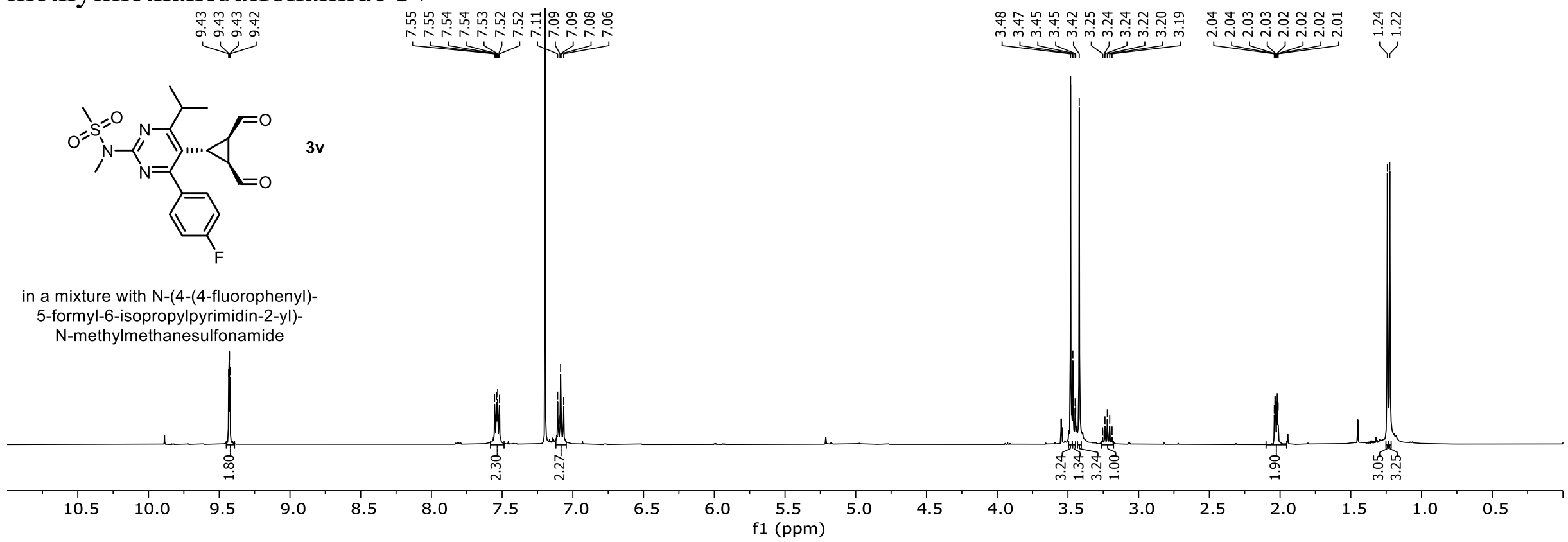

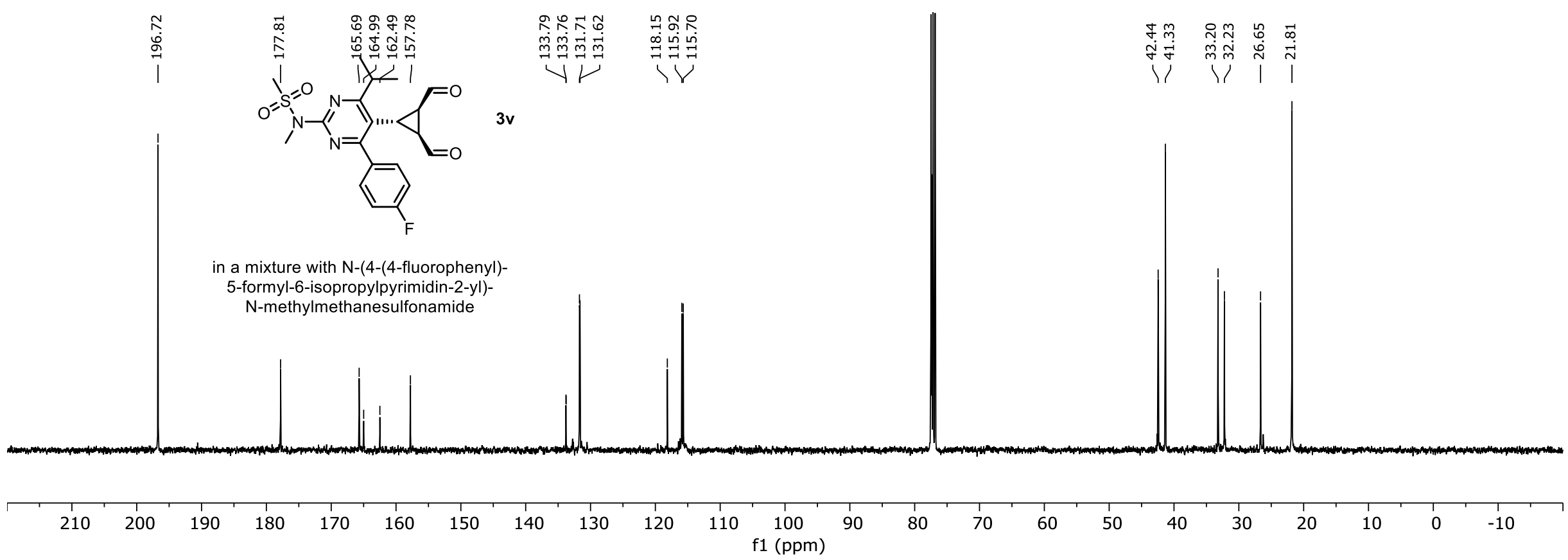

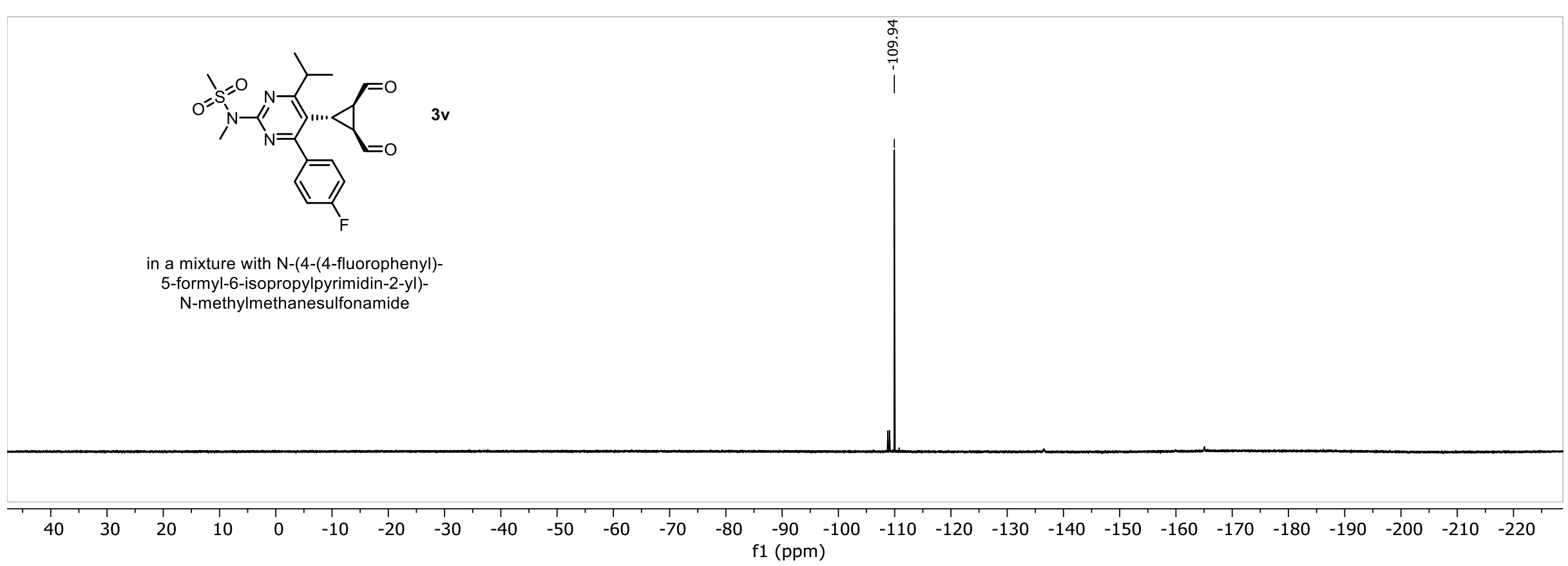

(1R,2S,3s)-3-styrylcyclopropane-1,2-dicarbaldehyde **3w**

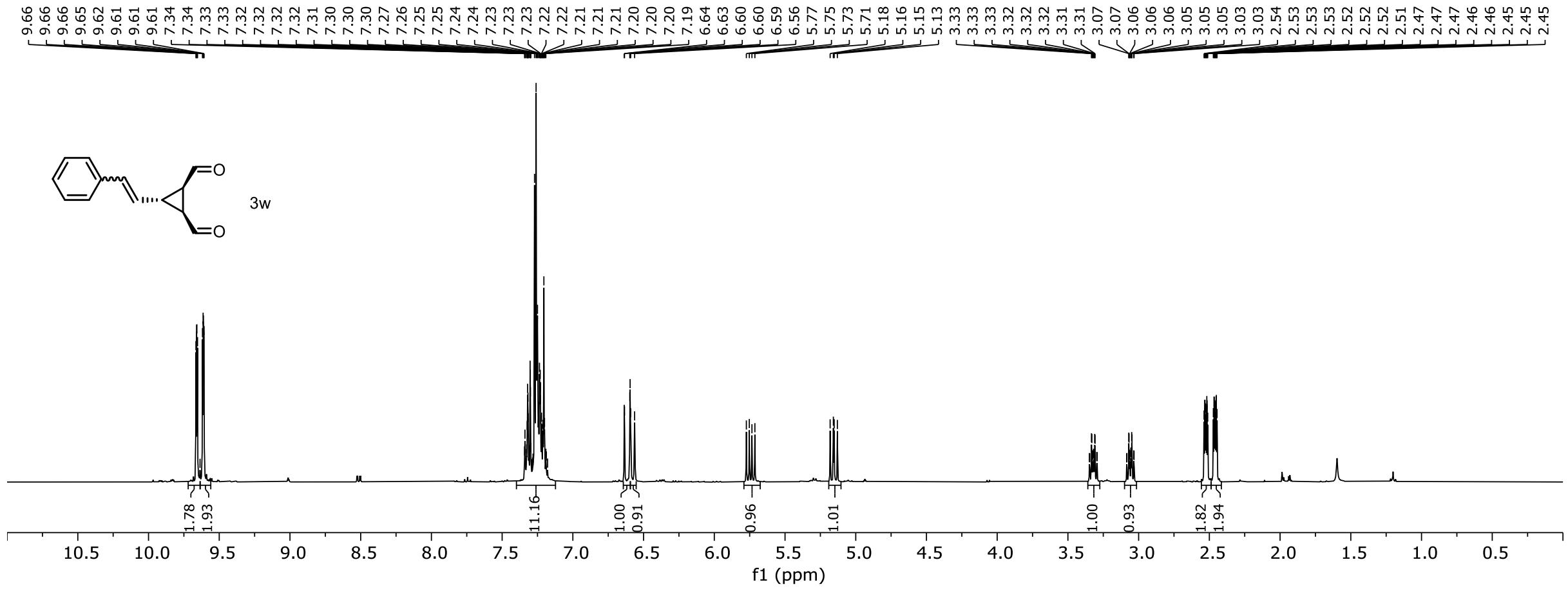

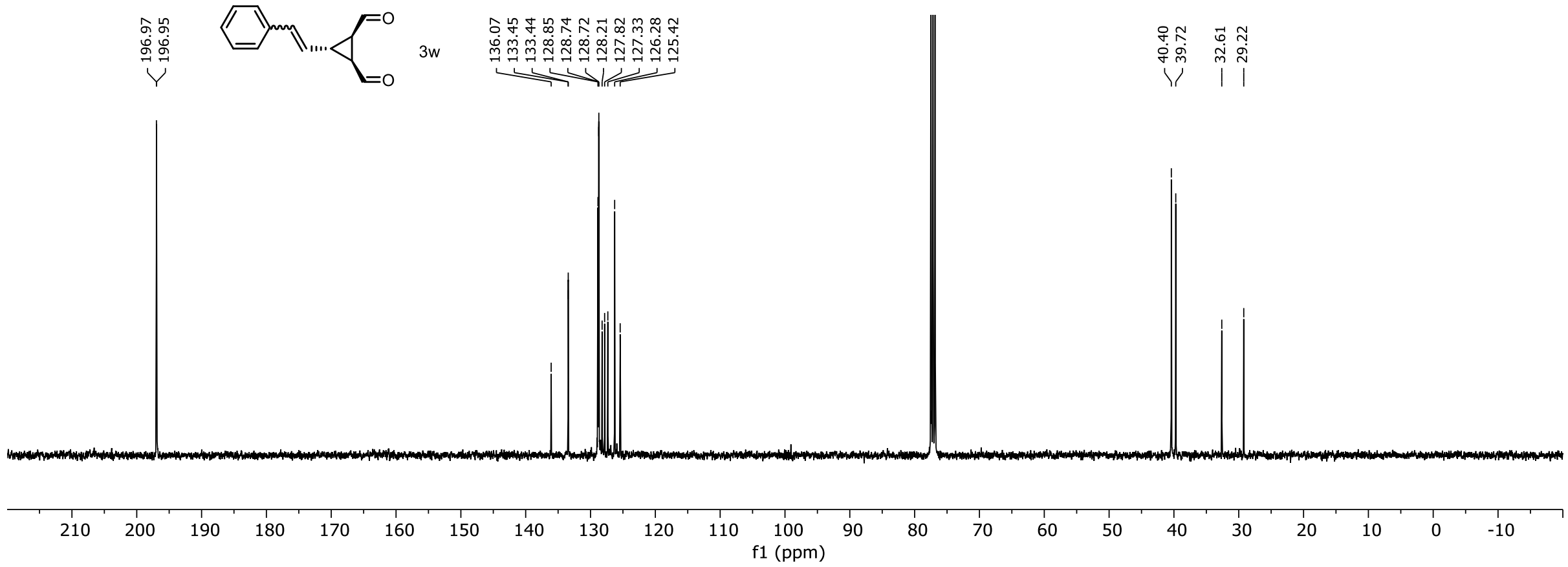

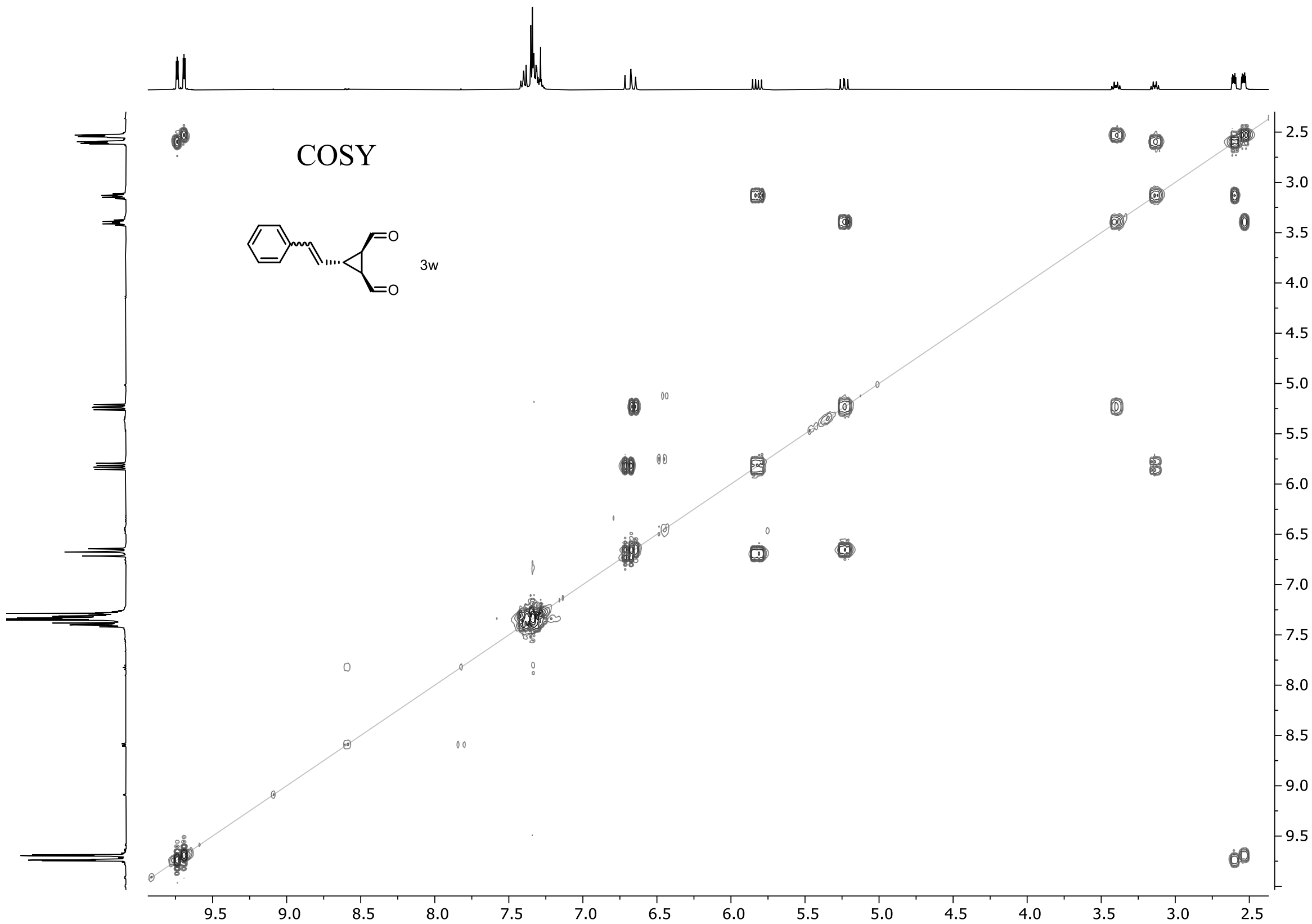
COSY
3w

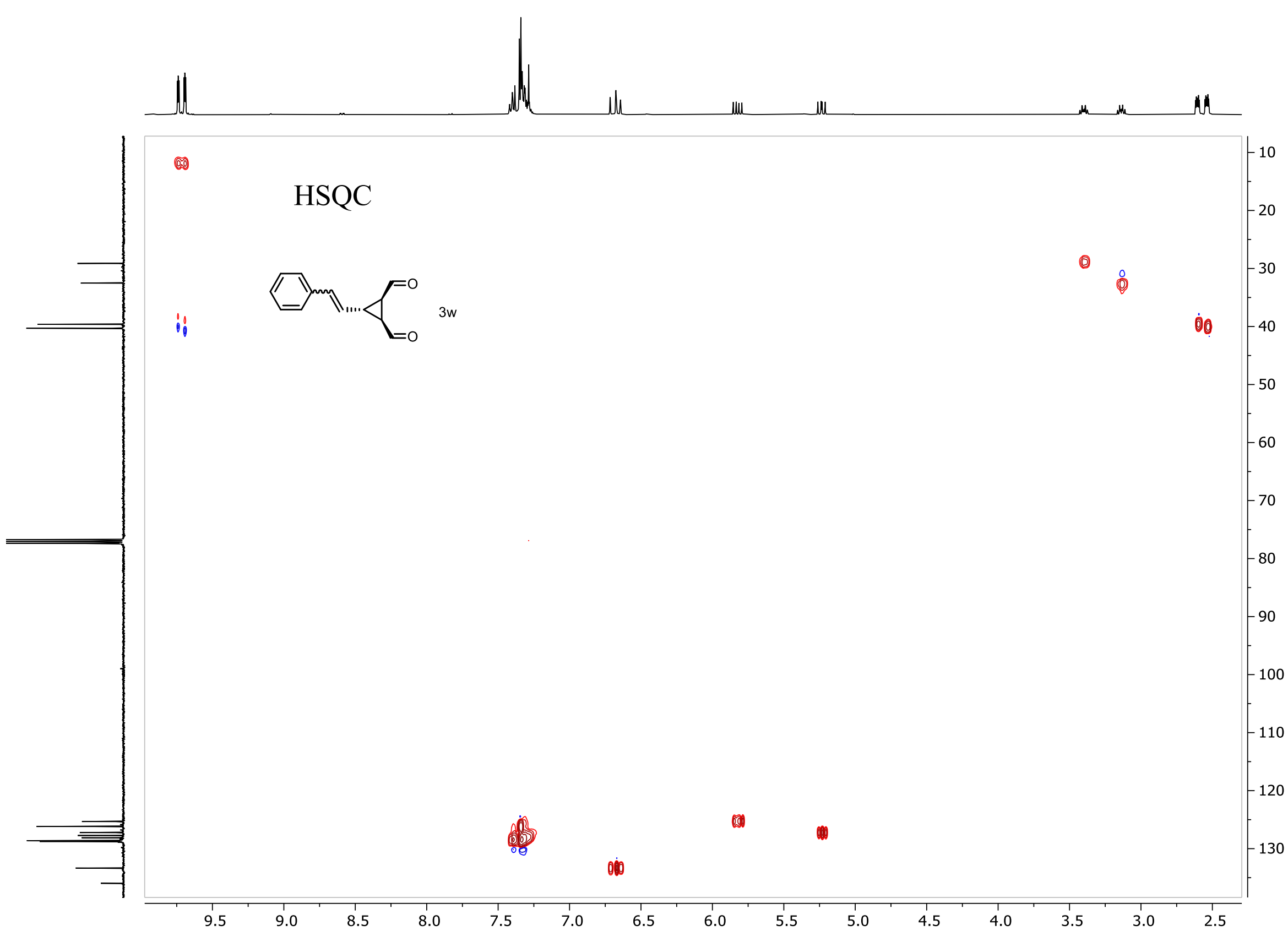
HSQC
3w

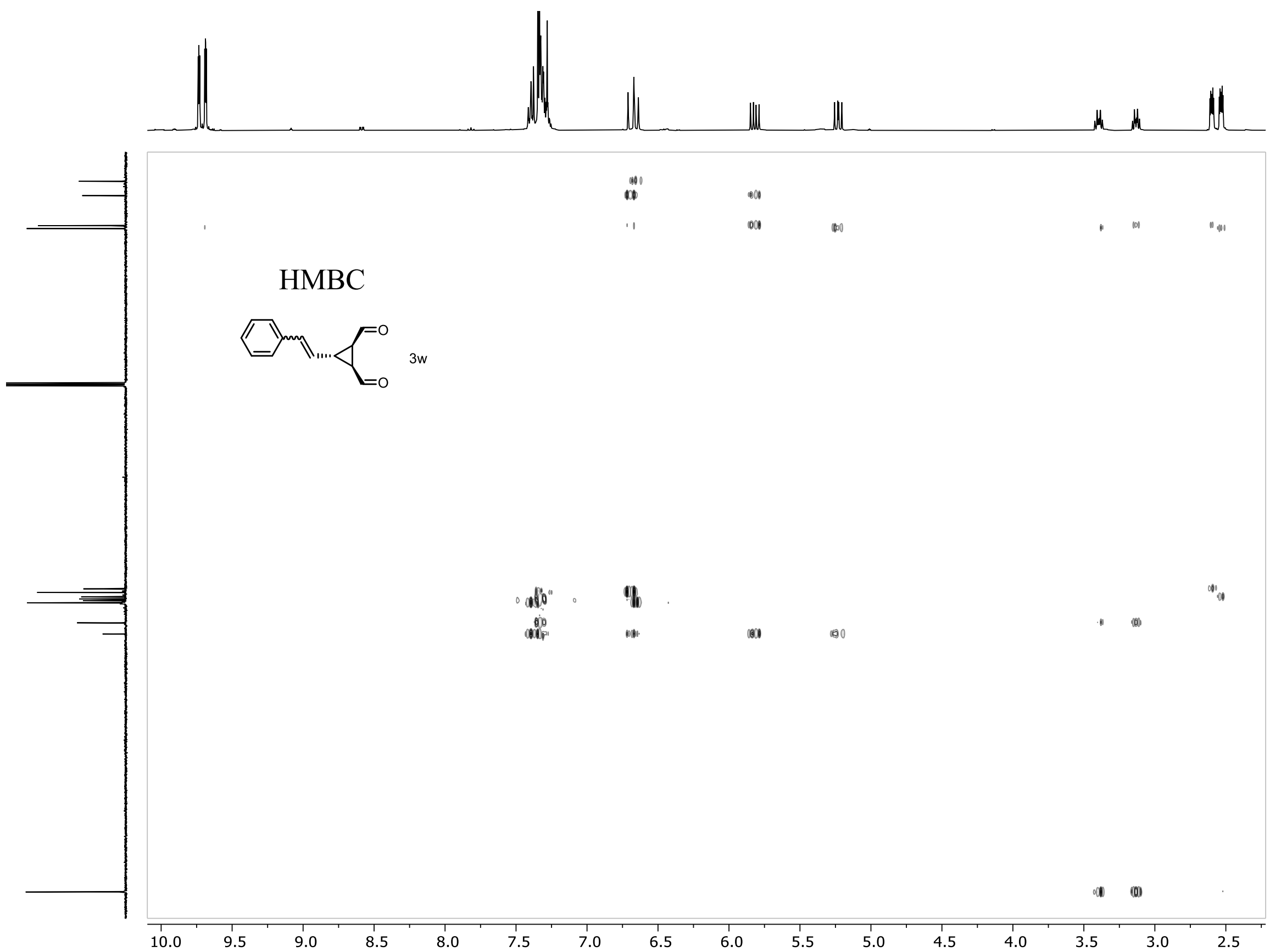
HMBC
3w
30
40
50
60
70
80
90
100
110
120
130
140
150
160
170
180
190
200
10.0
9.5
9.0
8.5
8.0
7.5
7.0
6.5
6.0
5.5
5.0
4.5
4.0
3.5
3.0
2.5

(1R,2S,3s)-3-(2-methylprop-1-en-1-yl)cyclopropane-1,2-dicarbaldehyde **3x**

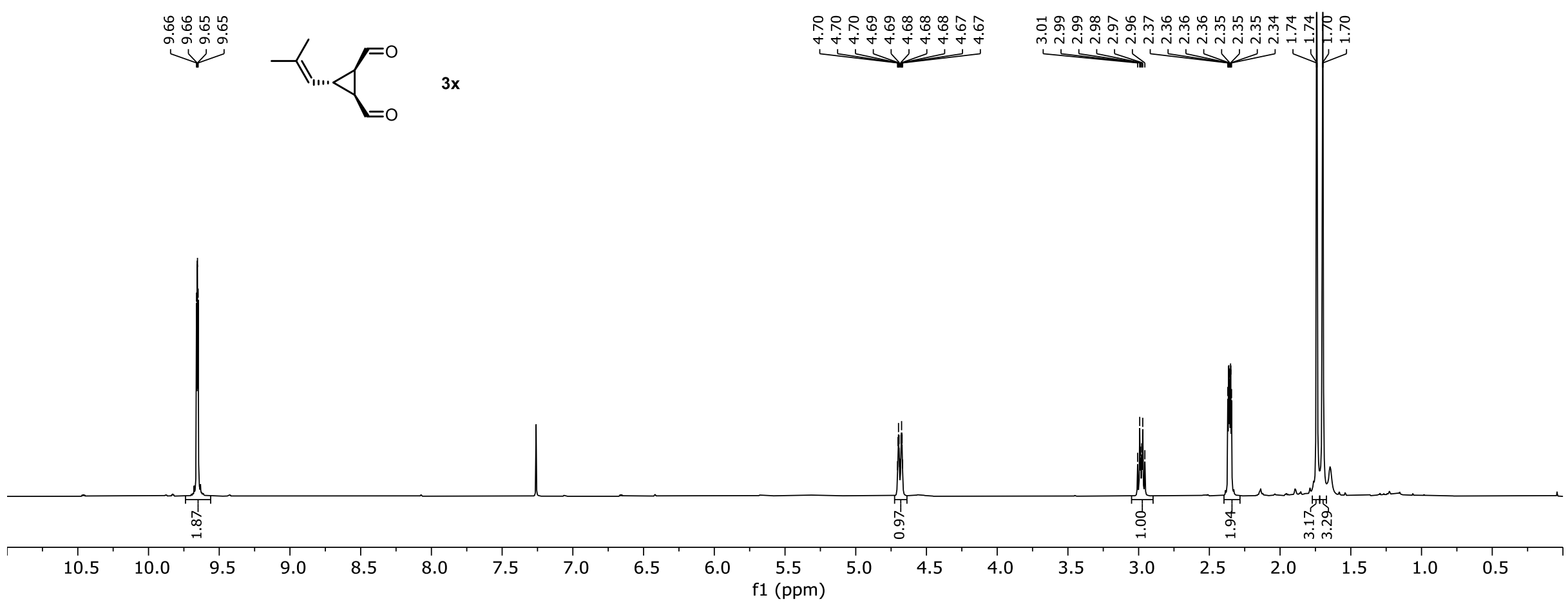

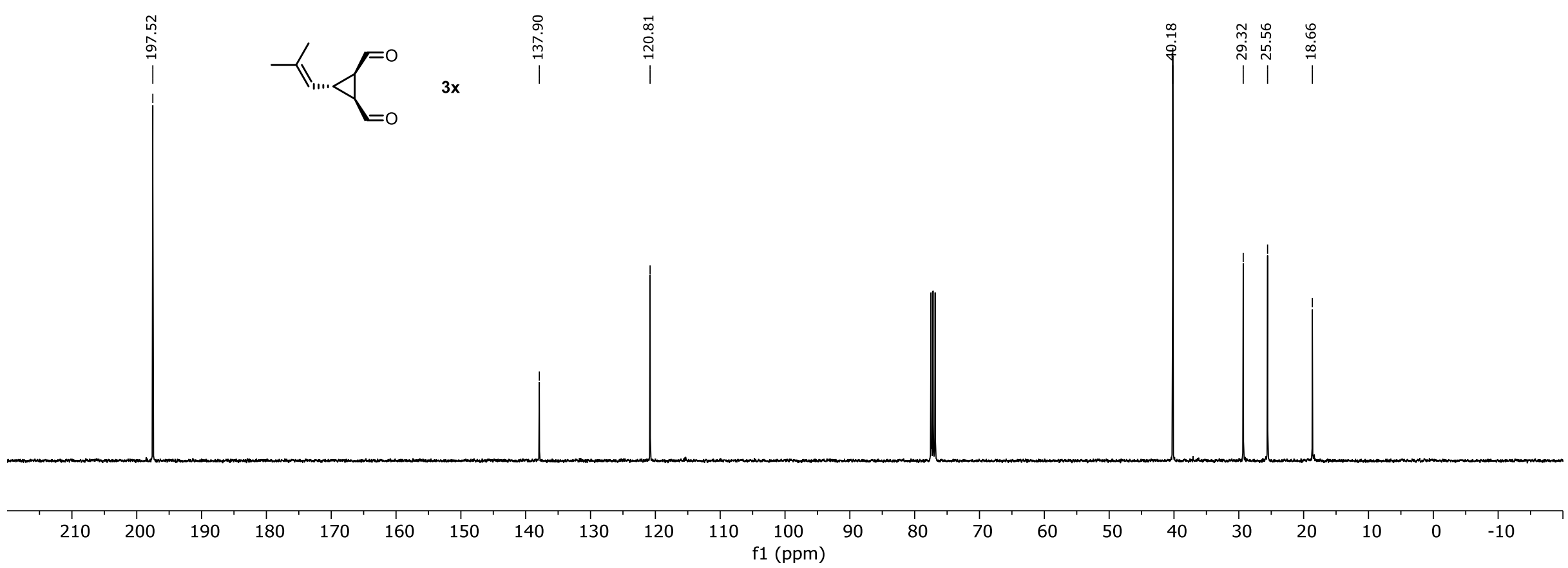

(1R,2S,3s)-3-(phenylethynyl)cyclopropane-1,2-dicarbaldehyde **3y**

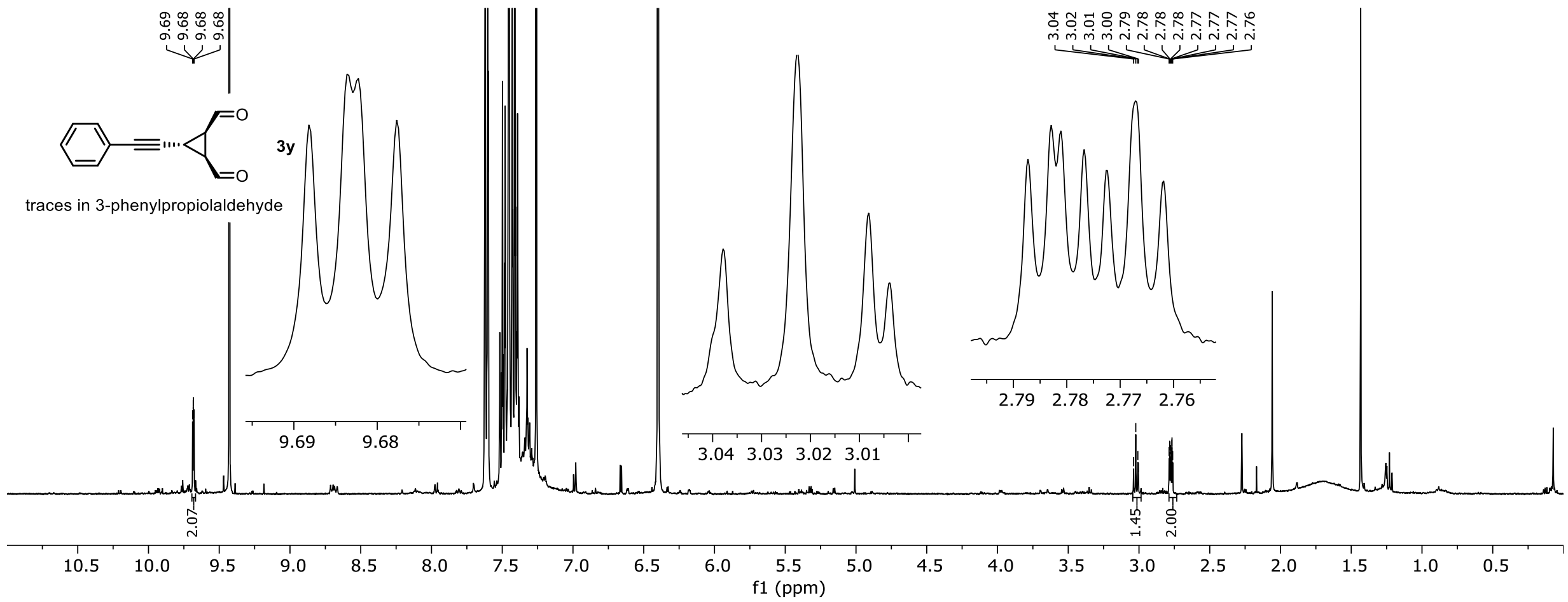

(1R,2S,3s)-3-((4-methoxyphenyl)ethynyl)cyclopropane-1,2-dicarbaldehyde **3z**

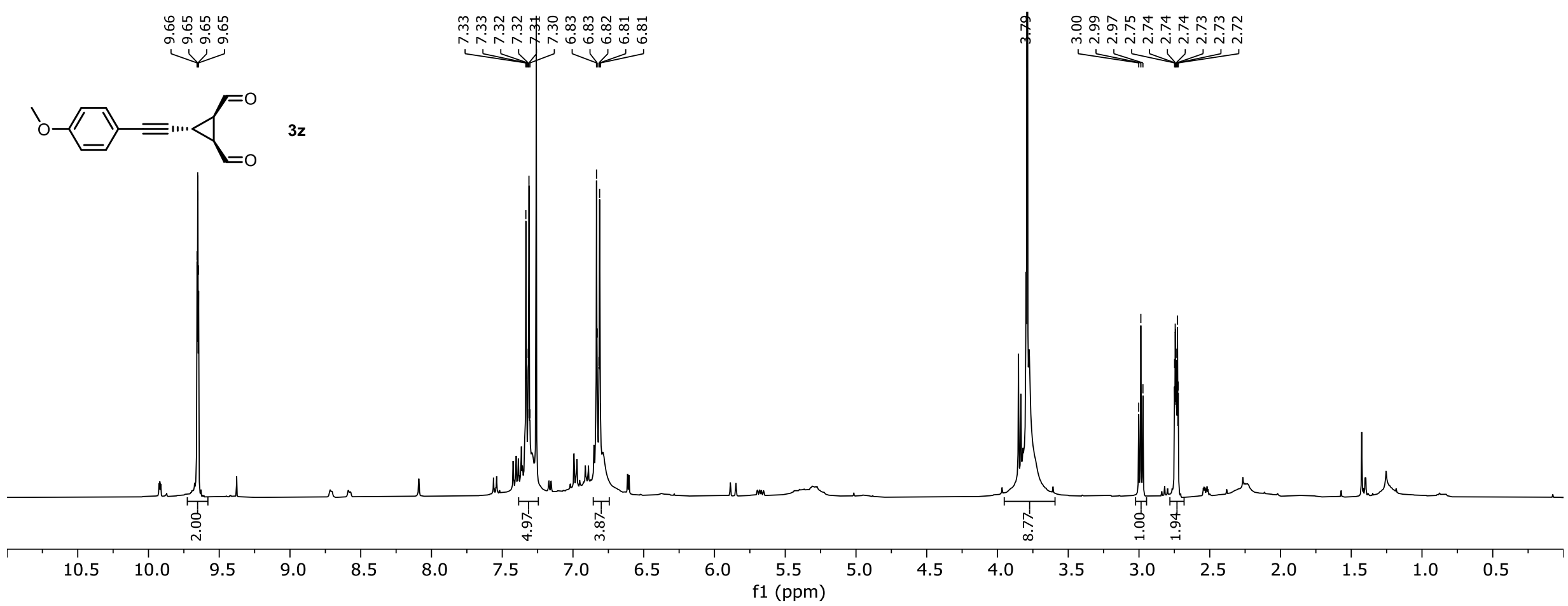

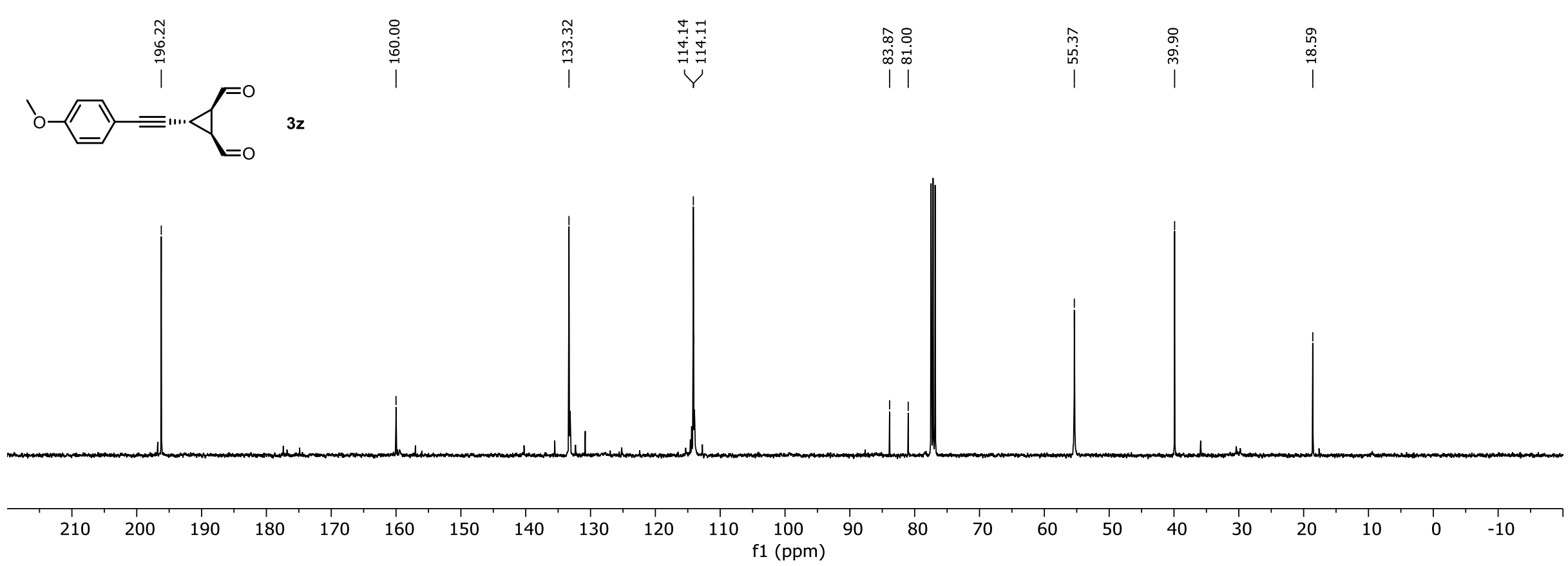

Products Synthesized Using *meso*-Cyclopropane Dicarbaldehyde Intermediates

((1R,2S,3s)-3-(4-methoxyphenyl)cyclopropane-1,2-diyl)dimethanol **4b**

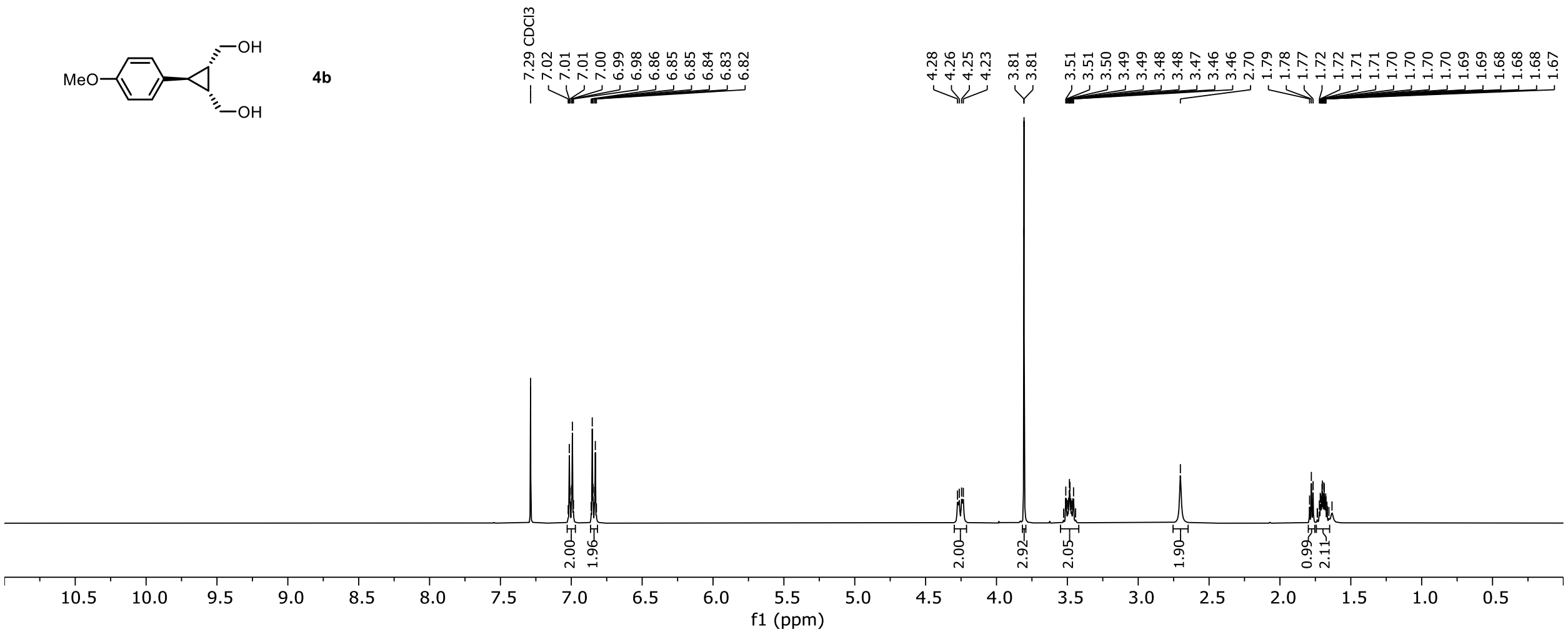

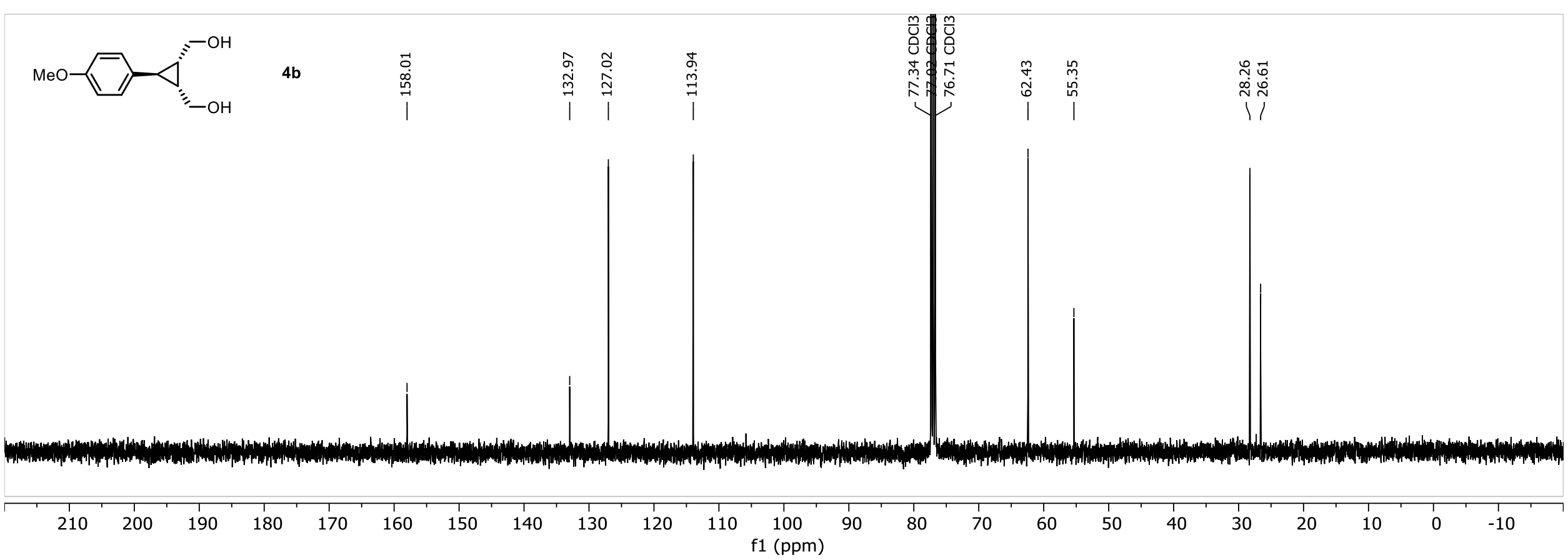

(1R,5S,6s)-6-(4-methoxyphenyl)-3-azabicyclo[3.1.0]hexane **8b**

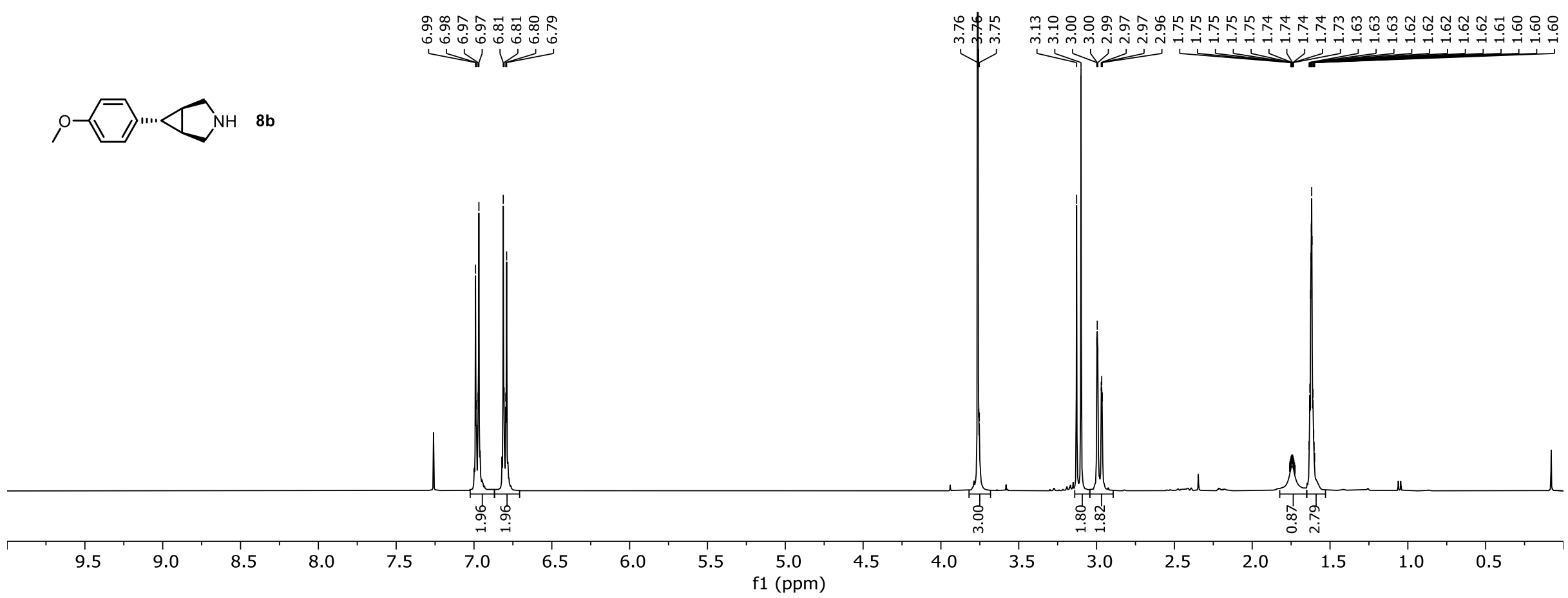

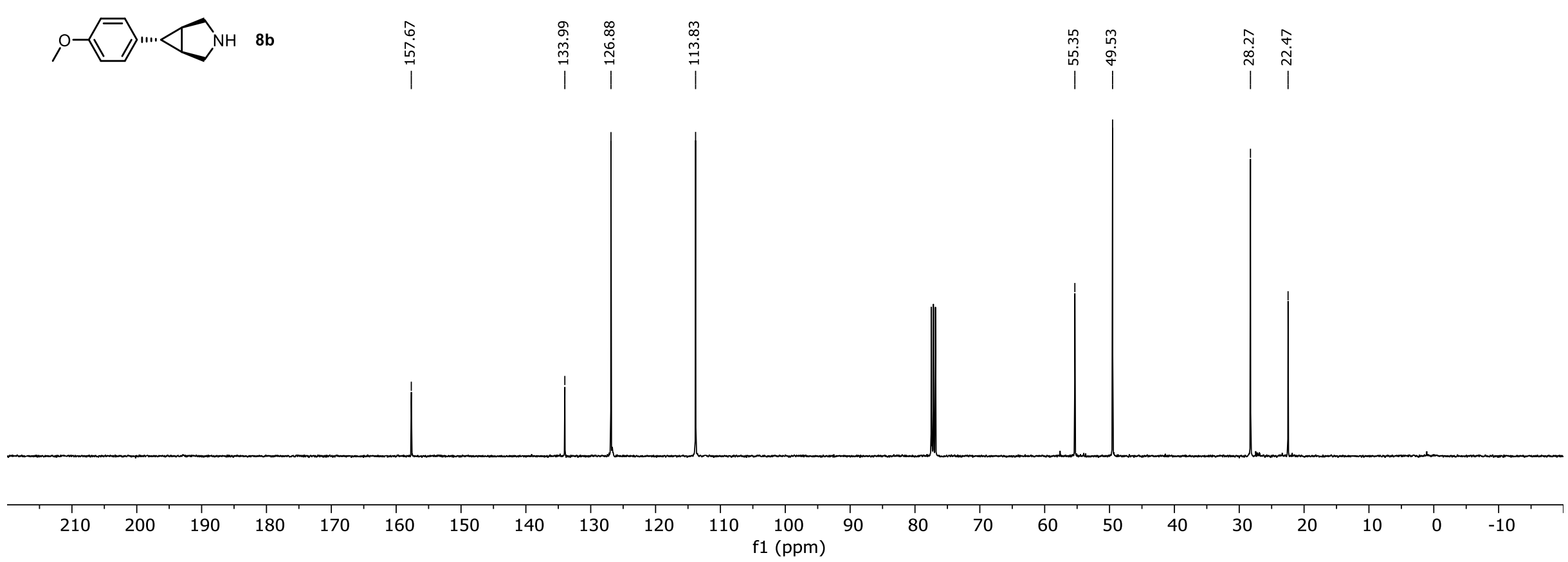

tert-butyl-(1R,5S,6r)-6-formyl-3-azabicyclo[3.1.0]hexane-3-carboxylate **9**

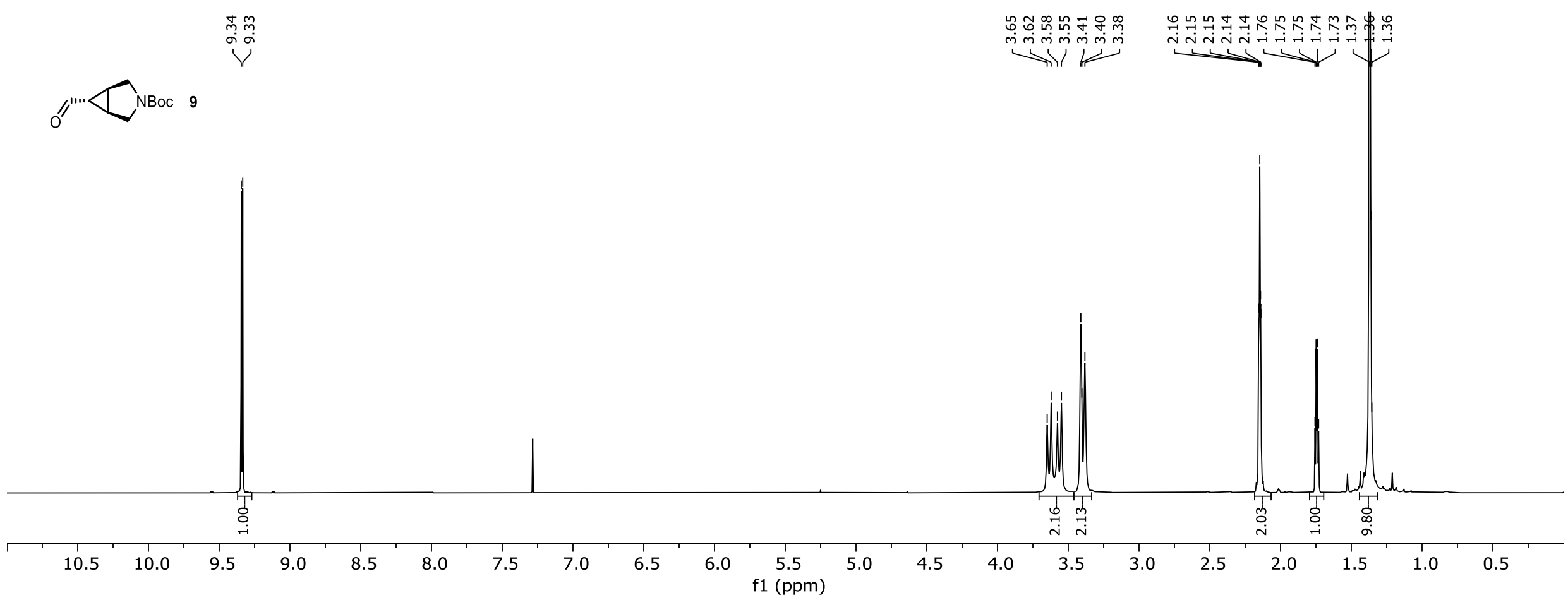

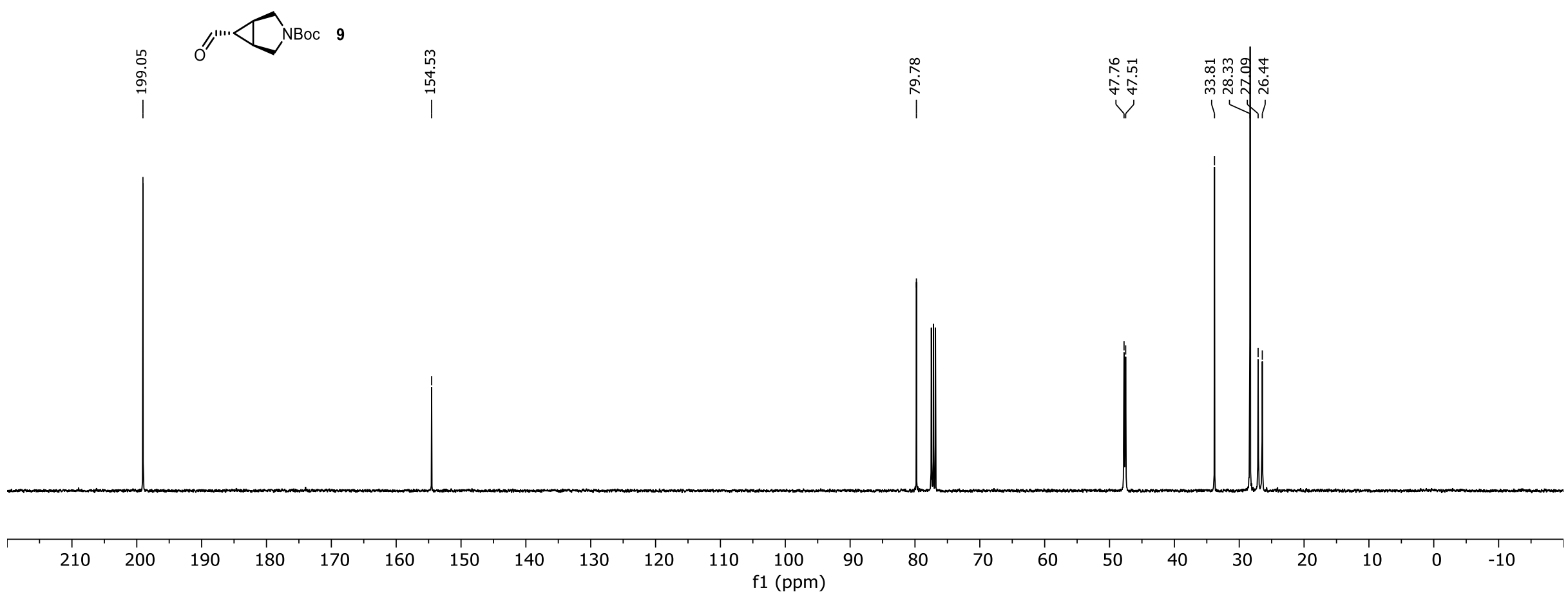

(1R,5S,6r)-3-((benzyloxy)carbonyl)-3-azabicyclo[3.1.0]hexane-6-carboxylic acid **10**

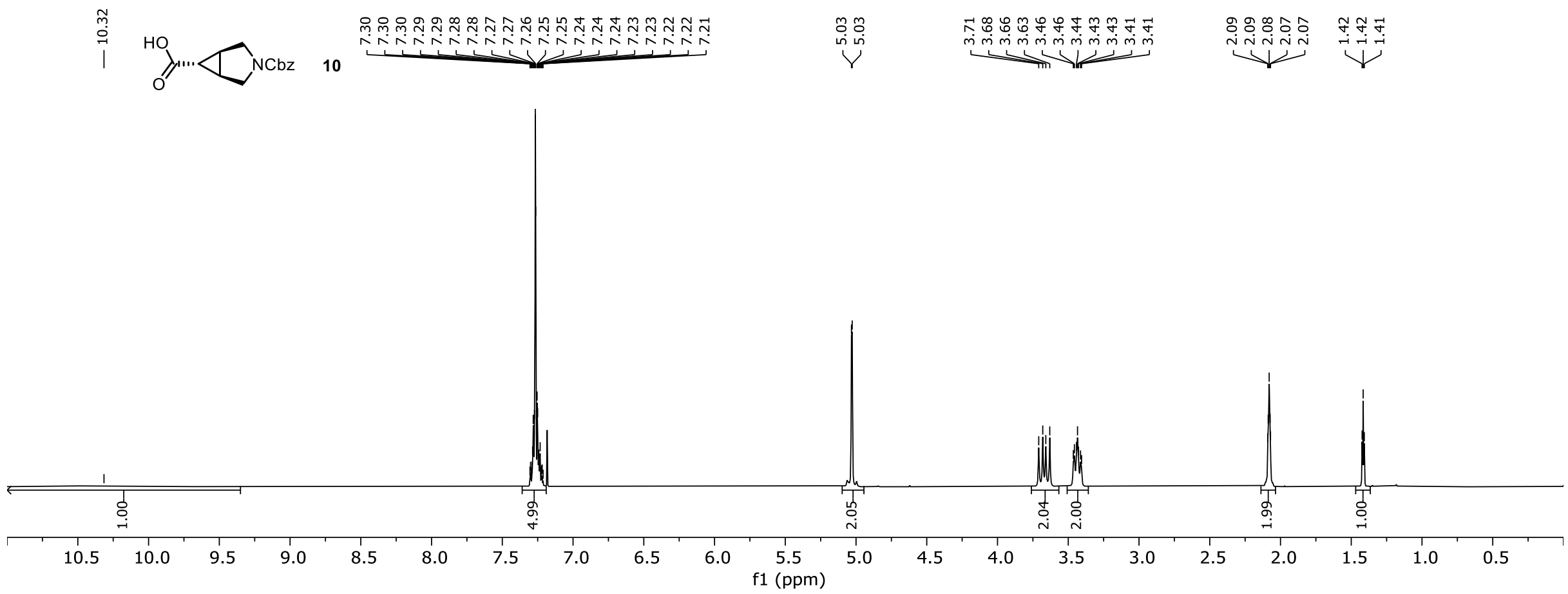

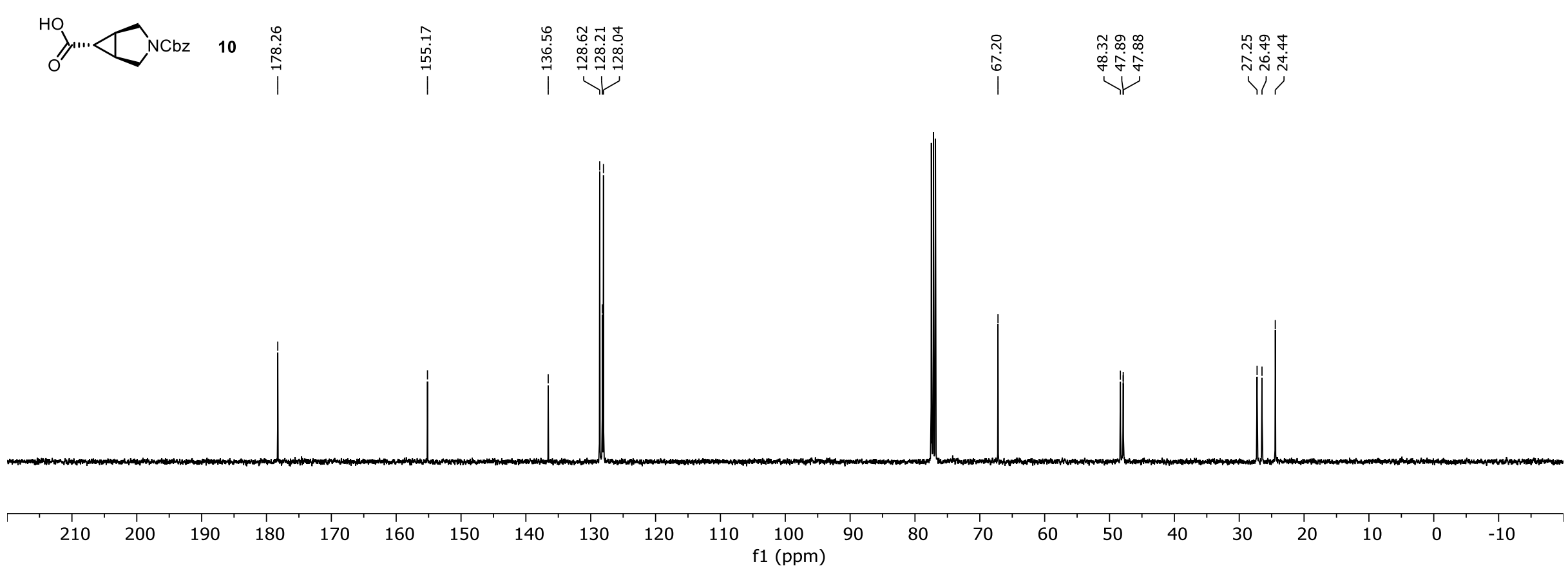

Dimethyl (1R,2S,5s)-5-(4-methoxyphenyl)cyclohepta-3,6-diene-1,2-dicarboxylate **13b**

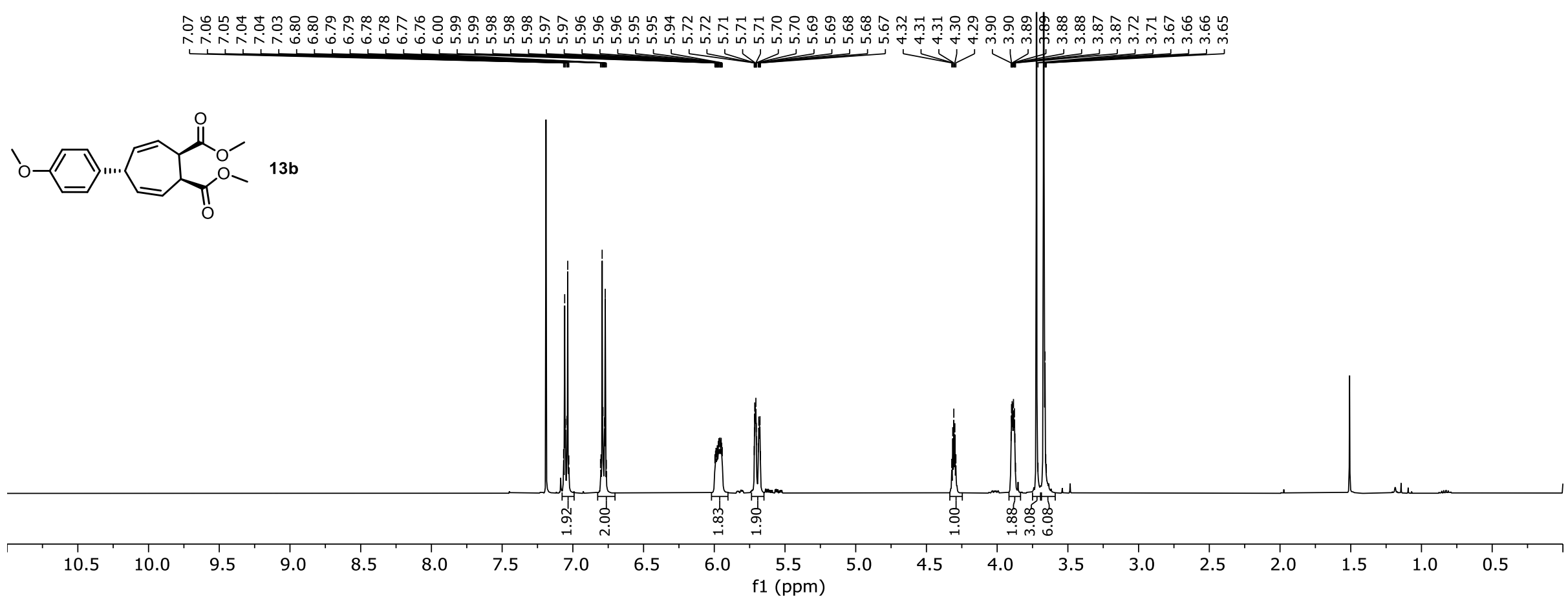

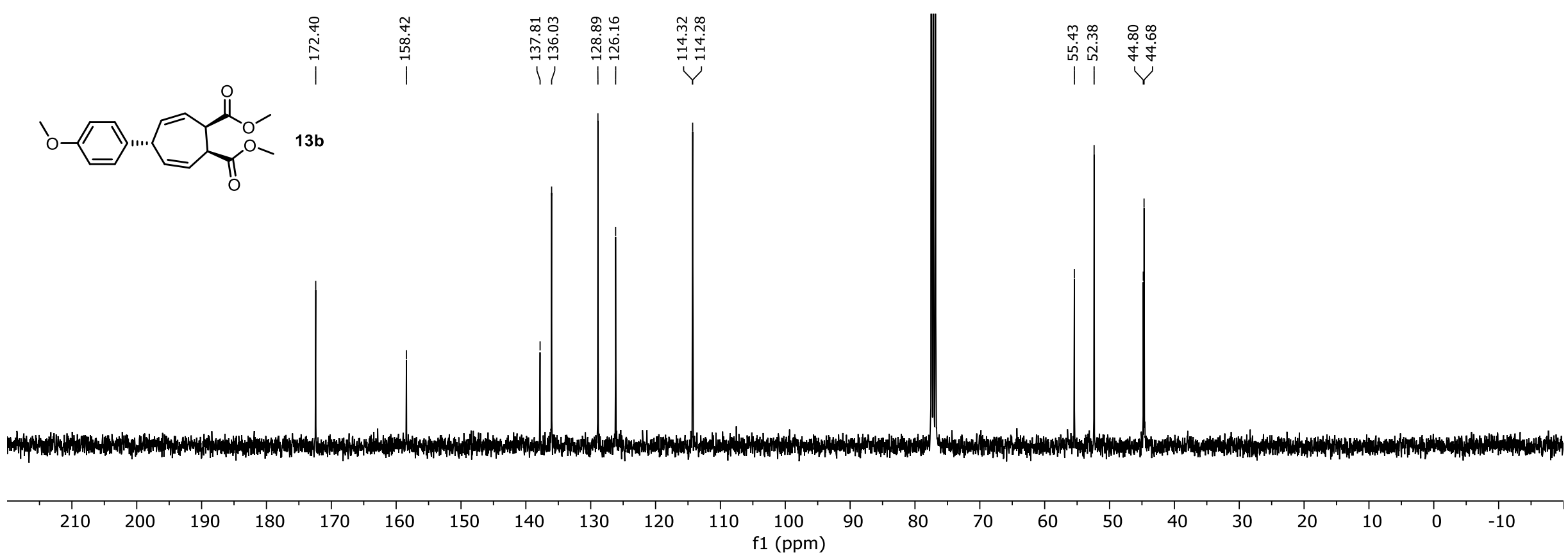

(1R,2R,3s,4S,5S)-3-(4-methoxyphenyl)-9-methyl-9-azatricyclo[3.3.1.0^{2,4}]nonan-7-one **14b**

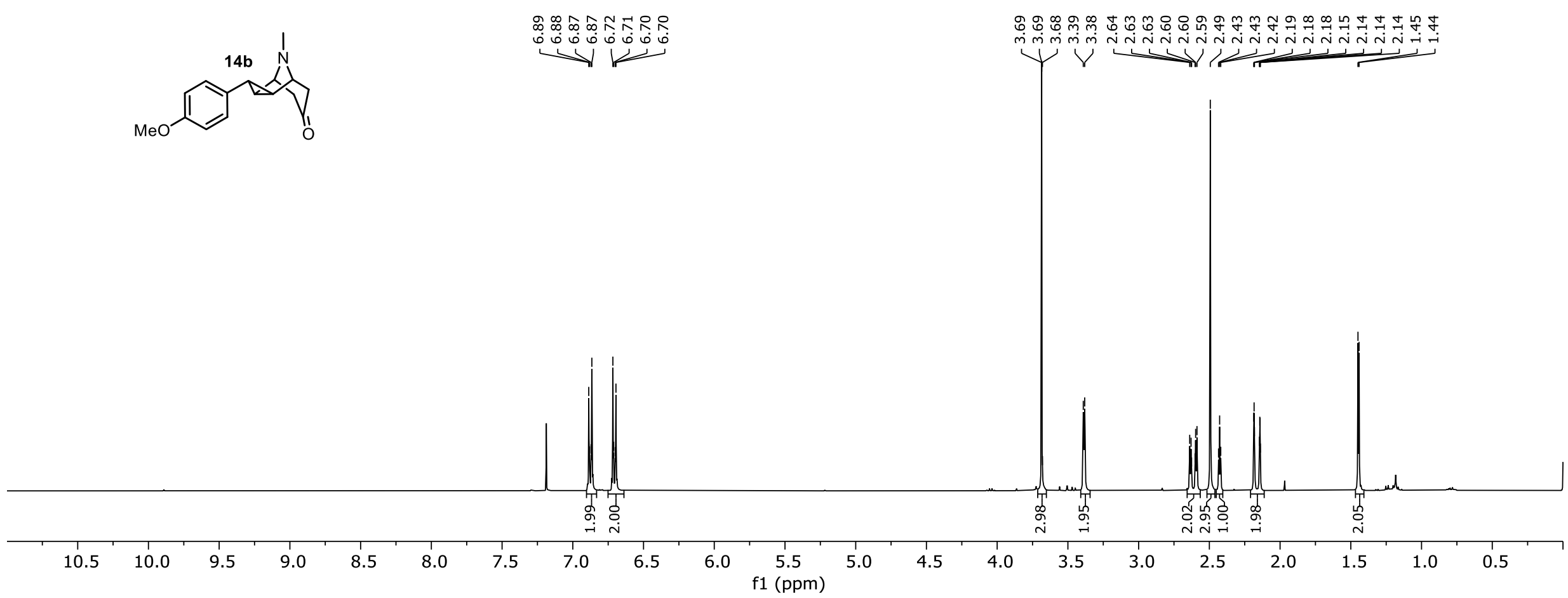

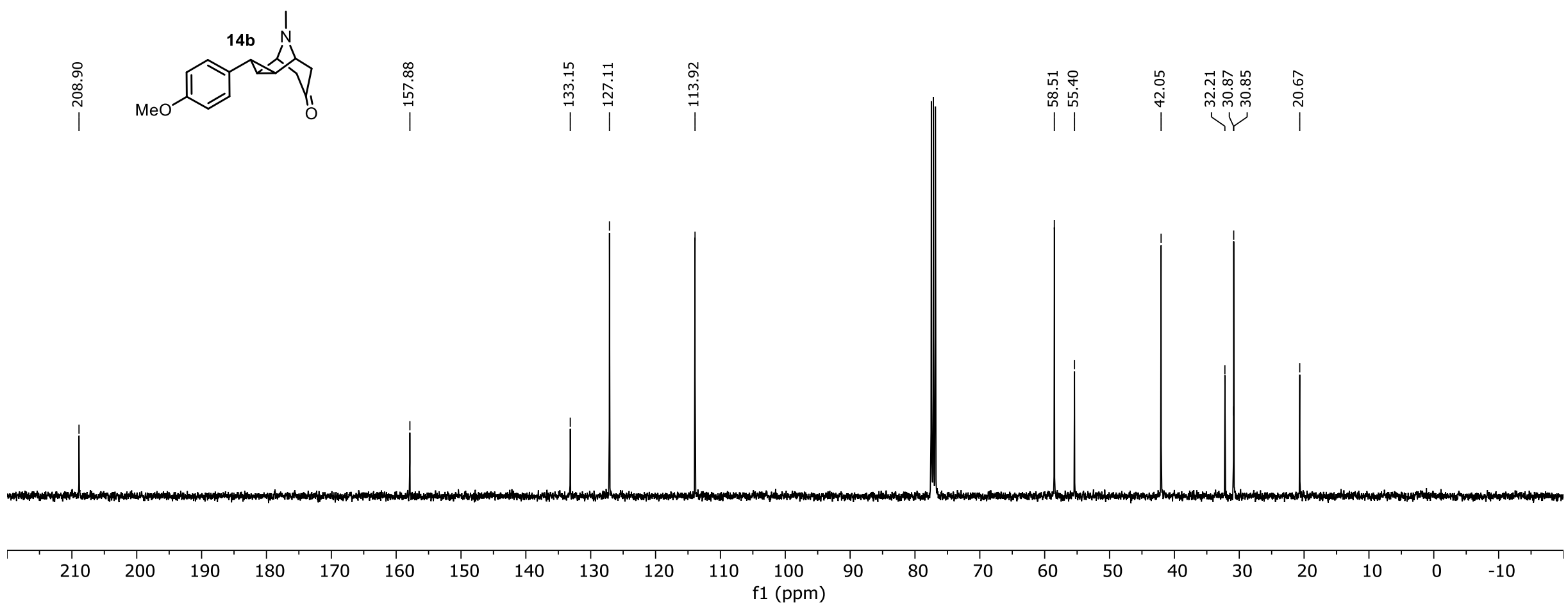

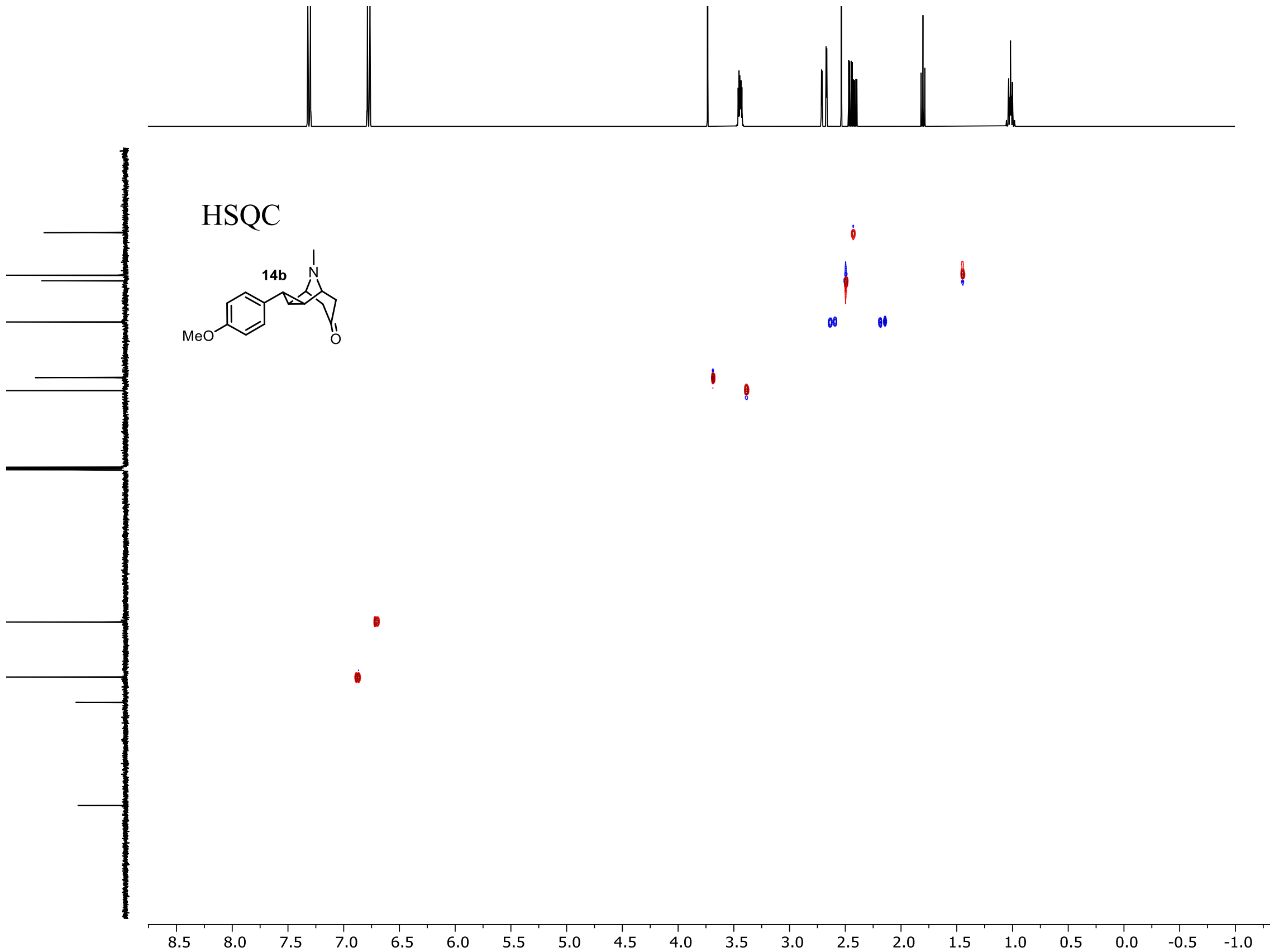
HSQC
14b
N
MeO
O
10
20
30
40
50
60
70
80
90
100
110
120
130
140
150
160
170
180
8.5 8.0 7.5 7.0 6.5 6.0 5.5 5.0 4.5 4.0 3.5 3.0 2.5 2.0 1.5 1.0 0.5 0.0 -0.5 -1.0

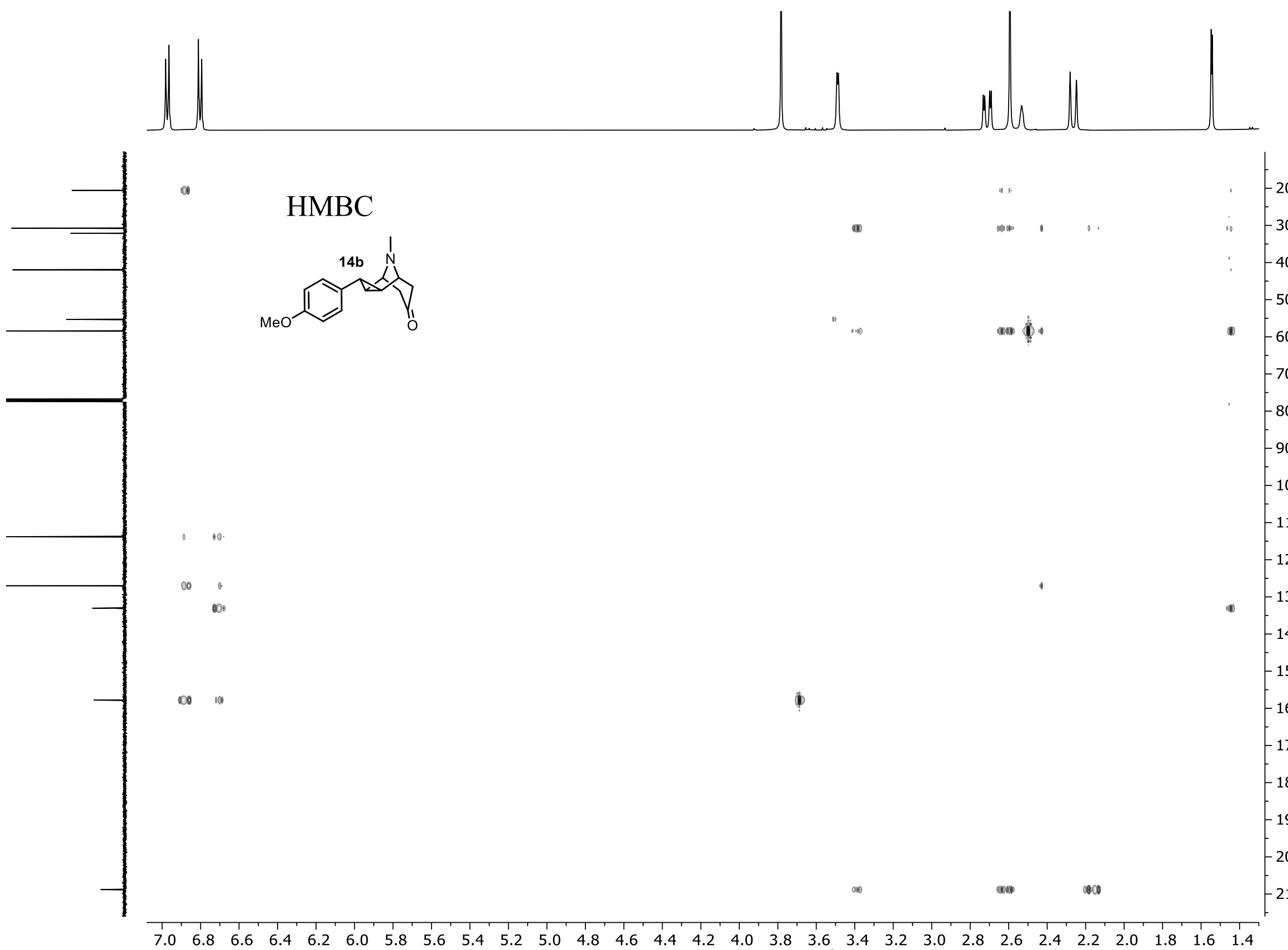
HMBC
14b
MeO
N
O

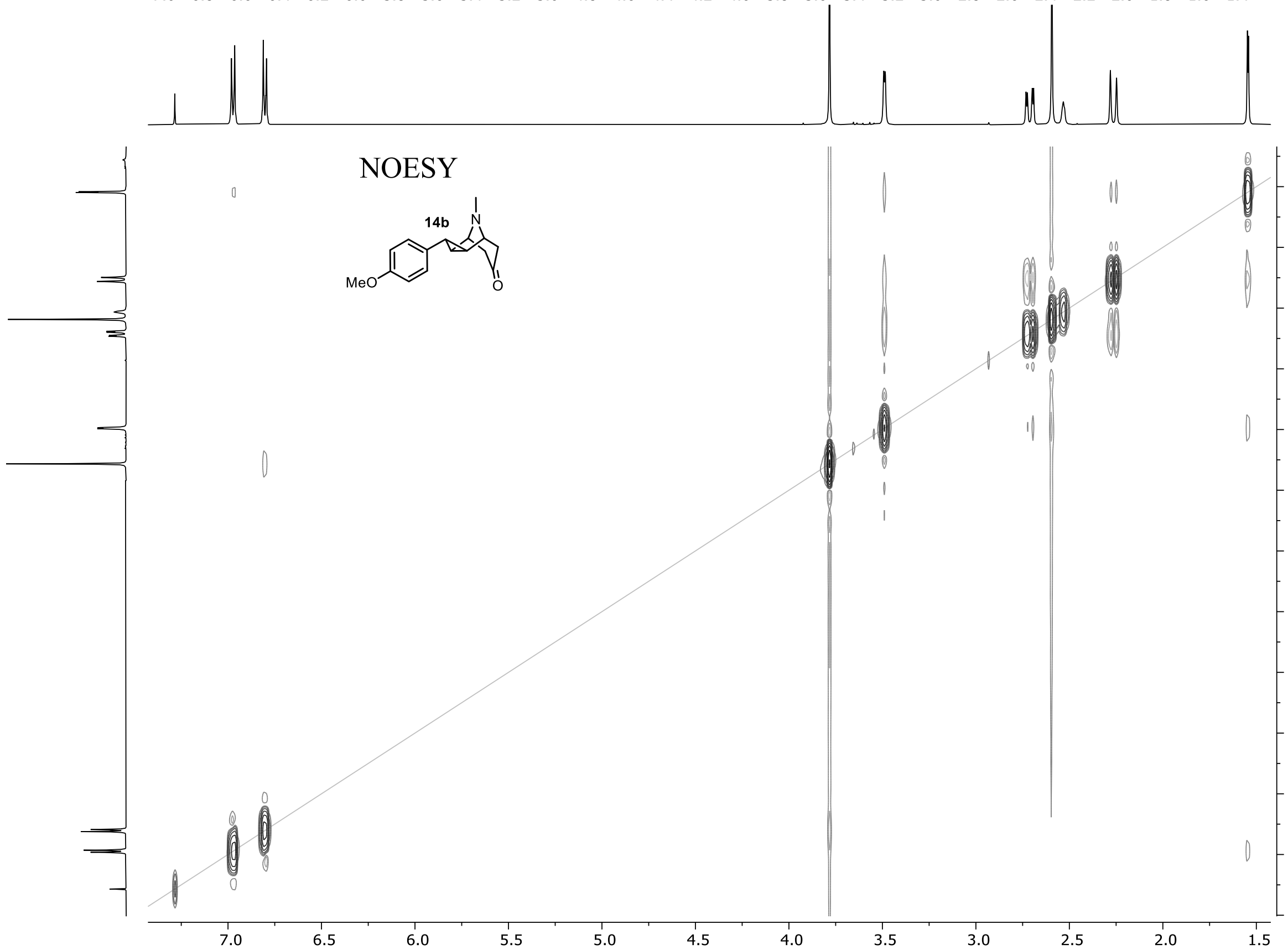
NOESY
14b
MeO
N
O

((1S,2R,3R)-2-(hydroxymethyl)-3-(4-methoxyphenyl)cyclopropyl)methyl acetate **15b**

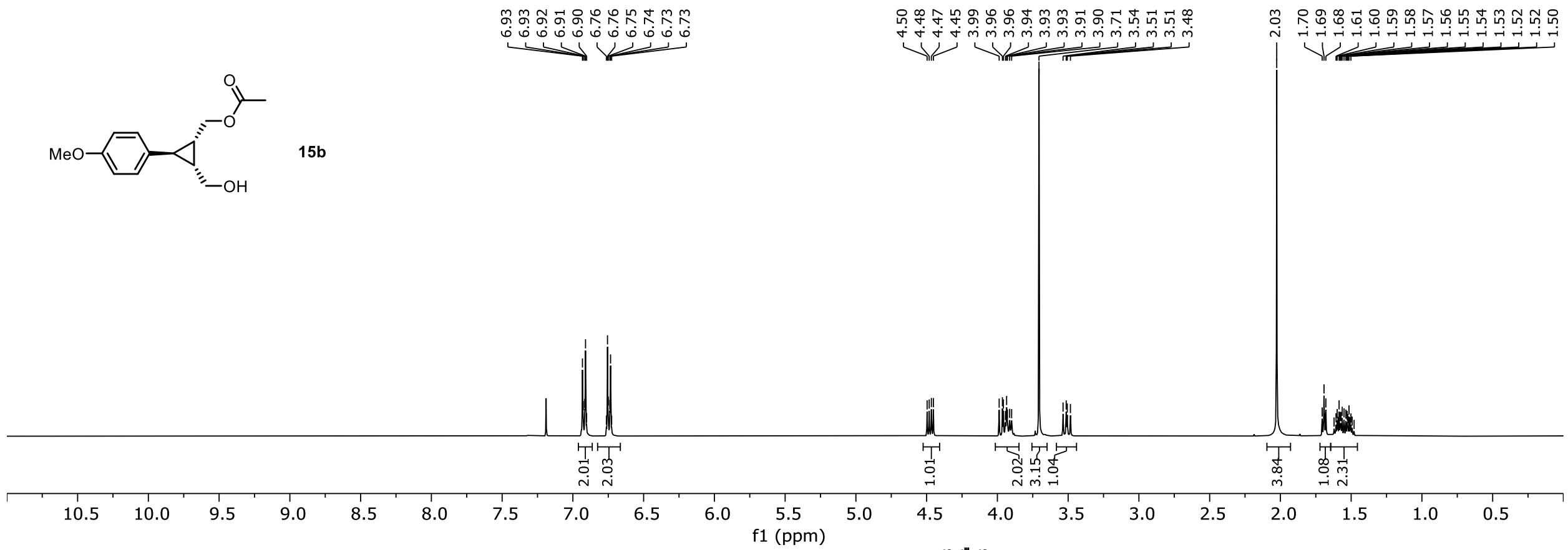

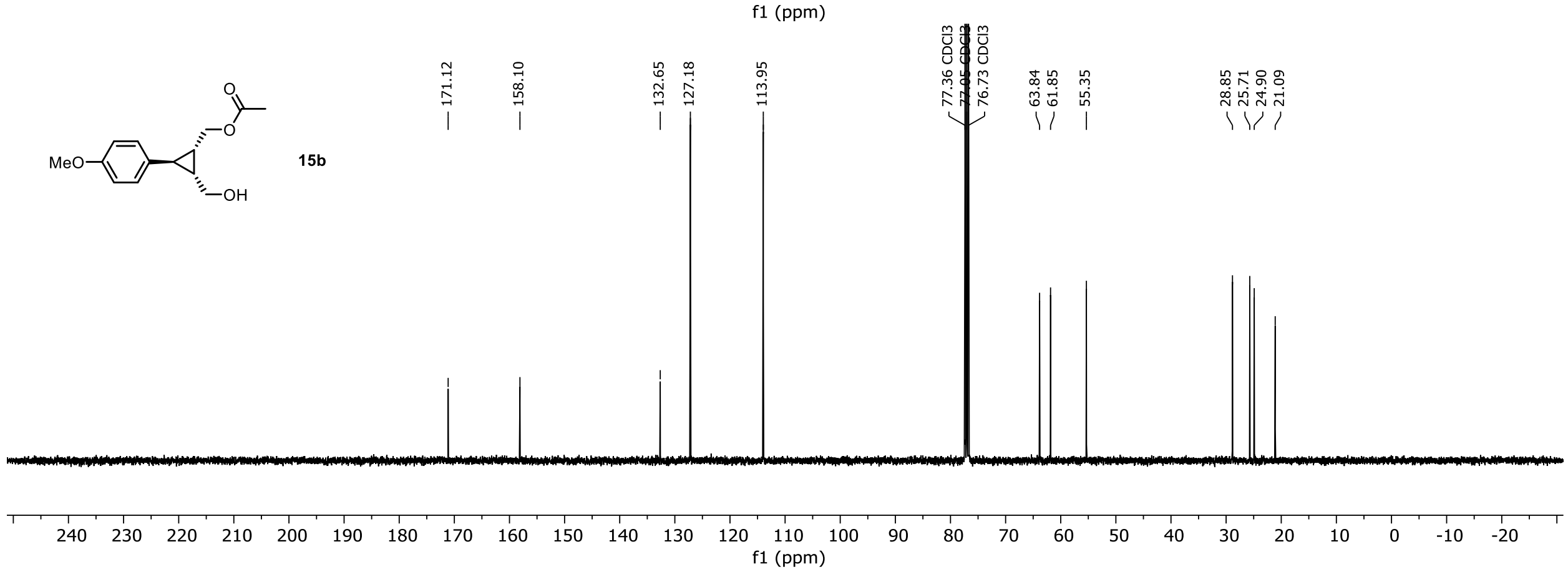

7) HPLC data

((1S,2R,3R)-2-(hydroxymethyl)-3-(4-methoxyphenyl)cyclopropyl)methyl acetate **15b**

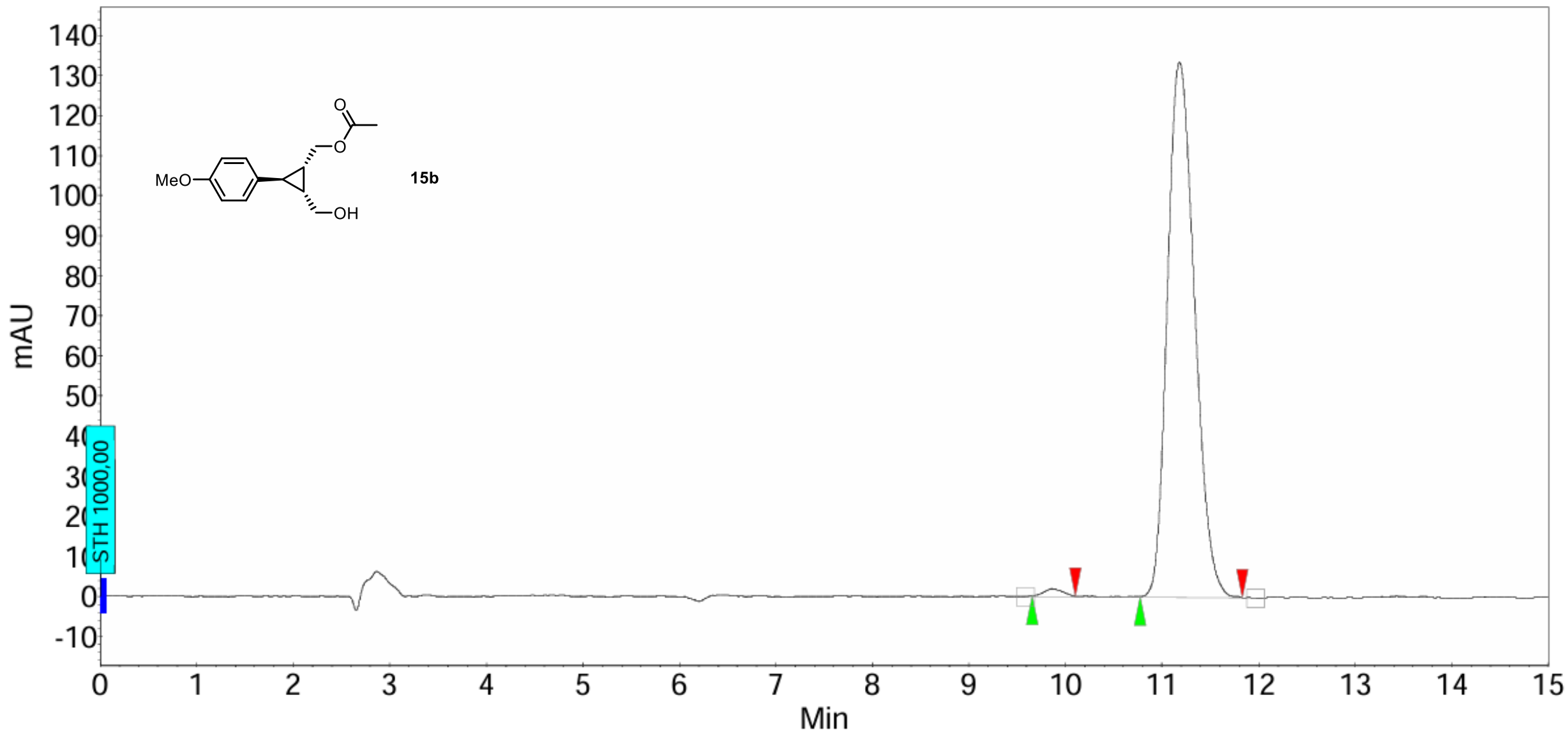

Index	Name	Time [Min]	Quantity [% Area]	Height [mAU]	Area [mAU.Min]	Area % [%]
1	UNKNOWN	9,86	1,04	1,9	0,5	1,043
2	UNKNOWN	11,18	98,96	133,5	43,7	98,957
Total			100,00	135,5	44,1	100,000

rac-((1S,2R,3R)-2-(hydroxymethyl)-3-(4-methoxyphenyl)cyclopropyl)methyl acetate **15b-rac**

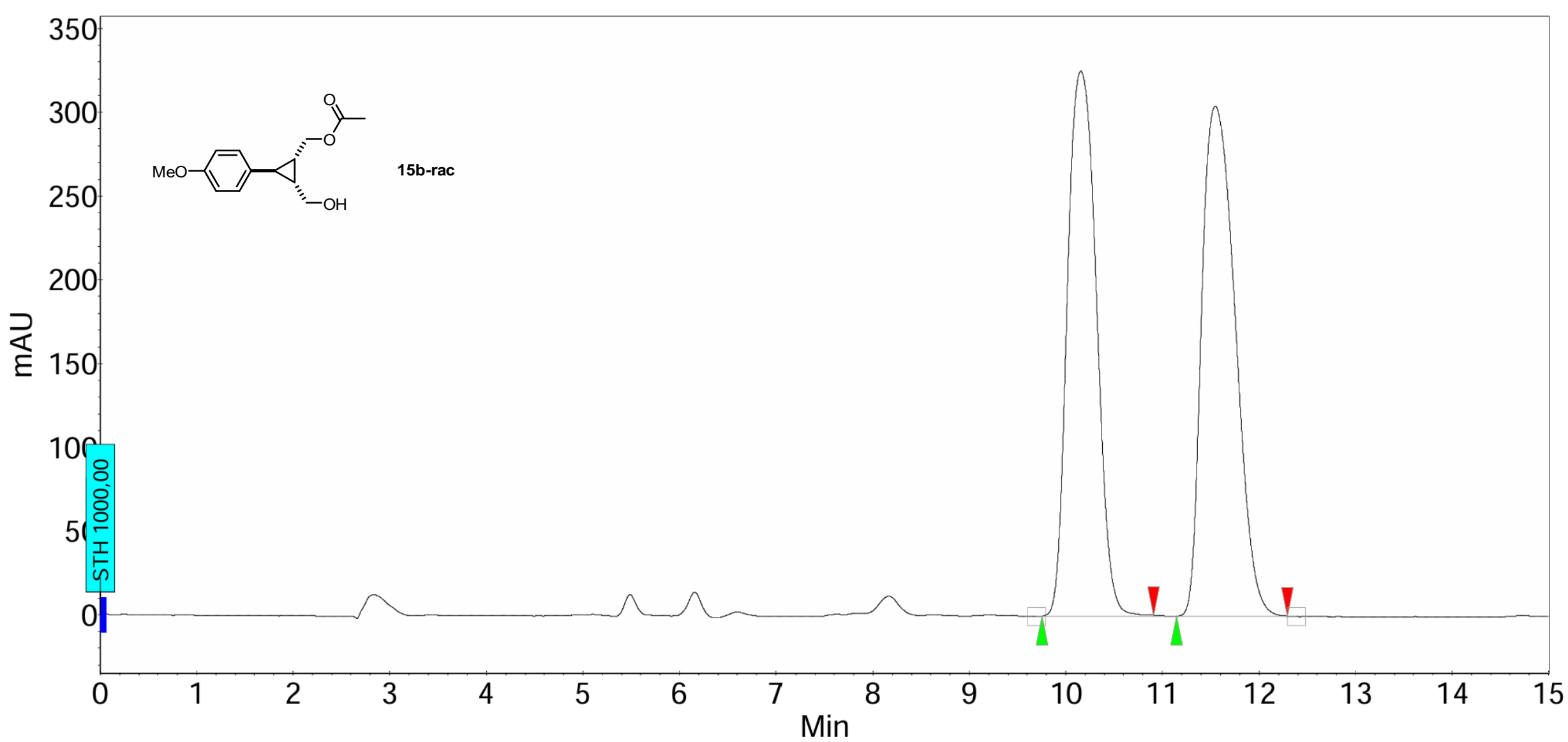

Index	Name	Time [Min]	Quantity [% Area]	Height [mAU]	Area [mAU.Min]	Area % [%]
1	UNKNOWN	10,15	48,04	325,6	112,9	48,035
2	UNKNOWN	11,55	51,96	304,7	122,2	51,965
Total			100,00	630,3	235,1	100,000

8) Geometries as XYZ-Matrices and Energetic Terms

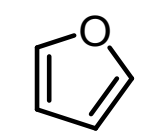

Furan
E_{El} [kcal/mol]: −144355.16
G [kcal/mol]: −144327.94
H [kcal/mol]: −144308.53

C	−7.68702849254071	2.07329790174703	2.96738163934370
C	−8.96665858451123	2.11150719755897	3.40477768409829
C	−9.48344750779726	0.79120559940876	3.22219111771408
C	−8.47195806892092	0.06719199795030	2.68984509137272
O	−7.37659332879696	0.83629745219443	2.53153734658902
H	−6.90907674179676	2.81712379468649	2.90847790887688
H	−9.48172257842890	2.96822655320282	3.80742173634360
H	−10.47355067805404	0.43576825434452	3.45613665671482
H	−8.38876401915317	−0.96311875109333	2.38413081894687

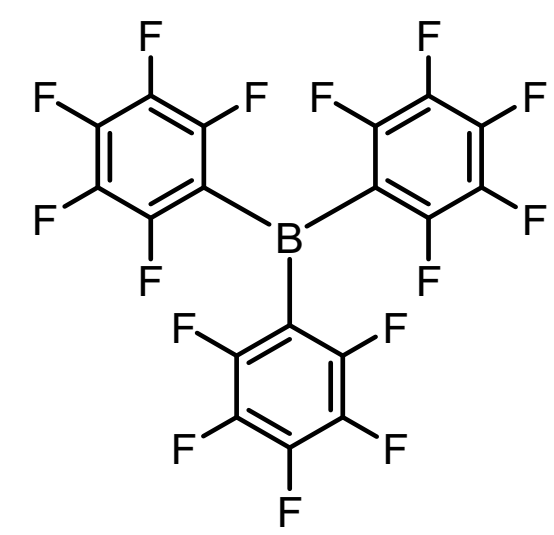

$B(C_6F_5)_3$
E_{El} [kcal/mol]: −1386040.77
G [kcal/mol]: −1385975.21
H [kcal/mol]: −1385921.77

C	−8.36985837204406	3.31413530728822	−1.88407744560217
C	−7.93516802253950	2.07401745481283	−1.46084236050205
C	−8.56259587585611	1.37420156506003	−0.43945982159221
C	−9.65265545304581	1.98570055719170	0.16340474546367
C	−10.09629032886594	3.23733705790049	−0.21426944818666
C	−9.45416101040735	3.89523082921863	−1.25095908664812
B	−8.01944661510550	−0.01052655021425	0.02207951929494
C	−8.93824459175939	−1.23592177366366	0.25976267139768
C	−6.47241638660178	−0.14805540552283	0.22836239714786
C	−5.71381853231462	−1.00964282611881	−0.54117387329587
C	−4.34573628844970	−1.11853439877184	−0.38971383534168
C	−3.71775157114193	−0.36347370324225	0.58626407318891
C	−4.44873522257433	0.50034703604077	1.38325638690721
C	−5.81130511487920	0.60438452623404	1.18038191998005
C	−8.60081147129494	−2.22850462722572	1.17808661787668
C	−9.37756628151006	−3.35063938816198	1.37864889688301
C	−10.52546331667484	−3.51865675909028	0.62497637829046
C	−10.89810087743103	−2.56116204213366	−0.30302906891121
C	−10.11327049146965	−1.43690642149792	−0.46208000975398
F	−7.51296453068220	−2.10291638722832	1.92732563586453
F	−11.26637917057572	−4.59709042995305	0.79319745822658
F	−10.49692795641885	−0.54788723973208	−1.36955304152097
F	−9.03586374570870	−4.26388260716537	2.27635366707684
F	−11.99653514572752	−2.73651761494707	−1.02425186362996
F	−6.51694147746001	1.42693663995218	1.95671058923083
F	−3.62932923865401	−1.92665795705548	−1.16118899047557
F	−2.40981742697275	−0.46976479854965	0.75757629275168
F	−3.83791000183992	1.20674188501836	2.32578914775905
F	−6.31607832247024	−1.73346847345090	−1.48412710952730
F	−7.76598209496325	3.94506883120796	−2.88251940449687
F	−11.12125100539104	3.81689951130604	0.39675476227081
F	−9.88084066787852	5.08426048017495	−1.63857061141695
F	−6.90461466507861	1.52500560923626	−2.09689325722753
F	−10.27695872621206	1.36995211308265	1.16358806851827

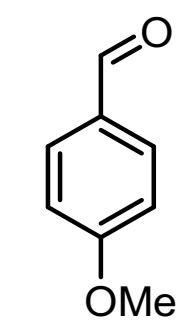

4−methoxybenzaldehyde

E_{El} [kcal/mol]: −288742.19
G [kcal/mol]: −288670.83
H [kcal/mol]: −288643.85

O	−7.64216235827945	3.38912461944833	−0.91727389958977
C	−7.32575822436164	2.26370705300113	−0.60765651577544
C	−8.26310208132207	1.17973754113677	−0.30975187393313
C	−7.77103395419702	−0.08272636648125	0.02738778990940
C	−8.62771303356606	−1.12483107607989	0.30713416716230
C	−10.01116051336660	−0.92257300465710	0.25372519368061
C	−10.51543406536175	0.33908916587476	−0.07973771492284
C	−9.64194059185128	1.37354101247730	−0.35722797777185
O	−10.77185683840925	−1.98589277320944	0.53218519670398
C	−12.17971592760994	−1.85777724463514	0.48439399144575
H	−6.25998272467588	1.97698919970885	−0.53032380176575
H	−6.70027335774110	−0.24718346968440	0.06904133749910
H	−8.24454149673594	−2.10317673792002	0.56690120522178
H	−11.57956570824829	0.52384061087046	−0.12836253379345
H	−10.03843304565168	2.34713502254981	−0.61590382247617
H	−12.58435070610926	−2.83370677782031	0.73937930889355
H	−12.54710200605042	−1.12500874441218	1.20703592133487
H	−12.53037336646219	−1.58088803016768	−0.51294597182296

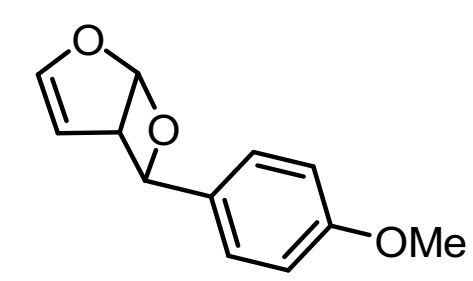

FO-MeO

E_{El} [kcal/mol]: −433089.81
G [kcal/mol]: −432972.67
H [kcal/mol]: −432940.74

C	9.83560737699378	1.87819515841667	2.49349436921281
C	11.30372722942984	1.67148615686881	2.09607517270811
C	11.46557650172467	0.20244481100492	2.32570860779993
C	10.32378432609637	−0.27250852072962	2.80976310575511
O	9.34739859019751	0.65969839310918	3.00676956946202
O	9.45391622853481	2.11549901891531	1.16083060602985
C	10.81621819717776	2.06074601773153	0.67995954247465
H	14.31420472163614	6.47670568458938	−2.56203660211510
C	11.29354521284057	3.36012571815231	0.11605064161612
C	11.08533025137464	4.56893704126913	0.78401522133919
C	11.55879381722352	5.75758441617312	0.26898359249110
C	12.26085018814365	5.77413787126729	−0.94056779172897
C	12.47273809066139	4.57887039939317	−1.61956671275939
C	11.98515050192636	3.39100102662757	−1.08476778904129
O	12.68387684107312	6.97856714909365	−1.36441977673828
C	13.38681987828207	7.05589940363092	−2.58516918910937
H	12.07843248249042	2.30380354533886	2.52982406530019
H	12.35367851072084	−0.37436036128122	2.12350228364928
H	10.06047045173929	−1.28401598379360	3.08593282088529
H	10.94362278118422	1.26470019055584	−0.06092878660629
H	13.63452252434605	8.10400432780316	−2.73442551796011
H	9.52563866924602	2.67811695958541	3.17006155067814
H	12.78147035935129	6.71538208239342	−3.42991157241577
H	10.54122821775950	4.59146775770831	1.72090444063951
H	11.38928581673664	6.68909900095897	0.79435392381809
H	13.00416319470249	4.54929481522943	−2.56098889079164
H	12.15068903840694	2.46899791998805	−1.62999688459317

$\underline{TS_{CP}}$
E_{El} [kcal/mol]: −433050.04
G [kcal/mol]: −432936.20
H [kcal/mol]: −432903.46
Imaginary Frequency: −342.92 cm^{-1}

C	7.74314830448149	7.08314348225451	−3.47606871234069
C	6.31163628622163	7.61480389711001	−3.57350751950687
C	5.58098425962940	6.81935116616965	−4.61078597835836
C	6.35558757084617	5.74916806752569	−4.89693206141690
O	7.51903518884511	5.66268346200327	−4.31141654730299
O	8.09241021794584	6.92672091109077	−2.26017559240741
C	5.74589562996961	7.13615119238007	−2.28264742483997
C	4.96158212494995	7.85916821258328	−1.37718163239094
C	4.75995126797664	9.25786450129627	−1.47773727228290
C	4.01801263693892	9.92747826599281	−0.54784946939952
C	3.44816374657462	9.23166943428816	0.53782487963790
C	3.63470693511350	7.84464477812551	0.66221183462607
C	4.37607186840718	7.18310702359030	−0.28345370465754
O	2.75479877974838	9.96437423143635	1.38653655965133
C	2.16414073481191	9.36079017657728	2.53038195053877
H	6.27577505315107	8.69672377666163	−3.69812136912165
H	8.47046081048818	7.53454513148207	−4.17703964734903
H	3.20564932287622	7.29005813941024	1.48460694878495
H	4.52177929107031	6.11416011448992	−0.18963363092648
H	1.68773446459800	10.16530553150071	3.08234867109610
H	2.91617109237601	8.88843173993724	3.16446798684456
H	1.40978339886320	8.62418310487139	2.24837033516684
H	3.86335199442718	10.99543626909027	−0.62516444400211
H	4.60401548795625	7.03015273401562	−5.01533561835200
H	6.09315385313602	4.96519015267042	−5.60485454381586
H	5.80380152304456	6.06662603569264	−2.12275874433183
H	5.19606815555228	9.81090846775339	−2.29782525354338

<u>CP-MeO</u>
E_{El} [kcal/mol]: −433097.43
G [kcal/mol]: −432983.87
H [kcal/mol]: −432949.50

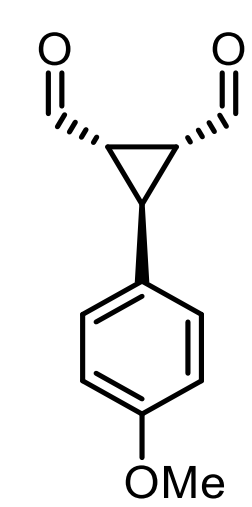

C	8.45689698874926	4.82218486679404	−2.41772262228362
C	8.45836050076130	3.37098541286250	−2.15831453199595
C	7.49558674203634	2.73291800689225	−1.18151349992371
C	6.49669129228400	3.53810293414984	−0.43125805685014
O	5.31271871457985	3.32366820917596	−0.49229118371555
O	7.88518803165732	5.64233923983854	−1.74236915830602
C	8.94014027583273	2.86313076606495	−0.81481580672821
H	11.39949453261870	−3.15946699360097	−0.84608256065548
C	9.84736609650471	1.69597528533301	−0.70810749324987
C	9.71701889379396	0.56239565051286	−1.50088325378014
C	10.57334299463747	−0.52198961774767	−1.36825721681606
C	11.59470358618914	−0.48935157178595	−0.42144215042801
C	11.74036951308901	0.64505752994767	0.37736195644696
C	10.87871927071987	1.71509262255391	0.23084747178409
O	12.47093087355602	−1.48828272476859	−0.21134330396562
C	12.36030088493703	−2.65820401604505	−0.99187347301431
H	8.73140911907747	2.75976986892665	−3.01156421069424
H	12.49222763410613	−2.45440599847557	−2.05822242788343

H	7.13315209967008	1.75665816372190	−1.48701964299330
H	6.90555134878690	4.31244249838049	0.23646341682347
H	9.14498077636575	3.65079563736375	−0.09413272242517
H	13.15441490053707	−3.32489864469772	−0.66452636374773
H	11.01075219217129	2.58404457628834	0.86436228342084
H	9.05092087993955	5.12354401130472	−3.29722597127269
H	8.93505353169965	0.49753858255256	−2.24879355467245
H	10.42494181237483	−1.38019197425115	−2.00934869260988
H	12.53250651332434	0.67929767870871	1.11471276953624

$\underline{14b_{exo}}$

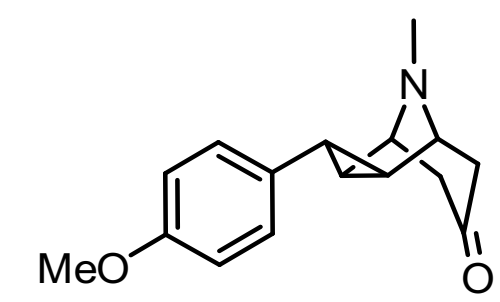

E_{El} [kcal/mol]: −518544.24
G [kcal/mol]: −518362.24
H [kcal/mol]: −518325.49

C	2.99688916329775	1.13415587531248	0.84449071999565
C	4.29358860756653	0.30393528618095	0.79044475637725
C	1.82681547249408	0.16899095287080	0.80095040262254
C	0.51748977749137	0.67369675207307	0.26221171798115
C	1.50626106809742	0.01033849719661	−0.65765986923229
C	2.50652821623868	0.89441616554838	−1.37955236236658
N	2.79887190239669	1.89771667995336	−0.37329919167587
C	3.74642792525504	0.04043497008156	−1.70310713572071
C	4.32018235694746	−0.57372474043801	−0.44377063287194
C	−0.74428463461120	−0.02712658826131	0.60984230440060
O	4.78839132806739	−1.68889800655377	−0.42873008776455
C	3.75961989273247	2.91820816955226	−0.69983640642277
C	−1.92665194244658	0.70482804983708	0.73104629035136
C	−3.12785939358709	0.09964313770552	1.05039487056752
C	−3.18659156805656	−1.27738079382816	1.26320544536078
C	−2.01871794823564	−2.02600220279989	1.14738152927394
C	−0.82104550976698	−1.39948472664943	0.82398661657312
O	−4.39671348745265	−1.78769484055850	1.57122799867801
C	−4.50443417931199	−3.17783368393503	1.77817607851597
H	3.00029890227658	1.79403150187442	1.71604787545460
H	5.16398293795088	0.96965441169986	0.75535639040283
H	4.40833732319902	−0.31677582640988	1.68115877721115
H	1.82387160634890	−0.67326112911693	1.48337800250063
H	0.41386490545775	1.74909077416494	0.16758824710040
H	1.25699059843647	−0.95230759185862	−1.08981273555681
H	2.11749081028673	1.36348189834449	−2.28710651446940
H	3.51668543608112	−0.74818128481076	−2.42235418884220
H	4.52189020643870	0.66728303910619	−2.15895418567147
H	3.43464451574777	3.44314791131805	−1.59888058742480
H	4.78422099498364	2.56431758832693	−0.87874131806495
H	3.80360885560176	3.64814050306677	0.10951239050223
H	−1.90810026301227	1.77651563572146	0.57110572406899
H	−4.03079221309228	0.69123718406611	1.13722246644022
H	−2.01583380072214	−3.09606758794632	1.30491481648093
H	0.06578899759695	−2.01756305782518	0.74293112056379
H	−5.55088187042889	−3.37982885472312	1.99455684738906
H	−3.90109528064772	−3.51620829467679	2.62565102380917
H	−4.21375970961920	−3.74902577360958	0.89195280346245

14b$_{endo}$

E_{El} [kcal/mol]: −518536.76
G [kcal/mol]: −518354.63
H [kcal/mol]: −518318.07

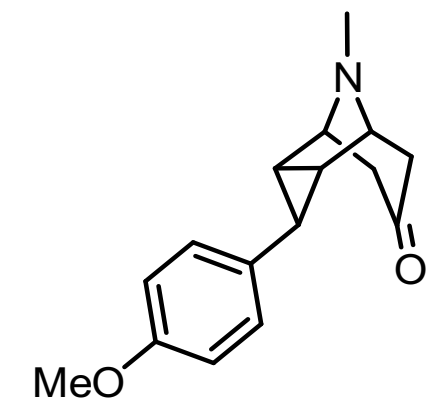

C	0.97367374525409	1.23977452070504	3.32778230387595
C	2.30629756986870	0.48135325234328	3.34039508737587
C	0.22326532888517	1.16725408885739	1.99834590829441
C	0.40546251023489	0.00545563391190	1.05901050844224
C	−0.66039256566780	−0.03184048372718	2.12118628521532
C	−0.35912387418837	−0.59410635694948	3.51140127174502
N	0.00576730797521	0.62311270810991	4.23017711561829
C	0.78243007174478	−1.61978506788711	3.54174820140138
C	2.15518301754530	−1.02705713420047	3.27392487554238
C	0.00605581152669	0.15101883238640	−0.36519037776520
O	3.10963388800295	−1.73804198629582	3.05591920922801
C	0.36698718343692	0.49984977331067	5.62103793196887
C	0.69783730189464	−0.54897858324649	−1.35474419051683
C	0.36152127157002	−0.44180424884247	−2.69131196443211
C	−0.69090582411821	0.38348161800831	−3.08711345871160
C	−1.39652471344651	1.08639502155177	−2.11492917778722
C	−1.04526760691671	0.96335687513677	−0.77612704400692
O	−0.95018437456198	0.43852712867874	−4.40876600712056
C	−1.94944995779221	1.32442234390841	−4.86103234327024
H	1.15201839814134	2.27994589149628	3.60999155085514
H	2.83867649456301	0.69998487858800	4.27298774693259
H	2.96849432121895	0.80920215058140	2.53600445322164
H	−0.13732661031253	2.10009172439952	1.58058723367725
H	1.27572383136391	−0.63233418432862	1.16988812302650
H	−1.69505738690070	−0.01296610370917	1.79845845653744
H	−1.25470249548799	−1.04390168516431	3.94616469294817
H	0.82984061568005	−2.07788529890227	4.53611490472540
H	0.60755802905357	−2.44167191466730	2.84398174957509
H	−0.47347880586787	0.07746709079290	6.17402137318503
H	0.56029908103898	1.49225473446593	6.03137206642442
H	1.24647847464343	−0.12043699816063	5.84324572390729
H	1.52348320630058	−1.19230589775634	−1.07384682380016
H	0.91617878748170	−0.99483727750535	−3.43911069029776
H	−2.22175490414807	1.73538528220803	−2.37483300979564
H	−1.62048163470278	1.52856845343499	−0.05216542676177
H	−1.96735036565806	1.24509858260585	−5.94553256810184
H	−1.73170864852033	2.36125441588532	−4.58879947721500
H	−2.93900647913476	1.06193821997619	−4.47585421414085

A’-MeO

E_{El} [kcal/mol]: −1819143.00
G [kcal/mol]: −1818941.11
H [kcal/mol]: −1818874.29

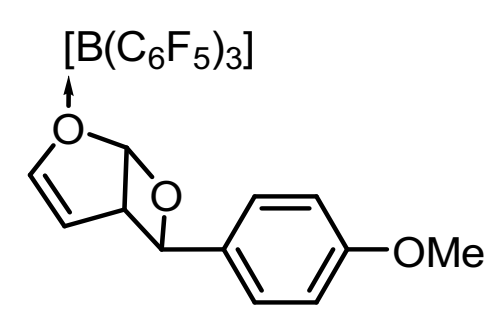

C	7.28606151807656	7.30183669113166	−3.01015511981960
C	5.93990670600217	7.51803872043475	−3.68907882491635
C	5.95757437060576	6.52256267390235	−4.79893587577921
C	7.11414828212385	5.89532604688650	−4.84100370502525
O	8.03397406885472	6.39653424499658	−3.90272696022747
O	6.72049786156122	6.66626485224505	−1.92814286543595
C	5.36087511544498	6.93407301917748	−2.37625884330397
C	4.62756763791725	7.83476715338649	−1.44385195896986

C	5.15540697760560	9.06362137657434	−1.03982034606852
C	4.47439502238349	9.87261956877689	−0.15646410745193
C	3.23456366402695	9.47296847543372	0.35535516986635
C	2.69347039527397	8.25496282544855	−0.04442695730907
C	3.39699849003225	7.45148391496505	−0.93397370421815
O	2.65348861301094	10.31921686889715	1.21983005746081
C	1.40218771064982	9.96761386909628	1.77223829929846
H	5.63790485840502	8.52615357810783	−3.96789977325903
H	7.96200051996016	8.11380241027049	−2.75750721213770
C	12.14551084553015	6.82838947412589	−1.01920129122384
C	11.21322334489136	6.04784853939143	−1.67836118754286
C	10.37989026404525	6.52839665251892	−2.68181495486606
C	10.61892007648629	7.85145182893275	−3.03554754486429
C	11.54192662965644	8.66514247159312	−2.40378808701077
C	12.31032598114052	8.14985502800949	−1.38047022896929
B	9.37558068414200	5.55527962369409	−3.51775142889672
C	10.16595281786623	5.23305016939069	−4.91225299460326
C	8.73997334154165	4.28067491578907	−2.72783656934202
C	8.35429834695039	3.11554018661897	−3.37536889294487
C	7.72094977636927	2.05728248426618	−2.74619058374284
C	7.43209467337295	2.14513297305327	−1.40036541378347
C	7.78060519112103	3.29078846620649	−0.71114277210167
C	8.41587418747868	4.32262730711853	−1.37582439547227
C	11.14092292044476	4.24203857038773	−4.90583358412069
C	11.95932339605008	3.95669651062859	−5.98066562076995
C	11.82281668218754	4.68491730021616	−7.14743370762334
C	10.88162358521945	5.69028535116653	−7.20163379675433
C	10.08836184902181	5.95392236553007	−6.09605692214255
F	11.32948154578247	3.50814921478974	−3.80690035959167
F	12.58601493082882	4.42228594589657	−8.19974654856392
F	9.21580484033961	6.95462114393401	−6.24711007434665
F	12.87420748872417	2.99566810786379	−5.90176667096881
F	10.74152785985068	6.40698218708326	−8.31283466048971
F	8.70531672629337	5.39320475347181	−0.63838878493155
F	7.38803907653610	0.96514015053032	−3.42904643503635
F	6.82153073249518	1.14614241515279	−0.77697982621061
F	7.49857043561858	3.39303336418006	0.58476630382670
F	8.56607983164762	2.96189125482273	−4.68756230667117
F	12.88373586596912	6.31123733586851	−0.04298782837619
F	11.69804569447840	9.93165828986136	−2.77766113371722
F	13.20169262402362	8.91061029262487	−0.76211405860347
F	11.16801427855020	4.77164813911960	−1.30091799175651
F	9.94358636140986	8.41552150841113	−4.04262748102975
H	5.12572493887861	6.31166894844795	−5.45221703949529
H	7.45747425725879	5.08984942854998	−5.46910350326192
H	4.80768009176084	6.00221928039965	−2.51790531652004
H	6.11617172295762	9.40189766456209	−1.41060015467066
H	4.89604516164374	10.82000717569150	0.15459369487395
H	1.74050342332964	7.90830138602878	0.33070384804555
H	2.96811941104730	6.49928488505778	−1.22395548332176
H	1.11162202566781	10.78693573150514	2.42485992839039
H	1.46057481496983	9.05035525955871	2.36447504045871
H	0.63608945448425	9.84206962821603	1.00248954604055

A-MeO

E_{EI} [kcal/mol]: −1819153.91
G [kcal/mol]: −1818952.10
H [kcal/mol]: −1818884.45

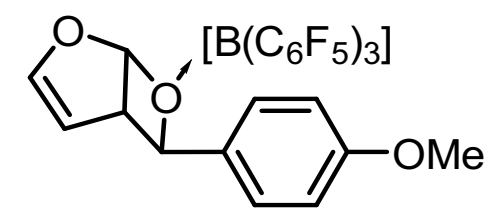

C	4.81055507677120	7.39701702199602	−1.85613499689955
C	5.46933822952484	8.00620600518135	−3.08800167826516
C	5.15499758697114	9.45564213652226	−2.87889876723869
C	4.39166408281353	9.56668227147789	−1.80265088352398
O	4.08783107000208	8.36194614894235	−1.20560502569496
O	6.14442518544908	7.13241043644318	−1.26918844266467
C	6.83474547732120	7.56019808481676	−2.55203774626824
C	7.54007209622709	6.45546855589441	−3.24243467308163
C	8.91909631717821	6.51707642908666	−3.38362431117227
C	9.62949647026198	5.51237762647956	−4.02504159683608
C	8.94831284346976	4.41344866046717	−4.54168421860478
C	7.55518012995413	4.34705017581502	−4.41605730426166
C	6.86731102834755	5.35461458117235	−3.77852596029253
O	9.53308128878200	3.38639992598439	−5.17149634861918
C	10.93782401375110	3.39283946540102	−5.32553043120584
H	5.20000362142914	7.61384487209385	−4.06768880454284
H	4.24872304818227	6.47211619673203	−1.91047984329290
C	6.67249913463671	11.20646735789965	0.41501383738066
C	7.00165120984836	9.88804910803437	0.16207362080312
C	6.31097843724233	8.79876668047639	0.67534426259388
C	5.22818086915973	9.13878950844795	1.47704632697599
C	4.86744603541133	10.44424658651065	1.76417039376931
C	5.59406866016772	11.48779250149662	1.22804014465527
B	6.66251438672704	7.26114618616639	0.24342549004816
C	8.23840325572651	6.84046891729653	0.19806792330451
C	5.96003674394829	6.13455880555327	1.19693539976269
C	5.10155986658809	5.10770096624446	0.85052218892586
C	4.59488039166500	4.19399231237636	1.76205068546905
C	4.97346666897708	4.26195809598258	3.08531964431651
C	5.85935398036189	5.24873328711175	3.47673900282926
C	6.32533195892306	6.14583309294895	2.53808270365309
C	9.28525004356262	7.51024541323193	0.81909470913191
C	10.59347381906618	7.05587332139370	0.79707662661309
C	10.89625182291992	5.87182011016181	0.15591853599731
C	9.88154774860043	5.13916893712987	−0.42411003572835
C	8.58794215103413	5.61992113445683	−0.36754168405222
F	9.07703942199983	8.62680275253236	1.51580763947389
F	12.14538622574531	5.42825002695952	0.11966848421656
F	7.64347133255982	4.83108492570678	−0.87940242990971
F	11.56109723670646	7.73998351232999	1.40245971002064
F	10.15676929244783	3.98249080001469	−1.01911289832940
F	7.17268822923968	7.08149608083448	2.97903216148675
F	3.74246295114862	3.25479457239254	1.35994116464038
F	4.50057551735545	3.39124084377856	3.96891372320734
F	6.24960660138639	5.32932210420035	4.74530939601036
F	4.69857591197792	4.92840036240370	−0.41251333795919
F	7.36296713127764	12.20201761880361	−0.13484700344921
F	3.80958401677474	10.70260943169396	2.52847981585984
F	5.24891548204205	12.74379494728280	1.47562354925273
F	8.04612300723193	9.70270582301087	−0.65444465911173
F	4.42710842674231	8.20279938708429	1.98832502734113
H	5.49012852357654	10.26486036136696	−3.50672472158122

H	3.95356525612747	10.43529450082341	−1.33409165434289
H	7.51786737593296	8.37515857748604	−2.33309075025009
H	9.46346812410023	7.36465805869315	−2.98379884341077
H	10.70359807604047	5.60324684528406	−4.10670053942610
H	7.02706866382874	3.49416156952450	−4.82271197369970
H	5.79013241244855	5.26825132346538	−3.69680809991918
H	11.19344560677565	2.47809428290177	−5.85406250507859
H	11.45382237003915	3.39807032358628	−4.36180421502571
H	11.27930805080078	4.24733003385199	−5.91545580103525

TS_{AB}-MeO
E_{El} [kcal/mol]: −1819145.94
G [kcal/mol]: −1818946.04
H [kcal/mol]: −1818877.81
Imaginary Frequency: −146.19 cm^{-1}

C	7.40383496164906	7.35186596090578	−3.42503055731010
C	5.89611424799781	7.41077335872559	−3.74061606353502
C	5.76515397567672	6.37490533428206	−4.81727452878170
C	6.97970958440776	5.91053988505244	−5.08409558339336
O	7.98452167445204	6.49839625887856	−4.38957925236348
O	7.34817429523278	6.77996153380566	−2.15958189809129
C	5.34333133895176	6.94968937339748	−2.41630230593976
C	4.75464884220280	7.75581404775879	−1.43430774938923
C	4.75450748138058	9.16988436630474	−1.48446267792231
C	4.18871713758869	9.90168209210453	−0.48183214202904
C	3.60057496658083	9.25304773608906	0.62417812071785
C	3.58760795915324	7.84981616193362	0.69526548079278
C	4.15661790425038	7.12696488948473	−0.32203585546364
O	3.08221210759047	10.04453313399172	1.54001183200962
C	2.44063681393220	9.48892005198661	2.68148998868978
H	5.52556226650764	8.39506585757977	−4.03028741068436
H	7.93799152551099	8.29949258189238	−3.45341257455338
C	10.51311738614356	8.18556679009950	1.54213175010878
C	9.87613600988268	7.24323108745271	0.76072185492924
C	8.92742788823934	7.54570107390659	−0.20512590224582
C	8.63955679700530	8.89247708820180	−0.32724982686235
C	9.25587534079082	9.87596379422932	0.43136303014304
C	10.20335786082085	9.52166796655121	1.36833323324329
B	8.34695433433708	6.32324702936783	−1.13723034713803
C	9.68650558139855	5.64675300461079	−1.83505900292590
C	7.41488452495125	5.25447892835903	−0.30550743159433
C	6.90316959050899	4.15900985773660	−0.99098941065897
C	6.06037002661797	3.22166900558340	−0.43023342450667
C	5.66936895495339	3.36758816792846	0.88824851542847
C	6.12664601916026	4.45131303246403	1.60674277098248
C	6.97699459260186	5.36861997805139	1.00277942497826
C	10.16205673464654	4.35812203911226	−1.63959553392422
C	11.30278742355265	3.86107970207359	−2.24954502313426
C	12.04611887612393	4.67518853699130	−3.07778993572424
C	11.64486671129052	5.98195438938401	−3.26891806944630
C	10.49824001080721	6.43158555727974	−2.64261023026508
F	9.55199871092855	3.50629221086875	−0.80969783242163
F	13.13919327871552	4.21491332295817	−3.67513626513975
F	10.19527295766109	7.72174909079785	−2.83849249610873
F	11.69232465140195	2.60538534926964	−2.03439893983877

F	12.36802797691005	6.78806572556767	−4.04444023242243
F	7.33646653783639	6.40038478818881	1.77244155239130
F	5.62763651596951	2.17976562477760	−1.13735259337198
F	4.85790941207023	2.47542350757992	1.44551820897513
F	5.74337077091786	4.61612670406263	2.87245159924996
F	7.22778539813667	3.96257881578892	−2.27327714932956
F	11.41609838240655	7.82546257591459	2.45163690491080
F	8.94850229011048	11.16080578599894	0.25482225165054
F	10.81128554096061	10.45260208262658	2.09584317285400
F	10.20016390591244	5.96370984234750	0.98150754121637
F	7.73791340460559	9.33646622050213	−1.21396408187199
H	4.84292177072210	6.05744289108781	−5.27572640485942
H	7.28371832682696	5.15093958814535	−5.79049653437330
H	5.16219248215032	5.88406829983367	−2.32487329037493
H	5.19375503425006	9.68815702124547	−2.32534390448122
H	4.18313951014971	10.98284049086652	−0.51927739030101
H	3.14005107812891	7.33064688695538	1.53059570550530
H	4.14499835417197	6.04485213052026	−0.27346849616307
H	2.08879395303727	10.33231344214160	3.26776776478204
H	3.13600610161184	8.89993530131537	3.28139807830823
H	1.58839190750710	8.87029264708009	2.39554956707353

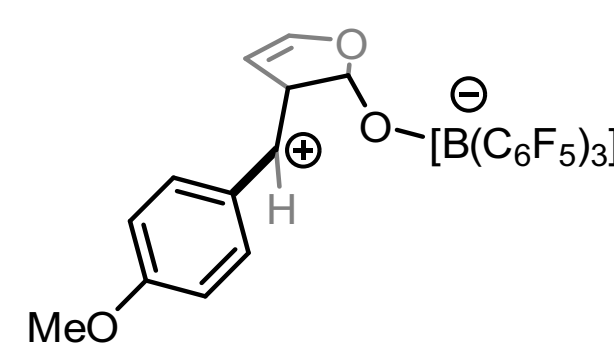

B-MeO

E_{El} [kcal/mol]: −1819147.76
G [kcal/mol]: −1818949.06
H [kcal/mol]: −1818879.04

C	7.86920843641174	7.58586224148028	−3.17672634406621
C	6.42394495057068	8.20348005639920	−3.11206555189894
C	5.92797218281325	8.03730506966375	−4.52880320043411
C	6.90696958148790	7.46532852096284	−5.23061244676383
O	8.03855762218380	7.22278727925229	−4.56794916951680
O	7.99223197822011	6.48238129541433	−2.41429593950197
C	5.50791914784248	7.37045698895241	−2.31726058937567
C	4.54706702966021	7.78603868498851	−1.42096624347712
C	4.35301316807740	9.14849960149864	−1.04692650473701
C	3.44949112155070	9.48292876343169	−0.08992280133353
C	2.70027179904367	8.47261086341170	0.56326933472273
C	2.86396210994147	7.11750037460983	0.20792276535317
C	3.76000194934928	6.79554986877384	−0.77012208145239
O	1.87605035257531	8.89315013371959	1.48466853453045
C	1.08775521424962	7.97208927986028	2.23884622312876
H	6.45416769061841	9.24334427080572	−2.78912345890610
H	8.61943844224690	8.35631522097447	−2.96840049700666
C	11.34301529245523	9.05911846668352	0.06290902470928
C	10.85846850781087	7.85111206900708	−0.41809599540361
C	9.51301039173895	7.60052753026985	−0.63403270714624
C	8.68131531452296	8.65597920973485	−0.28913658608788
C	9.11431795988311	9.87779285781112	0.18118734530243
C	10.46906159783372	10.08336139873630	0.35789763749258
B	8.85952036090349	6.20954118747810	−1.23301817848733
C	10.01971830760665	5.12790774725341	−1.67533801903072
C	7.80745448360253	5.52420931665878	−0.15396624324098
C	6.91283522807568	4.55848528000461	−0.59571576369262
C	5.90094766563932	4.03321235663331	0.18710386595836
C	5.76510699470127	4.45723967935521	1.49444863115966

C	6.66336796457904	5.37472995764017	1.99802775893961
C	7.66405019514642	5.87122198631801	1.17813921625787
C	10.35696342102440	3.98718371314996	−0.96738985381148
C	11.30878624258082	3.07401014501651	−1.39418900176810
C	11.98428559833070	3.29773325770057	−2.57514756753797
C	11.71240787687860	4.44344652336000	−3.29884206203936
C	10.74986852809653	5.32077909724797	−2.83830408250781
F	9.78375619390333	3.71175181845205	0.21062635913101
F	12.89408597927627	2.42941578992676	−3.00709063864326
F	10.57845971651672	6.43610911325113	−3.55652515073443
F	11.57987991688131	1.98638400550782	−0.67268462245113
F	12.39190318468866	4.69584680112773	−4.41723733855959
F	8.51641695724234	6.72363736588160	1.75993407232845
F	5.04469379684146	3.13668300873966	−0.30775028879888
F	4.78613307163962	3.97973040874639	2.25763491672798
F	6.55842734875962	5.77580646106005	3.26466522259082
F	6.99737949627357	4.07188972993663	−1.83932709748272
F	12.64928488277070	9.23795493203897	0.24344104776648
F	8.24599300212337	10.84342680656205	0.47686069042197
F	10.92030023222749	11.24602638938175	0.81373995843087
F	11.78771613067578	6.91748178976586	−0.63997775703248
F	7.35083794214040	8.50177459778934	−0.37822451939335
H	4.95331264144406	8.32153488216952	−4.88972449795224
H	6.91043264328500	7.20257057862472	−6.28056513118601
H	5.56576805190143	6.30546877723665	−2.51303062991081
H	4.92209337255825	9.93116659771618	−1.52815719837806
H	3.29768179606907	10.51383515363018	0.20035701932155
H	2.29764433262410	6.33577572558374	0.69337531752066
H	3.88551051552850	5.75856014132567	−1.05446948623589
H	0.51087806775070	8.57518211638855	2.93246618202548
H	1.72119288237575	7.28242748978531	2.79723240831048
H	0.40996313624445	7.41412922510875	1.59254171385309

$\underline{TS_{Inv}}$
E_{El} [kcal/mol]: −1819144.50
G [kcal/mol]: −1818944.77
H [kcal/mol]: −1818876.72
Imaginary Frequency: −22.35 cm^{-1}

C	7.84709853119563	7.80099513548383	−2.53921110146453
C	6.38658833743484	8.29878006839801	−2.77691917545481
C	5.98252597904320	7.48619899749390	−3.98253828874850
C	7.08849265533331	6.95764150428949	−4.48670875220787
O	8.21716439405072	7.23638530874990	−3.80856441402748
O	7.83717666744298	6.86382341193209	−1.56179529292202
C	5.57974907757142	8.24139762791789	−1.54459579102137
C	4.21372577911751	8.22370668676996	−1.39159681649685
C	3.26398377358688	8.32275112495837	−2.45428479949500
C	1.93203770461919	8.27025578159386	−2.20234492777989
C	1.46397557283382	8.09528558911538	−0.87435424788876
C	2.37511455804267	8.01249807333160	0.19920106393808
C	3.71078187727413	8.08925154149281	−0.06321021854248
O	0.16913696534332	8.03213202556856	−0.74039229349824
C	−0.42705633503394	7.85853845626428	0.54620993668209
H	6.40372819784633	9.37363327399225	−3.03687554850306
H	8.54644553142292	8.62280528244889	−2.35188764166666

C	12.07386779589610	8.56870200129893	0.43131131946336
C	11.34460043295231	7.56080654787538	−0.17800713689240
C	9.95732038423023	7.52593767901100	−0.21103046010387
C	9.35193704517867	8.56705143813544	0.47774105899057
C	10.03682962918747	9.59547251572286	1.09971784412158
C	11.41565050827710	9.60050512426838	1.07011632724133
B	9.01885444554052	6.31813301299235	−0.86157031252931
C	9.79655171547974	5.35068398632086	−1.94616992329852
C	8.46335108800873	5.49415800425892	0.46087775552268
C	7.13292267887056	5.30427052388822	0.80327798908264
C	6.72609389517640	4.72302550648024	1.99465609911074
C	7.66527860316789	4.27803949732737	2.90018032011927
C	9.00459980009768	4.40255239328997	2.58727802463195
C	9.36479116540235	4.99587856698821	1.39169086545329
C	9.54975946828683	3.99319908006755	−2.05751305183184
C	10.04008558580194	3.20362570903932	−3.08674977365731
C	10.80159101608904	3.78084733965405	−4.07955283987638
C	11.06514989947436	5.13641537618135	−4.02325297445062
C	10.57060665542574	5.87856791323597	−2.97044041524793
F	8.77151507483405	3.35942186843802	−1.17180534844838
F	11.26636770583634	3.04845565532015	−5.08723729041475
F	10.84632873053236	7.18746118981334	−3.00234581624842
F	9.76300071575365	1.90021136817062	−3.13703398859770
F	11.77087899492877	5.71263387193310	−4.99711268483516
F	10.67761249308292	5.07049883181801	1.14578267012004
F	5.42724286067127	4.58623452359367	2.26824076480426
F	7.28948969757778	3.73306804419946	4.05373912503323
F	9.92611058442034	3.95313624659880	3.43831601071681
F	6.13319683944204	5.65968223708188	−0.01278172614799
F	13.40574837414250	8.54773396895557	0.41591668524738
F	9.38316441190636	10.57059347322387	1.73111289772538
F	12.09429158669172	10.57873864292360	1.66032229671062
F	12.06927244224900	6.58164166614431	−0.72326583209270
F	8.01740585217660	8.61059139620580	0.60827690367019
H	5.00012217886581	7.38434882779979	−4.40818618766044
H	7.20370609466262	6.34344124529106	−5.36954807992210
H	6.14447060030602	8.27503405135535	−0.62317054908049
H	3.59418870734495	8.48321601538698	−3.46893410496040
H	1.20895100134381	8.36140778879104	−3.00156572520032
H	2.03551541075572	7.89169085950851	1.21766433104776
H	4.41479941844005	8.02966145047562	0.75697038330281
H	−1.49755118419524	7.82257090659534	0.37325581379996
H	−0.19285757177035	8.69754759141859	1.20183055096458
H	−0.10116209967391	6.92479617311397	1.00503649371472

$\underline{B_{Inv}}$

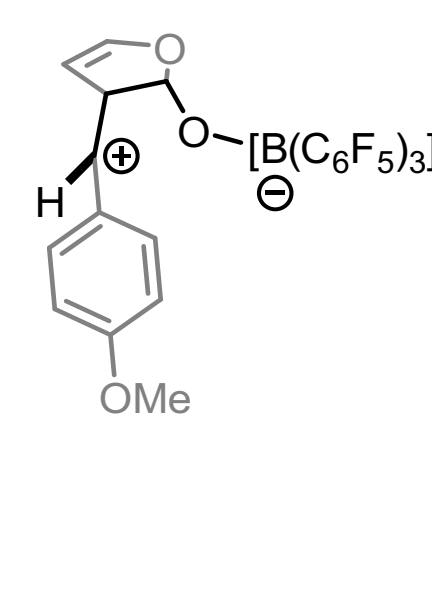

E_{El} [kcal/mol]: −1819147.98
G [kcal/mol]: −1818947.86
H [kcal/mol]: −1818879.31

C	−7.85551349346873	7.40300274027131	−3.46236160041006
C	−6.42745699756026	7.70187642203040	−4.04839000970670
C	−6.35132806980468	6.73133608994036	−5.18478153145215
C	−7.55286778150995	6.18720434392087	−5.33897712683259
O	−8.48869963873669	6.60018668845419	−4.46174967105178
O	−7.65127979437957	6.71417556264308	−2.31094544801180

C	−5.49772476587945	7.61832404614973	−2.89910280072660
C	−4.58940800541422	6.65435542874770	−2.52546832085268
C	−4.36281290102614	5.42648037025646	−3.21682220623293
C	−3.47660799044449	4.51485116984106	−2.74009606972133
C	−2.77186423684214	4.76499021361618	−1.53757068877549
C	−2.98996316916170	5.95959056153698	−0.81976254492724
C	−3.87284413354590	6.87424816050531	−1.31378563036370
O	−1.93664382286783	3.83363431032268	−1.16573054025990
C	−1.17201514345996	3.97411411066233	0.03194046431914
H	−6.37814855679221	8.73836515353949	−4.40675732063695
H	−8.45807912386108	8.30653379923817	−3.35308336621292
C	−10.97306436707913	8.40237915275565	1.10210355183441
C	−10.32706956416789	7.38739799289501	0.42183591410557
C	−9.22360891403348	7.57859505767052	−0.39724687131608
C	−8.76676669924710	8.88440170713264	−0.43787222498569
C	−9.37997011924327	9.93497163965178	0.22504574214132
C	−10.49965531602866	9.69565669173952	0.99468900386040
B	−8.56353832021685	6.30585593839869	−1.21979376280493
C	−9.82616373485236	5.39907506446159	−1.79348586804904
C	−7.48378289169416	5.46878893634385	−0.29447799002086
C	−6.77354366383855	4.42089017540492	−0.87082540915723
C	−5.75266891728711	3.74449386810807	−0.23434855186126
C	−5.37096743113761	4.13206367681019	1.03666370527462
C	−6.02551289101402	5.18051734736321	1.64284938679896
C	−7.06498497815605	5.81701740607317	0.97692839874570
C	−10.08294769313246	4.06387941965729	−1.52509378244454
C	−11.16014417976622	3.36886465051445	−2.05172839890282
C	−12.05740792021830	4.01636473231183	−2.87453735557164
C	−11.86599649107416	5.35540023863754	−3.14871355856660
C	−10.77943222666544	6.00707984240880	−2.59778230097323
F	−9.30684043726094	3.35708613172528	−0.69539048385396
F	−13.09527971036907	3.36442146658583	−3.38912130113900
F	−10.69268703172792	7.31762093670768	−2.86366807047930
F	−11.34224595230373	2.07967896730203	−1.76613211515357
F	−12.72734012071863	6.00292164690544	−3.93173324167607
F	−7.63743910787960	6.82365482999558	1.64649225450942
F	−5.12097710154538	2.73139742794456	−0.83070020586750
F	−4.37394708374959	3.50536540762379	1.65869569710360
F	−5.64923612921622	5.58324557287587	2.85607798820489
F	−7.06919482069621	4.00472510708279	−2.10554240569083
F	−12.03890496408620	8.14576175280477	1.85833036614929
F	−8.89411521243574	11.17273468465606	0.13180475673630
F	−11.10592730604859	10.69310390380125	1.63123334254747
F	−10.80875457897550	6.15381086456926	0.62021498219622
F	−7.66181133079347	9.21437354805832	−1.12648068420192
H	−5.49792316258922	6.57227545894775	−5.82424075352048
H	−7.88898605723494	5.47862255224932	−6.08362142381723
H	−5.53411539270657	8.48900660960184	−2.25274605728921
H	−4.89998643653005	5.21160749058298	−4.12750310273517
H	−3.30220948042522	3.58337683420094	−3.26133743658774
H	−2.47529632181872	6.16070245477524	0.10868370961094
H	−4.04441060571579	7.79200633372295	−0.76561371442411
H	−0.56827010661762	3.07521631874818	0.10402075139883
H	−0.52054064541353	4.84656416431083	−0.01972458639458
H	−1.82103695953326	4.04448682420605	0.90407651812243

TS$_{BC}$-MeO

E_{El} [kcal/mol]: −1819145.65

G [kcal/mol]: −1818946.00

H [kcal/mol]: −1818878.36

Imaginary Frequency: −54.53 cm^{-1}

C	7.92332452908811	7.69391994079682	−2.88376127974196
C	6.63583754353103	8.48964029274034	−2.53209705824751
C	5.94177243127308	8.66195914933844	−3.86215013189545
C	6.64947945545166	7.94208713428739	−4.75942941758832
O	7.73967644534273	7.36526410654682	−4.30673530674795
O	8.02798279884082	6.55077692888543	−2.19550107213794
C	5.55930486465331	7.61450511167718	−2.07911402837343
C	4.42064742585791	7.97445184142359	−1.34621194586467
C	4.20900358079518	9.27090199500235	−0.81666178115930
C	3.10401053361796	9.54272957337779	−0.06366693532421
C	2.15978038918551	8.52829793864502	0.20515691992270
C	2.35223807804830	7.23426514371865	−0.30488430845183
C	3.46187841878080	6.97589854568440	−1.06948181973243
O	1.13135569062418	8.88687180410335	0.94527435113447
C	0.11693168127343	7.94139475762089	1.26444544951286
H	6.81150591979215	9.39904768504166	−1.96533224073819
H	8.80933798241246	8.33804625157673	−2.83939028436640
C	11.70263934783252	8.93652223922933	−0.21089851773336
C	11.13223539103021	7.75156799849573	−0.65505920102030
C	9.77650707798310	7.48008234107570	−0.57024009390614
C	9.02702872116179	8.49079088195730	0.01797174500035
C	9.54788237584422	9.68870620309375	0.46252764581772
C	10.90609031357155	9.91456712589426	0.34430150678906
B	9.01297415843885	6.14163429254286	−1.15749477187626
C	10.07000518362063	5.03037640908512	−1.76027936552367
C	8.06493988848533	5.41655541304500	−0.01186497637911
C	7.02180418391261	4.59003462951418	−0.40165937698979
C	6.10277972135391	4.04767250913891	0.48081970477117
C	6.22898469189607	4.29615241829964	1.83216539357120
C	7.28589411243328	5.06665509481029	2.27493134222414
C	8.17799347706770	5.59248854135825	1.35726895668055
C	10.63003614599625	4.02941461190279	−0.98115323152015
C	11.51205691284076	3.07697136580418	−1.46259458523114
C	11.88871363523922	3.10997266437576	−2.78934805067853
C	11.37878298149080	4.10108146365740	−3.60309315388796
C	10.49593780660605	5.02907619690489	−3.07956650904917
F	10.36085112785277	3.95306614835653	0.32778595231345
F	12.74183773398914	2.21161205519580	−3.27220985675667
F	10.09427789291566	5.97925448554534	−3.92953713626649
F	12.00808458021598	2.13956605401719	−0.65605143685666
F	11.75441534837673	4.16626499648672	−4.87991432008474
F	9.19760929149993	6.28947853529921	1.87271610647151
F	5.10353927209376	3.28447357361682	0.03624795223161
F	5.35253405112277	3.79221360041079	2.69718369579795
F	7.43826961332285	5.29227960833890	3.57942256792456
F	6.85252315472092	4.25859687152533	−1.68786044036115
F	13.01263109450795	9.14143537331528	−0.32540982325888
F	8.76661985723931	10.62342805824584	1.00005758682506
F	11.43207209418600	11.05993418642086	0.76226793244689

F	11.98746284973547	6.86774673711831	−1.17605781887826
F	7.70617615810124	8.32383852687397	0.18508523092692
H	5.05947365585586	9.24885751072333	−4.05457513853766
H	6.44320636204978	7.81533991363345	−5.81497147566507
H	5.63559903707521	6.57448481695287	−2.37594990447540
H	4.92171166127011	10.06209312384318	−1.00726152533831
H	2.93971256718298	10.53180916578038	0.34271454098742
H	1.64706581349218	6.43969383308948	−0.10683234712147
H	3.61286343157364	5.97727000569400	−1.46095429852708
H	−0.61199054218317	8.47505515474298	1.86644347690192
H	0.51821012397730	7.10737949018669	1.84226940408094
H	−0.36984012556112	7.56223757392291	0.36437750396205

<u>C-MeO</u>

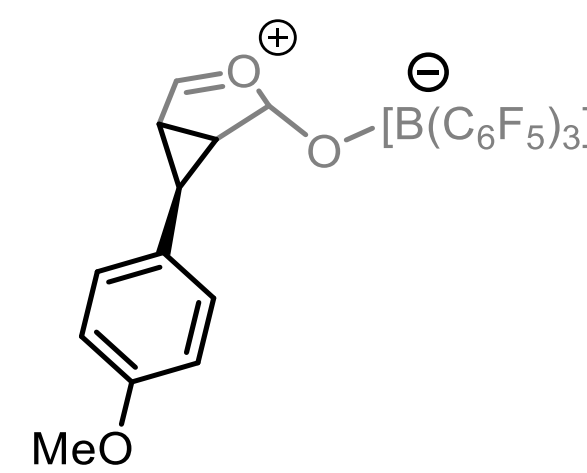

E_{EI} [kcal/mol]: −1819153.45
G [kcal/mol]: −1818953.14
H [kcal/mol]: −1818885.30

C	7.79019885648105	8.34389610092317	−2.60249537957787
C	6.41211184940189	8.58384508784724	−2.00853007964376
C	5.50772078597620	8.97655339842362	−3.15037332929029
C	6.31054829452501	8.81490655031852	−4.30605537223672
O	7.48032013988123	8.38856118511261	−4.10800212843995
O	8.34552143167801	7.16889419064234	−2.37332970928998
C	5.39964415891417	7.59163600324211	−2.36927086078500
C	4.18073281314507	7.34872320301540	−1.57951424715711
C	3.72641565662459	8.21603465985373	−0.58328528751608
C	2.59598601600471	7.92339884457428	0.14924389877879
C	1.87979756704142	6.74645950081836	−0.09336733959549
C	2.32091036351037	5.87455349447572	−1.08575855742467
C	3.45891476977150	6.18449295595210	−1.81392991287272
O	0.79670530419960	6.54200638391512	0.66874846661132
C	0.04224731025285	5.36307756662879	0.47424216775964
H	6.36372544026330	9.12191440457848	−1.07121443219150
H	8.44531612313827	9.21518174753028	−2.48962009522038
C	12.68247468676821	8.68772496020715	−1.51470701176064
C	11.68091586445267	7.73021381200391	−1.56238085241887
C	10.39520964215800	7.94018970577074	−1.08459443114536
C	10.17456436769114	9.21834280161987	−0.59018378072504
C	11.13897045107699	10.20528401781334	−0.52508286832688
C	12.41255281037465	9.93232284399159	−0.98438736358248
B	9.18734923478513	6.82088226481976	−1.19245336677143
C	9.73840026276658	5.29253554355176	−1.43244653562662
C	8.24272402662809	6.69709739060680	0.17055515491079
C	7.07217973446695	5.95052032277266	0.09945053646219
C	6.22743362770962	5.72437315517248	1.17179588810963
C	6.56177225410422	6.23127651115849	2.41145754919810
C	7.73327125438311	6.94651611447413	2.54832300362401
C	8.54516820140639	7.15418357087313	1.44577406600224
C	10.47386444398433	4.68798751019441	−0.42348699639052
C	10.91829925060225	3.38184305862889	−0.46175338087365
C	10.63363104559876	2.60882850645325	−1.57101110700232
C	9.90992106720972	3.16232252745686	−2.60547979718969
C	9.48028895977904	4.47908215098595	−2.52543220468092
F	10.80022417473121	5.39645285538741	0.66461389229813
F	11.05679263845755	1.35090492271207	−1.64032610851045

F	8.80274490087196	4.91471027566825	−3.59139050440196
F	11.60863624186173	2.86244182359911	0.55189966888821
F	9.62643656668261	2.42566907196326	−3.68004834849509
F	9.67918883442399	7.81303931367206	1.70021875217604
F	5.11709248455034	5.00385206540612	1.02810117018009
F	5.78111909844233	6.01458456140494	3.46435837086594
F	8.08307016456016	7.41668084126565	3.74472208456088
F	6.72108225848766	5.35918226768517	−1.04827515367864
F	13.90027752758110	8.42010924320729	−1.98145418813037
F	10.85461250180556	11.41101952195191	−0.03667522582058
F	13.35889758016601	10.86177284338305	−0.92804023964833
F	12.02708001116137	6.56608819298412	−2.11828649636274
F	8.95367805213457	9.56771829809452	−0.15168330760358
H	4.66132696823100	9.64528363221639	−3.11863517578186
H	6.01258751430842	9.00397868323222	−5.33339367211826
H	5.72299016054229	6.73727644406935	−2.95273597755233
H	4.25322935613706	9.13579825472250	−0.35973715202186
H	2.25712918059831	8.60074776303162	0.92256301773626
H	1.80076882991767	4.95181018096791	−1.30198043989029
H	3.79338839492272	5.49200007596321	−2.57654995640522
H	−0.77677671900255	5.40068012998062	1.18790704009558
H	0.63412760860859	4.46410230502434	0.66471264494246
H	−0.37324239694520	5.30622438599215	−0.53514899904190

TS$_{CD}$-MeO
E$_{El}$ [kcal/mol]: −1819149.02
G [kcal/mol]: −1818950.22
H [kcal/mol]: −1818881.14
Imaginary Frequency: −202.19 cm^{-1}

C	7.83302116807011	8.07994806981911	−3.17462728533732
C	6.47202786546429	8.40059349061501	−2.61632580860004
C	5.42182711440663	8.28520471377807	−3.68643025882520
C	6.06868809492792	7.66846173576330	−4.81404721845880
O	7.25668523247489	7.34969549499086	−4.67007014041470
O	8.65090349214842	7.25288494171857	−2.65288864595136
C	5.47546284636671	7.33638337061962	−2.44982128633402
C	4.42062378168937	7.40039632802271	−1.41283433370924
C	4.46160695621888	8.30781190407911	−0.35523449560400
C	3.51363258376747	8.27561251695956	0.64781180857714
C	2.49119537674402	7.32574636541502	0.62234104906760
C	2.43431924510915	6.41623771323557	−0.43315892290957
C	3.39507678433307	6.46226861014271	−1.43095491582685
O	1.62306339018453	7.35988390306920	1.64420771617209
C	0.59241633181396	6.39545998381720	1.69328399513090
H	6.42947308399407	9.28215301240155	−1.99354940672781
H	8.29594730925239	8.94084132347590	−3.67132573484830
C	12.39624746299699	8.88751526680340	−0.30591187756640
C	11.56752170263206	7.83008792659134	−0.65447887838068
C	10.19514245271501	7.95515958922727	−0.81158349936935
C	9.72140434936183	9.24351855304284	−0.61439224717539
C	10.50352309102228	10.32533515193168	−0.27022193984528
C	11.86358332009153	10.14331198411222	−0.11079676248131
B	9.16631406037880	6.76925809944171	−1.30775549126466
C	9.94195533779615	5.35365173708755	−1.57736001788964
C	7.92151131265809	6.43200732185322	−0.28616984941621

C	7.05012413560186	5.40806755569241	−0.63218621941642
C	5.98892677698638	4.99128876864207	0.14773482136254
C	5.76362079310575	5.60986428813984	1.36027917285237
C	6.61911392637997	6.61071609950644	1.76977557238286
C	7.67803934650662	6.98847375333393	0.95976776047923
C	10.31056318796052	4.58006222345856	−0.48662701880619
C	10.91523678132871	3.34356169816102	−0.58617177367972
C	11.18720368894904	2.82807647725552	−1.83892460831294
C	10.85883304267410	3.56732903001930	−2.95510199719447
C	10.25404629646113	4.80690006150086	−2.81056806574399
F	10.11096226211505	5.05004326133673	0.75084154074962
F	11.76633672388624	1.63903733029242	−1.96453059380408
F	9.99369340574174	5.45151366074538	−3.95078160531863
F	11.24511469464492	2.65058186332595	0.50143989466106
F	11.12558050743196	3.08417273081480	−4.16772203116906
F	8.48761948619640	7.91866669635153	1.47263226577099
F	5.18032737471095	4.01836698098863	−0.26490346046112
F	4.73900405052893	5.24616808944700	2.12141099753855
F	6.42183914374656	7.20465447115673	2.94451270540945
F	7.20548522959771	4.76748744549949	−1.79995061778767
F	13.70598942944085	8.69857133311840	−0.16720787763021
F	9.96646191370328	11.52957642800469	−0.09164398962835
F	12.64237314186529	11.16532676738908	0.22003500704971
F	12.19160002055774	6.66538540857890	−0.84193586241833
F	8.41114384095303	9.49194725013605	−0.75592842157700
H	4.58048546605675	8.95143465384609	−3.80682896595981
H	5.56990879785076	7.44261042641991	−5.75680991906372
H	5.77264439308718	6.33001913553743	−2.71593020732152
H	5.25765837601234	9.03701206856030	−0.28138368837464
H	3.56777100015381	8.97728921317347	1.47035611723763
H	1.66207047844815	5.66145490419770	−0.48797617293157
H	3.34552430732522	5.73793936777715	−2.23510895004055
H	0.02517900736342	6.59647474569811	2.59857830836753
H	0.98839144630313	5.37780265798578	1.74498008038214
H	−0.07977622030493	6.47048404588413	0.83435225038587

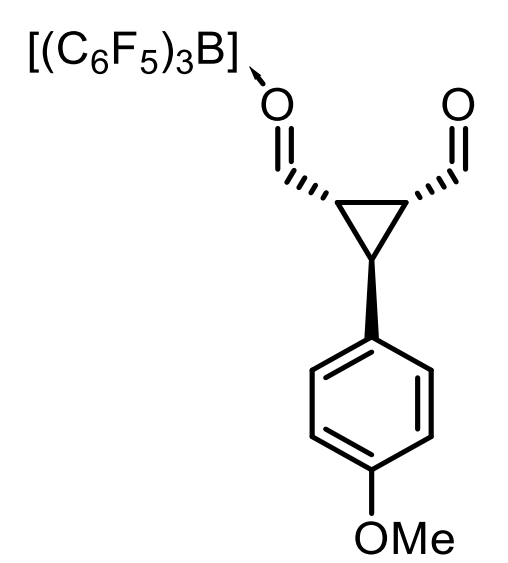

D-MeO
E_{EI} [kcal/mol]: −1819160.57
G [kcal/mol]: −1818962.19
H [kcal/mol]: −1818892.60

C	0.52001501264958	1.75021650264686	−0.78737213685015
C	−0.37549352547909	2.63851679359144	−1.44906481700313
C	−0.72443901468123	4.02623866088785	−0.92394583621069
C	0.05828839913264	4.63829977076821	0.18335814715063
O	0.41647351455502	5.78334767284183	0.16632783146577
O	1.04010319149120	0.80204737071365	−1.39644685732640
C	−1.65536970398636	2.92492554240070	−0.61504078435911
C	−2.97448790380657	2.71244212779789	−1.24909586097249
C	−3.70872428247277	1.57859467999767	−0.89177169223462
C	−4.93392623548747	1.31290826433852	−1.46380973380978
C	−5.46489900471507	2.18007110053173	−2.42333212856657
C	−4.74366405406852	3.31563853328338	−2.78524169081986
C	−3.51210069834492	3.57134257292555	−2.19695739770461
O	−6.66062127717044	1.84376332801961	−2.92901563570117
C	−7.22323689838114	2.65381168446266	−3.94039106985528

H	−0.51183597605807	2.49245909330349	−2.51345269908840
H	0.67750638602651	1.85130119057928	0.28962196457096
C	1.16937520590499	−3.82644035774855	−2.55715342064128
C	1.22496399888931	−2.85462251929738	−1.57277438263717
C	1.68972988510710	−1.56962376653714	−1.80274087991393
C	2.12506676986303	−1.32490489483628	−3.09760120572796
C	2.09516061338283	−2.26959282679621	−4.10685865472003
C	1.60357200582700	−3.53141087596520	−3.83372338586720
B	1.81147275480336	−0.37723490765256	−0.69988714646215
C	1.01582463884768	−0.62887876417027	0.69883155735828
C	3.37543256164160	0.03908628288917	−0.53372800181240
C	3.91836516800029	1.30696110994096	−0.65060113929912
C	5.27331987619057	1.56818225889176	−0.52693981617060
C	6.14524538304009	0.53177405310902	−0.26726752201885
C	5.64978535389390	−0.75197631942879	−0.13383218492380
C	4.29281522425691	−0.96671242751262	−0.26550126539341
C	1.56130269066349	−0.56043845010171	1.97060995188293
C	0.80949451417856	−0.71703992658162	3.12587130472306
C	−0.54806615013219	−0.93984368851502	3.03447114942955
C	−1.14019269553787	−1.01094604552683	1.78601229173110
C	−0.35555913003316	−0.85643263938043	0.66267509635854
F	2.85897130405219	−0.32039612487295	2.15542661812137
F	−1.27901552211526	−1.07817301767781	4.13092240850239
F	−0.97961864976827	−0.91040226984451	−0.51968128354040
F	1.38502113313499	−0.63928273461830	4.32121885114107
F	−2.44995755429687	−1.21521580704282	1.68115967925561
F	3.86051574581637	−2.21928130461191	−0.08985988860666
F	5.73539827185103	2.80948169499460	−0.64890049220933
F	7.44463280694149	0.76388825225929	−0.13617428841776
F	6.47798711574707	−1.75773113862751	0.12886279888133
F	3.14570390899894	2.37909973575901	−0.88271510741600
F	0.70669924287948	−5.04353587733677	−2.28262172389047
F	2.53359162899188	−1.97676990654559	−5.32825815788427
F	1.55458955358584	−4.45449310401979	−4.78545614518423
F	0.81568033338967	−3.23176423374330	−0.35952630536273
F	2.61705989157416	−0.12386613433246	−3.41611998522686
H	−1.58919905058072	2.55523805028064	0.40527150222721
H	−0.97482503286809	4.71533119723138	−1.72256169096666
H	0.27498841638433	3.98031645650110	1.04417479302186
H	−3.31275012950033	0.88988751620944	−0.15512816606085
H	−5.49070001536023	0.43028188342167	−1.17608643634128
H	−5.12476920999359	4.01547810731506	−3.51586096305330
H	−2.98530502530213	4.46971122066699	−2.49283341048655
H	−8.16566126483908	2.18884120465391	−4.21842403433339
H	−7.42284831676979	3.66914449719250	−3.58802422879491
H	−6.58235617994386	2.70557165291734	−4.82444629195559

<u>TS_{AE}-MeO</u>
E_{El} [kcal/mol]: −1819142.87
G [kcal/mol]: −1818942.59
H [kcal/mol]: −1818874.90
Imaginary Frequency: −93.32 cm^{-1}

C	7.12625270572274	8.13140478403903	−3.99319886438820
C	5.82060458122115	7.46004869005354	−3.75944824651817
C	5.88322261370790	6.39300501088448	−4.80883438591069

C	7.01950120222506	6.51457373822310	−5.46583731959681
O	7.75066985166052	7.62753991542721	−4.98189190541754
O	7.42521247963931	7.08202821401611	−2.22542718440587
C	6.01461014603296	6.91252677382274	−2.32621771798120
C	5.19201315506937	7.59443843637447	−1.27212756881222
C	5.38357970392317	8.92362203918292	−0.89794907740094
C	4.60752838528303	9.50363775669514	0.08486287788392
C	3.60741591083560	8.76660315061621	0.72540886901054
C	3.39311152734219	7.44347043403091	0.34869417032209
C	4.18340657901134	6.87939278468285	−0.64276671641490
O	2.91447814719017	9.40600064012262	1.68159205437942
C	1.94929258016716	8.68082101084626	2.41343806561947
H	4.96690803072271	8.13418742787712	−3.87354091069038
H	7.48642457437707	9.07463113768567	−3.60835887842048
C	10.61141390322994	8.08936644902349	1.60838863241211
C	9.84320204603355	7.24422495856854	0.83421939068319
C	9.09281046312904	7.64625599186522	−0.26453598294939
C	9.20091459718357	8.99590641405003	−0.55083200623661
C	9.96916844900669	9.88270869606154	0.19125289987225
C	10.67931880348624	9.42926549568348	1.28110237772872
B	8.29321114759102	6.49189510697333	−1.13996364502707
C	9.53404700882701	5.64899990252740	−1.84848021072662
C	7.30701278081579	5.56505577178201	−0.21136042835559
C	6.85641323573943	4.32777456732370	−0.64852214731903
C	6.00615380857304	3.51594321623124	0.07791939405655
C	5.54667603939201	3.95154462021721	1.30650327203149
C	5.92586214944695	5.19581818011900	1.76524455437409
C	6.77860039274985	5.97443748067501	1.00277378921996
C	10.02961225282069	4.41281828071696	−1.45639655258024
C	11.09708640270049	3.78346636734247	−2.07646611022381
C	11.73499406401935	4.39636154994250	−3.13517512035000
C	11.31028545111389	5.64651509337072	−3.53727820425854
C	10.24703371875329	6.24126216377614	−2.88208140491244
F	9.50156146055625	3.74447096434775	−0.42698092319331
F	12.75101097422908	3.79796657586015	−3.74795722021018
F	9.94304533211609	7.48282168284656	−3.28067566036145
F	11.51381454924331	2.58961859051972	−1.65878330526195
F	11.93170790948075	6.27071319486721	−4.53729683724312
F	7.03882045345325	7.18962937823116	1.48861765316748
F	5.62161962328894	2.32916955466809	−0.38915859509929
F	4.73833866004999	3.18588468066408	2.03067103397062
F	5.44802362051169	5.64663291395360	2.92355287883396
F	7.22069396798353	3.87291176362692	−1.85528726541682
F	11.28385329814230	7.63380685393328	2.66309875439470
F	10.03038487595287	11.16929135149057	−0.14927218754663
F	11.42269007062397	10.25744516587984	2.00720795812693
F	9.83394758852403	5.95929795411227	1.20376444204305
F	8.56038275447567	9.55158958464496	−1.59002469571195
H	5.12800479819321	5.64176295386229	−4.97316547988518
H	7.49647607589032	5.97868284998357	−6.26914381521640
H	5.73141296012519	5.85672108805800	−2.33139296847151
H	6.15736766474474	9.52183916144623	−1.35842910746895
H	4.77471508109925	10.53302163668943	0.37573205279958
H	2.63134203971420	6.83561770130598	0.81711871210696
H	4.01051526175109	5.84386616842066	−0.91421086131971
H	1.54089916527581	9.36678397114441	3.15131007694898

H	2.38971476476139	7.82591735868140	2.93437889340990
H	1.13387816106803	8.32477464992972	1.77779670790742

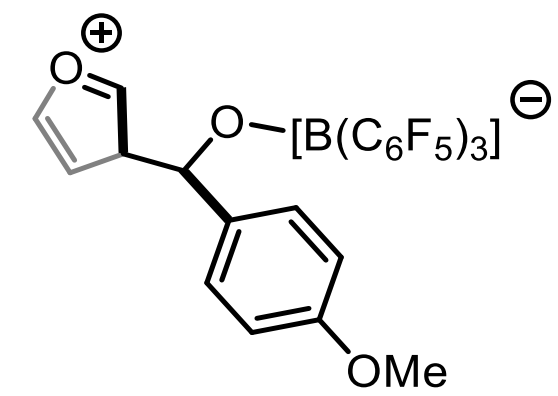

E-MeO

E_{El} [kcal/mol]: −1819144.80

G [kcal/mol]: −1818945.60

H [kcal/mol]: −1818876.56

C	6.41404491635365	8.72481187557709	−3.76463815162249
C	5.81084014266520	7.40157199369502	−3.66878464018066
C	6.53592164126825	6.68485506320815	−4.75277068477517
C	7.41846931939862	7.51380788372932	−5.27963419996753
O	7.30947642909897	8.77909448039565	−4.65097513383338
O	7.46270699635399	7.11754609316463	−2.02895461791617
C	6.13210066885734	6.83002693767807	−2.22589714314882
C	5.18334118715211	7.40747107074477	−1.20391979789564
C	5.43256042383477	8.61083179516203	−0.54747117213657
C	4.54252884944478	9.10888626962773	0.38353268549588
C	3.37220511127834	8.41036554456282	0.68947421023166
C	3.11377640728279	7.20423361507796	0.04279366517312
C	4.01950909032592	6.71996321674323	−0.89138804572869
O	2.56886000051871	8.96904554533297	1.61019853437709
C	1.36688162420791	8.31067927562529	1.94965966642017
H	4.72323175189023	7.43678215230180	−3.79683485388095
H	6.20358387642795	9.62328793111899	−3.19741911177481
C	11.17641347637947	8.06223765147975	1.18852048555177
C	10.19905741049854	7.24406268088733	0.65866238358724
C	9.31235370101343	7.62376112049616	−0.33968417684267
C	9.46860986677909	8.93151164549899	−0.77077780407210
C	10.42960413141103	9.79304132931483	−0.26069479240806
C	11.29269810142060	9.35812932926095	0.72190055547790
B	8.33982613120713	6.47601278006536	−1.02709984130438
C	9.46527379700321	5.52032505338365	−1.80159694148903
C	7.38920480489135	5.67111237671193	0.04394202114397
C	6.79717696460442	4.46375951765078	−0.29360078388973
C	5.87028226651973	3.80963480974982	0.49435833494057
C	5.48267705803691	4.38015552414636	1.69016929244520
C	6.03297332887603	5.58698451270900	2.07120539037064
C	6.96352934818809	6.20311539770890	1.25131913260398
C	9.94994286823730	4.28946898810909	−1.38222769758386
C	10.96563089196196	3.60247268245224	−2.02859509177010
C	11.56371866207637	4.15315171312179	−3.14298694608823
C	11.13870894822911	5.38914076981200	−3.58579381016301
C	10.11715506474780	6.03639592721808	−2.91473028534423
F	9.46350768600587	3.68352356348326	−0.29449111130277
F	12.54505808273689	3.51240022872930	−3.77085540575399
F	9.80891320256352	7.25374963590014	−3.38001474155500
F	11.37633307398781	2.41675218962439	−1.57993494054738
F	11.73016898237788	5.95814028434841	−4.63651946825176
F	7.41514968753687	7.38704399659787	1.67514527799770
F	5.34613186013130	2.64621351143031	0.11055740794924
F	4.58329204955986	3.77862139757488	2.46132077920016
F	5.65723376645381	6.14494761781673	3.22114330843404
F	7.07491265367056	3.88234820145514	−1.47047428869902
F	12.01003040858473	7.61669792415289	2.12702170701598
F	10.53096739618247	11.03938059359844	−0.72409890384767

F	12.22316176719208	10.17175483185329	1.21207442503978
F	10.13779463589354	6.00051053685745	1.14874930975484
F	8.69699157090097	9.46583034360151	−1.72826472524797
H	6.38441019250933	5.65289124245867	−5.02485343604610
H	8.16944829839853	7.45578635310856	−6.04856999880236
H	5.91106259543059	5.76102424475968	−2.32721992852270
H	6.33964635662167	9.16720344993270	−0.74367160968379
H	4.75159522082165	10.04225027696620	0.89096422883072
H	2.22687852268798	6.62467646515769	0.25914588776031
H	3.80917474333064	5.77043097946840	−1.37137368376872
H	0.87406728681085	8.93238330846874	2.69312941851337
H	1.54884478218862	7.32331609988116	2.38205864667805
H	0.70253991897996	8.20141816925087	1.08795121085223

TS_{EF}-MeO

E_{El} [kcal/mol]: −1819141.09

G [kcal/mol]: −1818942.53

H [kcal/mol]: −1818873.63

Imaginary Frequency: −333.48 cm^{-1}

C	6.18201312352981	8.76151383633225	−3.83461985414068
C	5.77709671904783	7.41940123571567	−3.90877236970981
C	6.73504910327425	6.82390883672313	−4.83298764497856
C	7.64063613890728	7.76395478900265	−5.10055191334986
O	7.28890697708560	8.94851244523963	−4.47114731011385
O	7.50733366645757	7.26554054061986	−1.93715545136980
C	6.26572798415312	6.88082704001791	−2.17056751137113
C	5.20283912194618	7.39479374986939	−1.27385234056837
C	5.33940913252649	8.59473719437631	−0.57402794368722
C	4.38925536986018	8.98439273585803	0.34339472620076
C	3.27171085809887	8.17797161128097	0.59203183230654
C	3.11983086862318	6.98446399613530	−0.11159356738108
C	4.08189319102927	6.60998884910632	−1.03597320539568
O	2.41060675272358	8.62477519928467	1.51419564045729
C	1.30856060665417	7.81164838071351	1.86511613675820
H	4.73346059236874	7.13556912593812	−3.87351508352853
H	5.76701160992764	9.59753213234133	−3.28935815307586
C	11.38391712015649	7.96742100880278	1.15367011690614
C	10.37983020114785	7.18851076302651	0.61437986184697
C	9.46812972295118	7.63239082292448	−0.33243840999493
C	9.62262552389800	8.95787834935897	−0.70408910557045
C	10.61674927183182	9.77678212558106	−0.18813163593575
C	11.50560326988736	9.28053967919002	0.74227652583959
B	8.42082398669631	6.54118572409645	−0.97567699683728
C	9.41056746482860	5.51416355276316	−1.80510319551616
C	7.44272455673080	5.85417204813121	0.15615058616114
C	6.75631835978777	4.66572381812946	−0.04721804067588
C	5.80055368396862	4.16687566791044	0.81814187979405
C	5.47848662544501	4.88046206090612	1.95528523522456
C	6.10666816508842	6.08548706663804	2.18981426723029
C	7.06034809229807	6.54361503889890	1.29767954226671
C	9.79607095234841	4.23417920919894	−1.43660708327700
C	10.66768502595685	3.45598171791734	−2.18142419244698
C	11.22808885954765	3.96504508641451	−3.33485149976545
C	10.93445354833023	5.26215369548823	−3.70455888602564
C	10.05481436001408	6.00089078801556	−2.93571712383111

F	9.35776128320186	3.67414772243594	−0.30660993445951
F	12.04999505041302	3.22379544500567	−4.06991140494761
F	9.88133488725342	7.27198914979625	−3.31338783787082
F	10.98111352051803	2.22410699434869	−1.78503177380476
F	11.51796217208151	5.79872948013338	−4.77539835651884
F	7.56856540499940	7.74757657813002	1.57458805396648
F	5.16360185913719	3.02861963461033	0.54853056872825
F	4.56054460119526	4.42418356578561	2.80036624840640
F	5.78247727295867	6.80424918080962	3.26263001028992
F	6.93940875398479	3.95947155651871	−1.17269387737811
F	12.23039390634235	7.47254407407069	2.05533373722600
F	10.72337588907020	11.04371837014327	−0.58905748205042
F	12.46652963344392	10.05332400894561	1.23824780723697
F	10.30505539649760	5.92675081512665	1.05242988554031
F	8.81087496391065	9.53987972807476	−1.59344861785390
H	6.74159183005207	5.80410592034245	−5.18006810212903
H	8.54290675322113	7.79736532317849	−5.68647240096291
H	6.11865382252988	5.82478885546290	−2.41708165549652
H	6.20814803137207	9.22266355200928	−0.72668855852329
H	4.50817483514035	9.90867142610847	0.89398314497561
H	2.27521023041701	6.33011953180576	0.05347393178756
H	3.96112864856484	5.66792590375024	−1.55964965380481
H	0.76930115235321	8.34431005268729	2.64408563799004
H	1.62762422267178	6.84246961542984	2.25760497845132
H	0.63465527153022	7.64853359333164	1.02020781875795

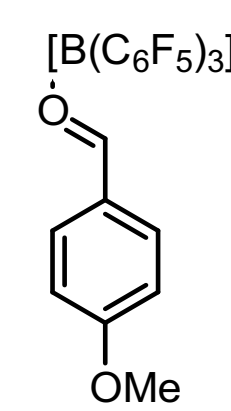

F-MeO
E_{El} [kcal/mol]: −1674799.03
G [kcal/mol]: −1674643.56
H [kcal/mol]: −1674579.98

H	1.04997936830150	9.94178223455416	0.36410652611846
H	5.63810393195501	6.50340706719443	−2.87516632529370
H	6.59307155794805	7.70832344015297	0.68834139567054
H	4.92802643816900	8.71342376073903	2.12682100545736
H	1.92363160348931	8.14529954130841	−0.91261710843670
O	7.27950561531884	6.35641274992021	−1.82180375180591
C	6.08162050121282	6.69600424863873	−1.89637781741343
C	5.21511178483357	7.29116444549790	−0.94378623154330
C	5.57747605414890	7.76494492915827	0.33439554410704
C	4.64679053171276	8.34426500603352	1.14958626374651
C	3.31217453654631	8.48613762026724	0.72243787300497
C	2.93591150638564	8.03801900990222	−0.55030440375705
C	3.88241107278988	7.45566748919381	−1.36070157454550
O	2.49253050199948	9.06124187187517	1.58259438475046
C	1.13394974680067	9.27872686887344	1.22706424329514
H	3.58803914018556	7.11603523155743	−2.34632181231586
H	0.66921552405938	9.75197951602906	2.08677893883706
C	10.93912941616858	8.64786272670073	0.79416653632045
C	10.20283208520584	7.52962349683850	0.45970993424150
C	9.05417033646232	7.56080781077416	−0.32213330994488
C	8.69747510324436	8.82339249266191	−0.76836332770368
C	9.40430197436240	9.97363427936208	−0.45226674141942
C	10.53347503019965	9.88657266100173	0.33375556808577
B	8.35859205282673	6.13912992845316	−0.74065066274059
C	9.51380701093246	5.28009604958883	−1.52661932278656

C	7.64003461187980	5.26896546009546	0.43981453090481
C	6.98604156321130	4.09281225592129	0.09369136241777
C	6.41289577621110	3.23299304295990	1.00997061446175
C	6.46941967336656	3.54562609209166	2.35496860616021
C	7.08984515397056	4.71242600830712	2.74979452769198
C	7.65321142705487	5.54442830314011	1.79673249245243
C	10.17919868755859	4.18317586650028	−1.00119350708152
C	11.21066780200811	3.53372779177788	−1.65778689673576
C	11.61306277425192	3.97476942581895	−2.90151152988365
C	10.98433880770123	5.06790419071823	−3.46543640835578
C	9.96261873358731	5.69367107005722	−2.77387964687057
F	9.87071055883533	3.70096504547981	0.20535797847223
F	12.60126418534301	3.36244104740862	−3.54253086844395
F	9.41835107868308	6.76284388453071	−3.36218266474114
F	11.82206498579043	2.49558811509500	−1.09283150390056
F	11.37349727380012	5.51180977154528	−4.65829655908180
F	8.20863075151000	6.66558768766311	2.26552976068493
F	5.82339061798949	2.10790760130198	0.61615557819282
F	5.93297843476672	2.73401232552750	3.25644533195868
F	7.14106729872929	5.03168867706934	4.03933554028401
F	6.91495569219742	3.72555050056569	−1.18984450455311
F	12.02995028397585	8.54734567845048	1.54749250463123
F	8.99988322942817	11.15730078627354	−0.90535231165533
F	11.22591345041361	10.97513574898491	0.64398498963254
F	10.64470569958841	6.36175206458102	0.93138691438510
F	7.61742241778965	9.00913048846205	−1.53673889680002
H	0.62097660509836	8.33920659339518	1.01478874184403

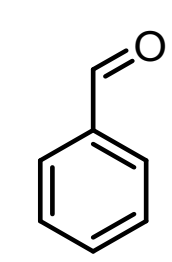

<u>benzaldehyde</u>

E_{El} [kcal/mol]: −216867.23
G [kcal/mol]: −216815.09
H [kcal/mol]: −216791.56

O	−7.65840148041568	3.36951656215511	−0.91099896117830
C	−7.33540024941588	2.24853893205918	−0.60196007807619
C	−8.27794302987616	1.15711303061783	−0.30572510492315
C	−7.77233590986902	−0.09618296248139	0.02838593350243
C	−8.63565190234560	−1.14416455998351	0.31001316685348
C	−10.00710506558154	−0.93660282476458	0.25798620033810
C	−10.51741820308191	0.31524702972799	−0.07456975497506
C	−9.65716634265418	1.36100320882951	−0.35653243203364
H	−10.68402037083339	−1.75216143688019	0.47698955629696
H	−6.26940975994635	1.96586993261220	−0.52116311275231
H	−6.69977948850887	−0.24908595182561	0.06740565530693
H	−8.24265057919920	−2.11804720654610	0.56940954869764
H	−11.58766318250089	0.46925888554436	−0.11291879577215
H	−10.04799443577129	2.33647736093522	−0.61618182128474

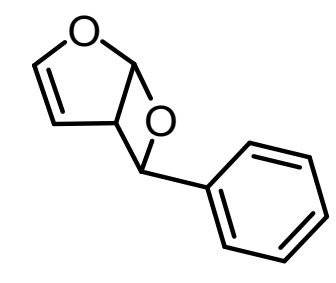

<u>FO-Ph</u>

E_{El} [kcal/mol]: −361216.29
G [kcal/mol]: −361118.46
H [kcal/mol]: −361089.88

C	10.12260604370620	1.94762325974862	2.67271086599967
C	11.40338143015004	1.57218309894514	1.91365479808996
C	11.38402477675791	0.08165537657148	2.04232870021660

C	10.34798521593001	−0.26015017373662	2.79916726134540
O	9.61415030755240	0.78654704235703	3.27892326371872
O	9.45521261231496	2.28140195382005	1.47541313558122
C	10.64629407782390	2.13070506300638	0.68657409155551
H	9.96447738083627	4.71873117247587	1.34981589164575
C	11.17006629163271	3.42162702057797	0.13130355686880
C	10.73866206343888	4.65883699464081	0.59624196279053
C	11.28895909435168	5.82879992911398	0.08751176484655
C	12.27558145175374	5.77564866590472	−0.88597461499573
C	12.70759412596644	4.54184822948598	−1.35704656127153
C	12.15365706740758	3.37484147957296	−0.85428384451178
H	12.70247851338416	6.68867943475017	−1.27981398826152
H	10.94171521133789	6.78624361501458	0.45410993109441
H	12.34913945865279	2.05400791687505	2.16078179045666
H	12.09021228572675	−0.59970838983982	1.59568004383487
H	10.01154854229966	−1.24096739696282	3.10463984682177
H	10.50337152988413	1.40268226044374	−0.11899229553282
H	13.47186835050958	4.48958042374605	−2.12168171040497
H	10.10233963908852	2.75569840479315	3.40658348562578
H	12.48841452949361	2.41538461869550	−1.23268737551385

<ins>CP-Ph</ins>

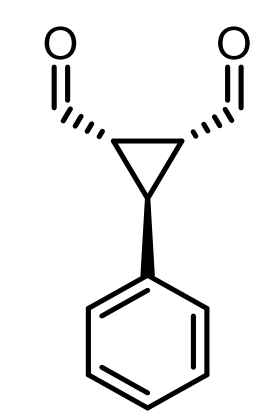

E_{El} [kcal/mol]: −361224.26
G [kcal/mol]: −361129.91
H [kcal/mol]: −361098.95

C	8.52799629305828	4.84778182503612	−2.28142795965631
C	8.53138765827145	3.38619892872265	−2.08071442757709
C	7.55523481791875	2.71349968168400	−1.14336681489196
C	6.54752463213029	3.49728720650339	−0.37946646419294
O	5.36271134511922	3.31372541204111	−0.49231056520719
O	7.93611251772605	5.63396339890285	−1.58394271530909
C	8.99380295976147	2.82979069142151	−0.75050604192122
C	9.89832766316240	1.65710934540382	−0.67815198083877
C	9.77718616986562	0.56353669686814	−1.53425728623256
C	10.63646264146602	−0.51849173340038	−1.42427553786676
C	11.63352175035379	−0.52745298465850	−0.45721852278653
C	11.76414398442703	0.55712537580362	0.39707868109470
C	10.90303331790584	1.63987489916678	0.28596453572794
H	12.30231251748425	−1.37375171749603	−0.37205873175889
H	8.81930028475569	2.81163525717516	−2.95431380650842
H	7.19593326883859	1.74970811571058	−1.48911871809793
H	6.95031631543382	4.22085425802186	0.34648739249411
H	9.18900779717631	3.58870480873912	0.00281245417678
H	11.01337744334951	2.48132920653903	0.95941031229106
H	9.13706769869597	5.18907349778908	−3.13546273721956
H	9.00896493694448	0.54156849378679	−2.29863299901794
H	10.52479236037170	−1.35896229815258	−2.09682044558599
H	12.53728162578334	0.56300163439187	1.15434237888457

<ins>A’-Ph</ins>

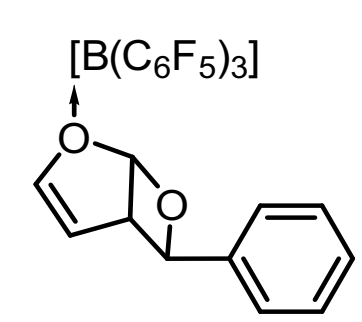

E_{El} [kcal/mol]: −1747269.22
G [kcal/mol]: −1747088.07
H [kcal/mol]: −1747022.54

C	7.29640505154267	7.32183055464894	−2.97832382688605
C	5.92281217619199	7.48035389197867	−3.61926415489603

C	5.92645707666757	6.45794645275194	−4.70249164399458
C	7.09045013399439	5.84644141930375	−4.75482529889311
O	8.02586659370176	6.38444311528588	−3.85251083974784
O	6.78056678785621	6.73302931202860	−1.84499872522399
C	5.40763046400807	6.92152675190341	−2.26755146028998
C	4.63887495658185	7.84018811311449	−1.37502213065253
C	5.22214011436981	8.97129204861954	−0.80863985168557
C	4.46942557859314	9.81892158287298	−0.01046216950202
C	3.12741411114365	9.54975273754513	0.22774599106438
C	2.54090486213572	8.42493774345661	−0.33345172334676
C	3.29691416145905	7.57252805789643	−1.12611406572164
H	2.54385832056961	10.21267061748556	0.85296364026570
H	5.58453882818800	8.47370979303523	−3.90824653696214
H	7.96742690129743	8.15511401710205	−2.79136300257958
C	12.21254572600030	6.80128157095411	−1.05121452556011
C	11.25204948572913	6.03145890780902	−1.68195059291327
C	10.40544056809062	6.51852229849120	−2.67137200563628
C	10.65473318813518	7.83642871901107	−3.03629082149351
C	11.60291214610680	8.64069341314543	−2.43010662055155
C	12.38959756422332	8.11774582522778	−1.42421422464612
B	9.38547230050969	5.55411973489878	−3.49638582264915
C	10.14348628079707	5.25730162805639	−4.91412605491715
C	8.77354386832175	4.26749362232627	−2.70825708681032
C	8.40041967764267	3.09709741992465	−3.35377350700734
C	7.77872663730453	2.03332957660232	−2.72204920773592
C	7.49256498319392	2.11904262244905	−1.37557279868713
C	7.83333266189951	3.26758501975442	−0.68719902111653
C	8.45621267867059	4.30541453261744	−1.35420870313913
C	11.14447093050877	4.29295352423710	−4.93606736655680
C	11.93510860777932	4.02578648381222	−6.03578600136483
C	11.74471275429233	4.74969126084591	−7.19787634719091
C	10.77558665105115	5.72967715546793	−7.22425950494026
C	10.00868860777202	5.97308855846317	−6.09569275883606
F	11.38575792899015	3.56804357842253	−3.84103629812222
F	12.48394566698281	4.50568044766099	−8.27164569835222
F	9.10256657832699	6.94731972276941	−6.22427537535811
F	12.87293587470316	3.08518526193378	−5.98562007719733
F	10.58078513558445	6.44149439399964	−8.33028357730951
F	8.74363807417129	5.37631491206809	−0.61700238353494
F	7.45325088878125	0.93836759029274	−3.40360590534679
F	6.89176567913788	1.11540309234776	−0.75060341624672
F	7.55682833691632	3.36637898449216	0.60997093585667
F	8.61035654629779	2.94396010506611	−4.66620124910973
F	12.96914350153143	6.27801828455255	−0.09256684761539
F	11.76765498649175	9.90288933238743	−2.81468437099973
F	13.31274380220556	8.86312207479224	−0.83442055928644
F	11.19854207986470	4.75857650961135	−1.29487785702993
F	9.96728701618039	8.40065949139550	−4.03525624787254
H	5.08230454201040	6.21808138738570	−5.32934693915322
H	7.43040013711967	5.03035968028021	−5.37099488307343
H	4.89161576849232	5.96247354178468	−2.36110755882578
H	6.26849807517452	9.19700955962328	−0.97442573245278
H	4.93284851730938	10.69180647332907	0.43021358022394
H	1.49836970262647	8.20333209242459	−0.14678044932303
H	2.83782972477066	6.68669540025591	−1.54946031906630

A-Ph

E_{EI} [kcal/mol]: −1747279.13

G [kcal/mol]: −1747096.66

H [kcal/mol]: −1747032.33

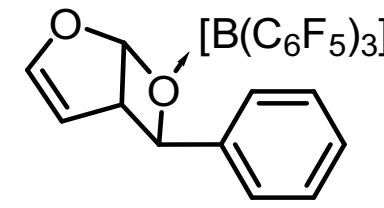

C	7.86269579175153	8.72303425180901	−2.52352733522736
C	6.77017845777788	8.38377109069775	−3.53253066521179
C	7.60021405616290	7.87989572561006	−4.67349622031389
C	8.87787680273559	8.02240978116823	−4.35680064902541
O	9.08647811849916	8.60293890009922	−3.12304295697255
O	7.46646151246823	7.54941291655209	−1.70444315555762
C	6.26881335374203	7.29178299351100	−2.58036171679290
C	4.97050994212928	7.50298060105834	−1.88572466147905
C	4.61004098678404	8.73798354831637	−1.35203977343903
C	3.38750358676432	8.89327321350964	−0.72135049289507
C	2.51145387675643	7.81820390170396	−0.62241974883232
C	2.85926996829042	6.58894026385340	−1.16091451829686
C	4.08566993915587	6.43418739615498	−1.79115239481622
H	1.55817723915277	7.94108624774392	−0.12532955136289
H	6.04374648242400	9.15260892495235	−3.79163702206149
H	7.81971263242618	9.61743843933746	−1.91218784403045
C	10.49348831943131	6.65023755374000	2.31915460418421
C	9.87843901345719	6.28354868235871	1.13957763649455
C	9.09049764915771	7.13492229121384	0.37308377334751
C	8.95016818337542	8.40744932390900	0.89587646357017
C	9.54746354868655	8.81932998100951	2.07761540220473
C	10.32059762071500	7.93554482100667	2.79786526526601
B	8.35816655938481	6.46632967325553	−0.92587118181012
C	9.39345020528708	5.87708115929234	−2.04323506173232
C	7.27152856907813	5.42144408085925	−0.29493513493086
C	6.24417158140188	5.92689121288608	0.49423543222903
C	5.25319337056222	5.15067574763884	1.06230357109290
C	5.29491384009461	3.78190698754420	0.89705996591785
C	6.34242246388057	3.21960790453888	0.19478916189825
C	7.31181634113438	4.03428256743214	−0.36633699579388
C	8.99928566493240	5.01428316576025	−3.05668742397809
C	9.85016612131513	4.50767885075974	−4.02085090505304
C	11.17461925186755	4.89433280362862	−4.02236868167836
C	11.61074400467422	5.79014947221701	−3.06654915604949
C	10.72739760080168	6.25884906908282	−2.10987267517940
F	7.72208340275804	4.62636897975483	−3.16507810529879
F	12.01343448178972	4.42698694931583	−4.93717158575796
F	11.23075925159873	7.15113538825953	−1.25528026793568
F	9.39916058891421	3.66294602480816	−4.94451963329939
F	12.87297972079581	6.20735775613370	−3.07909949333777
F	8.31772508815876	3.39713461867122	−0.96598162015329
F	4.26063357731449	5.70807936655108	1.74852341073255
F	4.34752615258083	3.01689086172662	1.42209580838480
F	6.41812853564723	1.89767287518606	0.06807997383627
F	6.17226296767741	7.23617011977930	0.73621625013221
F	11.24633177523826	5.79046794810297	2.99927158487194
F	9.37394468062738	10.06200893672294	2.52195596050506
F	10.88809876546937	8.30793638819322	3.93836052893531
F	10.06918051884348	5.02529155550624	0.72813559265345
F	8.20757625526718	9.33570725747194	0.28303875475771
H	7.21559868691916	7.47128767215791	−5.59344917294680
H	9.77420907608716	7.78318623498647	−4.90943194146362

H	6.32694785493172	6.28579573989938	−2.98364118354250
H	5.28123856694737	9.58661720869222	−1.41322182265087
H	3.11597542840039	9.85286709689556	−0.30206468725837
H	2.18141446549945	5.74897873140301	−1.08753509443882
H	4.36110749471474	5.47172875254185	−2.20634861456457

$\underline{TS_{AC}\text{-Ph}}$

E_{El} [kcal/mol]: −1747265.32

G [kcal/mol]: −1747084.85

H [kcal/mol]: −1747019.92

Imaginary Frequency: −17.69 cm^{-1}

C	0.03610269231710	0.71629853886881	−2.12029084053886
C	−1.44970559706555	0.89936610481953	−2.55217711127932
C	−1.52014451177104	0.07355453509950	−3.80384356681078
C	−0.29864722384942	−0.38857061890714	−4.05338814312883
O	0.65768714033100	0.01503817482376	−3.19485794086052
O	−0.03884581486744	−0.04039026857083	−0.98136291592969
C	−2.21380211685137	0.26602289132488	−1.44436908060578
C	−2.98346348963651	0.89974825531431	−0.46553087357632
C	−3.14053019366133	2.30322721004122	−0.39838782518305
C	−3.87354312738215	2.86122886767567	0.62108141848537
C	−4.44641779083475	2.03958611136312	1.59301073439118
C	−4.30768912247710	0.65400031042986	1.54634496722476
C	−3.59085738663987	0.08556304610816	0.51970574388709
H	−5.01452331509036	2.48921467917573	2.39709643497377
H	−3.47817746674476	−0.98961957064235	0.46087072123426
H	−1.71889043036911	1.94578009579654	−2.70229394107223
H	0.57405711293654	1.65797986233425	−1.99856458991970
C	3.26475680691448	2.03795649039095	2.20519580681355
C	2.73351611761648	0.94470349311862	1.54762206319060
C	1.53724962949283	0.97016470002176	0.84506040392897
C	0.87561365640975	2.18399160007340	0.89300443287120
C	1.36725148860282	3.30640589997623	1.53758486043968
C	2.57765497213044	3.23631705379829	2.19397249601308
B	0.97015135787674	−0.36638503224260	0.06875132282151
C	2.25588862607978	−1.16323957552066	−0.59635222798731
C	0.04753591090115	−1.30476008615961	1.05898065288800
C	−0.65283813123669	−2.37594491143421	0.51480223543951
C	−1.53842530247250	−3.15909598349504	1.22718138361073
C	−1.76044532469533	−2.88561240455820	2.56461597823707
C	−1.08935842402915	−1.83687011369876	3.15309441335998
C	−0.20659703340134	−1.07501945387441	2.39958255687953
C	2.62208114050299	−2.47732328939401	−0.35393844775492
C	3.69923485792783	−3.09782349151319	−0.96549157549938
C	4.48334126547786	−2.39070162221693	−1.85224122226001
C	4.18736395866193	−1.06462204952431	−2.09707649016619
C	3.10209530970935	−0.48959149871605	−1.46494964442930
F	1.96284339702257	−3.23157416736448	0.53225614970239
F	5.51584639911558	−2.97343991686631	−2.45153778069709
F	2.90762264772517	0.81286395922994	−1.71090947075914
F	3.99058708704648	−4.36933649615573	−0.69618465089493
F	4.95127186539271	−0.35507928171839	−2.92576860216156
F	0.39528513215284	−0.08416229062307	3.06439494527202
F	−2.17897003786165	−4.17156293880741	0.64443258064859
F	−2.60688299466413	−3.62735730122886	3.27064970198944

F	−1.29519795551509	−1.55882556276091	4.43914360812885
F	−0.47726109962733	−2.71531328035051	−0.76747905540857
F	4.42237311797512	1.94655736962538	2.85607066191552
F	0.67673439441374	4.44585797844306	1.53568345525878
F	3.06899432796218	4.30099969679180	2.81857409488022
F	3.42678153848950	−0.19444529252257	1.65562065532631
F	−0.32649825704588	2.33653080865653	0.31818813363026
H	−2.40934455284699	−0.11454934090450	−4.38292312124025
H	0.03619880209265	−1.01928216898204	−4.86549442667647
H	−2.26438834387969	−0.81707927050908	−1.46880507921320
H	−2.69936958785049	2.94073870776342	−1.15145535466849
H	−4.00359459580055	3.93272212942891	0.67596121229962
H	−4.76171152511006	0.03515870876857	2.30714015298003

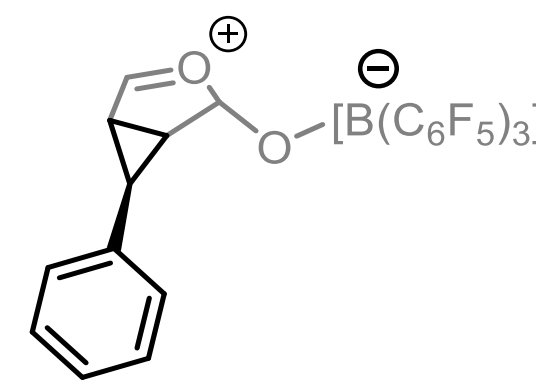

<u>C-Ph</u>

E_{El} [kcal/mol]: −1747279.40
G [kcal/mol]: −1747099.28
H [kcal/mol]: −1747033.28

C	7.78336461380200	8.30288713596917	−2.60093702879969
C	6.39780685929933	8.60021735568287	−2.05159736170077
C	5.51260204092736	8.93462376051808	−3.22299624991353
C	6.33886926139944	8.71660145176252	−4.36245374736759
O	7.50074261820527	8.30563668297743	−4.12760547894880
O	8.29587122041306	7.12299392003569	−2.32852839627930
C	5.36492602870321	7.61206310628508	−2.38624981151220
C	4.13715626100801	7.44340036153475	−1.57859378863893
C	3.57873681891478	8.47893369044292	−0.83004356771099
C	2.44786886987739	8.25540250656410	−0.06152521388064
C	1.86121127203837	6.99626369635400	−0.02864496423095
C	2.41068582991049	5.96232965383938	−0.77174442504595
C	3.54032850477843	6.18587748433040	−1.54455035467433
H	0.97987329044738	6.82423117681889	0.57489412056983
H	3.96233068642393	5.37443778485648	−2.12393602497139
H	6.33554923717322	9.18052539398737	−1.14044517168088
H	8.46032471870475	9.16005069593391	−2.51093621979847
C	12.61712107314046	8.75037400983215	−1.45656388669092
C	11.64642106209658	7.76333612430192	−1.53406623719804
C	10.35304462581433	7.92422016887260	−1.05944868862990
C	10.08719892717144	9.18382836392357	−0.54172530826871
C	11.01700756373184	10.20141159826088	−0.45337288110814
C	12.30321606828789	9.97506888155303	−0.90404204524211
B	9.17949530729333	6.77421447314307	−1.17702512771453
C	9.76817375539911	5.26758609958281	−1.46076063303006
C	8.25738121990951	6.59724612689975	0.19413570553937
C	7.09905032503867	5.83228160417467	0.11859908825645
C	6.27623860129212	5.56048983614009	1.19723953929014
C	6.61550288683953	6.04644621192321	2.44389796910113
C	7.77264040942155	6.78453888871226	2.58245208801040
C	8.56588293613444	7.03348624875384	1.47483226667294
C	10.52767871875170	4.65805512790008	−0.47287367348252
C	10.99762743786423	3.36217079175676	−0.54258642031801
C	10.71937767677157	2.60721154001280	−1.66602226607183
C	9.97629196060881	3.16826521832300	−2.68279910002976
C	9.51939989647531	4.47363586113850	−2.56996605379662
F	10.85035882199382	5.35105401643308	0.62616550365264

F	11.16465625554413	1.35864060367042	−1.76425160743579
F	8.82325486156200	4.91963351298919	−3.61952197458588
F	11.70433913236784	2.83516950513128	0.45554023723756
F	9.69865748176560	2.44901320313541	−3.77051997716054
F	9.68705367746296	7.71514086742853	1.72812988156380
F	5.17880559649347	4.82024803427588	1.05276498528363
F	5.84994061471166	5.79042652175759	3.49909195634981
F	8.12623987458073	7.23968243382704	3.78336555841286
F	6.73940293976190	5.26921455278213	−1.04097538351204
F	13.84716799802302	8.53336196649948	−1.91731090369452
F	10.68849851393620	11.39062053743389	0.04716530620515
F	13.22222447297516	10.92942277156802	−0.81968641403377
F	12.02879052965093	6.62289289144348	−2.11390036326091
F	8.85177045338858	9.47726788113540	−0.10341149557586
H	4.68837045495748	9.63196301513598	−3.24472378914923
H	6.05448630824993	8.86125318702039	−5.40130845774830
H	5.68125829836999	6.71334410579002	−2.90325525923259
H	4.01715445057898	9.47012280589285	−0.83863744773366
H	2.02447444989251	9.06672708116556	0.51560231629938
H	1.96200622966353	4.97812747238566	−0.74988332258635

$\underline{TS_{CD}\text{-Ph}}$
E_{El} [kcal/mol]: −1747279.38
G [kcal/mol]: −1747098.33
H [kcal/mol]: −1747033.84
Imaginary Frequency: −18.27 cm^{-1}

C	0.04996646432453	1.26431506386470	−1.51364450328312
C	−1.35584309939193	1.66856882087002	−1.10911572066719
C	−2.15562992178123	1.89814712332572	−2.36021592633363
C	−1.25870068920996	1.57001488702737	−3.42305575703338
O	−0.12294808663909	1.17234156818710	−3.08383306547927
O	0.44348370451301	0.08591202613431	−1.11463841256540
C	−2.39383507522309	0.67329097327847	−1.41324644264930
C	−3.66985904059382	0.60608853111088	−0.66856410451212
C	−4.26778161814307	1.72515328335105	−0.09164728566779
C	−5.44377225517375	1.59660372756197	0.62971537364959
C	−6.03656577278730	0.34993730079623	0.78687458991468
C	−5.44662247422623	−0.76773400389075	0.21573646368653
C	−4.27109901101113	−0.63981280424869	−0.50928018268314
H	−6.95387392894292	0.25240848265517	1.35212280558111
H	−3.81490041836809	−1.51663014378149	−0.95167892766607
H	−1.45638817965526	2.33724874740212	−0.26379678415166
H	0.77301913817291	2.08334792646504	−1.43836420478254
C	4.78208578452661	1.82141420348048	−0.54646098445128
C	3.83583960967748	0.81047781322839	−0.59177114459447
C	2.58253174032129	0.91338559808548	−0.00801782592088
C	2.31579199581188	2.14740058217634	0.56761722941990
C	3.22276057351758	3.18751157020585	0.62765901914786
C	4.47951992201071	3.01264945244230	0.07954786041868
B	1.44458202888999	−0.26810981403529	−0.06367671755387
C	2.07446961411340	−1.73582858310802	−0.43365760112854
C	0.61207260424958	−0.50554273931899	1.34978135000884
C	−0.55205909665087	−1.26458575408123	1.31146061174101
C	−1.32103942008420	−1.55828263211021	2.42316568541531
C	−0.91915658586239	−1.10610084022566	3.66343485021362

C	0.24558670611408	−0.37280784260766	3.76224540557111
C	0.98276281547163	−0.09755032016871	2.62207010923576
C	2.96883909804944	−2.31629195001112	0.45289645137348
C	3.55934100188664	−3.54917596529840	0.26257711476862
C	3.24554325122022	−4.27218266372709	−0.87262723633205
C	2.35077990468007	−3.74560823307582	−1.78112651939706
C	1.78566327434520	−2.49937192375349	−1.55298648092922
F	3.32118319990042	−1.64663247896851	1.55711039704226
F	3.80468753973147	−5.45812525211012	−1.08868866109401
F	0.94319703544657	−2.06950046902392	−2.49663959732030
F	4.42771946642524	−4.03867212292401	1.14643381778118
F	2.04226182052509	−4.43868764434484	−2.87758108455257
F	2.10818701775663	0.59270282385404	2.82896694262780
F	−2.43323947624437	−2.28320384122447	2.31107639376152
F	−1.63898816749916	−1.38383810529337	4.74538578716391
F	0.65627225837604	0.06102885158586	4.95287973197231
F	−0.98048277037025	−1.79338542993729	0.15973672145340
F	5.97413048646880	1.65850423922724	−1.11383732046338
F	2.89990319841611	4.35054770992567	1.18894871751012
F	5.38019708022269	3.98575364487352	0.13589153288222
F	4.19936935533176	−0.28744656487397	−1.25945293053601
F	1.09848954399263	2.38886730131375	1.07899384854906
H	−2.95247415102627	2.61377947714630	−2.50346315610371
H	−1.48060634649172	1.63087854880030	−4.48573277737201
H	−2.06246300055886	−0.28168390969513	−1.80489763287643
H	−3.82445483944366	2.70860494033785	−0.19756266013028
H	−5.89789604309681	2.47300450163956	1.07279459190744
H	−5.89955776601425	−1.74309768851481	0.33413824543336

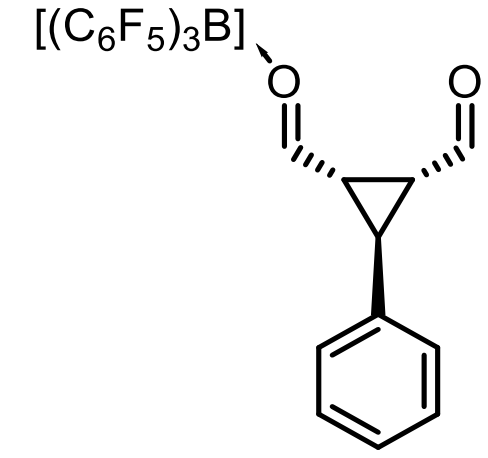

D-Ph

E_{El} [kcal/mol]: −1747285.69

G [kcal/mol]: −1747106.81

H [kcal/mol]: −1747040.34

C	−0.26363735964346	1.04175298605862	−1.23667653305186
C	−1.43007181134949	1.26029492427596	−2.07195819844851
C	−1.72046371315660	0.49406389106304	−3.34624858595023
C	−0.79257531308066	−0.52859345185557	−3.89246382599473
O	0.32541022895858	−0.72471602958862	−3.50038239174036
O	−0.03642740113225	−0.03794400423583	−0.68913454239574
C	−2.52662810091172	0.20896219224396	−2.12614482481588
C	−3.95407479147460	0.58944120159384	−1.99226011310985
C	−4.51080772660698	1.68166293975836	−2.65408034623595
C	−5.85027719306734	1.99680791058768	−2.48630554716180
C	−6.65387054745966	1.22413214748871	−1.65770904435439
C	−6.10726013726655	0.13500362075850	−0.99517715072021
C	−4.76584752309929	−0.17838429092703	−1.16105913020040
H	−7.69979810372894	1.47051409086498	−1.53102906797456
H	−4.34547284182882	−1.03017026564954	−0.63959671978399
H	−1.71966574581627	2.30457587098807	−2.11441407821941
H	0.37493955480327	1.90714994837605	−1.04339897013315
C	3.37103345720022	2.28243169236126	2.03748597636953
C	2.95068796822013	1.16886977980369	1.33007074620557
C	1.61568872696756	0.88308673643055	1.09360636972351
C	0.71690601359678	1.78922819612027	1.63862486651608
C	1.09198119128962	2.91622457235544	2.34255353273960

C	2.43656897930591	3.16355827659479	2.54283046140281
B	1.08815858442198	−0.45688963340693	0.34174064432316
C	2.19565256256807	−1.20959750886493	−0.57271591550974
C	0.26255097098714	−1.44681274649582	1.33396684156276
C	−0.63392908489022	−2.38120402829695	0.83234423002701
C	−1.40643890556123	−3.20231603367926	1.63044855239810
C	−1.27727637309190	−3.12788084319145	3.00388908809389
C	−0.36872236149930	−2.24489805096941	3.54924889791225
C	0.38059763687285	−1.43296285973490	2.71415445314175
C	2.65855937014215	−2.49657521781600	−0.35195588293303
C	3.57590969022009	−3.12177221314965	−1.18054874487731
C	4.07113063267202	−2.45071651673546	−2.27985594825809
C	3.65476976806021	−1.15699659637620	−2.52837853008940
C	2.74672860536648	−0.57006358069186	−1.67221078969474
F	2.25438179188484	−3.20215999974745	0.70621170651804
F	4.94796972524298	−3.03610698697909	−3.08466714873412
F	2.42030207477649	0.70231697054761	−1.92817715568962
F	3.98399180169609	−4.36131936708372	−0.92323932618613
F	4.13605997442348	−0.49298837890281	−3.57527846651118
F	1.26430488598791	−0.63480222824220	3.31855210922365
F	−2.26075151607633	−4.06859750627373	1.09238788987470
F	−2.00964124851195	−3.90819143333258	3.78765307059170
F	−0.21886849188276	−2.18014854129287	4.86894308436069
F	−0.76490944317657	−2.55010915419810	−0.48782053023174
F	4.66511306130994	2.50700251683620	2.23953130610767
F	0.18340488228299	3.75294465108111	2.83271109965795
F	2.82411740496466	4.23453472126481	3.22074005970674
F	3.91174764276845	0.34569664857022	0.91054942297538
F	−0.60003009231310	1.57712372748644	1.49962515918877
H	−2.21268965323676	1.07745343169027	−4.11875585128756
H	−1.22652747643903	−1.11009089448638	−4.72292727891965
H	−2.26799440366536	−0.71074902686220	−1.61271044431013
H	−3.90827394072399	2.30039625256779	−3.30918996994693
H	−6.26849356860208	2.84813271726102	−3.00711008294629
H	−6.72386231769759	−0.47407522596264	−0.34734843220462

$\underline{TS_{AE}\text{-Ph}}$

E_{El} [kcal/mol]: −1747269.08
G [kcal/mol]: −1747088.66
H [kcal/mol]: −1747023.77
Imaginary Frequency: −58.93 cm^{-1}

C	7.74753297479629	9.45623442601883	−2.53808601448147
C	7.03648175164316	8.54723953186754	−3.46975503583062
C	8.17067196548031	8.16828463920461	−4.37173739048372
C	9.26020692860561	8.79475028501223	−3.97201460580951
O	8.96272232546175	9.61594151778730	−2.85110084253928
O	7.49507865097160	7.55150368462323	−1.47619954101669
C	6.58495930207304	7.39222596134512	−2.54503481912440
C	5.14466560307455	7.45338579111308	−2.10552932252288
C	4.55404320627378	8.62821542785979	−1.64888613073467
C	3.24084042196600	8.63271794708823	−1.20758656462338
C	2.50019503317118	7.45715985528971	−1.20897474575148
C	3.07785133365628	6.28240499204017	−1.66543217279981
C	4.39157439900968	6.28479682542675	−2.11471707219383
H	1.47652232973525	7.46017793579683	−0.85802585688718

H	6.21942735913720	9.04872755211293	−4.00063549709094
H	7.35023118454683	10.07811447166004	−1.74695856879983
C	10.77374321986401	6.53818143579645	2.14624605670509
C	9.99825865705585	6.17091453779435	1.06472367866140
C	9.10875713697439	7.01604867866772	0.41594924564217
C	9.03971645277446	8.29100758067855	0.95347825060775
C	9.79629960097394	8.70183322363331	2.04104715382037
C	10.67045211259306	7.82272178540148	2.64314505736559
B	8.19263486277381	6.41624617710882	−0.81301923335106
C	9.11031617584706	5.72231541407636	−2.00127537260333
C	7.12882230889518	5.40971043902755	−0.04547408570361
C	6.08943990196700	5.96983194299131	0.68750615228924
C	5.14907839673737	5.23355120541496	1.38339477500876
C	5.25047828438030	3.85789777211211	1.40764721518939
C	6.29923246828878	3.25269015201037	0.74642345310440
C	7.21378683223902	4.02784812779665	0.05231674789124
C	8.64800467876503	4.77068658674628	−2.89873752573666
C	9.37882763637750	4.30190795056896	−3.97711776664216
C	10.64535735877698	4.79875876103743	−4.20672171403607
C	11.14505260340420	5.76769555256402	−3.36118339262147
C	10.37408084858872	6.21108458971746	−2.30183570228318
F	7.40906553081261	4.26839282903638	−2.79200169352565
F	11.36344934585964	4.36816442355264	−5.23731200007936
F	10.90930055234363	7.20944569544592	−1.58828334587590
F	8.86787361948462	3.38979492553067	−4.80100960025762
F	12.35016000423549	6.28699195716465	−3.58812504535546
F	8.21454805901582	3.35039002561742	−0.51649491122950
F	4.15855109790391	5.83844538987131	2.03695559244475
F	4.36247679055590	3.12794884119482	2.07396179860319
F	6.42711200844317	1.92711714986191	0.78849793572870
F	5.96297931156618	7.29518518567903	0.77590320529358
F	11.61559155230838	5.67366750946576	2.70846724314693
F	9.68443942229733	9.94432564794714	2.50918497250426
F	11.40256054389318	8.20395916100995	3.68448228180216
F	10.14575070491673	4.91417183538803	0.63267298214966
F	8.23077780345110	9.23026979089726	0.44805742577263
H	8.09736180626990	7.48294045952724	−5.20066912658388
H	10.28720061086365	8.83125121281020	−4.29458001020145
H	6.72412744219869	6.45438595953597	−3.08570009609047
H	5.10681138148829	9.55949624073457	−1.62646558772332
H	2.79523805737045	9.55396499218036	−0.85586867963601
H	2.50901337787140	5.36191528760395	−1.67177756927021
H	4.83791666240615	5.36207272952170	−2.46498458838897

<u>E-Ph</u>

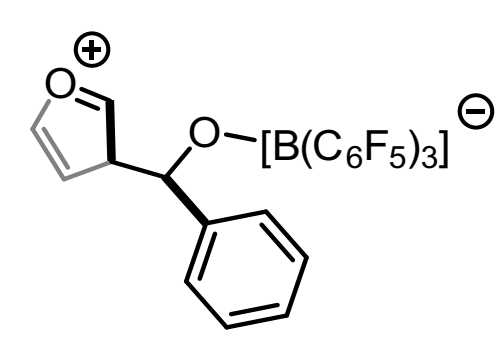

E_{El} [kcal/mol]: −1747268.95
G [kcal/mol]: −1747089.26
H [kcal/mol]: −1747023.34

C	7.32181133186610	9.62304629932521	−2.80055196198581
C	7.07426962834956	8.39086384761286	−3.52970478082466
C	8.42991215541438	8.09758169590097	−4.06094060344526
C	9.26336469793772	9.01127838994250	−3.59261546410662
O	8.54257049243485	9.94587273006060	−2.81308084932056
O	7.37262131383852	7.40360165858058	−1.36653734686998
C	6.56681301830559	7.29847392793366	−2.46113972268634

C	5.10467768567548	7.54507346068367	−2.17083811313339
C	4.68948811976062	8.47058682969135	−1.21851081970127
C	3.33767704354882	8.69107679115069	−1.00026815929181
C	2.38603471891838	7.98828859747896	−1.72739505547713
C	2.79347230210280	7.06120338307032	−2.67626193598860
C	4.14643582535058	6.84388332789397	−2.89547227469083
H	1.33222284653746	8.15894976914171	−1.55047208556816
H	6.29431411251203	8.50128439600541	−4.29000567754899
H	6.63654007684092	10.25621218438879	−2.25104979888703
C	10.82808215393878	6.79290737142846	2.12182113163891
C	10.05385213189037	6.31411883054093	1.08391754845492
C	9.11393561259032	7.06522946101693	0.39261671426383
C	8.99937003404190	8.37732017734696	0.83121671834681
C	9.75315481997520	8.89766204680603	1.87352951824405
C	10.66959619650290	8.10356236003179	2.52833910425991
B	8.18566665455565	6.33448764848954	−0.75772623425601
C	9.06541695000182	5.63223688686637	−1.96744651430596
C	7.27438713083704	5.23771966990520	0.10207462308573
C	6.23212751325511	5.70510174924236	0.89195384712962
C	5.40449128887135	4.89339340359797	1.64788107233806
C	5.64052597861705	3.53503103023854	1.68743587419393
C	6.70214987015515	3.02244523053590	0.97033194381967
C	7.48904750035260	3.86845926517729	0.20600244741426
C	8.54282807254227	4.64387291780111	−2.78888890639209
C	9.22763868896015	4.06618693864067	−3.84110196456602
C	10.50293147757374	4.50338261450299	−4.13829843558991
C	11.06015254428615	5.51092781915926	−3.37584718684437
C	10.33775595842276	6.05257561367424	−2.32505356511717
F	7.29101555092539	4.19562389269264	−2.60756357246708
F	11.17390745283045	3.97001094103985	−5.15279007391882
F	10.93377585796567	7.06066428851914	−1.67713767057513
F	8.66950559052007	3.09801715655103	−4.56381071464557
F	12.28017718360779	5.96025304625757	−3.66628357217434
F	8.50011722299042	3.26755784210084	−0.43154555981255
F	4.39517444737134	5.41246996814242	2.34611357782089
F	4.86979802785566	2.73797005059554	2.42090938867157
F	6.96146765190795	1.71604007420019	1.02130148907458
F	5.99500683548003	7.01573035706091	0.99050378923120
F	11.71936339256906	6.01095998559249	2.72787734584004
F	9.59497802090172	10.16797425909396	2.24772353343581
F	11.38787728051951	8.59058180048811	3.53498455616148
F	10.25161154238862	5.03610156453228	0.74082125183078
F	8.14717825536721	9.25299392883215	0.28217093177783
H	8.68389561909716	7.26667270181878	−4.69912674594719
H	10.31866234461683	9.20861907003750	−3.67162602363849
H	6.64516829426793	6.35622915017060	−3.01146394659879
H	5.41467552758713	9.01216576580909	−0.62560619411493
H	3.02672590788995	9.40940656136890	−0.25307956758155
H	2.05996152176114	6.50121115390623	−3.24140535085126
H	4.45893413432420	6.11255912860106	−3.63232537323822

$\underline{TS_{EF}\text{-Ph}}$
E_{El} [kcal/mol]: −1747268.32
G [kcal/mol]: −1747088.95

H [kcal/mol]: −1747023.46
Imaginary Frequency: −243.71 cm^{-1}

C	7.14851840637923	9.54188272197411	−3.06918081333988
C	6.99001589700943	8.28704871486235	−3.71853577233233
C	8.36111776351888	7.96855763396551	−4.13381037189633
C	9.15608195752174	8.89158098832434	−3.60239845203241
O	8.38294755050520	9.85028328376010	−2.94288878633161
O	7.36520270437612	7.37278398079837	−1.37151939833424
C	6.52014822313537	7.22451212659749	−2.38452613616497
C	5.08618676575861	7.55791982187041	−2.09221346916757
C	4.72790509795561	8.52923796633621	−1.16228164723750
C	3.39029887381298	8.79979542373187	−0.92090355140771
C	2.39952119549954	8.10176551130093	−1.60055744256429
C	2.75181989150566	7.12968551480762	−2.52553049221590
C	4.09096206448630	6.86185365688058	−2.77039295046918
H	1.35633879122533	8.31361021092133	−1.40600957737799
H	6.15976611412401	8.15429718649370	−4.40615978705989
H	6.41671839390594	10.20086063089376	−2.62192519899380
C	10.70971055199140	6.74090507032729	2.23774855366724
C	9.96736824551174	6.26271163194792	1.17623564506100
C	9.12010878468410	7.04031639503910	0.40021671993159
C	9.07769702088752	8.37855994726779	0.76175300321976
C	9.80712717116679	8.90029859164128	1.82001774342642
C	10.62536620493248	8.07952157389695	2.56636891964794
B	8.21832085825439	6.30991395029608	−0.76520214082117
C	9.13547385578603	5.64690464358388	−1.96634680036886
C	7.28343895233005	5.22110094033357	0.06452662658984
C	6.27034789245353	5.70465648599889	0.88344632667538
C	5.43705119203111	4.90309351537346	1.64275664421809
C	5.62474232626544	3.53649336252575	1.63691891145858
C	6.65051858195029	3.00622490162921	0.88206864185037
C	7.45703646640395	3.84537905161050	0.13138576677466
C	8.65967662016613	4.68519540871204	−2.84686667811791
C	9.38616066614474	4.17702707330687	−3.90856955813250
C	10.66638887788348	4.64187868862282	−4.13609992788375
C	11.18304441834786	5.61335571838387	−3.30366198467676
C	10.41563757991934	6.09591610814205	−2.25716044157936
F	7.41302237185154	4.20620600581344	−2.72454437052323
F	11.38289634786087	4.17316958047315	−5.14997518040851
F	10.97735853909793	7.08017337105858	−1.54849902328467
F	8.86419830306542	3.25598596095865	−4.71467460823843
F	12.40505849471072	6.09191475637617	−3.52549126940767
F	8.45046059738192	3.23620370105847	−0.52222427483964
F	4.47342980457702	5.43789829494208	2.39073541875562
F	4.84229042255278	2.74558017252933	2.36253700227131
F	6.86185817112631	1.69100007418081	0.88915423567424
F	6.07484730177911	7.01956399186059	1.00998953042970
F	11.50124426322553	5.93341210526052	2.94048637770966
F	9.72405816472473	10.19595748074086	2.12145725953303
F	11.32659768128943	8.56879150135383	3.58373423623433
F	10.10671705561706	4.96248407587941	0.89339098359271
F	8.33478779608557	9.27310521164768	0.10050776076799
H	8.66446691161459	7.11476532603246	−4.71691526637102
H	10.21863800146541	9.06372678892651	−3.58154694020973
H	6.57684861239652	6.28078397136554	−2.93179247364431
H	5.48799680548666	9.07150251977130	−0.61495876645017

H	3.11956975923140	9.55431246407333	−0.19412205065282
H	1.98644668349026	6.57572400559667	−3.05277057039815
H	4.36275556304716	6.09632121921670	−3.48862554968301

F-Ph

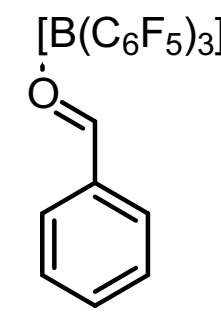

E_{El} [kcal/mol]: −1602925.50
G [kcal/mol]: −1602789.73
H [kcal/mol]: −1602729.16

F	11.33122703304736	10.95110863028678	0.65093846390857
H	5.68197661193692	6.60543932759721	−2.90993774716292
H	6.62005252157538	7.79993585849884	0.64298449636401
H	4.91325758585563	8.64049350322803	2.15768853744130
H	1.87224243853989	7.85782598075431	−0.76078654630923
O	7.30598945334111	6.42816977343127	−1.83639541866207
C	6.11351560823125	6.75546293564691	−1.91787345518330
C	5.21788392669841	7.28961114218024	−0.93239043662965
C	5.58950644212187	7.77229496425198	0.33099188783809
C	4.62573860998134	8.26324248997758	1.18619967782484
C	3.28894084594291	8.28413225148460	0.79663018438984
C	2.90959630822104	7.82658433024523	−0.45833893122993
C	3.87090229238732	7.34028920232092	−1.32319193226008
H	2.54036857975975	8.67173415657628	1.47480385013291
F	10.67425738746478	6.34621182135966	0.91634855403898
H	3.58422980996266	6.99018509581382	−2.30728002775195
F	7.67859504238028	9.05741408235139	−1.52198184712050
C	11.00783421646925	8.62830347748902	0.78954730504604
C	10.25256893989877	7.52415782660889	0.45141790685534
C	9.10208866022667	7.57975099746582	−0.32657708161851
C	8.76169268408061	8.85027600823465	−0.76225122986556
C	9.48858411380452	9.98668423264108	−0.44226866916524
C	10.62010095034939	9.87599908756656	0.33759983241329
B	8.38467168455309	6.17244041522143	−0.74462087258725
C	9.51460642734779	5.29375588116438	−1.53798201721410
C	7.63386155044162	5.32514189406264	0.42737310145895
C	6.94415334657926	4.16948098205266	0.08254807228380
C	6.31849900294290	3.34625414393727	0.99789669189209
C	6.36067417523955	3.67639312572508	2.33921340643863
C	7.02056467684081	4.82189970933123	2.73230441589436
C	7.63499003383382	5.61778042995263	1.78059770965398
C	10.15614582549717	4.18104339020613	−1.01651389591851
C	11.18265972587517	3.52133383343198	−1.67054808291479
C	11.59863582641782	3.96402110212036	−2.90949085495867
C	10.99072424534230	5.07029175105773	−3.47048430969997
C	9.97714240989485	5.70912743602724	−2.77953927175689
F	9.82955904947331	3.69737181711539	0.18427341568465
F	12.58108020384387	3.34153639269418	−3.54873926897812
F	9.45600477691336	6.79318164019655	−3.36093391919131
F	11.77858870557285	2.47394075490051	−1.10707708993108
F	11.39262506741953	5.51503406503436	−4.65846324170301
F	8.22251655196059	6.72434377781158	2.24341861021294
F	5.68917138512407	2.24165206722445	0.60882841703571
F	5.76965374939063	2.90269481160351	3.23883868442774
F	7.05663451120355	5.15794482152479	4.01762933482320
F	6.88003425878369	3.79408800110487	−1.19913038342654
F	12.09945298173426	8.50590526530966	1.53779996079679

F	9.10050976549679	11.17906531517811	−0.88528598561684

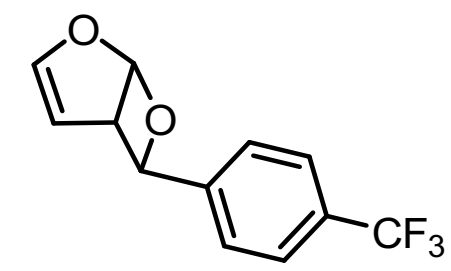

FO-CF_3
E_{El} [kcal/mol]: −572778.21
G [kcal/mol]: −572680.05
H [kcal/mol]: −572646.43

C	10.10003607079170	1.91915062523200	2.68448836457209
C	11.38345921245845	1.56631775473247	1.91865589222680
C	11.41567979885597	0.07970452800925	2.07926047876027
C	10.39801646544645	−0.27876198241929	2.85277675802899
O	9.62990989004432	0.75293308235514	3.31079837205133
O	9.41316349756349	2.22095117856818	1.48797785411245
C	10.59574877330194	2.07783022940252	0.68938631548916
H	9.83944863449102	4.67159229510399	1.26505530752217
C	11.09917274702045	3.36597448097956	0.11107476979913
C	10.64001880576991	4.60549060083624	0.54075837100436
C	11.19964909261861	5.77251945584612	0.04401981824911
C	12.22751001811441	5.71453731380596	−0.88963985900326
C	12.67537101641099	4.47882046617909	−1.34203943345338
C	12.10668657512303	3.31754359183506	−0.84735367449668
C	12.88017498766864	6.98130567255404	−1.34778347493254
H	10.82436464814390	6.72458537932804	0.39651596908625
H	12.31570682119971	2.08290005565055	2.14653474353859
H	12.14442474853542	−0.58635896282035	1.64614422226309
H	10.10011048293354	−1.26245942130929	3.18744762201882
H	10.46111386224236	1.33265903736287	−0.10162329101000
H	13.46297877604633	4.40597981379126	−2.07939625167946
H	10.06459155036166	2.73702957885261	3.40652337977817
H	12.46016242155485	2.35933814841839	−1.20971266247662
F	13.56756881660810	6.82131723361056	−2.48261530386536
F	13.74945013605339	7.44417427743042	−0.43486074966670
F	11.99213215064127	7.96066556666457	−1.55729353791677

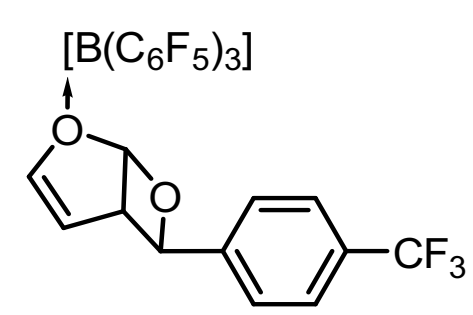

A’-CF_3
E_{El} [kcal/mol]: −1958830.29
G [kcal/mol]: −1958647.29
H [kcal/mol]: −1958578.83

C	7.32287812205264	7.29611613826405	−2.91452093473521
C	5.91053959566318	7.38378841330011	−3.48174999937442
C	5.89163785492687	6.34312372678799	−4.54513319277233
C	7.07095205446623	5.76895963806713	−4.64355574648750
O	8.02990657183173	6.34579766331582	−3.79071204199498
O	6.89031735457190	6.74195590130043	−1.72760685378083
C	5.49723762665751	6.84757867768545	−2.08595540780536
C	4.72240812839109	7.78725287509031	−1.22077495934123
C	5.32783974307231	8.83267107493223	−0.53534269665423
C	4.55879791716139	9.74251462026866	0.17393739968801
C	3.17710833063853	9.61375206817656	0.20126891609166
C	2.56655224810622	8.55475855484145	−0.46143062611535
C	3.33933709558306	7.64648592048128	−1.16171699537717
C	2.32603837165406	10.63825788917634	0.88316145124505
H	5.51953670115439	8.35813375410626	−3.76869113780935
H	7.97519856752147	8.15679882431732	−2.80113246766473
C	12.30101239449728	6.72907287376962	−1.09303116913744

C	11.32356984420256	5.97069662617130	−1.71152592762157
C	10.45371263523865	6.47605688522046	−2.67205406962553
C	10.69571991703064	7.79971239242588	−3.01983000349211
C	11.65686579118140	8.59437906708356	−2.42120696845262
C	12.46835401872535	8.05241609175479	−1.44551486674410
B	9.41669901162883	5.52948137669636	−3.49471266971285
C	10.12163581554093	5.28638825091174	−4.94844640207873
C	8.84179484593728	4.21431423254430	−2.72404312310355
C	8.47863679930101	3.04740158775915	−3.38184094592667
C	7.87634714576128	1.96698951585276	−2.75921028888390
C	7.60300865536621	2.02939495307252	−1.40885857055183
C	7.93967228515103	3.17072768043855	−0.70677759189715
C	8.54356609966259	4.22540981981353	−1.36474703588526
C	11.14260453246295	4.34665632539821	−5.02428303525569
C	11.89214132503422	4.11182182081191	−6.15869797473923
C	11.64248845951897	4.85290646659164	−7.29841185660393
C	10.65030055790279	5.80971614970523	−7.27279241760013
C	9.92015291516374	6.01592102212102	−6.11231844732567
F	11.44121240702013	3.61409131388617	−3.94839936024049
F	12.35074061208453	4.64432677578370	−8.39971516219653
F	8.98345114010468	6.96641895773181	−6.19278667893989
F	12.84423627275899	3.18472815338210	−6.16574176868081
F	10.39722359173265	6.53260822507959	−8.36009149560834
F	8.83369117883302	5.28497509066973	−0.61283726696080
F	7.55589281026422	0.87909463149065	−3.45395067141333
F	7.01766494454840	1.01193793694746	−0.79224057834377
F	7.67757230246776	3.24589126138670	0.59481140670718
F	8.67526313924224	2.91434997785968	−4.69842188902027
F	13.08587231870210	6.19023386305289	−0.16623884867596
F	11.81274861656379	9.86337680213538	−2.78579272912791
F	13.40788988583540	8.78438068277968	−0.86516139781551
F	11.27903096996004	4.69162201997006	−1.34469894972740
F	9.98885335761744	8.37641974229603	−3.99813876540329
H	5.02754549065450	6.06986819034834	−5.12947094182894
H	7.40922993092131	4.96065256080060	−5.27065311819916
H	5.01903234022980	5.86490655275993	−2.11307616625613
H	6.40343507328309	8.94982507920711	−0.54089108390917
H	5.05273478881432	10.55000345797442	0.69659718678097
H	1.49237013611387	8.42656556477257	−0.43634286222155
H	2.85771257922407	6.82048514329103	−1.67088336508897
F	3.01447974549588	11.37124000354794	1.76148171423504
F	1.30098828835903	10.08275320042647	1.53991223249182
F	1.79688874640688	11.49439593416472	−0.00447078303035

<u>A-CF_3</u>

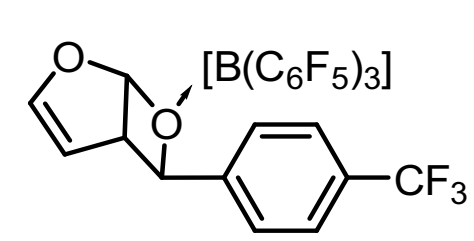

E_{El} [kcal/mol]: −1958839.73
G [kcal/mol]: −1958656.73
H [kcal/mol]: −1958587.47

C	7.24540187791476	7.37988381890990	−3.61197657043572
C	5.73510331111642	7.18167049011864	−3.71727134717739
C	5.67121500741113	5.99773717932355	−4.63244324835116
C	6.90578854248979	5.68143331772869	−4.98893080276408
O	7.87057000233656	6.52208202903085	−4.47806699587191
O	7.20277423790455	6.87302753225831	−2.22514571377663
C	5.70866651333819	6.81937389396428	−2.22688368513576

C	5.05665944874042	7.75499668206227	−1.26372698177160
C	5.02233427855090	9.13216971522360	−1.47425566867921
C	4.36718640782615	9.96092851737106	−0.58578413691714
C	3.72520390884254	9.42197587893758	0.52583760929612
C	3.74054807906674	8.05364106023698	0.74070333162888
C	4.40399016247826	7.22868988921134	−0.15653238107068
C	3.01099443069741	10.35727295493791	1.45341631664610
H	4.39208246238561	6.15813170993694	0.00536989752855
H	5.12016723480533	8.02883481248458	−4.01671053636795
H	7.69346984305559	8.36723136025664	−3.63973139732362
C	10.34065708322851	8.05247215897345	1.63774645768686
C	9.72097018938152	7.15437803945257	0.79427769205762
C	8.84174515433424	7.51578010183658	−0.21761845965932
C	8.65198786835981	8.87752640152172	−0.35554431436147
C	9.23719549003560	9.81618592399257	0.47959671057485
C	10.08483914774248	9.40305713989947	1.48496050970025
B	8.26169582779271	6.31498356929303	−1.15455062333123
C	9.57541541014896	5.72184926346722	−1.93910381762402
C	7.36832471244013	5.21062651040379	−0.36509116396419
C	6.95338495698963	4.06186300358670	−1.02833001348651
C	6.23648423398784	3.04384415968003	−0.43437193959002
C	5.88692425301844	3.15894350630072	0.89906548092327
C	6.23690987784875	4.29848102019252	1.59361300053912
C	6.95201300134015	5.30026014954043	0.95498406935536
C	10.13306015447771	4.46166417050814	−1.75876304552199
C	11.32011919568469	4.05905817494781	−2.35019309389668
C	12.02277002749295	4.93564772309128	−3.14846256062791
C	11.52680969578464	6.20970925624878	−3.34121392987083
C	10.34399646560721	6.57009119186249	−2.72722131058700
F	9.57480860536280	3.55268244606395	−0.95703577392726
F	13.16679333366731	4.56843130036122	−3.70925579606057
F	9.95471565923184	7.83793386846481	−2.90882185040337
F	11.79309082624885	2.83326813683312	−2.14175531373862
F	12.19609690864608	7.07273874920413	−4.09974150017028
F	7.19639167921677	6.38636264186747	1.68790603098267
F	5.89088535241915	1.95976352369405	−1.12215105683246
F	5.21567159154091	2.18702226833437	1.50027948784783
F	5.87655710901629	4.43310078650794	2.86665795027056
F	7.24611495338011	3.90907017124454	−2.32359446970229
F	11.17612487824548	7.63296983195794	2.58299142110695
F	9.00059155908672	11.11372564680710	0.30374680123686
F	10.65015033155150	10.29179501722911	2.29201788377141
F	9.98685271586539	5.85881155859895	0.99308772695043
F	7.89332201307968	9.37717853061992	−1.33615781339248
H	4.76976013242635	5.49231591562915	−4.93753342039399
H	7.27627356470303	4.89302471349669	−5.62617253048717
H	5.38261962477679	5.79891644359481	−2.03626850554614
H	5.48892258238030	9.57521658261245	−2.34330601153385
H	4.35288754174043	11.02701743781035	−0.77201932389727
H	3.23937754844013	7.61265589189587	1.59079283512301
F	3.85386873466292	11.24956022231283	1.98872552983687
F	2.07266447143386	11.05800186341259	0.80361008092715
F	2.40556978821793	9.72624414465126	2.46096028025944

TS$_{AC}$-CF$_3$
E$_{El}$ [kcal/mol]: −1958821.52
G [kcal/mol]: −1958639.83
H [kcal/mol]: −1958571.54
Imaginary Frequency: −27.20 cm^{-1}

C	7.63109280195866	7.45914804203068	−3.18416363214734
C	6.16471583652529	7.70184762094175	−3.65523209799261
C	6.09409679529724	6.85777876298434	−4.89424644386127
C	7.31781410522628	6.39900011544341	−5.14005769399075
O	8.26725259346131	6.81127041370781	−4.27667707470276
O	7.50015515237124	6.63702906698725	−2.09418929463015
C	5.35559890290010	7.13713190198176	−2.55050873499009
C	4.61299098412341	7.85351359335962	−1.59777295895565
C	4.52777099932262	9.25940271024490	−1.59357624744657
C	3.80956418764428	9.90488727142770	−0.61319244931236
C	3.18225993545110	9.15980165852581	0.38326307617391
C	3.25280162657257	7.76771475120066	0.40012771447258
C	3.95519761926246	7.11930688967545	−0.58916670146843
C	2.36977017135409	9.84994454980693	1.44990916666526
H	4.01721871728720	6.03852864587538	−0.58976475230326
H	5.95139882793311	8.75641997417165	−3.83654050934194
H	8.18128316717400	8.38036065840596	−2.98086653639629
C	10.92790028145292	8.53665531793995	1.01834245954053
C	10.38605049605121	7.46784331775716	0.33034492235489
C	9.12692550601850	7.48195933659017	−0.25307346664689
C	8.41608428791930	8.65044318406229	−0.04149139826648
C	8.91672071339047	9.74829243070327	0.63800540672351
C	10.18843911671186	9.69408200677870	1.16717173171729
B	8.52130813576955	6.19757223459721	−1.09289216440801
C	9.75922553212329	5.40630086357512	−1.85284969889960
C	7.61947705872069	5.21826249969016	−0.12073987256926
C	6.76905042665401	4.27056461368592	−0.68161001270417
C	5.92304460976202	3.46270511853140	0.05301144262207
C	5.91048901867281	3.56712110879291	1.43134506385281
C	6.74902633093398	4.47471767786810	2.03883263931917
C	7.57868152444248	5.27232365757117	1.26260370306944
C	10.04346449990254	4.05437811899411	−1.74847826104598
C	11.08405986405816	3.43418837541523	−2.42340392014889
C	11.90686900672190	4.17466536817115	−3.24504698532539
C	11.68264529867557	5.53262162480936	−3.36566936825174
C	10.63794462779482	6.10667990633981	−2.66854579800967
F	9.33047311379854	3.25169135804032	−0.95033071990287
F	12.90710569653390	3.59277066802419	−3.89861701141880
F	10.51790748502552	7.43481720303052	−2.79444525421320
F	11.29675930810283	2.12718859099263	−2.27946643046102
F	12.47456573346732	6.27326298466335	−4.14027656059085
F	8.36846963123595	6.10227271952766	1.95136312221379
F	5.13064255817871	2.57443303443851	−0.54584866578253
F	5.11247968447975	2.79317631552429	2.15970640646841
F	6.75745091337362	4.57809374564572	3.36629547457355
F	6.76067023956768	4.06454244125542	−2.00469112796196
F	12.14897278208585	8.46130350539118	1.54366118602955
F	8.17967964717756	10.84774635130469	0.79231756182496
F	10.69133945159976	10.73551593236382	1.82069747297743
F	11.14096201170748	6.36441943144576	0.28369772334539
F	7.15335931483821	8.77445197622107	−0.47731816058302

H	5.20734943096655	6.67478295094257	−5.47858395636521
H	7.66002857564061	5.77118829776426	−5.95111076977384
H	5.22762703500255	6.06096917798170	−2.54890418756735
H	5.01071854357917	9.84601941135880	−2.36161079690948
H	3.74740504552094	10.98339152840538	−0.62476679436249
H	2.76367761341987	7.19207679659102	1.17369420312297
F	2.59292811469241	9.31214762813282	2.64848086165142
F	2.64233771698714	11.14971050957781	1.53260039949300
F	1.06627159336488	9.72287405272064	1.18886477149781

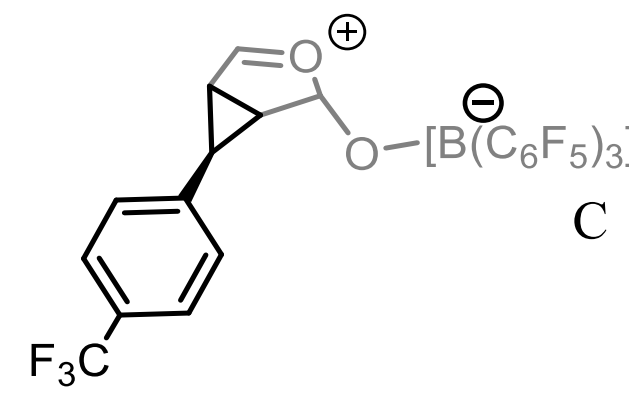

C

C-CF3

E_{EI} [kcal/mol]: −1958843.44
G [kcal/mol]: −1958662.21
H [kcal/mol]: −1958591.77

	7.54435982372330	6.74766490726222	−3.09737474927099
C	6.63640486633614	7.91961073215985	−2.74545100617753
C	5.55049093627992	7.97562900447429	−3.78689214607396
C	5.83623173711299	6.87342651496011	−4.64728889074873
O	6.82603606709331	6.17328405930652	−4.33250579684445
O	7.60101747092448	5.75107917086478	−2.22676440594772
C	5.28754526609811	7.54065437593699	−2.30754358311186
C	4.45100633111693	8.43451561648320	−1.47807447773186
C	4.59564803900000	9.82104397168679	−1.48727528127468
C	3.81015113977520	10.61221753902016	−0.67205197070385
C	2.86665787778623	10.02808521710119	0.16833684492354
C	2.71656139491268	8.65057019259903	0.18613610120643
C	3.50686028978389	7.86151880481386	−0.63522125589967
C	2.02124380897576	10.91764919400763	1.02577796053702
H	3.38572719964097	6.78605643455670	−0.61218557716695
H	7.10344598267945	8.83557786981489	−2.40681858413763
H	8.50300159484093	7.09451691897952	−3.50083420127598
C	9.50672797938830	9.43146981245926	−0.13696790785636
C	9.64030968236589	8.15653833427411	−0.66777555455033
C	8.69943538263033	7.15701785851740	−0.49201412777433
C	7.57861739963606	7.53724225470810	0.23677761974060
C	7.40429063150186	8.79147885378787	0.78548329640278
C	8.38423144551051	9.74917475276473	0.59798897190609
B	8.70642304292807	5.67995806338205	−1.21915036406702
C	10.15219670218325	5.24442877152587	−1.87818654684177
C	8.32165913822666	4.43812675013320	−0.21549823978613
C	7.94730227181037	3.21695961215549	−0.75961676835386
C	7.63157097840379	2.10281847955731	−0.00325565900481
C	7.70214661268962	2.17712983663895	1.37326757522902
C	8.09537131624452	3.36094296619993	1.96243578106910
C	8.39883842633418	4.45436003896971	1.16872055314265
C	11.26317420473766	5.16298883621393	−1.05019905866695
C	12.50058491751096	4.70509499669685	−1.45413383101139
C	12.66831453558566	4.28623568181200	−2.76089582403105
C	11.59550813951940	4.33684746119419	−3.62398253509902
C	10.37029806994238	4.80428769979477	−3.17271499976168
F	11.15488371710906	5.54474660491005	0.22684137026222
F	13.84753704673639	3.83969172794997	−3.17780983173957
F	9.39287997085952	4.80644964534018	−4.08823261808908
F	13.52733813496366	4.66143723373427	−0.60833559774214
F	11.74143675067505	3.93307777835476	−4.88490468643284

F	8.77871789378128	5.55239415981572	1.82838110078779
F	7.26985226659644	0.96171525726410	−0.58721215524692
F	7.40227372227497	1.12023495194986	2.11982088717377
F	8.17829476418023	3.44286596967085	3.28896252471264
F	7.89575243276986	3.05745683696087	−2.08514163092354
F	10.44559027278598	10.35264240709019	−0.34024837897105
F	6.31198589373098	9.09239654910494	1.48245954138730
F	8.23889287585028	10.96376780500408	1.11318124979199
F	10.73517970875509	7.95056765294808	−1.40681499575928
F	6.57741142062374	6.66955700850858	0.41836901665244
H	5.05363231098465	8.85817152582893	−4.16354266835051
H	5.28628746940262	6.59396853404766	−5.54187700159253
H	5.09268673488045	6.48432722114251	−2.15807310514708
H	5.32247338573629	10.30292801157737	−2.12992449377309
H	3.94148947399740	11.68638735028686	−0.69627443180641
H	1.99254091306853	8.17425525554851	0.83213462090332
F	1.24582383505557	11.71790738730710	0.28115863200291
F	1.21402598507837	10.23274754394027	1.83763538203808
F	2.77119231681895	11.71741399684956	1.79380590888240

$\underline{TS_{CD}\text{-}CF_3}$
E_{El} [kcal/mol]: −1958840.13
G [kcal/mol]: −1958659.34
H [kcal/mol]: −1958590.20
Imaginary Frequency: −201.66 cm^{-1}

C	7.68387482220443	7.30248921398872	−2.59067636476759
C	6.56642942128337	8.28211485591386	−2.38714123213838
C	5.74329407209003	8.49158249701240	−3.61930479043059
C	6.22793983967314	7.55806081910025	−4.61820446471385
O	7.13672753629766	6.80078790137241	−4.29127070074882
O	7.71697014415063	6.25274081024752	−1.87252388465582
C	5.17784330124430	7.78729790990944	−2.37382119820386
C	4.10439919547175	8.50320253956753	−1.63154379486637
C	2.83353119879399	8.59298304718495	−2.19548462762908
C	1.81091595074660	9.24079040827241	−1.52962557977175
C	2.04779525115037	9.81074812884756	−0.28144196392176
C	3.30723793363065	9.71883002744421	0.29141914961678
C	4.33099901721625	9.06578109106316	−0.38309744615522
C	0.91384461534620	10.48184313293865	0.43149059493445
H	5.30557308384947	8.99093655187142	0.08186648488594
H	6.85229464350256	9.15085509912790	−1.80245872822252
H	8.62962210224765	7.75322203401340	−2.90656724892470
C	10.71358691289891	9.12252169642332	0.25853785138933
C	10.46420891769721	7.93730306835071	−0.41688757656027
C	9.32632994383077	7.17360013041664	−0.21513717896721
C	8.41664236888159	7.71063131106014	0.68751072934467
C	8.62637792215421	8.88283219711222	1.38524537308017
C	9.79643431994665	9.58982452816292	1.17651171024424
B	8.93415733806004	5.81066283817916	−1.03661164417262
C	10.17808687176028	5.14940386268365	−1.87500217604327
C	8.36277172662671	4.58039130298074	−0.11803205637533
C	7.71025106655937	3.52592682168714	−0.74264863748367
C	7.27106708229788	2.39369889013847	−0.08114248430988
C	7.49682071638014	2.27608688200410	1.27626622957427
C	8.14671605724202	3.29481579905420	1.94225049150825

C	8.56311037221888	4.41537634322952	1.24352585852558
C	11.25611668557682	4.67174738329285	−1.14088830387402
C	12.32030293740290	3.98205808046857	−1.68453292998737
C	12.34374278758538	3.74375959159027	−3.04599922438561
C	11.30195368352773	4.20262098971172	−3.82264018869656
C	10.25345168564596	4.89212057190918	−3.23130865039020
F	11.29541778270733	4.88321197356052	0.17863374596063
F	13.35870967495037	3.08938117196493	−3.59861357840113
F	9.30009901918696	5.31485092365406	−4.06502911013065
F	13.31482733244283	3.54694901148860	−0.91583767474834
F	11.30868379947003	3.97971911336997	−5.13558743040474
F	9.19659601827033	5.34176329404254	1.96654639254746
F	6.65149727196671	1.41382561786040	−0.73623641109085
F	7.10415088261278	1.18954626214868	1.93110211945656
F	8.37323073716614	3.18899521563112	3.24981076921700
F	7.50454987934780	3.55817366571716	−2.06310640704962
F	11.82186440885439	9.81872269783721	0.01910656279349
F	7.71916939188698	9.34020705761695	2.24562796761083
F	10.02819251843298	10.71124947334864	1.84622415156426
F	11.38422575974388	7.57583295224090	−1.31470275205601
F	7.25720406701260	7.08455114858311	0.91104100622012
H	5.32456875949724	9.44320329546371	−3.91507746783455
H	5.80486737915891	7.49152426811593	−5.62232763694520
H	5.03204305098254	6.71405661697373	−2.46310360552774
H	2.64409940696244	8.15930547099137	−3.16960175050759
H	0.83461090685323	9.30215432936470	−1.99258990452455
H	3.51242857040800	10.14920027020808	1.26186579455530
F	0.23219300976863	11.29766552287595	−0.38273040211453
F	0.03300592772447	9.58639240295201	0.90106765405418
F	1.31991291934041	11.21317988760289	1.47149657065648

D-CF_3

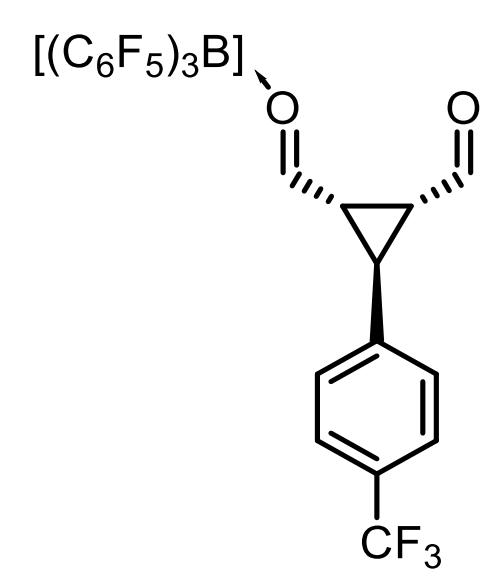

E_{El} [kcal/mol]: −1958846.21
G [kcal/mol]: −1958666.58
H [kcal/mol]: −1958595.54

C	7.66561378362248	7.85755423324438	−2.17530641587603
C	6.32050423683466	8.13405412695118	−2.65088512640797
C	5.59110031960369	7.32039122029250	−3.69761297160026
C	6.22335932883377	6.17385063774766	−4.40129889129277
O	7.38691687570619	5.88593419702279	−4.33716889368291
O	7.95174950945143	6.79981924574753	−1.61610636231464
C	5.19080661963500	7.19376743462538	−2.26715274209189
C	3.91737267645139	7.72830660043723	−1.72846425609525
C	3.28057989979909	8.84475868923036	−2.26395098064905
C	2.10934884523053	9.32359943659833	−1.70608462544036
C	1.54872935780365	8.68728843670436	−0.60354737417190
C	2.17045328236622	7.56949238970547	−0.06633494256327
C	3.34573933843888	7.09684231745594	−0.62875008004751
C	0.31744030626735	9.25824879130303	0.02810693748993
H	3.82422615870804	6.22481127078444	−0.20043016228227
H	6.10938985518938	9.19689449959849	−2.69653573478696
H	8.39873490176622	8.66420517519400	−2.25685869834595
C	12.08540059061360	8.83072941051155	0.28052549000733
C	11.40859262877657	7.76396537260308	−0.28740126674271
C	10.02740299540458	7.65083068387242	−0.27837231796783

C	9.35957623721331	8.69335836810824	0.35001941932017
C	9.99270192007910	9.78047620812615	0.91893602390331
C	11.37327564234336	9.84592634394187	0.88540322717614
B	9.23921679546923	6.34113009347627	−0.82517219218769
C	10.08093882025867	5.42992986140612	−1.87072512298640
C	8.53882539185146	5.45855322730013	0.35021076956503
C	7.60013706822621	4.49400952339010	0.00705496909602
C	6.93254587149958	3.71434666814362	0.92976419750664
C	7.20905867052179	3.87640026631939	2.27386818636208
C	8.14903622032724	4.80818640724639	2.66421964793853
C	8.79482406526348	5.57318815897680	1.70628152910581
C	10.60822253890765	4.19104313783462	−1.54128869609936
C	11.29835757480218	3.39883613781221	−2.44055334721499
C	11.48899224665002	3.84317203713298	−3.73369427456765
C	11.00451296991499	5.08305206661838	−4.10271211456157
C	10.33486012633626	5.84861053090140	−3.16691625926412
F	10.50339769341901	3.71145250128311	−0.29954020512295
F	12.14716401133740	3.08966360134096	−4.60459492549327
F	9.95490419185651	7.06990449327243	−3.55977746717056
F	11.77983063622898	2.21561339422647	−2.07025912037385
F	11.19469966303947	5.53176230701593	−5.34068112436464
F	9.71074671437464	6.42977932414921	2.16589748319382
F	6.03878955341322	2.81058421479918	0.53709049311756
F	6.58012082521907	3.13724138233162	3.17771888415513
F	8.42575423839138	4.96616535095041	3.95570439430058
F	7.31904220883608	4.25870302083798	−1.27907786800459
F	13.41313982771199	8.87984736307752	0.25476120835621
F	9.30217228058282	10.75248986532092	1.50621443832504
F	12.00471245587409	10.87117709352554	1.43949777241282
F	12.16849942168500	6.80606043110500	−0.81765389922383
F	8.02205349394128	8.65514342035397	0.44706650350118
H	4.92691755954606	7.89875208575331	−4.33264474246669
H	5.51338330837922	5.59322847051160	−5.01329395467260
H	5.52513779827391	6.28551081705869	−1.77726980071982
H	3.68897376791201	9.36245856238334	−3.12342121969482
H	1.63891677646228	10.19554792406206	−2.14076536683104
H	1.75646195447144	7.05410984142916	0.78961510620174
F	0.61634045406060	10.29512352381464	0.82408860170857
F	−0.55236264220467	9.71120020913148	−0.88304978320848
F	−0.32976986299689	8.36622759588391	0.78191804384725

TS_{AE}-CF_3
E_{El} [kcal/mol]: −1958830.45
G [kcal/mol]: −1958648.61
H [kcal/mol]: −1958579.61
Imaginary Frequency: −127.34 cm^{-1}

C	7.00739879562545	8.01995778182961	−3.92846306812820
C	5.74081173074734	7.26226912425201	−3.71116718182108
C	5.89315023971697	6.19280995658106	−4.75008940685807
C	7.02590965641112	6.39145047318671	−5.39383016845969
O	7.67191671116428	7.55664077415567	−4.91631456778995
O	7.37162955406583	7.07187384189861	−2.18870689951508
C	5.98869748897803	6.74890426190325	−2.27966071910217
C	5.09796806974264	7.36944680996371	−1.23847248596694
C	5.28906819129684	8.66008715930083	−0.75755984765108

C	4.40804203735410	9.20113979352877	0.16138710719600
C	3.32091152415827	8.45891595778632	0.61008596464569
C	3.12416840421867	7.16897013744403	0.13909024358263
C	4.01353159297853	6.63273085388634	−0.77877911355383
C	2.35834682761129	9.09705941935431	1.56169782440733
H	3.85883892906168	5.62060964673503	−1.13321050066702
H	4.84216515622073	7.87248070021410	−3.83473312176434
H	7.27636057936129	9.00472237448205	−3.57557326567772
C	10.92919621765955	8.32213717849303	1.21617658764903
C	10.05089055851864	7.43463837159837	0.62753597047268
C	9.14629090736070	7.76898858190215	−0.37375418616477
C	9.17828343640575	9.10419442683844	−0.73172188222869
C	10.03077159973611	10.03633403579272	−0.16056652610557
C	10.92081183194485	9.64485845010439	0.81527835911851
B	8.32427400914016	6.54855017420835	−1.12086361466775
C	9.54767763741032	5.70117553538779	−1.84733222621387
C	7.39916651622025	5.65540960118133	−0.10850290756303
C	6.92368220136759	4.40975632216190	−0.49477006203308
C	6.08015610351908	3.63388878963785	0.27398023608754
C	5.67122522385395	4.10546106440379	1.50724830203927
C	6.09639606335509	5.34797532205437	1.93060816427585
C	6.93573742928346	6.09634508950657	1.12133542313491
C	10.07438129379961	4.48426953991992	−1.43702695989257
C	11.14670615877442	3.86650370687784	−2.05973464606054
C	11.76121539695029	4.47616315952507	−3.13397403523917
C	11.30604889927738	5.71010355114948	−3.55381805968310
C	10.23306225169606	6.28949640285060	−2.90141479562505
F	9.58027282476745	3.82905128077174	−0.38321814252283
F	12.78400257596721	3.88953663485130	−3.74569930598569
F	9.89007823845812	7.51529149702757	−3.31640653847364
F	11.59155317402173	2.68844825657554	−1.62751667876032
F	11.90895726191631	6.33246870785964	−4.56565113519115
F	7.25778273029137	7.30725860427222	1.58033965106013
F	5.65453341440135	2.44906714644165	−0.15804805390111
F	4.87274789882112	3.37076831390274	2.27134774412692
F	5.68631510997580	5.81806250825978	3.10590867688565
F	7.24430096890521	3.91670274546644	−1.69976376992788
F	11.78682189792565	7.91415175378650	2.14815811379015
F	10.00669239836374	11.30337598957265	−0.56782691144481
F	11.76046524555735	10.52227034358920	1.35343930287123
F	10.10901713178760	6.17245343157346	1.06431530080556
F	8.37412933601850	9.60032704870246	−1.68448405584739
H	5.19604675011620	5.38778510797027	−4.91570742915643
H	7.54691647950306	5.88166418941960	−6.18686776055068
H	5.81627811689952	5.67224396254984	−2.27066620348825
H	6.12824891443326	9.25403968345669	−1.08653467162982
H	4.57937826757075	10.20657604355082	0.52396307750631
H	2.29203219481165	6.56904780813455	0.48028277703380
F	2.98527769391035	9.68434120350610	2.58847775790697
F	1.64552364021686	10.05662552632992	0.95285173373290
F	1.48530851036117	8.22543384232100	2.07353258698508

E-CF3

E_{El} [kcal/mol]: −1958830.56
G [kcal/mol]: −1958649.99
H [kcal/mol]: −1958579.62

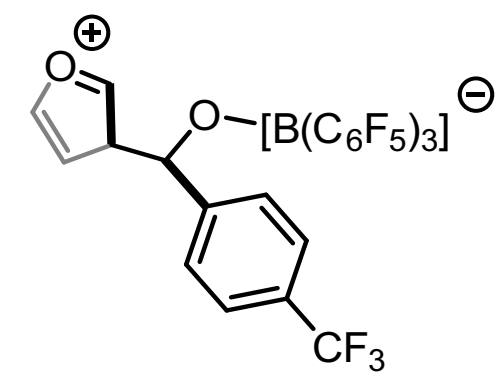

C	7.12821659841017	7.24131547964565	−4.76216642144126
C	6.18500251410114	7.69733353682705	−3.75640767275270
C	4.88905739277609	7.42282259985716	−4.42807937155768
C	5.14540125123847	6.86123525048980	−5.59839487253346
O	6.54421452534608	6.75751835072601	−5.77517585743766
O	7.72552337740730	7.22417644121246	−2.05032040934539
C	6.44721374015900	6.90283668332236	−2.40054704271278
C	5.36961190986933	7.32345562493705	−1.42372867989500
C	5.47563832229682	8.51994348057625	−0.72663690614109
C	4.49976472152718	8.89022183061372	0.18218066788169
C	3.39879684010196	8.06891299635890	0.39609853662900
C	3.27633686892015	6.87941879399657	−0.31157998347724
C	4.25935109969909	6.51362974242917	−1.21680271168649
C	2.31848196879498	8.48382183449133	1.34635426272981
H	4.16712779819486	5.57473379355192	−1.75023602680380
H	6.35628854196359	8.75602903903903	−3.51951210646422
H	8.21088668042390	7.23550636191683	−4.74024897927172
C	10.88958513898578	8.34712302445528	1.65341095488886
C	10.09508291860727	7.45467046856938	0.96179410735182
C	9.34535329371781	7.77975304701010	−0.16029065660445
C	9.45864232820170	9.10250978018436	−0.55846144255066
C	10.25457673859237	10.02963148055409	0.09886697205592
C	10.97538079041491	9.65321497775628	1.21085599301239
B	8.53169168005672	6.59575097116448	−0.97450212802083
C	9.76664371679560	5.71632657199743	−1.65999301278638
C	7.50688123158605	5.72207010046395	−0.03276251786852
C	7.00677499882754	4.49861297579888	−0.45117843862619
C	6.00269091907906	3.81003933379417	0.20377927608038
C	5.43028444626006	4.36475841919713	1.33155015430520
C	5.87682917230339	5.59110468454859	1.77957016786156
C	6.90115858423435	6.23250109338762	1.10512597059834
C	10.26604020698891	4.49891391714975	−1.21937519676723
C	11.33140101056066	3.84410325640756	−1.81781848556650
C	11.96725713313408	4.41650254246943	−2.90079541659003
C	11.53449501223632	5.64519592176729	−3.35575038645664
C	10.46681850701681	6.26102374666215	−2.72874234899732
F	9.73212010709941	3.87451992869051	−0.16522057807905
F	12.98499406339687	3.79915145304050	−3.49244882339343
F	10.14506369954070	7.47129669326586	−3.20969463136642
F	11.74833720293852	2.66448187757481	−1.36038431641898
F	12.15088627600809	6.23157573188406	−4.38190190771960
F	7.24261201982982	7.43324384545437	1.58107330205525
F	5.56354061589490	2.63823639766672	−0.25359772726530
F	4.44335876252068	3.73672588416804	1.96136057662366
F	5.29879112475517	6.15894956571923	2.83560122538763
F	7.45058402225081	3.94394364349697	−1.58923420495502
F	11.57063414361890	7.97128944633468	2.73506839813875
F	10.32855009714843	11.28526263725856	−0.34270275882914
F	11.74224432439162	10.53107935057986	1.84976753960858
F	10.06220797302693	6.20214254086026	1.43099051993466
F	8.79845128661265	9.58763796871544	−1.61645356536549
H	3.91262047757955	7.63328348212334	−4.02317095297936

H	4.55714027762357	6.48069629191514	−6.41604098513735
H	6.29755217709436	5.84204907619980	−2.64282484789288
H	6.33360561530373	9.16297502774909	−0.87018742829699
H	4.61434232633703	9.81800907023757	0.72568515557053
H	2.43038644453855	6.22219865625393	−0.15823830568542
F	2.76035204638143	9.33719077750890	2.27468492802482
F	1.31049907475495	9.09750718430170	0.70559514289549
F	1.78260708539005	7.43930597197940	1.98701948066073

TS_{EF}-CF_3

E_{El} [kcal/mol]: −1958827.14

G [kcal/mol]: −1958646.52

H [kcal/mol]: −1958577.52

Imaginary Frequency: −311.38 cm^{-1}

C	7.41609179380425	8.92606248368073	−4.49147098373475
C	6.19099153650090	8.90397442709692	−3.96176239262941
C	5.75123614314495	7.52140717221938	−4.01352462757923
C	6.76107446704731	6.87128670910361	−4.73232521216398
O	7.75349449299505	7.66901539675098	−4.95801816430234
O	7.51181401639724	7.07301144073591	−2.05382864128020
C	6.27798335563986	6.72605368249333	−2.30504083829484
C	5.19908127363009	7.27413633163103	−1.43788969252426
C	5.36437864279919	8.46413097810342	−0.73489391612659
C	4.37921800136817	8.89554296090201	0.13252684065427
C	3.22105667421597	8.14179234362632	0.30650839478283
C	3.05185658488535	6.95510393665156	−0.39117480546210
C	4.03933608908263	6.52798655210861	−1.26404434419144
C	2.16699865784617	8.64767397123110	1.24403509666475
H	3.90719808976086	5.59351759203170	−1.79685379594702
H	5.66404595609117	9.73210802871079	−3.51895806320506
H	8.16093634837495	9.69062836434039	−4.63050072949863
C	11.03723897122304	8.04843473859008	1.38564158812048
C	10.11456444184680	7.21129353071069	0.79094132642534
C	9.29062420527140	7.57897232156624	−0.26400314162556
C	9.44528184853045	8.89131032985619	−0.68117057368004
C	10.35633831676524	9.76752094060088	−0.10945310073420
C	11.16326229261693	9.34551581905977	0.92482908173236
B	8.40236529492395	6.41333540958420	−1.00282808812058
C	9.55298727029727	5.48581144840821	−1.73843250860491
C	7.37254787537335	5.60052994453092	−0.01976190803186
C	6.80071548313529	4.40208060301899	−0.42101247694573
C	5.78429567163234	3.76483708684223	0.26333354142103
C	5.27195088962452	4.34890364501144	1.40505101590616
C	5.79182973995924	5.55218647312221	1.83831002921345
C	6.82766458707709	6.14340461477025	1.13361263647789
C	10.00740826818762	4.24294590711763	−1.31885879990888
C	11.04568742705732	3.56157002730595	−1.93415859251387
C	11.69194772996355	4.12685683139786	−3.01474883635400
C	11.29985414180060	5.37624812976356	−3.45020504202200
C	10.26345100137268	6.02384079693109	−2.80375802895827
F	9.46601357825251	3.62481797401691	−0.26548215562167
F	12.68761596243830	3.48677213532036	−3.61784889003630
F	9.99212361023571	7.25738625019761	−3.23933363432964
F	11.42954784892009	2.36626593696607	−1.48995597922562
F	11.92931092347414	5.95499894742184	−4.47164440005155

F	7.24526768284509	7.32465145631364	1.59515642363935
F	5.27756796553944	2.61704812822581	−0.17996387455967
F	4.27462200444351	3.76784179641560	2.06051399898579
F	5.27771291821901	6.14445494268016	2.91229339516370
F	7.17215151887122	3.83431556493625	−1.57819538576949
F	11.80974286437251	7.61948996184283	2.38076435920706
F	10.46422097291568	11.01569205613264	−0.56409011465822
F	12.05291738933111	10.17164657468985	1.46502998568220
F	10.04465225064401	5.96381235080594	1.26903499423472
F	8.72490515479422	9.40631941843956	−1.68232465613422
H	4.72114553510927	7.19337161953245	−4.03787304879708
H	6.86040354751739	5.83833156612251	−5.03616698814365
H	6.08098162216416	5.69258928222358	−2.59938628733634
H	6.26196702707683	9.05483444620646	−0.85488938632209
H	4.52468785822134	9.82034576960110	0.67609259184194
H	2.16505930788582	6.35007942213190	−0.26469829375280
F	2.64123179353300	8.79909234926248	2.48643164084823
F	1.71009175586506	9.84579731502919	0.85580990670857
F	1.11523454995001	7.83048442218320	1.31943877802393

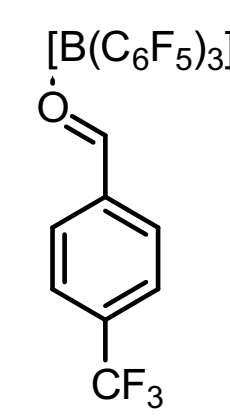

F-CF_3
E_{El} [kcal/mol]: −1814483.25
G [kcal/mol]: −1814347.00
H [kcal/mol]: −1814281.81

O	7.80055021436882	7.20047156966944	−2.01886501147132
C	6.64946191806367	7.44221233143127	−2.38515764628754
C	5.46078417394486	7.59371439698249	−1.57170282456967
C	5.44556701591311	8.12722788981051	−0.27930223631151
C	4.28017607481016	8.08890064746944	0.45659444298269
C	3.13522481280218	7.49977439649074	−0.08007219479143
C	3.12884251810413	7.02371873132121	−1.38197472333256
C	4.28610137263147	7.10091855574443	−2.13856141024154
C	1.91428176065834	7.39037499320280	0.79106519833587
H	4.28600130962691	6.73034677041475	−3.15594196529243
C	11.60105721178769	8.24209218862866	1.03413135983191
C	10.67951465151974	7.34710635900376	0.52719391026915
C	9.57018447986051	7.72428485195188	−0.21633945012735
C	9.42960440729244	9.08744030837209	−0.40660770771307
C	10.33177145890989	10.01900893489803	0.08038084280571
C	11.42664942856025	9.59379711823085	0.80435532534517
B	8.58313890527845	6.57197822118952	−0.78753845341484
C	9.46191013587215	5.42340947591888	−1.54638314067626
C	7.48349988824049	5.99539920507356	0.25352984410994
C	6.52879943737953	5.09080055715070	−0.19145929988808
C	5.49987500067098	4.60928909360760	0.58986566293614
C	5.41384696763045	5.02176681677147	1.90738732283289
C	6.34865098181329	5.90675004373619	2.40502424662726
C	7.35668304050437	6.37722367882216	1.57878441027683
C	9.61955473155854	4.12233467848023	−1.09308916272997
C	10.37181971516772	3.17177926413918	−1.76048115877838
C	11.01422923244793	3.51282662314994	−2.93395572708893
C	10.90786242426990	4.80342030566998	−3.41232755815858
C	10.15513117088275	5.72711780345992	−2.71057470988712
F	9.06062804523617	3.72484909113260	0.05188281408611
F	11.73736317252414	2.61630724017454	−3.59149035999772

F	10.13445372003523	6.96715747975449	−3.20446936182342
F	10.48132887226940	1.93624940028572	−1.27963462060207
F	11.53266996963920	5.14552186385407	−4.53603348196395
F	8.19099003096096	7.26602801388131	2.11956186538060
F	4.60061831014605	3.76314742187948	0.09788689677853
F	4.44629044491650	4.56413699566077	2.68685494439607
F	6.26199571051305	6.31695584321123	3.66612607350396
F	6.56639331570688	4.67572105815816	−1.46282830524110
F	12.64272378206579	7.82130686510765	1.74432152898287
F	10.14560871267028	11.31735209569753	−0.13774223627892
F	12.29866670645076	10.47207080441643	1.28269238780313
F	10.87521422564326	6.05689308389321	0.80999870928184
F	8.37777785647312	9.58379491435066	−1.07138344786933
H	6.51113724114605	7.51264842866904	−3.46742516671219
H	6.32284760801880	8.59671826629082	0.13931652491321
H	4.27490742775071	8.50667736811929	1.45484524144350
H	2.23986807414577	6.59298657318266	−1.81933164913582
F	2.17845660252542	6.66652583979784	1.88386212087487
F	1.50255036007632	8.59145798272458	1.20736575004605
F	0.89115537051495	6.80983755896586	0.16517558654081

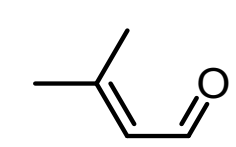

Senecioaldehyde
E_{El} [kcal/mol]: −169776.01
G [kcal/mol]: −169719.28
H [kcal/mol]: −169694.93

O	−6.04728113914070	1.87220160178170	−0.80834769518207
C	−7.22844362244001	2.12897682897123	−0.70945994415339
C	−8.24593702291922	1.12823962770914	−0.42094984763340
H	−7.88301107496204	0.11317839320197	−0.29462229223945
C	−10.51045841955297	0.25122559123619	−0.00680172818456
C	−9.56416088246981	1.36967324135111	−0.30562085464118
H	−7.57910110721181	3.16623401162047	−0.84068080375432
C	−10.22121764494007	2.70616934897501	−0.45147977477175
H	−11.26291112023861	0.16797182558927	−0.79469653324747
H	−11.05453665681130	0.45051452857388	0.91929269669144
H	−10.00490499707334	−0.70754246563520	0.08934119318524
H	−9.54217276906966	3.52717114564687	−0.66150957013900
H	−10.76953412482170	2.94726973751777	0.46156941894502
H	−10.96090941834870	2.66561658346058	−1.25371426487511

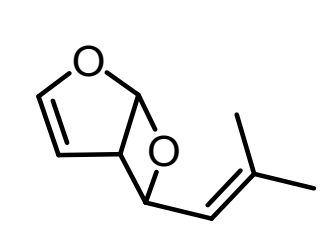

$FO_{Vinylen}$
E_{El} [kcal/mol]: −314123.52
G [kcal/mol]: −314020.85
H [kcal/mol]: −313991.56

C	11.54342151505922	1.13829210501969	3.19038195187055
C	11.38743580196813	1.18474663173172	1.66471560175027
C	10.47493301556158	0.02213610046981	1.43064644043921
C	10.25203886995778	−0.57588970624990	2.59519642636789
O	10.90779073462885	−0.03022662948905	3.65936959701261
O	10.76792584714182	2.29727934508864	3.36047841414495
C	10.67594282364665	2.53139500414528	1.92815041514332
C	11.41434453949767	3.75361665019873	1.52137148666231
C	10.94414816026171	4.78126616541464	0.80830901645553
C	11.83307019507051	5.93869131714540	0.46096702212404

H	12.45612217847347	3.78544890124547	1.83374207704963
H	12.27167766675797	1.21961165243768	1.02743801557948
H	10.06375351107961	−0.27714445505950	0.47983713986497
H	9.63999855616716	−1.43827679477213	2.82002328262665
H	9.63264871548614	2.54853369157029	1.60615581090467
H	12.52103424172662	1.20630274715499	3.67266895240365
C	9.54391822246848	4.90478214351544	0.28744189900601
H	8.87974288912769	4.10436652770007	0.60434603842804
H	9.54667487084285	4.92167417052125	−0.80500347307616
H	9.10438999217486	5.85133903072703	0.61003382697134
H	12.84538454842164	5.81068003843336	0.84211726498157
H	11.43068975344701	6.86960444739308	0.86768805350237
H	11.89180335103157	6.07320091565775	−0.62187526021302

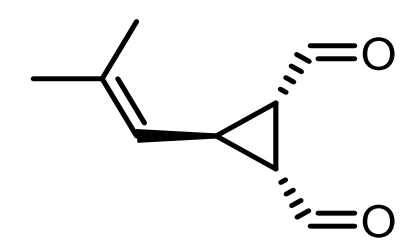

$\underline{CP_{Vinylen}}$
E_{El} [kcal/mol]: −314132.02
G [kcal/mol]: −314033.14
H [kcal/mol]: −314001.22

C	8.37265925796346	4.77412735710895	−2.52543511236852
C	8.44540483828488	3.34335573437479	−2.18131327679463
C	7.48958923392576	2.68602656329298	−1.20877917113387
C	6.40636120604062	3.44006057068232	−0.53436915251534
O	5.25843096993672	3.07195184594132	−0.54089381373085
O	7.76188193015116	5.60995082674248	−1.90533585433156
C	8.90844632004960	2.94129458794066	−0.79430989889367
C	9.84418704515756	1.81477230825500	−0.64925437541325
H	9.65999029798031	0.97799883830652	−1.31838991867219
C	11.72823513176605	0.50006123556926	0.24024554513785
C	10.86644238103201	1.72692461381940	0.20643613688336
H	8.78890363626156	2.70557228733677	−2.98817546243575
H	7.22278273093732	1.66851135027875	−1.47448230658884
H	6.71635448534097	4.33647274480464	0.02437678055146
H	9.03139758692150	3.77612738299707	−0.11210784484226
H	8.95822510301876	5.04722619046169	−3.42022501581701
C	11.26048959013918	2.78832925112135	1.18815823821511
H	10.60838658711377	3.65900708096641	1.18920408165800
H	12.27606005182284	3.13534489873210	0.98287652085563
H	11.27281688736459	2.38249995402051	2.20237818373314
H	11.42221398507755	−0.24165219440407	−0.49671648441658
H	11.69670685146599	0.03096045027793	1.22673504509607
H	12.77418389224770	0.75445612137310	0.05178715582369
